# High resolution class I *HLA-A, -B*, and -*C* diversity in Eastern and Southern African populations

**DOI:** 10.1038/s41598-025-06704-4

**Published:** 2025-07-02

**Authors:** Alabi W. Banjoko, Tiza Ng’uni, Nitalia Naidoo, Veron Ramsuran, Ollivier Hyrien, Zaza M. Ndhlovu

**Affiliations:** 1 Africa Health Research Institute (AHRI), Nelson R. Mandela School of Medicine, Durban, South Africa; 2 Ragon Institute of Massachusetts General Hospital, Massachusetts Institute of Technology, and Harvard University, Cambridge, MA USA; 3 School of Laboratory Medicine and Medical Sciences, College of Health Sciences, University of KwaZulu-Natal, Durban, South Africa; 4 Biostatistics, Bioinformatics and Epidemiology Program, Fred Hutchinson Cancer Center, Vaccine and Infectious Disease Division, Seattle, USA; 5 Department of Statistics, University of Ilorin, Ilorin, Kwara State Nigeria

**Keywords:** Africa, HLA, Alleles, Haplotypes, Diversity indices, Haplotypes, Genetics, Population genetics, Genetic variation

## Abstract

Africa, being one of the most genetically diverse regions in the world, remains significantly underrepresented in high-resolution Human Leukocyte Antigen (HLA) data. The extensive genetic variation in HLA alleles across the region underscores the need for population-specific immunogenetic data to guide T-cell vaccine development. This study analysed Class I HLA data from Eastern and Southern African populations to assess regional genetic diversity. Analyses included allele and haplotype frequency distributions, deviations from Hardy–Weinberg equilibrium, linkage disequilibrium, and homozygosity test of neutrality across various populations. To further contextualise African HLA diversity, comparisons were made among African populations and also with African American and European American populations using the Hellinger diversity index and multidimensional scaling methods. The results revealed that South African populations exhibited an estimated average of 34.1% genetic diversity with respect to other African populations. Rwanda demonstrated an estimated 26.9% genetic diversity, Kenya (26.5%), Zambia (26.5%), and Uganda (24.7%). Additionally, in-country analyses revealed variations in HLA diversity among different tribes within each country. The estimated average in-country diversity was 51% in Kenya, 35.8% in Uganda, and 33.2% in Zambia. These results reveal various levels of genetic diversity among African populations. The highlighted differences in HLA Class I allele frequencies between Eastern and Southern African populations compared to US populations, demonstrate that it is inappropriate to extrapolate HLA data from US populations including that of African Americans when designing T-cell-inducing vaccines tailored to African populations. Our findings underscore the urgent need to generate high-resolution HLA data to guide vaccine development tailored to African populations.

## Introduction

The HLA complex consists of highly polymorphic genes that code for surface proteins responsible for antigen presentation to T cells as part of the immune response to infections^1^. According to the IPD-IMGT/HLA database, more than 40,000 HLA alleles have been identified and the total HLA allele variation is estimated to be several millions across different populations around the world^2–4^. Africa, often referred to as the cradle of humankind^5,6^, exhibits some of the highest levels of human genetic diversity globally^7^. This rich genetic diversity results from the continent’s long evolutionary history, complex demographic processes and genetic admixture that have shaped its populations over time^6,8^. However, population data sets in some of the databases such as the Allele Frequency Net Database (AFND), which provide the scientific community with a freely available repository for the storage of frequency data including alleles, genes, haplotypes, and genotypes have reported minimal HLA frequency data for African populations^9–11^. Moreover, many ethnic populations in Sub-Saharan Africa are underrepresented in medical genomics studies due to limited research, particularly on *HLA* alleles, compared to developed countries^12^. This discrepancy in *HLA* allele data is also reflected in the IPD-IMGT/HLA database, where most submissions originate from Europe, America, and Australia (IMGT/HLA Database, released in July 2024, https://www.ebi.ac.uk/ipd/imgt/hla/about/statistics/world/). This indicates a significant lack of HLA typing infrastructure in Sub-Saharan Africa, further contributing to the scarcity of HLA data for these populations.

The *HLA* genes have been widely studied for years due to their extensive allelic variability across diverse populations and their importance in host immune responses, immunotherapy and organ transplantation^2,10,13–15^. In addition, some *HLA* alleles have been associated with either protection against or susceptibility to a wide range of autoimmune and infectious diseases, drug-induced hypersensitivity and cancer^16^. For instance, HLA Class I alleles such as HLA-B*27, HLA-B*52, HLA-B*57 and HLA-B*81 have been linked to protection against HIV disease progression (protective alleles) whereas HLA-B*35, HLA-B*51:01, and HLA-B*58:02 have been linked to rapid disease progression (disease-susceptible alleles)^17,18^. Notably, HLA alleles and haplotypes do not occur at the same frequency in different populations. For example, in European Americans, HLA-B*58:02 which is linked to HIV disease susceptibility is mainly absent^19^. In contrast, the HLA-B*58:02 allele is highly prevalent in the African population^17^. Similarly, the protective allele HLA-B*57:01 is highly prevalent in the European American population and largely absent in the African population^17,20,21^.

Leveraging HLA diversity data can lead to more tailored therapies and inform the rational design of T cell-based vaccines that will be efficacious across diverse populations. This study used population genetics approaches to understanding Class I HLA (genetic) diversity in the eastern and southern African regions. The study provides insight into the extensive diversity of the allelic and haplotype frequencies within five African populations compared to the African American and European American populations. This study primarily focuses on HLA Class I loci due to their extensive polymorphism reflected in the higher absolute number of distinct alleles compared to Class II loci. However, HLA Class II loci exhibit a unique form of diversity through the formation of heterodimeric proteins resulting in combinatorial numbers of expressed Class II HLA proteins that present many possible functional molecules, which, in aggregate, may rival or even exceed the diversity seen in Class I proteins^22^. Future work will extend the current study to include Class II loci to better understand HLA diversity and its implications for disease susceptibility and vaccine development across diverse African populations.

## Results

The African sub-regions represented in this study include Eastern and Southern Africa. The Eastern African populations consist of individuals from Kenya, Rwanda, Uganda and their respective ethnic groups, while the Southern African populations include individuals from the Republic of South Africa (RSA), Zambia, and their associated tribes.

### Genetic diversity between African and U.S. populations

**Fig. 1:**
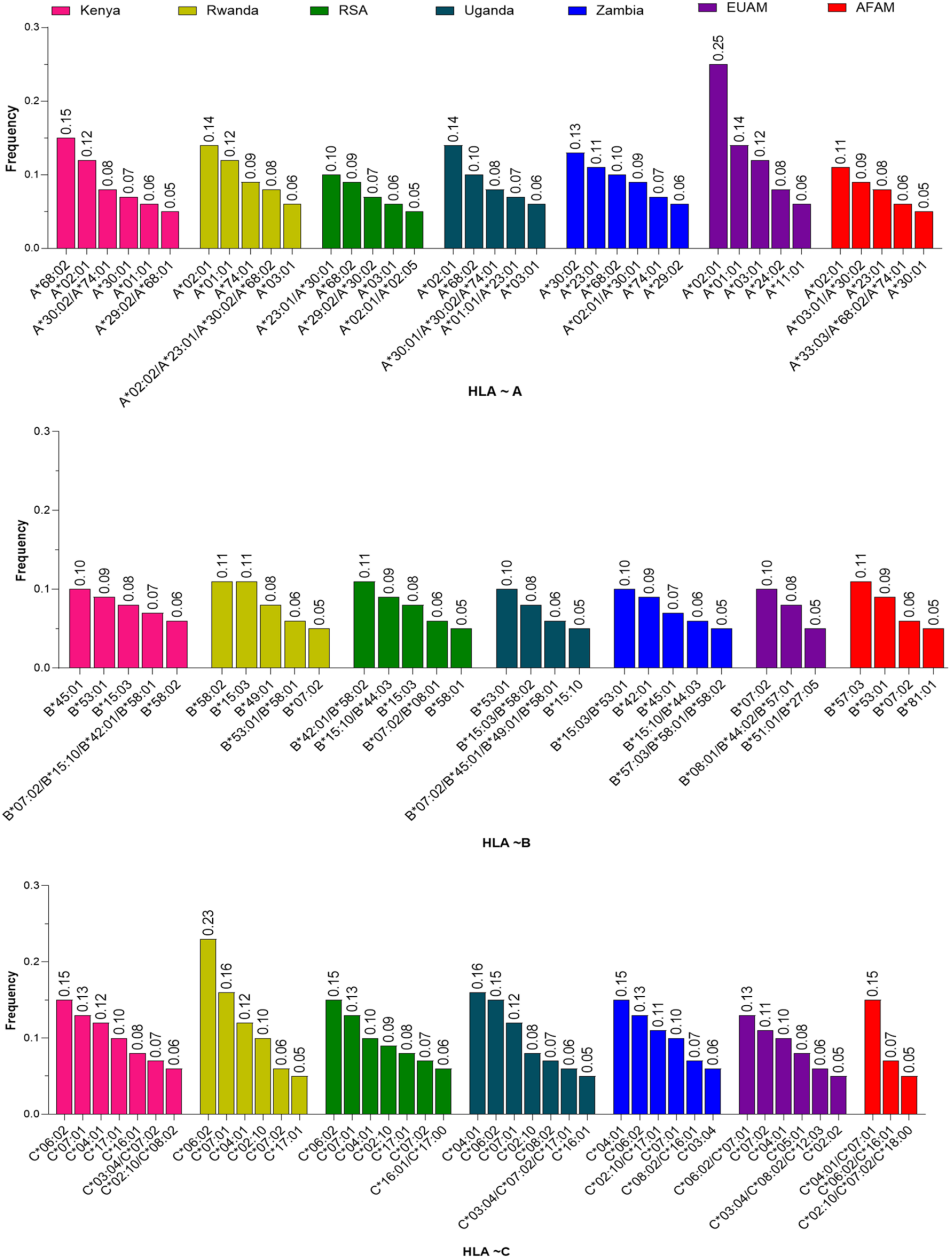
Most frequent ($\ge$ 5%) HLA alleles within each population. The figure shows the graph of the top 5% most frequent alleles in each population. The different colours indicate different populations as shown in the legend. All the three graphs in the figure were plotted using the same scale on the y axis. The graph provides an overview of the frequencies of common alleles at each locus. The distinction in allele frequencies testifies to HLA genetic diversity among the populations. HLA-B alleles have relatively lower frequencies due to the greater overall number of HLA-B alleles in these populations, so fewer of them can achieve frequencies over 5% (see supplementary Table 2 for full list of alleles and associated frequencies). The presentation of allele frequencies ($\ge 5$% as high prevalence) follows a general and widely accepted framework commonly used in population genetics and HLA studies and is intended to facilitate a concise graphical representation of allele frequency distributions in this study. Additionally, combining two or more allele frequency distributions into a single bar serves to conserve space and reflects the fact that the corresponding alleles exhibit approximately similar frequencies at the locus of interest within the same population.

We compared HLA data from the African sub-regions to the African American and European American populations to address the extent of HLA differences between African and US populations. Although, African American and European American HLA studies have received some attention in the literature^23–26^, this study demonstrated that HLA data from the US populations cannot represent the African HLA population data. In this study, we computed allele frequencies across all populations to help identify complex genetic traits and discover HLA disease associations^27,28^. Frequencies of alleles were estimated by direct counting and estimation of their respective proportions. The complete list of alleles and their frequencies across populations is detailed in Supplementary Tables 1–3 . Allelic frequency distributions vary across populations. Allele frequencies are either high $(\ge 5\%)$, low $(<1\%)$ or intermediate $(1\%$ to less than $5\%)$. The Allele frequencies were sorted in descending order within each population and alleles with frequencies of at least 5% were plotted and presented in Fig. 1 for all loci. Comprehensive stacked bar plots illustrating the proportion of alleles at each locus across the different populations are presented in Supplementary Figs. 8, 9, and 10. While the Common, Intermediate, and Well-Documented (CIWD) catalogue provides a standardized framework for categorizing alleles into Common, Intermediate, and Well-Documented groups^29^, specific studies may apply different frequency thresholds depending on their research objectives and the populations under investigation^30–33^.

#### HLA-A

HLA-A*02:01 ranks among the top 5% most frequent alleles across all populations studied, exhibiting varying high-frequency levels, with the highest observed in the European American population at 25%. This allele was relatively low in the South African (RSA) population with a frequency of 5.1% as presented in Fig. 1. Also, HLA-A*68:02 was observed at a relatively high frequency (> 8%) in the African populations but at a low frequency (6%) in the African American population. Interestingly, this allele was not among the top 5% in the European American population. HLA-A*68:01, A*02:02, A*02:05, and A*11:01 were among the top 5% most frequent alleles, with unique distributions across specific populations. HLA-A*68:01 was observed in the Kenyan population (5%), A*02:02 in Rwanda (8%), A*02:05 in South Africa (RSA, 5%), and A*11:01 in the European American population (6.4%). These alleles were uniquely observed among the top > 5% of most frequent alleles in each country. These observations highlight the diverse allele frequencies across populations, reflecting genetic variation in HLA diversity. (Fig. 1 and Supplementary Table 1).

#### HLA-B

HLA-B*15:03, HLA-B*58:01, and HLA-B*58:02 were among the top 5% most frequent alleles across all populations albeit at varying frequency levels except in the U.S. populations, where they were notably absent or less prominent (Fig. 1). HLA-B*57:03 was only present in African American (10.8%) and Zambia (5.3%) populations among the top 5% frequent alleles. HLA-B*27:05 and HLA-B*57:01 were only present in the European American population, whereas HLA-B*81:01 was only present in the Africa American population among the top 5% of frequent alleles. HLA B*07:02 was among the top 5% frequent alleles in all populations except in Zambia (Fig. 1, Supplementary Table 2).

#### HLA-C

At the HLA-C locus, HLA-C*04:01, HLA-C*06:02, and HLA-C*07:01 were consistently observed among the top 5% most frequent alleles across all populations (Fig. 1). In contrast, HLA-C*02:10 ranked within the top 5% only in the African and African American populations but was not among the top 5% alleles in the European American population. Also, HLA-C*17:01 was among the top 5% alleles only in the African populations. In the European American population, HLA-C*05:01 and HLA-C*12:03 were present at 8% and 6% respectively but were not among the top 5% of frequent alleles in other populations **(**Fig. 1, Supplementary Table 3).

**Fig. 2:**
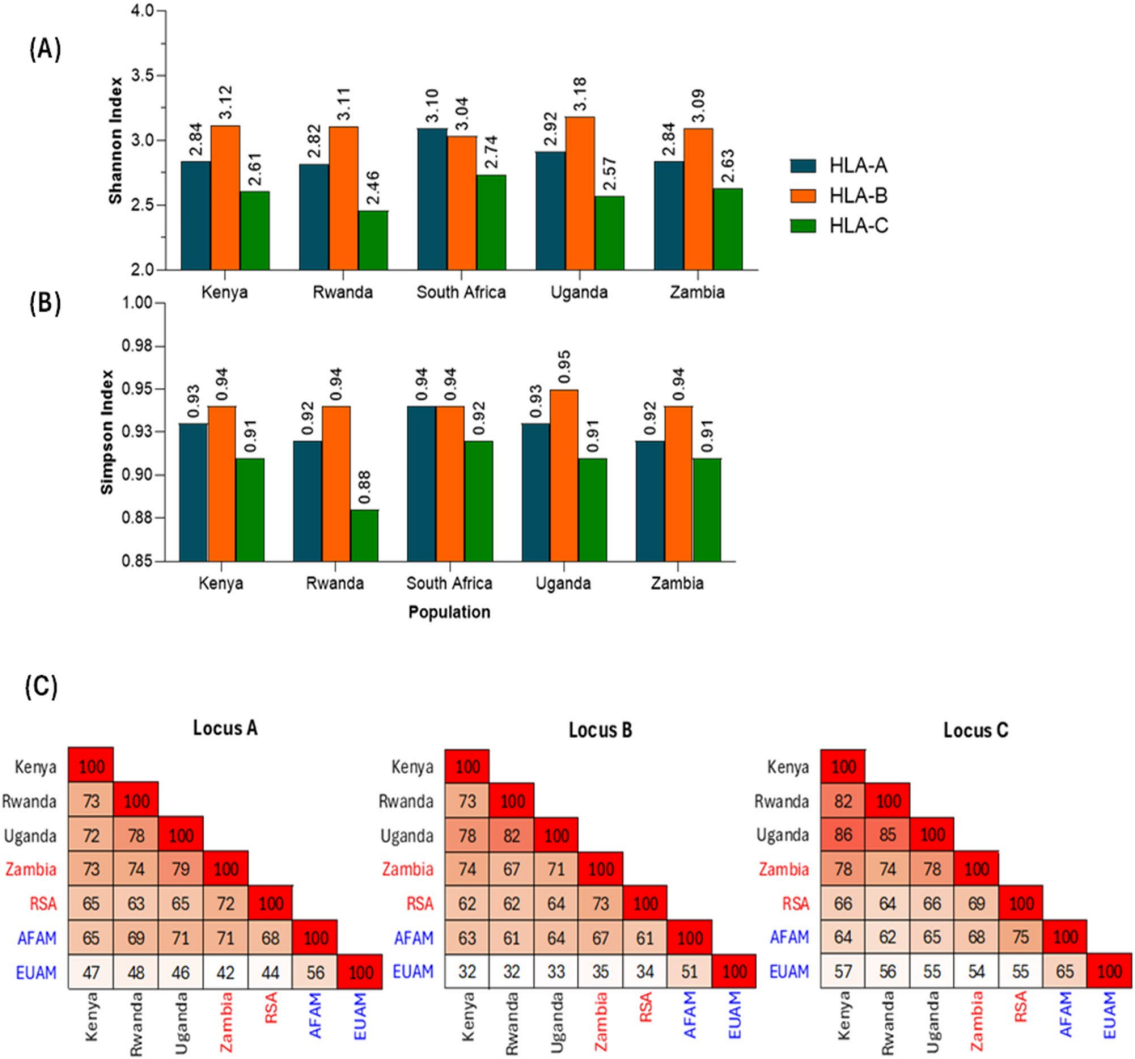
Graphs of Shannon (**A**), Simpson (**B**) indices across African populations and (**C**) non-clustered heatmap of similarity index (Hellinger Distance) among populations. Panels A and B explain the in-country diversity. The higher the index values the greater the population diversity at that locus. Panel C quantifies the genetic similarities (in %) among populations. The darker the colour, the more similar the two populations involved.

The Shannon and Simpson indices were determined at each locus and across populations (Supplementary Fig. 1 for African tribes). Figure 2A summarizes the Shannon index which accounts for allele type richness and evenness of their abundance. Figure 2B also summarizes the Simpson index which accounts for the probability that two alleles taken from the sample at random are of different types. The Hellinger distance (HD) index was used to quantify the similarity between two populations regarding allele composition and genetic makeup relative abundance. The HD index was obtained by determining the relative frequency of alleles in any two populations. The structure of the allele frequencies was then used to determine the HD diversity, which was used to obtain the similarity indices, converted into percentages and drawn as a non-clustered heat map. The darker the colour in the heatmap, the more similar the corresponding populations. Figure 2C shows the low level of genetic similarities between the African and US populations. The European American population had the lowest similarity index to the African populations in all the HLA loci considered. Similarly, the African American population showed relatively higher similarity indices to the African populations than the European American population in all loci.

**Fig. 3:**
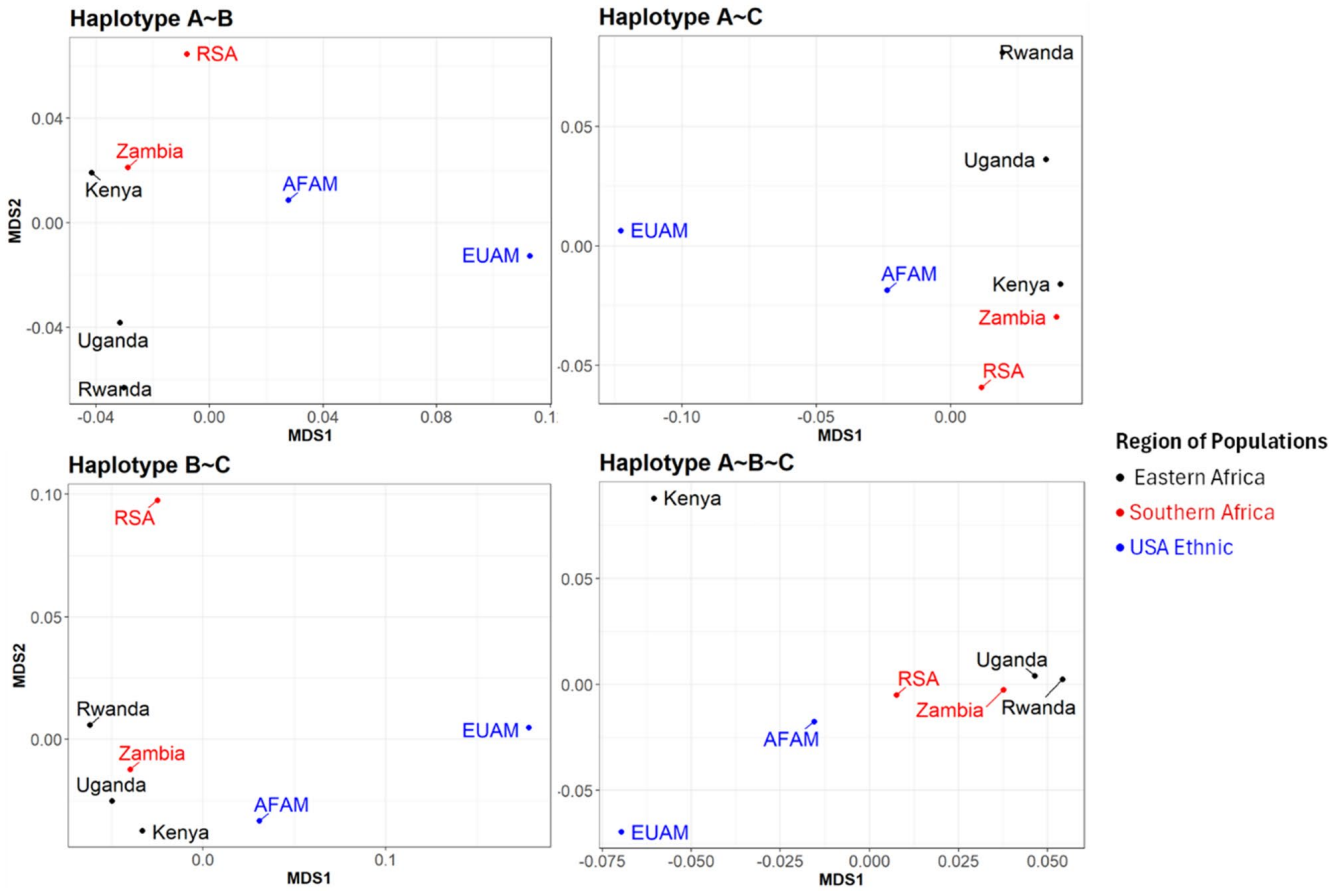
Cartography of the genetic distance in global haplotypes between populations. The figure visualizes the genetic distance between African and US ethnic populations.

In addition to the individual *HLA* alleles, the study determined the extent to which haplotypes (specific combinations of alleles inherited on the same chromosome) overlap between populations. This was determined using multidimensional scaling (MDS) to visualize the genetic distances (cartography) at all the haplotype loci. A similar analysis was carried out using an unrooted phylogenetic tree approach to complement the 2-dimensional cartography (Supplementary Fig. 11). Analysis was carried out on the relative frequency of haplotypes in each population relative to others. Haplotype frequencies from each population’s data were dimensionally reduced using MDS to create a 2-dimensional genetic cartography. Based on the analysis, two countries are close to each other on the map if the distribution of the haplotypes in these two populations is close to one other, relative to the distribution observed in the other countries. In Fig. 3, the African American population is relatively closer to the African populations than the European American population which is farther away from the African populations at all haplotypes.

### Genetic diversity within African populations

To better define HLA genetic diversities among African populations at both the country and tribal levels, we computed within-population diversity estimates using the Shannon and Simpson diversity indices. In Figs. 2A and 2B, all populations present the natural polymorphic structures of the HLA alleles except for South Africa wherein *HLA–A* is suggested to be slightly more evenly distributed than *HLA–B*. Generally, the higher values observed in all populations at different loci indicate a high level of genetic diversity within each of the African populations. A similar trend was observed in the tribal populations of each country in the African sub-region (Supplementary Figs. 2A and B). Similarly, the HD similarity index in Fig. 2C presents a diversity index to compare the HLA differences between African populations.

The highest values of the HD similarity index among the eastern African countries were observed between Rwanda and Uganda with similarity indices of 78%, 82% and 85% at locus A, B and C respectively (Fig. 2C). South Africa and Zambia had similarity indices of 72%, 73% and 69% at locus A, B and C respectively. Interestingly, Uganda and Zambia had the highest similarity index of 79% at locus A. Conversely, among the African countries, South Africa had the lowest similarities with other African countries at all loci except with Zambia at locus B where the similarity index was relatively higher (73%). At the tribal level (Supplementary Fig. 2C), the HD similarity index also shows various values of similarity indices across tribes within the African countries. The Kikuyu tribe showed relatively low $(38\%\le \text{HD}\le 69\%)$ similarity to other African tribes at all loci. It was also observed that despite belonging to the same African country, Kikuyu and Luhya tribes had low similarity of 47% and 38% at loci A and B respectively. Generally, the similarity between most tribes is less than 70% at all loci except for a few tribes across the African countries.

Also, the cartograph (Fig. 3) shows that at B:C, South Africa (RSA) was observed to be farther away from other African countries. Similarly, Kenya is seen to be far from the rest of the populations at A:B:C. Kenya and Zambia (from different geographical regions) were observed to be closer at A:B and A:C, while Uganda and Rwanda were closer at A:B and A:B:C. We also observed close genetic distances among Kenya, Zambia and Uganda at B:C, whereas Uganda and Rwanda were relatively closer at A:B:C. Furthermore, the cartograph at tribal level as presented in Supplementary Fig. 3 shows that the genetic distances between the Kenyan tribes (Kikuyu and Luhya) are farther apart from each other at all loci. The Ugandan tribes in this study (Muganda, Munyankole and Munyarwanda) were observed to be far apart from each other at all loci except for B:C. The Lozi tribe in Zambia did not exhibit the same genetic closeness as other Zambian tribes at all loci (Supplementary Fig. 3). Finally, the Zulu tribe from South Africa exhibited genetic closeness to tribes in Zambia and Uganda at all loci except B:C where it diverged from other African tribes.

Rarefaction and extrapolation curves were employed to evaluate completeness of samples and uniqueness of alleles for each population (country and tribe) as a function of the number of participants. This was determined by creating a subsample of size $n$ and counting the number of unique HLA alleles included in the subsample for any given (HLA alleles, population) pair. Subsampling was done at random without replacement and was repeated for different values of $n=1, ..., {N}_{p}$, where ${N}_{p}$ denotes the number of participants from the selected population. We then plotted the number of unique HLA alleles as a function of the number of participants by population. Table 1 below reports the number of unique alleles and associated relative frequency at different HLA loci across populations.

**Table 1:** Number of unique Class I alleles according to country of subjects.

Populations	No of participants	No of unique alleles			Frequency of unique alleles (%)		
		HLA-A	HLA-B	HLA-C	HLA-A	HLA-B	HLA-C
Kenya	109	25	37	22	11.5	17.0	10.1
Rwanda	173	32	35	21	9.2	10.1	6.1
South Africa	1640	48	56	36	1.5	1.7	1.1
Uganda	231	32	41	21	6.9	8.9	4.5
Zambia	565	37	46	28	3.3	4.1	2.5
European American	1765	61	101	40	1.7	2.9	1.1
African American	661	50	78	40	3.8	5.9	3.0

The table shows number of unique alleles in each population at different loci and their respective relative frequencies.

The rarefaction and extrapolation curves of HLA-A, HLA-B and HLA-C (Supplementary Fig. 4) show the allele richness across different African populations. The rarefaction curve indicates current diversity estimates, while the extrapolation curve suggests how much more diversity might be discovered with larger samples. The Shaded areas represent the 95% confidence intervals around the diversity estimates. Wider intervals suggest greater uncertainty, usually due to smaller sample sizes. The dashed lines show extrapolated diversity i.e., predictions about what the diversity would be if more individuals were sampled. The curves demonstrate a characteristic pattern: a rapid initial increase as common alleles are observed, followed by a plateau as only a few rare alleles remain to be detected. This trend reflects the underlying allelic diversity structure at each HLA locus, with the shape of each curve providing insight into the richness and evenness of allele distributions across populations^34^. HLA-B had the highest allelic diversity in all populations (countries and tribes), followed by HLA-A, and HLA-C had the least allelic diversity. Visual inspection of the curves also supports the genetic diversities (number of unique alleles) observed in Table 1 across the African countries. Kenya is observed to have the highest (%) allelic diversity at all loci, followed by Rwanda, Uganda and Zambia, while South Africa has the least (%) diversity at all loci among the African countries.

### HLA alleles linked to immune responses and disease outcomes

In addition to analyzing overall HLA diversity, we investigated immune- and disease-associated alleles at each locus to identify key differences in HLA variants linked to immune responses and disease outcomes across populations. This analysis enhances our understanding of the genetic basis of immunity and its relevance to diseases such as HIV/AIDS, with implications for improving diagnostics, treatment, and preventive strategies^35,36^. The Bw4 or Bw6^37^ allele group are epitopes located on most HLA-B and a few HLA-A proteins, playing a critical role in immune recognition^38^. Additionally, alleles may be classified as protective or disease-susceptible, depending on population-specific associations with immune outcomes^37–41^. This study examined the frequencies of known immune- and HIV-associated HLA alleles across the populations (Table 2). Notably, Bw4 alleles at loci A and B were either absent or present at extremely low frequencies in all populations. HLA-A*74:01 (a protective allele) appeared at relatively high frequencies in Kenya, Rwanda, Uganda, Zambia, and African Americans, but at lower frequencies in South Africa, and was very rare in European Americans. HLA-A*25:01 was absent in all African populations and occurred at a very low frequency in the African American population. The HIV-susceptible allele HLA-A*36:01 was observed at low frequencies in South African and European American populations compared to others. HLA-A*24:03, a Bw4 allele, was not observed in any African population but was found in U.S. ethnic groups. Although HLA-B*39:01 was detected in the Zambian population, it occurred at an extremely low frequency. The allele was absent in the other African populations but was observed at a frequency above 1% in the European American population. This pattern suggests that HLA-B*39:01 may be largely absent or extremely rare in African populations. Furthermore, the protective allele, HLA-B*27:05 was found at low frequencies in South Africa, but more commonly in African American and European American populations. HLA-B*42:01 and B*44:03 showed high frequencies in both South African and Zambian populations. HLA-B*52:01 was only observed at low frequencies in Kenya and South Africa. HLA-B*57:01, although rare in African populations, was found at a notably high frequency (7.9%) in European Americans. Similarly, among HIV-susceptible alleles, HLA-B*35:01, B*35:02, and B*35:03 were generally rare in African populations, with particularly low levels in South Africa. HLA-B*07:02 appeared at a relatively high frequency in Kenya among the African populations, but with the highest overall frequency in the European American population. HLA-B*08:01 was found at higher frequencies in European Americans and also relatively elevated in South Africans compared to other African populations. HLA-B*58:02 showed significantly higher frequencies in African populations, particularly in South Africa (11.4%) and Rwanda (11.3%), compared to the U.S. populations.

**Table 2:** Grouping of alleles and allele frequencies among populations at different loci.

Locus	Groups	HLA	Population						
			Kenya	Rwanda	South Africa	Uganda	Zambia	EUAM	AFAM
A	BW4	A*24:03			0.0003			0.0037	0.0023
	Protective	A*25:01						0.0292	0.0061
		A*32:01		0.0029	0.0043	0.0152	0.0035	0.0462	0.0166
		A*74:01	0.0780	0.0896	0.0366	0.0844	0.0690	0.0006	0.0575
	HIV disease-susceptible	A*36:01	0.0275	0.0318	0.0043	0.0433	0.0460	0.0014	0.0212
B	BW4	B*51:02							0.0008
	BW6	B*39:01					0.0009	0.0105	0.0045
		B*39:02						0.0003	
	Protective	B*13:02	0.0138	0.0145	0.0162	0.0108	0.0106	0.0283	0.0129
		B*14:02	0.0321	0.0318	0.0125	0.0346	0.0327	0.0456	0.0272
		B*27:05			0.0015			0.0501	0.0144
		B*42:01	0.0734	0.0405	0.1079	0.0433	0.0912	0.0011	0.0378
		B*44:03	0.0275	0.0347	0.0918	0.0216	0.0646	0.0473	0.0484
		B*52:01	0.0046		0.0003			0.0193	0.0197
		B*57:01			0.0003	0.0022	0.0018	0.0793	0.0106
		B*57:02	0.0092		0.0079	0.0130	0.0097	0.0014	0.0129
		B*57:03	0.0413	0.0347	0.0229	0.0390	0.0531	0.0105	0.1082
		B*58:01	0.0688	0.0607	0.0503	0.0584	0.0549	0.0133	0.0416
		B*81:01	0.0183	0.0260	0.0418	0.0433	0.0257	0.0006	0.0522
	HIV disease-susceptible	B*07:02	0.0734	0.0549	0.0561	0.0563	0.0336	0.0952	0.0552
		B*08:01	0.0046	0.0173	0.0616	0.0238	0.0301	0.0756	0.0371
		B*18:01	0.0183	0.0289	0.0354	0.0346	0.0319	0.0428	0.0242
		B*35:01	0.0321	0.0318	0.0177	0.0238	0.0354	0.0456	0.0386
		B*35:02		0.0029	0.0015	0.0022	0.0009	0.0079	0.0015
		B*35:03			0.0003			0.0162	0.0023
		B*45:01	0.1009	0.0491	0.0299	0.0628	0.0735	0.0077	0.0303
		B*51:01	0.0092	0.0173	0.0085	0.0152	0.0204	0.0527	0.0250
		B*53:01	0.0872	0.0636	0.0183	0.0974	0.0973	0.0096	0.0930
		B*58:02	0.0550	0.1127	0.1143	0.0801	0.0531	0.0006	0.0235

### Genetic basis for observed differences

To further understand the underlying basis of the observed genetic diversity across different populations, this study employed several population genetic tools: Hardy–Weinberg Equilibrium (HWE), homozygosity-based neutrality tests, haplotype estimation, asymmetric linkage disequilibrium (ALD) analysis, and inheritance patterns of alleles across loci. These tools are foundational in population genetics and provide critical insights into allele frequency distribution, population structure, and evolutionary forces shaping genetic variation^42,43^. HWE and homozygosity neutrality tests were performed for each African population at different HLA loci. Significant deviations from expected HWE proportions were observed, notably at the HLA-C locus in South African populations (Table 3) and at the HLA-B locus in the Ngoni tribe (Supplementary Table 10), suggesting potential effects of selection, population structure, or non-random mating. Similarly, the homozygosity test of neutrality revealed significant departures from neutrality at locus A in Kenya, South Africa, and Uganda, and at locus B in Rwanda and Uganda (Table 4). At the tribal level, deviations were observed at locus B for the Muganda, Nsenga, and Tumbuka tribes, and at locus C for the Chewa and Muganda tribes (Supplementary Table 11). These findings may reflect unique evolutionary pressures or demographic histories affecting specific loci within populations or subgroups.

**Table 3:** Exact test using Markov chain for HWE parameters for the five countries.

Country	No of genotype	Locus								
		A			B			C		
		Obs. Het	Exp. Het	P-value (Adj)	Obs. Het	Exp. Het	P-value (Adj)	Obs. Het	Exp. Het	P-value (Adj)
Kenya	109	0.9358	0.9299	0.8451	0.9633	0.9470	0.4505	0.9174	0.9143	0.6548
Rwanda	173	0.9249	0.9256	0.3050	0.9364	0.9459	0.8707	0.8786	0.8855	0.6548
RSA	1640	0.9348	0.9426	0.3750	0.9323	0.9355	0.4180	0.9012	0.9172	0.0155*
Uganda	231	0.9351	0.9330	0.8324	0.9351	0.9499	0.7771	0.9048	0.9071	0.6548
Zambia	565	0.9221	0.9252	0.1445	0.9469	0.9424	0.4180	0.9062	0.9098	0.6390

*Statistically significant. Obs. Het, observed heterozygosity; Exp. Het, expected heterozygosity.

**Table 4:** Slatkin’s implementation of the EW homozygosity test of neutrality for the five African countries.

Country	Locus											
	A				B				C			
	Obs. (Homo) F	Exp. (Homo) F	Fnd	p-value (Adj)	Obs. (Homo) F	Exp. (Homo) F	Fnd	p-value (Adj)	Obs. (Homo) F	Exp. (Homo) F	Fnd	p-value (Adj)
Kenya	0.0744	0.1238	− 1.2527	0.0430*	0.0574	0.0764	− 0.9347	0.1231	0.0899	0.1431	− 1.1121	0.0591
Rwanda	0.0771	0.1060	− 0.8577	0.1502	0.0568	0.0955	− 1.3298	0.0338*	0.1171	0.1703	− 0.8657	0.1440
RSA	0.0577	0.1111	− 1.4135	0.0240*	0.0648	0.0940	− 0.9500	0.1231	0.0831	0.1492	− 1.1969	0.0503
Uganda	0.0690	0.1147	− 1.2026	0.0430*	0.0522	0.0861	− 1.3086	0.0338*	0.0948	0.1816	− 1.2900	0.0503
Zambia	0.0756	0.1204	− 1.0813	0.0776	0.0584	0.0945	− 1.1905	0.0583	0.0910	0.1613	− 1.1671	0.0503

*Statistically significant.

Haplotype and ALD analyses were performed to characterize the patterns of alleles inherited on the same chromosome and evaluate the non-random associations between alleles at different loci. These analyses offer a more comprehensive view of genomic architecture and historical recombination events within populations^44–46^.

The most frequent two- and three-locus haplotypes are summarized in Supplementary Table 12, with the complete list provided in Supplementary Tables 4–7. In South Africa and Zambia, the top haplotypes were A*30:01 ~ B*42:01, A*30:01 ~ C*17:01, and A*30:01 ~ B*42:01 ~ C*17:01. In Rwanda and Uganda, haplotypes such as A*02:01 ~ B*15:03 and A*02:01 ~ B*15:03 ~ C*02:10 were observed at similarly high frequencies. Interestingly, both South Africa and Rwanda shared the same dominant haplotype at the B:C locus pair (B*58:02 ~ C*06:02). However, across all populations, distinct top three-locus haplotypes were identified, indicating region-specific evolutionary dynamics.

**Fig. 4:**
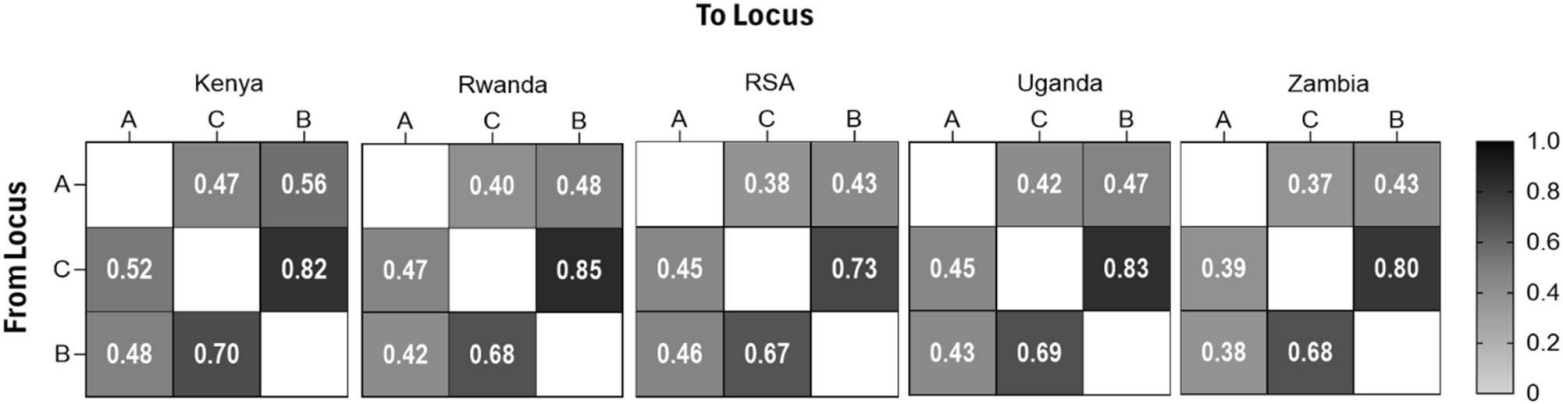
Linkage Disequilibrium plots based on asymmetric linkage disequilibrium measures (row gene conditional on column gene) for HLA genes across countries.

Figure 4 displays heatmaps of asymmetric linkage disequilibrium (ALD) values among the HLA-A, HLA-B, and HLA-C loci across five African populations. The ALD metric quantifies non-random associations between alleles at different loci, incorporating both the magnitude and directionality of these associations through conditional entropy. Strong LD signals were observed in the directional associations from HLA-B to HLA-C (ALD B → C) and the strongest LD from HLA-C to HLA-B (ALD C → B), with values ranging from 0.67 to 0.70 and 0.73 to 0.85, respectively, across the populations. These results suggest a consistently strong, bidirectional dependency between HLA-B and HLA-C alleles. In contrast, moderate to weak LD were observed in other pairwise directions: from HLA-A to HLA-B (ALD A → B: 0.43–0.56), HLA-A to HLA-C (ALD A → C: 0.37–0.47), HLA-B to HLA-A (ALD B → A: 0.38–0.48), and HLA-C to HLA-A (ALD C → A: 0.39–0.52). These patterns reflect variable, and generally weaker, directional associations between HLA-A and the other Class I loci across all populations. A similar pattern of results was also observed across the tribal populations (Supplementary Fig. 1).

These analyses underscore the complexity and population-specific nature of HLA genetic diversity in African populations, which may affect immune function, disease susceptibility, and vaccine development.

Additionally, this study investigated alleles that appeared unique to specific populations (Supplementary Tables 8 and 9). While these alleles were observed exclusively in certain groups based on the current sample sizes, it is essential to note that these findings may be provisional. Broader studies with larger and more diverse population samples may reveal wider allele distributions.

**Fig. 5:**
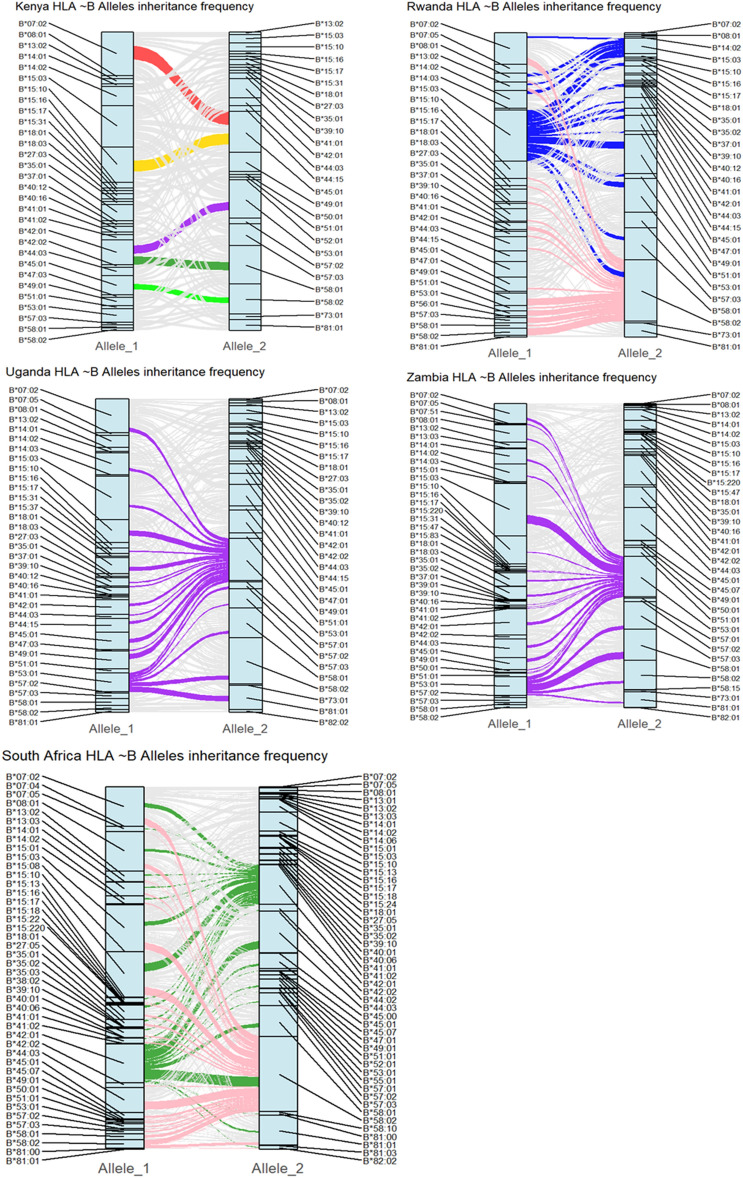
Alluvial plots showing frequency of the inheritance pattern of HLA ~ B alleles in each country. Each box represents the frequency of corresponding alleles. The wider the box, the greater the frequency of the corresponding allele. A wide box linking allele_1 to allele_2 indicates the alleles are highly inherited together at that locus. Narrow boxes may suggest rare allele inheritance pattern.

Furthermore, Alluvial plots were employed to present the inheritance patterns of alleles observed in this study. Each block size in the alluvial plot represents the frequency of the corresponding alleles and the thickness of the flow streams denotes the frequency of the allele inheritance pattern. These provide an understanding of predicting the likelihood of inheriting specific traits or conditions^47,48^. Figure 5 highlights the inheritance patterns of HLA-B alleles as observed in the African populations (Supplementary Figs. 5, 6, and 7 for complete list). In Kenya, HLA-B*07:02 was more frequently co-inherited with HLA-B*45:01 than with other alleles, suggesting a potential haplotypic or ancestral linkage between these two alleles in the population. Additionally, both copies of HLA-B*45:01 were frequently inherited alongside HLA-B*15:10 and HLA-B*58:02, suggesting a recurring pattern of co-occurrence that may reflect underlying linkage disequilibrium within the Kenyan population. Similarly, HLA-B*42:01 demonstrated notable co-inheritance with HLA-B*53:01 and HLA-B*58:01, indicating the presence of shared haplotypes or possible population-specific selective pressures shaping HLA-B allele associations in the Kenyan population. In Rwanda, HLA-B*15:03 and HLA-B*58:02 were frequently co-inherited with multiple other alleles, pointing to their widespread presence and potential central role in the population’s genetic structure. In both Uganda and Zambia, HLA-B*53:01 displayed the highest level of co-inheritance with other alleles, which may suggest a strong ancestral or evolutionary role for this allele within these populations. HLA-B*58:02 exhibited the most prominent inheritance pattern in Rwanda and South Africa, followed by HLA-B*15:03 and HLA-B*42:01 in Rwanda and South Africa, respectively. This consistent pattern across multiple populations may reflect common selective advantages or demographic history influencing HLA allele distribution and inheritance.

## Discussion

This study employed multiple population genetic diversity approaches to investigate Class I HLA diversity in Eastern and Southern African populations compared to U.S. populations. It is important to recognize that the diversity observed in HLA genes cannot be fully accounted for by neutral theory alone, indicating that balancing selection plays a key role in maintaining this polymorphism^49,50^. Although the neutral theory accounts for the baseline levels of genetic variation through mutation and genetic drift, it fall short in explaining the extraordinary allelic diversity and heterozygosity consistently observed in HLA genes loci^51,52^. This weakness highlights the important role of balancing selection in shaping and maintaining HLA diversity over neutral theory as shown in numerous studies^53,54^. Mechanisms such as heterozygote advantage, where individuals with diverse HLA alleles can present a broader repertoire of pathogen-derived peptides, and frequency-dependent selection, which favours rare alleles escaping pathogen recognition, have been implicated in this process. Consequently, the evolutionary relationships inferred in this study should be regarded as tentative^55–57^.

This study observed differences in allele frequencies in all populations. The distribution of allele frequencies is influenced by several factors such as genetic drift, gene flow, mutation, population history, and natural selection, making each population genetically unique^58–60^. Allele frequencies vary across populations and the topmost 5% of frequent alleles reported in this study have also been reported in other studies^23,29,61–63^ at higher or lower frequencies. For example, HLA-A*02:01 is a common allele of the *HLA-A* gene, playing a crucial role in the immune system^64,65^. The high prevalence of HLA-A*02:01 in a population has been associated with certain cancer cells such as some melanoma cells. (https://www.cancer.gov/publications/dictionaries/cancer-terms/def/hla-a0201-antigen)^66–70^. This distinction among the allele frequencies across populations is a testament to the HLA genetic architectural diversity among the populations.

The HD similarity index heatmap demonstrates various degrees of allelic similarities among populations (regions, countries and tribes). The heatmap analysis revealed a marked dissimilarity between the US populations and the African populations across all HLA loci, as evidenced by the consistently low similarity indices. This pronounced divergence underscores the substantial allelic diversity between African populations and those of the United States. The low similarity values also suggest that the US populations possess numerous alleles that are either absent or present at markedly different frequencies in the African populations. For instance, certain protective alleles such as HLA-B*27:05 and B*57:01 are relatively frequent in the EUAM population but are either rare or absent in African populations. Conversely, alleles such as A*74:01 and B*53:01, which are more frequent in African populations, occur at very low frequencies or are absent in European Americans. These patterns reflect the distinct evolutionary histories, demographic pressures, and selective forces that have shaped the HLA landscapes in these populations. Moreover, while the African American population shows slightly higher similarity to African populations than the European Americans, likely due to ancestral ties, the overall dissimilarity still indicates considerable genetic divergence, attributable to admixture with European ancestries, genetic drift, and population bottlenecks experienced during the African diaspora. This allelic distinction has important implications for immune response variation, disease susceptibility, and therapeutic strategies. It highlights the importance of including diverse African populations in immunogenetic studies to ensure equitable and effective medical interventions, particularly for vaccine development and disease association studies. Population comparisons based on haplotype frequencies using MDS showed distinct genetic differences within African populations and between African and US populations. The cartographies and unrooted phylogenetic trees clearly show the distinction between the African and US populations. The European American population show high genetic distances to the African populations at all loci which indicates high diversity between the two populations. The African American population though genetically close to the African population due to their historical background^71^, still maintains a level of distinction which suggests it is not representative of the entire African populations.

The Shannon and Simpson diversity indices further underscore the polymorphic nature of each HLA locus and reveal variations in allele diversity across the populations studied. These indices, which account for a number of distinct alleles richness and their relative frequency distributions (evenness), provide a more nuanced understanding of HLA diversity. Both indices reflected the expected trend of HLA-B being the most polymorphic locus in most populations. However, a notable exception was observed in the South African population. Despite HLA-B exhibiting more detected alleles compared to HLA-A, its diversity indices were comparatively lower in the South African population compared to HLA-A. This unexpected pattern may be attributed to an uneven distribution of alleles at the HLA-B locus, where a few alleles dominate the population leading to reduced overall diversity^23,72^. In contrast, although characterized by fewer total alleles, HLA-A displayed a more even distribution of allele frequencies in the South African population^23,73^. This evenness resulted in a higher Shannon diversity index at this locus, highlighting that allele frequency distribution can significantly influence diversity metrics. This observation reinforces the importance of using indices like Shannon and Simpson, which capture not just allele counts but also their relative abundance, in evaluating true genetic diversity across populations and loci.

The highest values of the HD similarity index in the African populations were observed in the Eastern African region at all loci. This suggests high similarity in terms of their combination of common alleles. Most of the top 5% most frequent alleles at locus A shared between Uganda and Zambia are similar, despite these countries being located in different geographical regions of Africa. This genetic overlap is particularly noteworthy as it highlights shared ancestral or evolutionary patterns that transcend geographical boundaries. The similarity index between Uganda and Zambia at locus A underscores the potential influence of common selective pressures or historical migration events, making this observation both intriguing and significant for understanding genetic diversity between the two countries. Even Kenya, which shares the same geographical region and border with Uganda, exhibits a relatively lower similarity index of 72% compared to the 79% similarity observed between Uganda and Zambia. This disparity highlights that geographic proximity sometimes does not always correlate with genetic similarity, and other factors such as shared ancestry, migration patterns, or historical interactions may play a more significant role in shaping genetic relationships^74–77^. South Africa maintains relatively low similarity to other African countries at all loci except with Zambia at a relatively high percentage similarity. At the tribal level, the Kikuyu tribe also exhibited low similarity to all other tribes within the African region. Generally, most of the African tribes have low (< 70%) similarity between them, even when those tribes are from the same country. While there isn’t a universal quantitative cutoff, thresholds of at least ($\ge$) 70% is usually considered in practical applications and scientific goals such as large-scale vaccine design or HLA-based matching^78–80^. Summarily, the similarity indices observed at the tribal levels also affirm the existence of allelic diversity among tribes of the same countries within African populations. The genetic distances observed in the cartograph suggest allelic diversity among the populations as previously established by other analyses in this study. High diversity was observed in some HLA loci (A:B and A:C) than others (B:C and A:B:C) among African countries. Countries from the same region tended to be in the same location on the cartograph for haplotypes A ~ B and B ~ C. This suggests similar genetic diversities between those countries in the same region. Also, South Africa seemed to have a close genetic distance to Zambia at loci A:B and A:C compared to other countries and a closer genetic distance at loci A:B:C. Interestingly, there was a wide genetic distance between the two countries at loci B:C. This could be linked to some allelic bias towards South Africa compared to Zambia even though both countries are from the same African region. Kenya showed closer genetic distance to Zambia at loci A:B and A:C compared to any other African countries. Similar closeness was also observed at loci B:C between Kenya and Uganda. This suggests low diversity between the two countries at those loci. Furthermore, there was high diversity between Kenya and all other African populations at loci A:B:C. Also, we observed distinct levels of diversity between Rwanda and Uganda at different loci. While high diversity was observed at loci A:C, relatively low diversity was observed between the two countries at loci A:B, B:C and A:B:C. Furthermore, the HD index analysis revealed a high genetic similarity index (75%) at HLA locus C between African Americans and South Africans, which is more than the similarity observed with East African populations such as Uganda (68%), Rwanda (66%), and Kenya (64%) in this study. Although, the African Americans are of predominantly West and Central African ancestry, the result here reveals that there is a significant overlap in HLA-C allele composition between South African and African American populations. Several factors such as less polymorphic nature of HLA-C compared to HLA-A and HLA-B, which result in a slower rate of allele diversification and as a result, may reflect shared deep ancestry^53,54,81^. Also, demographic events, such as genetic drift, admixture and sampling variation, may also influence observed allele frequencies resulting in the high similarity between the two populations. This highlights the importance of including more representative African immunogenetic datasets in African population-specific vaccine design research to better understand trans-population allele sharing and its implications for vaccine designs and disease association studies^58,82,83^.

Additionally, the rarefaction and extrapolation curves support the comparison of allelic diversity between populations (countries and tribes). Although, by observing the shape of the curves, we can infer that allelic variants have been observed within a given number of samples, yet more participants are required to be HLA-typed to observe more unique alleles in all the African populations. South Africa consistently demonstrates the highest observed and extrapolated (number of unique alleles) diversity across all three loci, likely reflecting greater historical population sizes and admixture. Zambia and Uganda also show potential for high diversity upon further sampling. Conversely, Rwanda displays the lowest diversity levels, although extrapolation suggests moderate increases with additional data. Notably, diversity at locus B is more pronounced across populations due to its highly polymorphic nature^84^. These findings underscore the regional heterogeneity of HLA diversity in sub-Saharan Africa and the importance of broad geographic sampling in immunogenetic studies.

Generally, the absence of Bw4 and Bw6 alleles in African populations indicates non-expression of serological markers at the respective locus, which can affect organ transplantation compatibility, immune responses and disease susceptibility within the continent^85,86^. Similarly, the absence of HLA-B*27:05 in Africa is supported by the uncommon presentation of ankylosing spondylitis (AS) disease^87^. HLA-B*27:05 has been reported in literature to be associated with AS^88–90^ and high prevalence of AS disease in African American and European American populations is said to be associated with HLA-B*27:05^91,92^.

Also, the study observed significant deviation from HWE in South Africa at locus C. Similar deviations were observed in the Ngoni population from Zambia at locus B. Potential causes of significant deviation from HWE have been noted in the literature^23,62,63^. Deviations from HWE at these loci in the two populations might indicate inbreeding, which can reduce genetic diversity and the population’s ability to adapt to environmental changes at these loci^93,94^. However, due to the retrospective nature of this study, we acknowledge allelic bias and/or HLA genotyping error as major potential causes of the deviations, as also reported in the literature^95^. The Ewens-Watterson (EW) homozygosity test of neutrality was significant for different populations at different loci. The significant deviations observed for the various populations at different loci suggest balancing selection which helps preserve multiple alleles at each locus and contributes to genetic diversity^96–99^. This is vital for the adaptability and long-term survival of populations, enabling them to cope with changing environments and disease pressure that are associated with alleles in that locus^100^. Notably, the observed significant changes in p-values in HWE and EW homozygosity analyses for the broader populations (Countries) and the subgroup (Tribes) at all loci suggest a shift toward non-significance or otherwise in deviation from HWE and EW homozygosity test for each affected population. For instance, while the overall South African population displays a statistically significant deviation from HWE (p = 0.0155) and EW homozygosity (p = 0.0240) at loci C and A respectively, this deviation appears diminished within the Zulu tribe (p = 0.0533 (HWE), p = 0.0598 (EW)), potentially reflecting a more homogeneous ancestral background compared to the broader South African population. This may reflect population substructure, random mating patterns or demographic history, which tend to promote adherence to HWE^101,102^. The observed changes in p-value may result from reduction in sample sizes, which limits the power to detect significant deviations from HWE and EW homozygosity tests^103–106^. Nevertheless, the proximity of the observed p-value to the threshold still suggests a trend toward deviation, and when considered within the context of the broader South African dataset, the result remains consistent with general HWE and EW homozygosity patterns. These findings underscore the importance of considering both sample size and subpopulation structure in the analyses of HWE and EW homozygosity test for genetically diverse populations.

Although only the Zulu tribal analysis was included in the subgroup analyses for South Africa populations, the genetic background of the Tswana and Xhosa populations in South Africa provides critical insight into the interpretation of HLA diversity within the country. The Tswana people, collectively referred to as Batswana (singular: Motswana), are a Bantu-speaking ethnic group who predominantly reside in Botswana and parts of South Africa^107^. Genetic studies have provided evidence of shared ancestry between the Tswana and other Bantu-speaking groups, tracing their origins to migratory movements from West and Central Africa into Southern Africa^81,108^. Following this migration, the Batswana are believed to have experienced admixture with indigenous Khoisan populations, contributing to the unique genetic makeup being observed^109^. Similarly, the Xhosa people, a major subgroup of the Nguni-speaking Bantu populations, primarily inhabit the Eastern Cape province of South Africa. They exhibit a complex genetic structure shaped by historical admixture between Bantu and Khoisan groups. Genome-wide studies have revealed significant Khoisan maternal contributions to the Xhosa gene pool, reflecting sex-biased gene flow likely resulting from historical social and demographic patterns^110^. Furthermore, Fine-Scale Human population structure analyses among southeastern Bantu-speaking populations, including the Xhosa, suggest that geographic separation and linguistic affiliations have also contributed to their genetic differentiation^111^. These findings underscore the rich and complex demographic history that underpins the genetic landscape of South African populations. Therefore, any interpretation of HLA diversity and immune-related genetic variation within these groups must consider the unique patterns of ancestral contributions and admixture that characterize each population.

Southern African populations, particularly South Africa exhibit notably high Class I HLA genetic diversity, estimated at 34.1% compared to other African populations. This observed diversity suggests ancient human genetic lineages in the region, as noted by Tishkoff et al.^81^ and Prugnolle et al.^53^ that the presence of ancient human populations has allowed for sufficient time for the accumulation of extensive allelic variation at HLA loci through mutation and recombination. Additionally, the high pathogen diversity and infectious disease burden, such as HIV and tuberculosis, in the region, may further suggest factors driving the high HLA diversity, providing a selective advantage by enhancing immune response capabilities^53,54,112^.

The top haplotypes observed between populations affirm the closeness among such populations at the respective locus. The observed asymmetry in LD values indicate non-reciprocal dependencies between HLA loci, suggesting complex recombination histories and the influence of population-specific evolutionary forces, such as balancing selection, genetic drift, or historical bottlenecks^53,113^. The results provide strong evidence that certain alleles of HLA-B and HLA-C are tightly associated than allele pairs of other loci. These asymmetric patterns may reflect differing rates or directions of recombination and selective pressure acting on specific allele combinations within each population^49^. Collectively, these results underscore regional differences in HLA haplotype architecture, highlighting the diversity and structure of genetic linkages that may not be captured by global reference datasets. This has important implications for HLA-based disease association studies, transplant compatibility assessments, and vaccine design, where reliance on generalized or non-representative HLA data may lead to inaccurate inferences or suboptimal outcomes^23^. The findings emphasize the critical need for population-specific HLA datasets to inform precise immunogenetic research and tailor interventions to local genetic landscapes.

The discrepancies in the unique alleles observed in the population groups might be due to the sample size of the populations in this study. Hence, larger sample sizes with more African countries need to be studied to get a comprehensive picture of the HLA genetic diversity across Africa.

While this study investigated classical Class I *HLA* genes and the patterns of alleles inheritance at each locus, further fine-tuning and refinement are needed to give a more holistic picture across all populations. Furthermore, additional focus on non-classical and Class II genes is warranted to assess the genetic profiles relative to disease susceptibility and protection in each population. These studies will assist in understanding genetic diversity and inform in *HLA* population-based therapeutic development.

In this study, we have established HLA diversity in the Eastern (Kenya, Rwanda and Uganda) and Southern (South Africa and Zambia) African region of the African continent. Comparison of the HLA data at both country and tribal levels suggests genetic differences within the African populations and uniqueness of the Eastern and Southern African populations relative to the US-based (African American) population. These analyses demonstrate the limitations of applying HLA data from one region to another, reinforcing the necessity of collecting high-quality HLA data from all regions of Africa and its varied ethnicities. Comprehensive data collection is crucial for enhancing vaccine design and advancing our understanding of HLA disease associations, ultimately improving healthcare outcomes across the continent. Finally, due to genetic admixture, cautions must be made against extrapolating HLA data from other continents to inform African vaccine development.

## Materials and methods

### Ethics statement

The study was approved by the Biomedical Research and Ethics Committee (BREC), UKZN as well as Massachusetts General Hospital (MGH) Ethics Review Board. Participation in the HLA study is voluntary, and participants provided both verbal and signed informed consent.

### Population and sample

In conjunction with our collaborators, the Class I HLA data used in this study were five distinct cohorts within African populations and two ethnic groups in the United States, all of which are part of HIV research cohorts. This study attempts to understand the HLA diversity in Africa population in general and the need for more HLA data for T-cell vaccine design for African populations. Most readily available HLA data to us are derive from People living with HIV (PWLH), reflecting the intense research focus on HIV disease dynamics and cure strategies. Future studies will interrogate other infectious diseases as Africa-specific HLA data becomes available. The African cohorts are comprised of the Centre for The Aids Programme of Research In South Africa (CAPRISA), International AIDS Vaccine Initiative (IAVI), Female Rising through Education, Support and Health (FRESH), and Sinikithemba in South Africa. The ethnic groups from the US are the African Americans (AFAM) and European Americans (EUAM)^114^. Necessary approvals were granted for all the HLA studies across the different cohorts. The present study includes 2,718 anonymous samples from unrelated subjects across the various cohorts. African samples were obtained from three eastern and two southern African countries and are distributed as follows: Kenya $\left(n=109\right)$, Rwanda $\left(n=173\right)$, Uganda $\left(n=231\right)$, South Africa – RSA $\left(n=1640\right)$ and Zambia $\left(n=565\right)$. Of the five countries sampled within the African sub-region, tribal information of major tribes was obtained from four countries excluding Rwanda due to historical development. Meanwhile, other tribal groups (such as Tswana and Xhosa from South Africa) with very few participants were excluded from the subgroup analysis due to insufficient sample sizes, which could compromise the statistical validity of the analyses. The tribal groups sampled within the four countries are Bemba (Zambia, n = 142), Chewa (Zambia, n = 63), Kikuyu (Kenya, n = 25), Lozi (Zambia, n = 23), Luhya (Kenya, n = 21), Muganda (Uganda, n = 134), Munyankole (Uganda, n = 25), Munyarwanda (Uganda, n = 26), Ngoni (Zambia, n = 44), Nsenga (Zambia, n = 70), Tonga (Zambia, n = 29), Tumbuka (Zambia, n = 29) and Zulu (South Africa, n = 1624). Similarly, US ethnic groups were distributed as EUAM (n = 1765) or AFAM (n = 661). This is according to the data from the author of the publication^115^where the data was presented. In accordance with the World Medical Association Declaration of Helsinki^116^, participants’ personal identifiers were not accessed to maintain confidentiality.

### HLA

HLA typing was performed using a targeted next-generation sequencing method. Briefly, locus-specific primers were used to amplify all exons of *HLA-A*, *HLA-B*, and *HLA-C* genes with the Fluidigm Access Array (Fluidigm, Singapore PTW Ltd, Singapore). The Fluidigm PCR amplicons were pooled and subjected to sequencing either on the Roche 454 platform (Roche, Indianapolis, IN, USA) or the Illumina MiSeq platform (Illumina, San Diego, CA, USA). HLA alleles and genotypes were called using the Omixon HLA Explore (beta version) software (Omixon, Budapest, Hungary)^117–119^.

### Data cleaning and validation

All the HLA data used in this study was thoroughly examined for inconsistencies and ambiguities (e.g. missing alleles, duplications etc.), which were resolved using an in silico approach as previously described^120^. Few participants were observed to have similar information such as the same patient identifier (PID). Participants with more allelic information were retained for the study while participants with partially or entirely missing allelic information were excluded from the analysis. Furthermore, all the HLA data were analysed at 2-field (4-digit) resolution in this study.

All the HLA data were verified for allele validity, and all allele nomenclature reported before 2010 were updated using current nomenclature conversion tables and conversion tools provided by IMGT/HLA database (IMGT/HLA Database, IPD-IMGT/HLA 3.56, release of January 2024, https://www.ebi.ac.uk/ipd/imgt/hla/alleles/). Similarly, haplotype nomenclature was done in accordance with the genotype list string report^121^ aimed at organizing and discriminating phased genes, genotypes, and ambiguous assignments.

### Statistical analysis

The primary objective of this analysis was to characterize HLA diversity within five African countries and compare these diversities among the five countries and with populations from the United States. Additionally, the analysis sought to explore HLA diversity across various African tribes. However, due to the limited sample sizes available for some tribes, these tribal-level analyses are considered exploratory, and the findings should be validated in future studies when more sample sizes become available. Allele frequencies were estimated by direct counting using Python for population genomics (PyPop) version 1.0.0^122^. The haplotypes and haplotype frequencies (HF) were estimated by resolving phase and allelic ambiguities using the expectation–maximization (EM) steps with progressive insertion algorithm by setting the posterior probability to 0.0001 in the haplo.stats version 1.9.5.1 R package^123^. The HLA data were converted to Arlequin version 3.5.2 software^124^ input files using CREATE software version 1.37^125^ to examine deviations from HWE, adopting a modification of the Markov random walk algorithm with 100 000 dememorization steps^105^. Estimation of non-random and directional association between alleles was determined via ALD^46,126,127^. The Ewens-Watterson homozygosity test of neutrality was implemented in PyPop using the Slatkin principle of implementation^128,129^. Multiple comparisons of homozygosity test of neutrality was addressed via Benjamini & Hochberg correction method^130^. Aplha diversity indices (species richness—number of alleles^131,132^, Shannon index–entropy^133^, and Gini-Simpson index—probability that two alleles taken from the sample at random are of different types^134,135^) were employed to measure within population diversities. The Hellinger distance, a measure of beta diversity based on relative frequencies^136,137^, was employed to determine diversity between populations. Furthermore, a rarefaction and extrapolation analysis with bootstrap sampling size of 50 and 95% confidence interval was used to gain quantitative insights into the number of alleles that were observed in each population as a function of the number of participants^138^. Similarly, as a measure of genetic distance between populations, relative haplotype frequency data from each country were dimensionality reduced using classical multidimensional scaling (MDS) to create a 2-dimensional genetic cartography. A threshold of relative HF $\ge 1\%$ was used to perform the MDS analysis^31,139^. Based on the analysis, two countries are close to each other on the map if the distribution of the HLA alleles in these two countries are close to each other, relative to the distribution observed in the other countries.

## Supplementary Information

Supplementary Information.

## Data Availability

The datasets used and/or analysed during the current study are available from the corresponding author on reasonable request. All materials supporting the analysis during this study are included in this published article and its supplementary information files.

## Abbreviations

RSA: Republic of South Africa
HF: Haplotype frequency
LD: Linkage disequilibrium
HWE: Hardy–Weinberg equilibrium
AFAM: African American
EUAM: European American
HD: Hellinger distance
ALD: Asymmetric linkage disequilibrium
EW: Ewens-Watterson
