# Supplementary material for: High resolution class I HLA-A, -B, and -C diversity in Eastern and Southern African populations
Source: Sci Rep. 2025 Jul 2;15:23667. doi: 10.1038/s41598-025-06704-4 (PMC12222908; doi:10.1038/s41598-025-06704-4)
Supplement: Supplementary file 1 — Supplementary Information. [file 41598_2025_6704_MOESM1_ESM.docx]

**Supplementary Table 1|** Gene frequencies of HLA ~A alleles according to population of participants

| **HLA ~A** | **Kenya**  $(n = 109)$ | **Rwanda**  $(n = 173)$ | **South Africa**  $(n = 1640)$ | **Uganda**  $(n = 231)$ | **Zambia**  $(n = 565)$ | **EUAM**  $(n = 1765)$ | **AFAM**  $(n = 661)$ |
| --- | --- | --- | --- | --- | --- | --- | --- |
| A*01:01 | 0.0642 | 0.1156 | 0.0317 | 0.0714 | 0.0159 | 0.1371 | 0.0409 |
| A*01:02 |  | 0.0029 | 0.0003 | 0.0108 | 0.0009 | 0.0006 | 0.0038 |
| A*01:03 | 0.0092 | 0.0087 |  | 0.0043 | 0.0027 | 0.0006 | 0.0008 |
| A*01:09 |  | 0.0029 |  |  |  |  | 0.0015 |
| A*01:22N |  |  |  |  |  |  | 0.0008 |
| A*01:23 |  |  | 0.0024 |  |  |  |  |
| A*02:01 | 0.1239 | 0.1387 | 0.0512 | 0.1364 | 0.0912 | 0.2535 | 0.1082 |
| A*02:02 | 0.0459 | 0.0809 | 0.0137 | 0.0455 | 0.0381 | 0.0017 | 0.0363 |
| A*02:04 |  |  |  | 0.0022 | 0.0009 | 0.0017 | 0.0023 |
| A*02:05 | 0.0459 | 0.0145 | 0.0515 | 0.0043 | 0.0186 | 0.0096 | 0.0174 |
| A*02:06 |  |  |  |  |  | 0.0054 | 0.0038 |
| A*02:09 |  |  |  |  |  | 0.0006 |  |
| A*02:11 |  |  | 0.0006 |  |  | 0.0023 | 0.0008 |
| A*02:14 | 0.0046 | 0.0058 | 0.0034 | 0.0065 | 0.0018 |  |  |
| A*02:16 |  |  |  |  |  |  | 0.0008 |
| A*02:17 |  |  |  |  |  | 0.0011 |  |
| A*02:20 |  |  |  |  |  | 0.0003 |  |
| A*02:22 |  |  |  |  |  | 0.0003 |  |
| A*02:274 |  |  |  |  |  | 0.0003 |  |
| A*02:30 |  |  |  |  |  | 0.0003 |  |
| A*02:724 |  |  |  |  |  | 0.0003 |  |
| A*03new |  |  |  |  | 0.0009 |  |  |
| A*03:01 | 0.0229 | 0.0636 | 0.0601 | 0.0563 | 0.0460 | 0.1241 | 0.0946 |
| A*03:02 |  |  | 0.0003 |  |  | 0.0026 | 0.0008 |
| A*03:05 |  |  |  |  |  | 0.0003 |  |
| A*03:21 |  |  | 0.0003 |  |  |  |  |
| A*03:21N |  |  |  |  |  | 0.0003 |  |
| A*03:49 |  |  |  |  |  | 0.0003 |  |
| A*11:01 | 0.0092 |  | 0.0012 |  |  | 0.0640 | 0.0159 |
| A*11:02 |  |  |  |  |  |  | 0.0008 |
| A*11:04 |  |  |  |  |  | 0.0006 |  |
| A*11:67 |  |  |  |  |  | 0.0003 |  |
| A*23new |  |  | 0.0003 |  |  |  |  |
| A*23:01 | 0.0321 | 0.0838 | 0.1037 | 0.0714 | 0.1142 | 0.0181 | 0.0787 |
| A*23:02 | 0.0046 |  |  |  | 0.0018 |  |  |
| A*24:02 | 0.0138 | 0.0145 | 0.0268 |  | 0.0106 | 0.0807 | 0.0189 |
| A*24:03 |  |  | 0.0003 |  |  | 0.0037 | 0.0023 |
| A*24:05 |  |  |  |  |  | 0.0003 |  |
| A*24:07 |  |  | 0.0003 |  |  |  | 0.0008 |
| A*24:23 |  |  |  |  |  | 0.0003 |  |
| A*24:31 |  |  |  |  | 0.0009 |  |  |
| A*24:314 |  |  |  |  |  | 0.0003 |  |
| A*24:95 |  |  |  |  |  | 0.0003 |  |
| A*25:01 |  |  |  |  |  | 0.0292 | 0.0061 |
| A*26:01 | 0.0229 | 0.0058 | 0.0198 | 0.0173 | 0.0062 | 0.0298 | 0.0121 |
| A*26:03 |  | 0.0029 |  |  |  |  |  |
| A*26:08 |  |  |  |  |  | 0.0017 |  |
| A*26:12 |  | 0.0087 | 0.0018 |  | 0.0009 |  |  |
| A*26:121 |  |  | 0.0006 |  |  |  |  |
| A*29:01 |  |  | 0.0049 | 0.0022 | 0.0027 | 0.0037 | 0.0008 |
| A*29:02 | 0.0505 | 0.0318 | 0.0689 | 0.0390 | 0.0628 | 0.0365 | 0.0318 |
| A*29:10 |  |  |  |  |  | 0.0006 |  |
| A*29:11 |  |  | 0.0140 |  |  |  |  |
| A*30:01 | 0.0688 | 0.0462 | 0.0997 | 0.0758 | 0.0920 | 0.0193 | 0.0522 |
| A*30:02 | 0.0826 | 0.0751 | 0.0726 | 0.0758 | 0.1310 | 0.0105 | 0.0855 |
| A*30:03 |  |  | 0.0003 |  |  |  | 0.0008 |
| A*30:04 | 0.0275 | 0.0087 | 0.0213 | 0.0087 | 0.0080 | 0.0034 | 0.0023 |
| A*30:09 |  | 0.0029 | 0.0046 | 0.0087 | 0.0080 |  | 0.0008 |
| A*30:10 |  |  |  |  |  | 0.0006 | 0.0015 |
| A*30:151 |  |  |  |  |  |  | 0.0008 |
| A*31:01 |  | 0.0029 | 0.0012 | 0.0022 | 0.0018 | 0.0357 | 0.0197 |
| A*31:02 |  |  |  |  |  | 0.0003 |  |
| A*31:03 |  |  |  | 0.0022 |  |  |  |
| A*31:04 | 0.0046 |  |  | 0.0043 | 0.0009 |  |  |
| A*32:01 |  | 0.0029 | 0.0043 | 0.0152 | 0.0035 | 0.0462 | 0.0166 |
| A*32:106 |  |  | 0.0018 |  |  |  |  |
| A*32:114 |  |  |  |  |  | 0.0003 |  |
| A*33:01 | 0.0138 | 0.0029 | 0.0073 | 0.0173 | 0.0124 | 0.0094 | 0.0174 |
| A*33:03 | 0.0046 | 0.0087 | 0.0149 | 0.0130 | 0.0124 | 0.0051 | 0.0590 |
| A*33:05 |  |  |  |  |  | 0.0003 | 0.0008 |
| A*34:01 |  |  | 0.0003 |  |  |  |  |
| A*34:02 | 0.0183 | 0.0202 | 0.0439 | 0.0281 | 0.0425 | 0.0023 | 0.0363 |
| A*36:01 | 0.0275 | 0.0318 | 0.0043 | 0.0433 | 0.0460 | 0.0014 | 0.0212 |
| A*43:01 |  |  | 0.0271 |  | 0.0018 |  |  |
| A*66:01 | 0.0229 | 0.0260 | 0.0399 | 0.0346 | 0.0327 | 0.0040 | 0.0212 |
| A*66:02 |  |  | 0.0104 | 0.0043 | 0.0027 |  | 0.0083 |
| A*66:03 |  |  | 0.0009 |  | 0.0018 |  | 0.0015 |
| A*68:01 | 0.0505 | 0.0087 | 0.0284 | 0.0087 | 0.0124 | 0.0278 | 0.0287 |
| A*68:02 | 0.1514 | 0.0838 | 0.0875 | 0.0974 | 0.1000 | 0.0145 | 0.0598 |
| A*68:03 |  |  |  |  |  | 0.0009 |  |
| A*68:04 |  |  | 0.0003 |  |  |  |  |
| A*68:07 |  |  |  |  |  | 0.0006 |  |
| A*68:10 |  |  |  |  |  |  | 0.0008 |
| A*68:15 |  |  |  |  |  | 0.0003 |  |
| A*68:27 |  |  | 0.0024 |  |  |  |  |
| A*69:01 |  |  |  |  |  | 0.0028 | 0.0008 |
| A*69:02 |  |  | 0.0003 |  |  |  |  |
| A*74:00 |  |  | 0.0226 |  |  | 0.0006 | 0.0151 |
| A*74:01 | 0.0780 | 0.0896 | 0.0366 | 0.0844 | 0.0690 | 0.0006 | 0.0575 |
| A*74:02 |  | 0.0029 |  |  |  |  |  |
| A*74:03 |  |  |  | 0.0022 |  | 0.0003 | 0.0038 |
| A*74:05 |  | 0.0029 |  |  |  |  |  |
| A*74:11 |  |  |  |  |  |  | 0.0008 |
| A*80:01 |  | 0.0029 | 0.0088 | 0.0065 | 0.0044 | 0.0003 | 0.0068 |

**Supplementary Table 2|** Gene frequencies of HLA ~B alleles according to population of participants

| **HLA ~B** | **Kenya**  $(n = 109)$ | **Rwanda**  $(n = 173)$ | **South Africa**  $(n = 1640)$ | **Uganda**  $(n = 231)$ | **Zambia**  $(n = 565)$ | **EUAM**  $(n = 1765)$ | **AFAM**  $(n = 661)$ |
| --- | --- | --- | --- | --- | --- | --- | --- |
| B*07:02 | 0.0734 | 0.0549 | 0.0561 | 0.0563 | 0.0336 | 0.0952 | 0.0552 |
| B*07:04 |  |  | 0.0003 |  |  | 0.0003 |  |
| B*07:05 |  | 0.0145 | 0.0070 | 0.0043 | 0.0018 | 0.0048 | 0.0091 |
| B*07:08 |  |  |  |  |  | 0.0003 |  |
| B*07:09 |  |  |  |  |  |  | 0.0008 |
| B*07:51 |  |  |  |  | 0.0009 |  |  |
| B*08:01 | 0.0046 | 0.0173 | 0.0616 | 0.0238 | 0.0301 | 0.0756 | 0.0371 |
| B*08:02 |  |  |  |  |  | 0.0003 |  |
| B*13:01 |  |  | 0.0003 |  |  |  |  |
| B*13:02 | 0.0138 | 0.0145 | 0.0162 | 0.0108 | 0.0106 | 0.0283 | 0.0129 |
| B*13:03 |  |  | 0.0012 |  | 0.0009 |  |  |
| B*14:01 | 0.0046 |  | 0.0229 | 0.0022 | 0.0283 | 0.0122 | 0.0091 |
| B*14:02 | 0.0321 | 0.0318 | 0.0125 | 0.0346 | 0.0327 | 0.0456 | 0.0272 |
| B*14:03 |  | 0.0029 |  | 0.0022 | 0.0027 |  | 0.0023 |
| B*14:06 |  |  | 0.0003 |  |  | 0.0003 |  |
| B*15:01 |  |  | 0.0021 |  | 0.0009 | 0.0487 | 0.0121 |
| B*15:02 |  |  |  |  |  | 0.0006 |  |
| B*15:03 | 0.0826 | 0.1098 | 0.0832 | 0.0823 | 0.1035 | 0.0028 | 0.0492 |
| B*15:07 |  |  |  |  |  | 0.0006 | 0.0008 |
| B*15:08 |  |  | 0.0003 |  |  |  |  |
| B*15:10 | 0.0734 | 0.0347 | 0.0887 | 0.0519 | 0.0602 | 0.0011 | 0.0250 |
| B*15:11 |  |  |  |  |  | 0.0003 |  |
| B*15:13 |  |  | 0.0006 |  |  |  |  |
| B*15:16 | 0.0138 | 0.0145 | 0.0131 | 0.0108 | 0.0071 | 0.0009 | 0.0174 |
| B*15:17 | 0.0092 | 0.0087 | 0.0009 | 0.0130 | 0.0062 | 0.0065 | 0.0038 |
| B*15:18 |  |  | 0.0009 |  |  | 0.0011 | 0.0015 |
| B*15:22 |  |  | 0.0012 |  |  |  |  |
| B*15:220 |  |  | 0.0009 |  | 0.0035 | 0.0006 | 0.0008 |
| B*15:24 |  |  | 0.0003 |  |  | 0.0009 |  |
| B*15:31 | 0.0092 |  |  | 0.0043 | 0.0009 | 0.0006 | 0.0008 |
| B*15:37 |  |  |  | 0.0022 |  |  |  |
| B*15:40 |  |  |  |  |  | 0.0003 |  |
| B*15:47 |  |  |  |  | 0.0027 |  |  |
| B*15:83 |  |  |  |  | 0.0018 |  |  |
| B*18:01 | 0.0183 | 0.0289 | 0.0354 | 0.0346 | 0.0319 | 0.0428 | 0.0242 |
| B*18:03 | 0.0046 | 0.0029 |  | 0.0043 | 0.0009 |  |  |
| B*18:05 |  |  |  |  |  | 0.0003 |  |
| B*18:20 |  |  |  |  |  | 0.0003 |  |
| B*27:02 |  |  |  |  |  | 0.0068 | 0.0008 |
| B*27:03 | 0.0092 | 0.0087 |  | 0.0043 |  | 0.0003 | 0.0045 |
| B*27:05 |  |  | 0.0015 |  |  | 0.0501 | 0.0144 |
| B*27:06 |  |  |  |  |  |  | 0.0008 |
| B*27:07 |  |  |  |  |  | 0.0009 |  |
| B*27:09 |  |  |  |  |  | 0.0003 |  |
| B*27:12 |  |  |  |  |  | 0.0003 | 0.0008 |
| B*27:13 |  |  |  |  |  | 0.0003 |  |
| B*35:01 | 0.0321 | 0.0318 | 0.0177 | 0.0238 | 0.0354 | 0.0456 | 0.0386 |
| B*35:02 |  | 0.0029 | 0.0015 | 0.0022 | 0.0009 | 0.0079 | 0.0015 |
| B*35:03 |  |  | 0.0003 |  |  | 0.0162 | 0.0023 |
| B*35:04 |  |  |  |  |  |  | 0.0008 |
| B*35:05 |  |  |  |  |  | 0.0003 | 0.0008 |
| B*35:08 |  |  |  |  |  | 0.0037 | 0.0008 |
| B*35:12 |  |  |  |  |  | 0.0006 |  |
| B*35:14 |  |  |  |  |  | 0.0003 |  |
| B*35:17 |  |  |  |  |  | 0.0003 |  |
| B*35:187 |  |  |  |  |  | 0.0003 |  |
| B*35:32 |  |  |  |  |  | 0.0003 |  |
| B*35:41 |  |  |  |  |  | 0.0003 |  |
| B*35:42 |  |  |  |  |  | 0.0003 |  |
| B*35:43 |  |  |  |  |  | 0.0023 | 0.0008 |
| B*37:01 | 0.0046 | 0.0116 |  | 0.0022 | 0.0009 | 0.0119 | 0.0038 |
| B*38:01 |  |  |  |  |  | 0.0221 | 0.0023 |
| B*38:02 |  |  | 0.0003 |  |  |  |  |
| B*39:01 |  |  |  |  | 0.0009 | 0.0105 | 0.0045 |
| B*39:02 |  |  |  |  |  | 0.0003 |  |
| B*39:05 |  |  |  |  |  | 0.0006 | 0.0008 |
| B*39:06 |  |  |  |  |  | 0.0043 | 0.0008 |
| B*39:08 |  |  |  |  |  | 0.0009 |  |
| B*39:10 | 0.0046 | 0.0145 | 0.0210 | 0.0130 | 0.0124 | 0.0011 | 0.0250 |
| B*39:11 |  |  |  |  |  | 0.0006 |  |
| B*39:12 |  |  |  |  |  | 0.0003 |  |
| B*39:20 |  |  |  |  |  |  | 0.0008 |
| B*39:24 |  |  |  |  |  | 0.0009 | 0.0008 |
| B*40:01 |  |  | 0.0009 |  |  | 0.0385 | 0.0129 |
| B*40:02 |  |  |  |  |  | 0.0190 | 0.0061 |
| B*40:04 |  |  |  |  |  | 0.0006 |  |
| B*40:06 |  |  | 0.0009 |  |  | 0.0003 | 0.0023 |
| B*40:08 |  |  |  |  |  | 0.0006 |  |
| B*40:12 | 0.0046 | 0.0029 |  | 0.0065 |  |  | 0.0008 |
| B*40:16 | 0.0092 | 0.0058 |  | 0.0022 | 0.0062 | 0.0003 | 0.0008 |
| B*40:31 |  |  |  |  |  | 0.0003 |  |
| B*41:01 | 0.0138 | 0.0116 | 0.0122 | 0.0087 | 0.0044 | 0.0062 | 0.0030 |
| B*41:02 | 0.0092 |  | 0.0012 |  | 0.0009 | 0.0037 | 0.0053 |
| B*41:03 |  |  |  |  |  |  | 0.0008 |
| B*42:01 | 0.0734 | 0.0405 | 0.1079 | 0.0433 | 0.0912 | 0.0011 | 0.0378 |
| B*42:02 | 0.0046 |  | 0.0162 | 0.0043 | 0.0097 | 0.0003 | 0.0045 |
| B*44:02 |  |  | 0.0003 |  |  | 0.0754 | 0.0121 |
| B*44:03 | 0.0275 | 0.0347 | 0.0918 | 0.0216 | 0.0646 | 0.0473 | 0.0484 |
| B*44:04 |  |  |  |  |  | 0.0009 |  |
| B*44:05 |  |  |  |  |  | 0.0020 | 0.0008 |
| B*44:10 |  |  |  |  |  |  | 0.0015 |
| B*44:15 | 0.0092 | 0.0376 |  | 0.0390 |  |  | 0.0015 |
| B*44:27 |  |  |  |  |  | 0.0003 |  |
| B*45:00 |  |  | 0.0006 |  |  |  |  |
| B*45:01 | 0.1009 | 0.0491 | 0.0299 | 0.0628 | 0.0735 | 0.0077 | 0.0303 |
| B*45:07 |  |  | 0.0046 |  | 0.0009 |  |  |
| B*46:01 |  |  |  |  |  | 0.0003 |  |
| B*47:01 |  | 0.0087 | 0.0006 | 0.0065 |  | 0.0011 | 0.0015 |
| B*47:03 | 0.0046 |  |  | 0.0022 |  |  |  |
| B*48:01 |  |  |  |  |  | 0.0023 | 0.0008 |
| B*48:02 |  |  |  |  |  | 0.0006 |  |
| B*49:01 | 0.0459 | 0.0838 | 0.0073 | 0.0649 | 0.0115 | 0.0145 | 0.0265 |
| B*50:01 | 0.0046 |  | 0.0003 |  | 0.0053 | 0.0065 | 0.0053 |
| B*50:02 |  |  |  |  |  | 0.0006 |  |
| B*51:01 | 0.0092 | 0.0173 | 0.0085 | 0.0152 | 0.0204 | 0.0527 | 0.0250 |
| B*51:02 |  |  |  |  |  |  | 0.0008 |
| B*51:05 |  |  |  |  |  | 0.0003 |  |
| B*51:07 |  |  |  |  |  | 0.0009 | 0.0008 |
| B*51:08 |  |  |  |  |  | 0.0003 | 0.0008 |
| B*51:09 |  |  |  |  |  | 0.0011 |  |
| B*51:22 |  |  |  |  |  | 0.0003 |  |
| B*51:64 |  |  |  |  |  |  | 0.0008 |
| B*52:01 | 0.0046 |  | 0.0003 |  |  | 0.0193 | 0.0197 |
| B*52:02 |  |  |  |  |  | 0.0003 |  |
| B*53:01 | 0.0872 | 0.0636 | 0.0183 | 0.0974 | 0.0973 | 0.0096 | 0.0930 |
| B*54:01 |  |  |  |  |  | 0.0003 |  |
| B*55:01 |  |  | 0.0006 |  |  | 0.0119 | 0.0015 |
| B*56:01 |  | 0.0029 |  |  |  | 0.0051 | 0.0015 |
| B*57:01 |  |  | 0.0003 | 0.0022 | 0.0018 | 0.0793 | 0.0106 |
| B*57:02 | 0.0092 |  | 0.0079 | 0.0130 | 0.0097 | 0.0014 | 0.0129 |
| B*57:03 | 0.0413 | 0.0347 | 0.0229 | 0.0390 | 0.0531 | 0.0105 | 0.1082 |
| B*58:01 | 0.0688 | 0.0607 | 0.0503 | 0.0584 | 0.0549 | 0.0133 | 0.0416 |
| B*58:02 | 0.0550 | 0.1127 | 0.1143 | 0.0801 | 0.0531 | 0.0006 | 0.0235 |
| B*58:10 |  |  | 0.0003 |  |  |  |  |
| B*58:11 |  |  |  |  |  |  | 0.0015 |
| B*58:15 |  |  |  |  | 0.0009 |  |  |
| B*67:01 |  |  |  |  |  | 0.0003 |  |
| B*73:01 | 0.0092 | 0.0029 |  | 0.0022 | 0.0027 | 0.0006 | 0.0008 |
| B*78:01 |  |  |  |  |  |  | 0.0061 |
| B*81:00 |  |  | 0.0058 |  |  |  |  |
| B*81:01 | 0.0183 | 0.0260 | 0.0418 | 0.0433 | 0.0257 | 0.0006 | 0.0522 |
| B*81:03 |  |  | 0.0006 |  |  |  |  |
| B*82:01 |  |  |  |  | 0.0009 |  | 0.0015 |
| B*82:02 |  |  | 0.0049 | 0.0043 |  |  | 0.0008 |

**Supplementary Table 3|** Gene frequencies of HLA ~C alleles according to population of participants

| **HLA ~C** | **Kenya**  $(n = 109)$ | **Rwanda**  $(n = 173)$ | **South Africa**  $(n = 1640)$ | **Uganda**  $(n = 231)$ | **Zambia**  $(n = 565)$ | **EUAM**  $(n = 1765)$ | **AFAM**  $(n = 661)$ |
| --- | --- | --- | --- | --- | --- | --- | --- |
| C*01:02 |  | 0.0029 | 0.0012 |  | 0.0009 | 0.0476 | 0.0098 |
| C*01:127 |  |  |  |  |  | 0.0006 |  |
| C*02:02 | 0.0092 | 0.0087 | 0.0134 | 0.0087 | 0.0080 | 0.0527 | 0.0242 |
| C*02:05 |  |  | 0.0003 |  |  |  |  |
| C*02:10 | 0.0642 | 0.1012 | 0.0851 | 0.0758 | 0.1071 | 0.0026 | 0.0507 |
| C*02:17 |  |  | 0.0067 |  |  |  |  |
| C*02:29 |  |  |  |  |  | 0.0003 |  |
| C*03:02 | 0.0183 | 0.0116 | 0.0174 | 0.0303 | 0.0133 | 0.0034 | 0.0212 |
| C*03:03 |  |  | 0.0052 |  | 0.0080 | 0.0388 | 0.0083 |
| C*03:04 | 0.0688 | 0.0405 | 0.0488 | 0.0584 | 0.0637 | 0.0606 | 0.0439 |
| C*03:05 |  |  |  |  |  | 0.0009 | 0.0008 |
| C*03:06 |  |  |  |  |  | 0.0003 |  |
| C*03:09 |  |  |  |  |  | 0.0003 |  |
| C*04:01 | 0.1193 | 0.1243 | 0.1049 | 0.1645 | 0.1540 | 0.0977 | 0.1498 |
| C*04:03 |  |  | 0.0006 |  |  |  |  |
| C*04:04 |  |  | 0.0003 |  |  |  |  |
| C*04:07 | 0.0138 | 0.0347 |  | 0.0390 | 0.0035 | 0.0006 | 0.0030 |
| C*04:08 |  |  |  |  |  | 0.0003 |  |
| C*04:13 |  |  |  |  |  |  | 0.0015 |
| C*04:226 |  |  | 0.0003 |  |  |  |  |
| C*04:27 |  |  |  |  |  |  | 0.0008 |
| C*04:29 |  |  |  |  |  |  | 0.0008 |
| C*05:01 | 0.0046 | 0.0029 | 0.0049 | 0.0022 | 0.0088 | 0.0754 | 0.0227 |
| C*06:02 | 0.1468 | 0.2283 | 0.1530 | 0.1537 | 0.1265 | 0.1286 | 0.0741 |
| C*06:03 |  |  |  |  | 0.0009 |  |  |
| C*06:08 |  |  |  |  |  |  | 0.0015 |
| C*07:01 | 0.1284 | 0.1561 | 0.1253 | 0.1234 | 0.0965 | 0.1300 | 0.1475 |
| C*07:02 | 0.0734 | 0.0607 | 0.0726 | 0.0606 | 0.0310 | 0.1077 | 0.0537 |
| C*07:04 | 0.0229 | 0.0116 | 0.0171 | 0.0346 | 0.0168 | 0.0091 | 0.0068 |
| C*07:05 |  |  |  |  |  |  | 0.0015 |
| C*07:06 |  |  | 0.0052 |  | 0.0044 | 0.0003 | 0.0015 |
| C*07:18 |  |  | 0.0006 |  |  |  |  |
| C*07:621 |  |  |  |  |  |  | 0.0008 |
| C*08:01 |  |  | 0.0006 |  |  | 0.0026 | 0.0023 |
| C*08:02 | 0.0642 | 0.0434 | 0.0198 | 0.0714 | 0.0690 | 0.0569 | 0.0461 |
| C*08:03 |  |  |  |  |  | 0.0006 |  |
| C*08:04 |  |  | 0.0402 | 0.0022 | 0.0035 | 0.0003 | 0.0287 |
| C*08:13 |  |  |  |  |  |  | 0.0008 |
| C*08:43 |  |  |  |  |  |  | 0.0008 |
| C*12:02 | 0.0046 |  | 0.0003 |  |  | 0.0170 | 0.0030 |
| C*12:03 | 0.0046 | 0.0116 | 0.0183 | 0.0130 | 0.0133 | 0.0589 | 0.0356 |
| C*14:02 | 0.0138 | 0.0145 | 0.0052 | 0.0108 | 0.0097 | 0.0170 | 0.0197 |
| C*14:03 | 0.0046 | 0.0029 | 0.0006 | 0.0022 | 0.0035 |  | 0.0053 |
| C*15:02 | 0.0138 | 0.0145 | 0.0003 | 0.0087 | 0.0018 | 0.0266 | 0.0114 |
| C*15:05 |  |  | 0.0055 |  | 0.0027 | 0.0060 | 0.0144 |
| C*15:06 |  |  |  |  |  | 0.0003 |  |
| C*15:13 |  |  |  |  |  | 0.0006 |  |
| C*15:25 |  |  | 0.0006 |  | 0.0018 |  |  |
| C*16:01 | 0.0780 | 0.0260 | 0.0585 | 0.0519 | 0.0690 | 0.0374 | 0.0696 |
| C*16:02 | 0.0046 | 0.0058 |  | 0.0022 |  | 0.0034 | 0.0015 |
| C*16:04 | 0.0046 |  | 0.0003 |  |  | 0.0014 |  |
| C*17 new |  |  |  |  | 0.0009 |  |  |
| C*17:00 |  |  | 0.0552 |  |  | 0.0034 | 0.0129 |
| C*17:01 | 0.1009 | 0.0549 | 0.0829 | 0.0584 | 0.1097 | 0.0040 | 0.0340 |
| C*17:03 |  |  |  |  |  | 0.0031 | 0.0015 |
| C*18:00 |  |  | 0.0363 |  |  | 0.0023 | 0.0545 |
| C*18:01 | 0.0367 | 0.0434 | 0.0070 | 0.0281 | 0.0487 | 0.0003 | 0.0083 |
| C*18:02 |  |  | 0.0055 |  | 0.0230 | 0.0006 | 0.0250 |

**Supplementary Table 4|** full list of haplotype A~B for all populations

| **KENYA** | | **RWANDA** | | **SOUTH AFRICA** | | **UGANDA** | | **ZAMBIA** | | **EUAM** | | **AFAM** | |
| --- | --- | --- | --- | --- | --- | --- | --- | --- | --- | --- | --- | --- | --- |
| A~B | HF | A~B | HF | A~B | HF | A~B | HF | A~B | HF | A~B | HF | A~B | HF |
| A*01:01~B*07:02 | 0.0092 | A*01:01~B*07:05 | 0.0116 | A*01:01~B*07:02 | 0.0003 | A*01:01~B*07:05 | 0.0043 | A*01:01~B*08:01 | 0.0009 | A*01:01~B*07:02 | 0.0074 | A*01:01~B*08:01 | 0.0149 |
| A*01:01~B*15:16 | 0.0092 | A*01:01~B*15:16 | 0.0029 | A*01:01~B*08:01 | 0.0005 | A*01:01~B*08:01 | 0.0022 | A*01:01~B*15:10 | 0.0009 | A*01:01~B*07:05 | 0.0004 | A*01:01~B*15:10 | 0.0038 |
| A*01:01~B*35:01 | 0.0092 | A*01:01~B*37:01 | 0.0116 | A*01:01~B*15:03 | 0.0003 | A*01:01~B*15:03 | 0.0043 | A*01:01~B*49:01 | 0.0017 | A*01:01~B*08:01 | 0.0501 | A*01:01~B*37:01 | 0.0015 |
| A*01:01~B*41:01 | 0.0092 | A*01:01~B*41:01 | 0.0029 | A*01:01~B*15:08 | 0.0003 | A*01:01~B*18:03 | 0.0022 | A*01:01~B*51:01 | 0.0009 | A*01:01~B*13:02 | 0.0011 | A*01:01~B*38:01 | 0.0015 |
| A*01:01~B*44:15 | 0.0046 | A*01:01~B*44:03 | 0.0029 | A*01:01~B*15:10 | 0.0002 | A*01:01~B*37:01 | 0.0022 | A*01:01~B*53:01 | 0.0019 | A*01:01~B*14:02 | 0.0019 | A*01:01~B*39:10 | 0.0008 |
| A*01:01~B*45:01 | 0.0046 | A*01:01~B*44:15 | 0.0288 | A*01:01~B*15:16 | 0.0003 | A*01:01~B*42:01 | 0.0022 | A*01:01~B*57:01 | 0.0009 | A*01:01~B*15:01 | 0.0022 | A*01:01~B*40:06 | 0.0008 |
| A*01:01~B*49:01 | 0.0046 | A*01:01~B*45:01 | 0.0202 | A*01:01~B*15:22 | 0.0003 | A*01:01~B*44:15 | 0.0346 | A*01:01~B*81:01 | 0.0088 | A*01:01~B*15:17 | 0.0015 | A*01:01~B*44:02 | 0.0008 |
| A*01:01~B*58:01 | 0.0046 | A*01:01~B*49:01 | 0.0102 | A*01:01~B*15:220 | 0.0003 | A*01:01~B*45:01 | 0.0043 | A*01:02~B*35:01 | 0.0009 | A*01:01~B*18:01 | 0.0017 | A*01:01~B*45:01 | 0.0016 |
| A*01:01~B*81:01 | 0.0092 | A*01:01~B*57:03 | 0.0000 | A*01:01~B*18:01 | 0.0001 | A*01:01~B*57:03 | 0.0043 | A*01:03~B*15:03 | 0.0009 | A*01:01~B*27:02 | 0.0005 | A*01:01~B*51:01 | 0.0014 |
| A*01:03~B*49:01 | 0.0046 | A*01:01~B*58:01 | 0.0030 | A*01:01~B*47:01 | 0.0006 | A*01:01~B*58:01 | 0.0022 | A*01:03~B*15:16 | 0.0009 | A*01:01~B*27:05 | 0.0021 | A*01:01~B*52:01 | 0.0002 |
| A*01:03~B*57:02 | 0.0046 | A*01:01~B*58:02 | 0.0071 | A*01:01~B*51:01 | 0.0003 | A*01:01~B*81:01 | 0.0087 | A*01:03~B*51:01 | 0.0009 | A*01:01~B*35:01 | 0.0009 | A*01:01~B*57:01 | 0.0045 |
| A*02:01~B*15:03 | 0.0344 | A*01:01~B*81:01 | 0.0145 | A*01:01~B*58:01 | 0.0004 | A*01:02~B*44:03 | 0.0022 | A*02:01~B*07:02 | 0.0016 | A*01:01~B*35:02 | 0.0020 | A*01:01~B*57:03 | 0.0044 |
| A*02:01~B*18:01 | 0.0115 | A*01:02~B*58:01 | 0.0029 | A*01:01~B*58:02 | 0.0021 | A*01:02~B*49:01 | 0.0022 | A*02:01~B*14:01 | 0.0000 | A*01:01~B*35:03 | 0.0017 | A*01:01~B*58:02 | 0.0015 |
| A*02:01~B*40:12 | 0.0046 | A*01:03~B*07:02 | 0.0050 | A*01:01~B*81:00 | 0.0030 | A*01:02~B*53:01 | 0.0022 | A*02:01~B*15:03 | 0.0221 | A*01:01~B*35:08 | 0.0003 | A*01:01~B*81:01 | 0.0023 |
| A*02:01~B*40:16 | 0.0092 | A*01:03~B*49:01 | 0.0029 | A*01:01~B*81:01 | 0.0225 | A*01:02~B*58:01 | 0.0043 | A*02:01~B*15:10 | 0.0026 | A*01:01~B*37:01 | 0.0064 | A*01:01~B*82:01 | 0.0008 |
| A*02:01~B*41:02 | 0.0046 | A*01:03~B*58:01 | 0.0007 | A*01:02~B*81:01 | 0.0003 | A*01:03~B*57:02 | 0.0043 | A*02:01~B*18:01 | 0.0019 | A*01:01~B*38:01 | 0.0005 | A*01:02~B*07:02 | 0.0008 |
| A*02:01~B*45:01 | 0.0275 | A*01:09~B*57:03 | 0.0029 | A*01:23~B*07:05 | 0.0003 | A*02:01~B*15:03 | 0.0426 | A*02:01~B*39:10 | 0.0017 | A*01:01~B*39:06 | 0.0009 | A*01:02~B*49:01 | 0.0015 |
| A*02:01~B*49:01 | 0.0092 | A*02:01~B*07:02 | 0.0087 | A*01:23~B*58:01 | 0.0018 | A*02:01~B*15:17 | 0.0070 | A*02:01~B*42:01 | 0.0049 | A*01:01~B*40:01 | 0.0027 | A*01:02~B*58:01 | 0.0015 |
| A*02:01~B*53:01 | 0.0092 | A*02:01~B*14:02 | 0.0148 | A*01:23~B*58:02 | 0.0003 | A*02:01~B*15:31 | 0.0022 | A*02:01~B*42:02 | 0.0017 | A*01:01~B*40:02 | 0.0003 | A*01:03~B*41:02 | 0.0008 |
| A*02:01~B*58:01 | 0.0092 | A*02:01~B*15:03 | 0.0513 | A*02:01~B*08:01 | 0.0028 | A*02:01~B*35:01 | 0.0043 | A*02:01~B*45:01 | 0.0232 | A*01:01~B*41:01 | 0.0015 | A*01:09~B*15:03 | 0.0008 |
| A*02:01~B*58:02 | 0.0046 | A*02:01~B*15:10 | 0.0029 | A*02:01~B*14:02 | 0.0001 | A*02:01~B*35:02 | 0.0022 | A*02:01~B*49:01 | 0.0052 | A*01:01~B*44:02 | 0.0005 | A*01:09~B*44:15 | 0.0008 |
| A*02:02~B*15:03 | 0.0138 | A*02:01~B*18:01 | 0.0029 | A*02:01~B*15:03 | 0.0040 | A*02:01~B*40:12 | 0.0043 | A*02:01~B*51:01 | 0.0072 | A*01:01~B*44:03 | 0.0018 | A*01:22N~B*52:01 | 0.0008 |
| A*02:02~B*41:01 | 0.0046 | A*02:01~B*35:01 | 0.0058 | A*02:01~B*15:10 | 0.0031 | A*02:01~B*40:16 | 0.0022 | A*02:01~B*53:01 | 0.0096 | A*01:01~B*45:01 | 0.0005 | A*02:01~B*07:02 | 0.0054 |
| A*02:02~B*42:01 | 0.0046 | A*02:01~B*45:01 | 0.0087 | A*02:01~B*15:13 | 0.0006 | A*02:01~B*41:01 | 0.0043 | A*02:01~B*57:03 | 0.0057 | A*01:01~B*46:01 | 0.0003 | A*02:01~B*07:09 | 0.0008 |
| A*02:02~B*53:01 | 0.0092 | A*02:01~B*47:01 | 0.0030 | A*02:01~B*15:16 | 0.0045 | A*02:01~B*45:01 | 0.0238 | A*02:01~B*58:01 | 0.0014 | A*01:01~B*49:01 | 0.0005 | A*02:01~B*08:01 | 0.0031 |
| A*02:02~B*58:01 | 0.0092 | A*02:01~B*49:01 | 0.0030 | A*02:01~B*15:22 | 0.0003 | A*02:01~B*49:01 | 0.0090 | A*02:01~B*58:02 | 0.0003 | A*01:01~B*50:01 | 0.0002 | A*02:01~B*13:02 | 0.0030 |
| A*02:02~B*58:02 | 0.0046 | A*02:01~B*51:01 | 0.0060 | A*02:01~B*18:01 | 0.0029 | A*02:01~B*51:01 | 0.0047 | A*02:01~B*81:01 | 0.0022 | A*01:01~B*51:01 | 0.0024 | A*02:01~B*14:01 | 0.0008 |
| A*02:05~B*27:03 | 0.0046 | A*02:01~B*53:01 | 0.0194 | A*02:01~B*35:01 | 0.0007 | A*02:01~B*53:01 | 0.0076 | A*02:02~B*07:02 | 0.0009 | A*01:01~B*52:01 | 0.0056 | A*02:01~B*14:02 | 0.0012 |
| A*02:05~B*35:01 | 0.0046 | A*02:01~B*57:03 | 0.0029 | A*02:01~B*40:01 | 0.0003 | A*02:01~B*57:03 | 0.0078 | A*02:02~B*08:01 | 0.0018 | A*01:01~B*55:01 | 0.0005 | A*02:01~B*15:01 | 0.0058 |
| A*02:05~B*42:01 | 0.0046 | A*02:01~B*58:02 | 0.0065 | A*02:01~B*42:01 | 0.0022 | A*02:01~B*58:01 | 0.0144 | A*02:02~B*14:02 | 0.0022 | A*01:01~B*56:01 | 0.0004 | A*02:01~B*15:03 | 0.0038 |
| A*02:05~B*44:03 | 0.0046 | A*02:01~B*73:01 | 0.0029 | A*02:01~B*42:02 | 0.0003 | A*02:02~B*07:02 | 0.0022 | A*02:02~B*15:03 | 0.0031 | A*01:01~B*57:01 | 0.0345 | A*02:01~B*15:10 | 0.0009 |
| A*02:05~B*45:01 | 0.0092 | A*02:02~B*07:02 | 0.0029 | A*02:01~B*44:03 | 0.0025 | A*02:02~B*14:02 | 0.0022 | A*02:02~B*18:01 | 0.0029 | A*01:01~B*57:02 | 0.0003 | A*02:01~B*15:16 | 0.0013 |
| A*02:05~B*49:01 | 0.0046 | A*02:02~B*15:03 | 0.0032 | A*02:01~B*45:00 | 0.0003 | A*02:02~B*18:01 | 0.0022 | A*02:02~B*40:16 | 0.0009 | A*01:01~B*57:03 | 0.0013 | A*02:01~B*18:01 | 0.0010 |
| A*02:05~B*58:01 | 0.0138 | A*02:02~B*15:16 | 0.0058 | A*02:01~B*45:01 | 0.0097 | A*02:02~B*35:01 | 0.0043 | A*02:02~B*42:01 | 0.0007 | A*01:01~B*58:01 | 0.0020 | A*02:01~B*27:05 | 0.0061 |
| A*02:14~B*73:01 | 0.0046 | A*02:02~B*18:01 | 0.0058 | A*02:01~B*45:07 | 0.0039 | A*02:02~B*51:01 | 0.0043 | A*02:02~B*44:03 | 0.0043 | A*01:01~B*67:01 | 0.0003 | A*02:01~B*35:01 | 0.0035 |
| A*03:01~B*15:03 | 0.0046 | A*02:02~B*41:01 | 0.0029 | A*02:01~B*49:01 | 0.0019 | A*02:02~B*58:02 | 0.0303 | A*02:02~B*51:01 | 0.0044 | A*01:02~B*15:01 | 0.0003 | A*02:01~B*39:01 | 0.0008 |
| A*03:01~B*47:03 | 0.0046 | A*02:02~B*51:01 | 0.0056 | A*02:01~B*51:01 | 0.0006 | A*02:04~B*53:01 | 0.0022 | A*02:02~B*53:01 | 0.0063 | A*01:02~B*51:08 | 0.0003 | A*02:01~B*39:10 | 0.0064 |
| A*03:01~B*49:01 | 0.0092 | A*02:02~B*58:01 | 0.0115 | A*02:01~B*53:01 | 0.0021 | A*02:05~B*49:01 | 0.0022 | A*02:02~B*57:01 | 0.0009 | A*01:03~B*35:187 | 0.0003 | A*02:01~B*39:24 | 0.0008 |
| A*03:01~B*57:03 | 0.0046 | A*02:02~B*58:02 | 0.0433 | A*02:01~B*57:02 | 0.0003 | A*02:05~B*58:01 | 0.0022 | A*02:02~B*57:02 | 0.0009 | A*01:03~B*57:02 | 0.0003 | A*02:01~B*40:01 | 0.0021 |
| A*11:01~B*35:01 | 0.0046 | A*02:05~B*07:05 | 0.0029 | A*02:01~B*57:03 | 0.0005 | A*02:14~B*14:02 | 0.0022 | A*02:02~B*58:01 | 0.0018 | A*02:01~B*07:02 | 0.0246 | A*02:01~B*42:01 | 0.0070 |
| A*11:01~B*44:03 | 0.0046 | A*02:05~B*27:03 | 0.0058 | A*02:01~B*58:01 | 0.0036 | A*02:14~B*18:03 | 0.0022 | A*02:02~B*58:02 | 0.0070 | A*02:01~B*08:01 | 0.0052 | A*02:01~B*44:02 | 0.0045 |
| A*23:01~B*13:02 | 0.0046 | A*02:05~B*58:01 | 0.0058 | A*02:01~B*58:02 | 0.0033 | A*02:14~B*49:01 | 0.0022 | A*02:04~B*51:01 | 0.0009 | A*02:01~B*13:02 | 0.0079 | A*02:01~B*44:03 | 0.0057 |
| A*23:01~B*14:02 | 0.0046 | A*02:14~B*18:01 | 0.0029 | A*02:01~B*58:10 | 0.0003 | A*03:01~B*14:02 | 0.0022 | A*02:05~B*07:02 | 0.0017 | A*02:01~B*14:01 | 0.0030 | A*02:01~B*45:01 | 0.0087 |
| A*23:01~B*15:10 | 0.0046 | A*02:14~B*18:03 | 0.0029 | A*02:01~B*81:00 | 0.0003 | A*03:01~B*15:10 | 0.0082 | A*02:05~B*15:03 | 0.0018 | A*02:01~B*14:02 | 0.0062 | A*02:01~B*49:01 | 0.0028 |
| A*23:01~B*15:17 | 0.0046 | A*03:01~B*14:02 | 0.0112 | A*02:02~B*14:01 | 0.0009 | A*03:01~B*18:01 | 0.0065 | A*02:05~B*18:01 | 0.0010 | A*02:01~B*15:01 | 0.0234 | A*02:01~B*50:01 | 0.0008 |
| A*23:01~B*44:03 | 0.0046 | A*03:01~B*15:03 | 0.0055 | A*02:02~B*15:03 | 0.0012 | A*03:01~B*35:01 | 0.0065 | A*02:05~B*35:01 | 0.0009 | A*02:01~B*15:10 | 0.0002 | A*02:01~B*51:01 | 0.0083 |
| A*23:01~B*57:02 | 0.0046 | A*03:01~B*15:10 | 0.0037 | A*02:02~B*15:16 | 0.0036 | A*03:01~B*47:01 | 0.0043 | A*02:05~B*42:01 | 0.0010 | A*02:01~B*15:16 | 0.0003 | A*02:01~B*52:01 | 0.0057 |
| A*23:01~B*58:02 | 0.0046 | A*03:01~B*35:01 | 0.0029 | A*02:02~B*18:01 | 0.0022 | A*03:01~B*49:01 | 0.0108 | A*02:05~B*45:01 | 0.0009 | A*02:01~B*15:17 | 0.0006 | A*02:01~B*53:01 | 0.0082 |
| A*23:02~B*14:02 | 0.0046 | A*03:01~B*41:01 | 0.0029 | A*02:02~B*42:01 | 0.0003 | A*03:01~B*53:01 | 0.0029 | A*02:05~B*58:01 | 0.0113 | A*02:01~B*15:31 | 0.0003 | A*02:01~B*57:02 | 0.0021 |
| A*24:02~B*07:02 | 0.0046 | A*03:01~B*47:01 | 0.0056 | A*02:02~B*44:03 | 0.0003 | A*03:01~B*57:03 | 0.0019 | A*02:14~B*14:01 | 0.0009 | A*02:01~B*18:01 | 0.0085 | A*02:01~B*57:03 | 0.0044 |
| A*24:02~B*18:01 | 0.0046 | A*03:01~B*49:01 | 0.0121 | A*02:02~B*53:01 | 0.0006 | A*03:01~B*58:01 | 0.0065 | A*02:14~B*18:03 | 0.0009 | A*02:01~B*18:20 | 0.0003 | A*02:01~B*58:01 | 0.0020 |
| A*24:02~B*57:03 | 0.0046 | A*03:01~B*53:01 | 0.0008 | A*02:02~B*57:03 | 0.0039 | A*03:01~B*58:02 | 0.0065 | A*03new~B*15:01 | 0.0009 | A*02:01~B*27:02 | 0.0013 | A*02:01~B*58:02 | 0.0010 |
| A*26:01~B*15:31 | 0.0046 | A*03:01~B*57:03 | 0.0058 | A*02:02~B*58:01 | 0.0004 | A*23:01~B*07:02 | 0.0130 | A*03:01~B*08:01 | 0.0009 | A*02:01~B*27:03 | 0.0003 | A*02:01~B*78:01 | 0.0003 |
| A*26:01~B*50:01 | 0.0046 | A*03:01~B*58:01 | 0.0029 | A*02:02~B*81:00 | 0.0003 | A*23:01~B*08:01 | 0.0065 | A*03:01~B*13:03 | 0.0009 | A*02:01~B*27:05 | 0.0151 | A*02:02~B*07:02 | 0.0019 |
| A*26:01~B*53:01 | 0.0092 | A*03:01~B*58:02 | 0.0103 | A*02:05~B*07:02 | 0.0012 | A*23:01~B*15:03 | 0.0028 | A*03:01~B*14:01 | 0.0024 | A*02:01~B*35:01 | 0.0044 | A*02:02~B*15:03 | 0.0018 |
| A*26:01~B*57:03 | 0.0046 | A*23:01~B*07:02 | 0.0029 | A*02:05~B*08:01 | 0.0025 | A*23:01~B*15:10 | 0.0087 | A*03:01~B*15:03 | 0.0102 | A*02:01~B*35:03 | 0.0023 | A*02:02~B*15:10 | 0.0008 |
| A*29:02~B*13:02 | 0.0092 | A*23:01~B*08:01 | 0.0087 | A*02:05~B*13:02 | 0.0006 | A*23:01~B*15:17 | 0.0017 | A*03:01~B*15:10 | 0.0045 | A*02:01~B*35:43 | 0.0003 | A*02:02~B*15:16 | 0.0015 |
| A*29:02~B*42:01 | 0.0183 | A*23:01~B*14:03 | 0.0029 | A*02:05~B*14:01 | 0.0103 | A*23:01~B*39:10 | 0.0027 | A*03:01~B*15:16 | 0.0009 | A*02:01~B*37:01 | 0.0010 | A*02:02~B*18:01 | 0.0016 |
| A*29:02~B*44:03 | 0.0046 | A*23:01~B*15:03 | 0.0059 | A*02:05~B*15:01 | 0.0003 | A*23:01~B*42:01 | 0.0047 | A*03:01~B*35:01 | 0.0009 | A*02:01~B*38:01 | 0.0043 | A*02:02~B*35:01 | 0.0046 |
| A*29:02~B*45:01 | 0.0138 | A*23:01~B*15:17 | 0.0029 | A*02:05~B*15:03 | 0.0000 | A*23:01~B*45:01 | 0.0139 | A*03:01~B*42:01 | 0.0012 | A*02:01~B*39:01 | 0.0027 | A*02:02~B*42:01 | 0.0009 |
| A*29:02~B*58:02 | 0.0046 | A*23:01~B*18:01 | 0.0087 | A*02:05~B*15:10 | 0.0051 | A*23:01~B*51:01 | 0.0040 | A*03:01~B*44:03 | 0.0011 | A*02:01~B*39:06 | 0.0008 | A*02:02~B*45:01 | 0.0009 |
| A*30:01~B*15:10 | 0.0046 | A*23:01~B*42:01 | 0.0029 | A*02:05~B*15:18 | 0.0003 | A*23:01~B*53:01 | 0.0048 | A*03:01~B*45:01 | 0.0009 | A*02:01~B*39:10 | 0.0006 | A*02:02~B*49:01 | 0.0013 |
| A*30:01~B*42:01 | 0.0413 | A*23:01~B*44:03 | 0.0087 | A*02:05~B*18:01 | 0.0010 | A*23:01~B*58:01 | 0.0025 | A*03:01~B*49:01 | 0.0008 | A*02:01~B*40:01 | 0.0166 | A*02:02~B*51:01 | 0.0015 |
| A*30:01~B*44:15 | 0.0046 | A*23:01~B*45:01 | 0.0058 | A*02:05~B*35:01 | 0.0003 | A*23:01~B*58:02 | 0.0061 | A*03:01~B*53:01 | 0.0017 | A*02:01~B*40:02 | 0.0051 | A*02:02~B*53:01 | 0.0088 |
| A*30:01~B*57:03 | 0.0138 | A*23:01~B*49:01 | 0.0143 | A*02:05~B*41:01 | 0.0011 | A*26:01~B*15:17 | 0.0022 | A*03:01~B*57:03 | 0.0011 | A*02:01~B*40:04 | 0.0003 | A*02:02~B*57:03 | 0.0031 |
| A*30:01~B*81:01 | 0.0046 | A*23:01~B*53:01 | 0.0087 | A*02:05~B*42:01 | 0.0009 | A*26:01~B*41:01 | 0.0022 | A*03:01~B*58:01 | 0.0012 | A*02:01~B*41:01 | 0.0017 | A*02:02~B*58:01 | 0.0037 |
| A*30:02~B*14:02 | 0.0092 | A*23:01~B*57:03 | 0.0058 | A*02:05~B*42:02 | 0.0003 | A*26:01~B*44:03 | 0.0022 | A*03:01~B*58:02 | 0.0173 | A*02:01~B*44:02 | 0.0453 | A*02:02~B*81:01 | 0.0040 |
| A*30:02~B*15:03 | 0.0069 | A*23:01~B*81:01 | 0.0058 | A*02:05~B*44:03 | 0.0016 | A*26:01~B*49:01 | 0.0050 | A*23:01~B*07:02 | 0.0048 | A*02:01~B*44:03 | 0.0085 | A*02:04~B*41:01 | 0.0008 |
| A*30:02~B*15:10 | 0.0094 | A*24:02~B*15:03 | 0.0029 | A*02:05~B*45:00 | 0.0003 | A*26:01~B*58:01 | 0.0037 | A*23:01~B*08:01 | 0.0094 | A*02:01~B*44:05 | 0.0012 | A*02:04~B*51:01 | 0.0015 |
| A*30:02~B*18:01 | 0.0023 | A*24:02~B*35:01 | 0.0029 | A*02:05~B*45:07 | 0.0007 | A*26:01~B*81:01 | 0.0022 | A*23:01~B*13:02 | 0.0006 | A*02:01~B*44:27 | 0.0003 | A*02:05~B*07:05 | 0.0008 |
| A*30:02~B*18:03 | 0.0046 | A*24:02~B*35:02 | 0.0029 | A*02:05~B*53:01 | 0.0003 | A*29:01~B*42:01 | 0.0022 | A*23:01~B*14:01 | 0.0025 | A*02:01~B*45:01 | 0.0030 | A*02:05~B*08:01 | 0.0008 |
| A*30:02~B*39:10 | 0.0046 | A*24:02~B*41:01 | 0.0029 | A*02:05~B*57:02 | 0.0000 | A*29:02~B*07:02 | 0.0087 | A*23:01~B*14:02 | 0.0081 | A*02:01~B*48:01 | 0.0006 | A*02:05~B*14:02 | 0.0008 |
| A*30:02~B*42:02 | 0.0046 | A*24:02~B*44:03 | 0.0029 | A*02:05~B*58:01 | 0.0211 | A*29:02~B*15:16 | 0.0022 | A*23:01~B*14:03 | 0.0009 | A*02:01~B*48:02 | 0.0003 | A*02:05~B*27:03 | 0.0008 |
| A*30:02~B*44:03 | 0.0046 | A*26:01~B*18:01 | 0.0029 | A*02:05~B*58:02 | 0.0035 | A*29:02~B*18:01 | 0.0065 | A*23:01~B*15:03 | 0.0119 | A*02:01~B*49:01 | 0.0028 | A*02:05~B*37:01 | 0.0008 |
| A*30:02~B*45:01 | 0.0273 | A*26:01~B*58:02 | 0.0029 | A*02:05~B*81:01 | 0.0002 | A*29:02~B*42:01 | 0.0024 | A*23:01~B*15:10 | 0.0059 | A*02:01~B*51:01 | 0.0201 | A*02:05~B*44:03 | 0.0010 |
| A*30:02~B*52:01 | 0.0046 | A*26:03~B*56:01 | 0.0029 | A*02:11~B*15:18 | 0.0003 | A*29:02~B*44:03 | 0.0019 | A*23:01~B*15:17 | 0.0009 | A*02:01~B*51:05 | 0.0003 | A*02:05~B*45:01 | 0.0008 |
| A*30:02~B*53:01 | 0.0000 | A*26:12~B*58:01 | 0.0029 | A*02:11~B*51:01 | 0.0003 | A*29:02~B*44:15 | 0.0022 | A*23:01~B*18:01 | 0.0035 | A*02:01~B*52:01 | 0.0044 | A*02:05~B*53:01 | 0.0008 |
| A*30:02~B*58:01 | 0.0046 | A*26:12~B*58:02 | 0.0058 | A*02:14~B*14:01 | 0.0003 | A*29:02~B*45:01 | 0.0050 | A*23:01~B*41:01 | 0.0009 | A*02:01~B*53:01 | 0.0003 | A*02:05~B*57:03 | 0.0068 |
| A*30:04~B*41:02 | 0.0046 | A*29:02~B*07:02 | 0.0029 | A*02:14~B*44:03 | 0.0027 | A*29:02~B*53:01 | 0.0036 | A*23:01~B*42:01 | 0.0014 | A*02:01~B*55:01 | 0.0015 | A*02:05~B*58:01 | 0.0035 |
| A*30:04~B*45:01 | 0.0092 | A*29:02~B*13:02 | 0.0058 | A*02:14~B*58:02 | 0.0003 | A*29:02~B*57:02 | 0.0022 | A*23:01~B*44:03 | 0.0142 | A*02:01~B*56:01 | 0.0027 | A*02:05~B*58:11 | 0.0008 |
| A*30:04~B*57:03 | 0.0138 | A*29:02~B*15:03 | 0.0058 | A*03:01~B*08:01 | 0.0138 | A*29:02~B*58:01 | 0.0022 | A*23:01~B*45:01 | 0.0178 | A*02:01~B*57:01 | 0.0225 | A*02:06~B*27:05 | 0.0030 |
| A*31:04~B*07:02 | 0.0046 | A*29:02~B*35:01 | 0.0058 | A*03:01~B*13:02 | 0.0006 | A*29:02~B*73:01 | 0.0022 | A*23:01~B*51:01 | 0.0010 | A*02:01~B*57:03 | 0.0008 | A*02:06~B*27:06 | 0.0008 |
| A*33:01~B*14:02 | 0.0046 | A*29:02~B*42:01 | 0.0029 | A*03:01~B*13:03 | 0.0012 | A*30:01~B*07:02 | 0.0023 | A*23:01~B*53:01 | 0.0105 | A*02:01~B*58:01 | 0.0019 | A*02:11~B*39:05 | 0.0008 |
| A*33:01~B*15:16 | 0.0046 | A*29:02~B*45:01 | 0.0058 | A*03:01~B*15:03 | 0.0019 | A*30:01~B*08:01 | 0.0022 | A*23:01~B*57:03 | 0.0043 | A*02:02~B*40:02 | 0.0003 | A*02:16~B*40:06 | 0.0008 |
| A*33:01~B*42:01 | 0.0046 | A*29:02~B*58:02 | 0.0029 | A*03:01~B*15:10 | 0.0081 | A*30:01~B*13:02 | 0.0041 | A*23:01~B*58:01 | 0.0092 | A*02:02~B*41:01 | 0.0006 | A*03:01~B*07:02 | 0.0144 |
| A*33:03~B*14:02 | 0.0046 | A*30:01~B*15:10 | 0.0116 | A*03:01~B*39:10 | 0.0008 | A*30:01~B*14:02 | 0.0038 | A*23:01~B*58:02 | 0.0054 | A*02:02~B*53:01 | 0.0003 | A*03:01~B*07:05 | 0.0008 |
| A*34:02~B*08:01 | 0.0046 | A*30:01~B*27:03 | 0.0029 | A*03:01~B*40:01 | 0.0003 | A*30:01~B*15:10 | 0.0027 | A*23:01~B*81:01 | 0.0009 | A*02:02~B*57:03 | 0.0006 | A*03:01~B*08:01 | 0.0015 |
| A*34:02~B*15:03 | 0.0046 | A*30:01~B*39:10 | 0.0087 | A*03:01~B*42:01 | 0.0028 | A*30:01~B*15:37 | 0.0022 | A*23:02~B*39:01 | 0.0009 | A*02:04~B*51:01 | 0.0014 | A*03:01~B*14:01 | 0.0030 |
| A*34:02~B*53:01 | 0.0046 | A*30:01~B*40:16 | 0.0029 | A*03:01~B*44:03 | 0.0023 | A*30:01~B*18:01 | 0.0043 | A*23:02~B*42:01 | 0.0009 | A*02:04~B*57:01 | 0.0003 | A*03:01~B*14:02 | 0.0031 |
| A*34:02~B*81:01 | 0.0046 | A*30:01~B*42:01 | 0.0173 | A*03:01~B*45:01 | 0.0004 | A*30:01~B*42:01 | 0.0231 | A*24:02~B*13:02 | 0.0009 | A*02:05~B*14:02 | 0.0006 | A*03:01~B*15:01 | 0.0008 |
| A*36:01~B*53:01 | 0.0275 | A*30:01~B*58:02 | 0.0029 | A*03:01~B*49:01 | 0.0042 | A*30:01~B*42:02 | 0.0043 | A*24:02~B*15:03 | 0.0012 | A*02:05~B*35:02 | 0.0003 | A*03:01~B*15:03 | 0.0019 |
| A*66:01~B*35:01 | 0.0046 | A*30:02~B*07:02 | 0.0045 | A*03:01~B*57:03 | 0.0007 | A*30:01~B*44:03 | 0.0024 | A*24:02~B*15:16 | 0.0009 | A*02:05~B*35:43 | 0.0006 | A*03:01~B*15:10 | 0.0029 |
| A*66:01~B*58:02 | 0.0183 | A*30:02~B*08:01 | 0.0023 | A*03:01~B*58:01 | 0.0011 | A*30:01~B*45:01 | 0.0087 | A*24:02~B*18:01 | 0.0009 | A*02:05~B*39:05 | 0.0003 | A*03:01~B*15:16 | 0.0019 |
| A*68:01~B*07:02 | 0.0138 | A*30:02~B*13:02 | 0.0029 | A*03:01~B*58:02 | 0.0202 | A*30:01~B*47:03 | 0.0022 | A*24:02~B*35:02 | 0.0009 | A*02:05~B*41:02 | 0.0003 | A*03:01~B*18:01 | 0.0043 |
| A*68:01~B*14:02 | 0.0046 | A*30:02~B*14:02 | 0.0058 | A*03:01~B*81:00 | 0.0004 | A*30:01~B*58:01 | 0.0043 | A*24:02~B*41:01 | 0.0018 | A*02:05~B*44:03 | 0.0002 | A*03:01~B*27:05 | 0.0015 |
| A*68:01~B*15:17 | 0.0046 | A*30:02~B*15:03 | 0.0087 | A*03:01~B*81:01 | 0.0012 | A*30:01~B*58:02 | 0.0069 | A*24:02~B*44:03 | 0.0041 | A*02:05~B*49:01 | 0.0017 | A*03:01~B*35:01 | 0.0075 |
| A*68:01~B*15:31 | 0.0046 | A*30:02~B*15:10 | 0.0029 | A*03:02~B*08:01 | 0.0003 | A*30:01~B*81:01 | 0.0022 | A*24:31~B*15:10 | 0.0009 | A*02:05~B*50:01 | 0.0017 | A*03:01~B*35:03 | 0.0008 |
| A*68:01~B*37:01 | 0.0046 | A*30:02~B*18:01 | 0.0058 | A*03:21~B*42:01 | 0.0003 | A*30:02~B*08:01 | 0.0065 | A*26:01~B*07:02 | 0.0018 | A*02:05~B*52:01 | 0.0004 | A*03:01~B*39:10 | 0.0019 |
| A*68:01~B*58:02 | 0.0183 | A*30:02~B*39:10 | 0.0029 | A*11:01~B*13:01 | 0.0003 | A*30:02~B*13:02 | 0.0022 | A*26:01~B*15:220 | 0.0027 | A*02:05~B*53:01 | 0.0003 | A*03:01~B*39:20 | 0.0008 |
| A*68:02~B*07:02 | 0.0413 | A*30:02~B*42:01 | 0.0058 | A*11:01~B*35:03 | 0.0003 | A*30:02~B*14:02 | 0.0130 | A*26:01~B*35:01 | 0.0009 | A*02:05~B*56:01 | 0.0003 | A*03:01~B*40:01 | 0.0032 |
| A*68:02~B*14:01 | 0.0046 | A*30:02~B*45:01 | 0.0029 | A*11:01~B*44:02 | 0.0003 | A*30:02~B*15:03 | 0.0043 | A*26:01~B*81:01 | 0.0009 | A*02:05~B*57:03 | 0.0008 | A*03:01~B*40:02 | 0.0015 |
| A*68:02~B*15:10 | 0.0548 | A*30:02~B*49:01 | 0.0048 | A*11:01~B*52:01 | 0.0003 | A*30:02~B*15:10 | 0.0130 | A*26:12~B*45:01 | 0.0009 | A*02:05~B*58:01 | 0.0022 | A*03:01~B*41:02 | 0.0023 |
| A*68:02~B*27:03 | 0.0046 | A*30:02~B*53:01 | 0.0058 | A*23new~B*44:03 | 0.0003 | A*30:02~B*18:01 | 0.0025 | A*29:01~B*14:01 | 0.0009 | A*02:06~B*13:02 | 0.0004 | A*03:01~B*44:03 | 0.0072 |
| A*68:02~B*45:01 | 0.0049 | A*30:02~B*57:03 | 0.0087 | A*23:01~B*07:02 | 0.0030 | A*30:02~B*39:10 | 0.0065 | A*29:01~B*58:02 | 0.0009 | A*02:06~B*15:01 | 0.0003 | A*03:01~B*44:05 | 0.0008 |
| A*68:02~B*49:01 | 0.0092 | A*30:02~B*58:01 | 0.0058 | A*23:01~B*07:05 | 0.0011 | A*30:02~B*42:01 | 0.0022 | A*29:01~B*82:01 | 0.0009 | A*02:06~B*15:11 | 0.0003 | A*03:01~B*47:01 | 0.0008 |
| A*68:02~B*53:01 | 0.0275 | A*30:02~B*58:02 | 0.0058 | A*23:01~B*08:01 | 0.0160 | A*30:02~B*45:01 | 0.0043 | A*29:02~B*07:02 | 0.0010 | A*02:06~B*15:24 | 0.0003 | A*03:01~B*49:01 | 0.0040 |
| A*68:02~B*73:01 | 0.0046 | A*30:04~B*07:02 | 0.0029 | A*23:01~B*13:02 | 0.0003 | A*30:02~B*49:01 | 0.0022 | A*29:02~B*13:02 | 0.0071 | A*02:06~B*27:05 | 0.0006 | A*03:01~B*51:01 | 0.0036 |
| A*74:01~B*15:03 | 0.0183 | A*30:04~B*08:01 | 0.0029 | A*23:01~B*14:01 | 0.0023 | A*30:02~B*53:01 | 0.0083 | A*29:02~B*15:03 | 0.0068 | A*02:06~B*35:01 | 0.0006 | A*03:01~B*52:01 | 0.0030 |
| A*74:01~B*35:01 | 0.0092 | A*30:04~B*44:03 | 0.0029 | A*23:01~B*14:02 | 0.0060 | A*30:02~B*57:03 | 0.0087 | A*29:02~B*15:31 | 0.0009 | A*02:06~B*35:17 | 0.0003 | A*03:01~B*57:01 | 0.0046 |
| A*74:01~B*44:03 | 0.0046 | A*30:09~B*81:01 | 0.0029 | A*23:01~B*15:01 | 0.0006 | A*30:02~B*81:01 | 0.0022 | A*29:02~B*35:01 | 0.0051 | A*02:06~B*37:01 | 0.0003 | A*03:01~B*57:02 | 0.0030 |
| A*74:01~B*45:01 | 0.0046 | A*31:01~B*53:01 | 0.0029 | A*23:01~B*15:03 | 0.0088 | A*30:04~B*14:02 | 0.0022 | A*29:02~B*39:10 | 0.0008 | A*02:06~B*39:06 | 0.0003 | A*03:01~B*57:03 | 0.0066 |
| A*74:01~B*49:01 | 0.0046 | A*32:01~B*53:01 | 0.0029 | A*23:01~B*15:10 | 0.0230 | A*30:04~B*41:01 | 0.0022 | A*29:02~B*42:01 | 0.0124 | A*02:06~B*39:08 | 0.0003 | A*03:01~B*58:01 | 0.0033 |
| A*74:01~B*51:01 | 0.0092 | A*33:01~B*53:01 | 0.0029 | A*23:01~B*18:01 | 0.0012 | A*30:04~B*53:01 | 0.0022 | A*29:02~B*42:02 | 0.0009 | A*02:06~B*44:02 | 0.0003 | A*03:01~B*58:02 | 0.0008 |
| A*74:01~B*58:01 | 0.0275 | A*33:03~B*15:16 | 0.0029 | A*23:01~B*35:01 | 0.0001 | A*30:04~B*58:02 | 0.0022 | A*29:02~B*44:03 | 0.0098 | A*02:06~B*48:01 | 0.0008 | A*03:01~B*58:11 | 0.0008 |
|  |  | A*33:03~B*44:15 | 0.0058 | A*23:01~B*35:02 | 0.0006 | A*30:09~B*81:01 | 0.0087 | A*29:02~B*45:01 | 0.0078 | A*02:06~B*51:01 | 0.0006 | A*03:01~B*81:01 | 0.0022 |
|  |  | A*34:02~B*40:12 | 0.0029 | A*23:01~B*39:10 | 0.0029 | A*31:01~B*82:02 | 0.0022 | A*29:02~B*53:01 | 0.0019 | A*02:09~B*35:03 | 0.0003 | A*03:02~B*35:03 | 0.0008 |
|  |  | A*34:02~B*44:03 | 0.0116 | A*23:01~B*41:01 | 0.0035 | A*31:03~B*15:16 | 0.0022 | A*29:02~B*57:03 | 0.0019 | A*02:09~B*58:01 | 0.0003 | A*11:01~B*15:01 | 0.0008 |
|  |  | A*34:02~B*53:01 | 0.0029 | A*23:01~B*42:01 | 0.0035 | A*31:04~B*15:10 | 0.0022 | A*29:02~B*58:01 | 0.0048 | A*02:11~B*45:01 | 0.0003 | A*11:01~B*18:01 | 0.0008 |
|  |  | A*34:02~B*57:03 | 0.0029 | A*23:01~B*42:02 | 0.0003 | A*31:04~B*51:01 | 0.0022 | A*29:02~B*58:02 | 0.0009 | A*02:11~B*48:02 | 0.0003 | A*11:01~B*27:05 | 0.0008 |
|  |  | A*36:01~B*13:02 | 0.0029 | A*23:01~B*44:03 | 0.0083 | A*32:01~B*07:02 | 0.0022 | A*29:02~B*81:01 | 0.0009 | A*02:11~B*52:01 | 0.0014 | A*11:01~B*35:01 | 0.0045 |
|  |  | A*36:01~B*15:17 | 0.0029 | A*23:01~B*45:01 | 0.0040 | A*32:01~B*39:10 | 0.0022 | A*30:01~B*07:02 | 0.0033 | A*02:11~B*52:02 | 0.0003 | A*11:01~B*35:04 | 0.0008 |
|  |  | A*36:01~B*42:01 | 0.0058 | A*23:01~B*53:01 | 0.0027 | A*32:01~B*40:12 | 0.0022 | A*30:01~B*13:02 | 0.0009 | A*02:17~B*08:01 | 0.0003 | A*11:01~B*37:01 | 0.0008 |
|  |  | A*36:01~B*45:01 | 0.0058 | A*23:01~B*57:02 | 0.0026 | A*32:01~B*47:01 | 0.0022 | A*30:01~B*15:03 | 0.0084 | A*02:17~B*15:40 | 0.0003 | A*11:01~B*39:01 | 0.0008 |
|  |  | A*36:01~B*49:01 | 0.0029 | A*23:01~B*58:01 | 0.0059 | A*32:01~B*81:01 | 0.0065 | A*30:01~B*15:10 | 0.0036 | A*02:17~B*51:01 | 0.0006 | A*11:01~B*40:01 | 0.0008 |
|  |  | A*36:01~B*53:01 | 0.0116 | A*23:01~B*58:02 | 0.0058 | A*33:01~B*14:03 | 0.0022 | A*30:01~B*15:47 | 0.0027 | A*02:20~B*27:05 | 0.0003 | A*11:01~B*40:02 | 0.0008 |
|  |  | A*66:01~B*44:03 | 0.0029 | A*23:01~B*81:01 | 0.0011 | A*33:01~B*15:10 | 0.0022 | A*30:01~B*18:01 | 0.0005 | A*02:22~B*41:01 | 0.0003 | A*11:01~B*42:01 | 0.0008 |
|  |  | A*66:01~B*58:01 | 0.0116 | A*24:02~B*07:02 | 0.0188 | A*33:01~B*15:16 | 0.0022 | A*30:01~B*35:01 | 0.0011 | A*02:274~B*13:02 | 0.0003 | A*11:01~B*44:02 | 0.0030 |
|  |  | A*66:01~B*58:02 | 0.0116 | A*24:02~B*08:01 | 0.0020 | A*33:01~B*42:01 | 0.0065 | A*30:01~B*39:10 | 0.0002 | A*02:30~B*38:01 | 0.0003 | A*11:01~B*51:01 | 0.0008 |
|  |  | A*68:01~B*15:16 | 0.0029 | A*24:02~B*15:10 | 0.0007 | A*33:01~B*44:03 | 0.0022 | A*30:01~B*40:16 | 0.0018 | A*02:724~B*15:03 | 0.0003 | A*11:01~B*51:08 | 0.0008 |
|  |  | A*68:01~B*15:17 | 0.0029 | A*24:02~B*15:16 | 0.0003 | A*33:01~B*81:01 | 0.0022 | A*30:01~B*42:01 | 0.0480 | A*03:01~B*07:02 | 0.0423 | A*11:02~B*40:01 | 0.0008 |
|  |  | A*68:01~B*40:16 | 0.0029 | A*24:02~B*18:01 | 0.0008 | A*33:03~B*15:03 | 0.0043 | A*30:01~B*42:02 | 0.0062 | A*03:01~B*07:04 | 0.0003 | A*23:01~B*07:02 | 0.0067 |
|  |  | A*68:02~B*07:02 | 0.0252 | A*24:02~B*35:01 | 0.0003 | A*33:03~B*15:31 | 0.0022 | A*30:01~B*45:01 | 0.0023 | A*03:01~B*07:05 | 0.0006 | A*23:01~B*08:01 | 0.0028 |
|  |  | A*68:02~B*08:01 | 0.0035 | A*24:02~B*35:02 | 0.0003 | A*33:03~B*53:01 | 0.0065 | A*30:01~B*49:01 | 0.0009 | A*03:01~B*08:01 | 0.0042 | A*23:01~B*14:01 | 0.0019 |
|  |  | A*68:02~B*13:02 | 0.0029 | A*24:02~B*38:02 | 0.0003 | A*34:02~B*08:01 | 0.0022 | A*30:01~B*50:01 | 0.0009 | A*03:01~B*13:02 | 0.0015 | A*23:01~B*14:02 | 0.0097 |
|  |  | A*68:02~B*15:10 | 0.0108 | A*24:02~B*40:01 | 0.0003 | A*34:02~B*15:03 | 0.0022 | A*30:01~B*53:01 | 0.0024 | A*03:01~B*14:01 | 0.0010 | A*23:01~B*14:03 | 0.0015 |
|  |  | A*68:02~B*39:10 | 0.0029 | A*24:02~B*40:06 | 0.0009 | A*34:02~B*15:16 | 0.0022 | A*30:01~B*57:03 | 0.0073 | A*03:01~B*14:02 | 0.0117 | A*23:01~B*15:03 | 0.0068 |
|  |  | A*68:02~B*42:01 | 0.0029 | A*24:02~B*42:01 | 0.0006 | A*34:02~B*18:01 | 0.0039 | A*30:01~B*81:01 | 0.0017 | A*03:01~B*15:01 | 0.0075 | A*23:01~B*15:10 | 0.0006 |
|  |  | A*68:02~B*44:15 | 0.0030 | A*24:02~B*44:03 | 0.0006 | A*34:02~B*27:03 | 0.0022 | A*30:02~B*07:02 | 0.0044 | A*03:01~B*15:10 | 0.0003 | A*23:01~B*15:16 | 0.0018 |
|  |  | A*68:02~B*49:01 | 0.0133 | A*24:02~B*51:01 | 0.0003 | A*34:02~B*35:01 | 0.0043 | A*30:02~B*07:05 | 0.0009 | A*03:01~B*15:18 | 0.0003 | A*23:01~B*39:01 | 0.0008 |
|  |  | A*68:02~B*53:01 | 0.0058 | A*24:02~B*55:01 | 0.0006 | A*34:02~B*44:03 | 0.0022 | A*30:02~B*08:01 | 0.0142 | A*03:01~B*18:01 | 0.0041 | A*23:01~B*42:01 | 0.0011 |
|  |  | A*68:02~B*58:01 | 0.0107 | A*24:03~B*07:02 | 0.0003 | A*34:02~B*44:15 | 0.0022 | A*30:02~B*14:01 | 0.0021 | A*03:01~B*18:05 | 0.0003 | A*23:01~B*44:03 | 0.0043 |
|  |  | A*68:02~B*81:01 | 0.0029 | A*24:07~B*44:03 | 0.0003 | A*34:02~B*53:01 | 0.0028 | A*30:02~B*14:02 | 0.0176 | A*03:01~B*27:02 | 0.0004 | A*23:01~B*44:10 | 0.0008 |
|  |  | A*74:01~B*15:03 | 0.0265 | A*26:01~B*07:04 | 0.0003 | A*34:02~B*81:01 | 0.0019 | A*30:02~B*14:03 | 0.0009 | A*03:01~B*27:05 | 0.0069 | A*23:01~B*45:01 | 0.0045 |
|  |  | A*74:01~B*35:01 | 0.0145 | A*26:01~B*07:05 | 0.0021 | A*34:02~B*82:02 | 0.0022 | A*30:02~B*15:10 | 0.0018 | A*03:01~B*35:01 | 0.0138 | A*23:01~B*49:01 | 0.0008 |
|  |  | A*74:01~B*42:01 | 0.0029 | A*26:01~B*08:01 | 0.0016 | A*36:01~B*35:01 | 0.0012 | A*30:02~B*18:01 | 0.0135 | A*03:01~B*35:03 | 0.0017 | A*23:01~B*50:01 | 0.0015 |
|  |  | A*74:01~B*44:03 | 0.0029 | A*26:01~B*14:01 | 0.0009 | A*36:01~B*49:01 | 0.0075 | A*30:02~B*35:01 | 0.0009 | A*03:01~B*37:01 | 0.0009 | A*23:01~B*51:01 | 0.0008 |
|  |  | A*74:01~B*49:01 | 0.0203 | A*26:01~B*15:03 | 0.0008 | A*36:01~B*53:01 | 0.0299 | A*30:02~B*39:10 | 0.0028 | A*03:01~B*38:01 | 0.0017 | A*23:01~B*51:64 | 0.0008 |
|  |  | A*74:01~B*51:01 | 0.0029 | A*26:01~B*15:10 | 0.0004 | A*36:01~B*57:03 | 0.0024 | A*30:02~B*41:01 | 0.0009 | A*03:01~B*39:06 | 0.0004 | A*23:01~B*53:01 | 0.0098 |
|  |  | A*74:01~B*57:03 | 0.0058 | A*26:01~B*15:22 | 0.0003 | A*36:01~B*81:01 | 0.0024 | A*30:02~B*42:01 | 0.0019 | A*03:01~B*40:01 | 0.0037 | A*23:01~B*57:03 | 0.0066 |
|  |  | A*74:01~B*58:01 | 0.0030 | A*26:01~B*39:10 | 0.0006 | A*66:01~B*15:03 | 0.0043 | A*30:02~B*42:02 | 0.0010 | A*03:01~B*40:02 | 0.0010 | A*23:01~B*58:01 | 0.0062 |
|  |  | A*74:01~B*58:02 | 0.0110 | A*26:01~B*41:01 | 0.0003 | A*66:01~B*18:01 | 0.0043 | A*30:02~B*45:01 | 0.0096 | A*03:01~B*40:08 | 0.0003 | A*23:01~B*58:02 | 0.0008 |
|  |  | A*74:02~B*51:01 | 0.0029 | A*26:01~B*42:01 | 0.0006 | A*66:01~B*44:03 | 0.0065 | A*30:02~B*49:01 | 0.0010 | A*03:01~B*41:02 | 0.0003 | A*23:01~B*81:01 | 0.0082 |
|  |  | A*74:05~B*58:02 | 0.0029 | A*26:01~B*51:01 | 0.0052 | A*66:01~B*57:01 | 0.0022 | A*30:02~B*51:01 | 0.0009 | A*03:01~B*44:02 | 0.0044 | A*24:02~B*13:02 | 0.0015 |
|  |  | A*80:01~B*15:10 | 0.0029 | A*26:01~B*58:01 | 0.0003 | A*66:01~B*58:02 | 0.0130 | A*30:02~B*53:01 | 0.0065 | A*03:01~B*44:03 | 0.0008 | A*24:02~B*15:01 | 0.0008 |
|  |  |  |  | A*26:01~B*58:02 | 0.0007 | A*66:01~B*81:01 | 0.0043 | A*30:02~B*57:02 | 0.0062 | A*03:01~B*44:05 | 0.0002 | A*24:02~B*15:07 | 0.0008 |
|  |  |  |  | A*26:01~B*81:00 | 0.0003 | A*66:02~B*14:02 | 0.0022 | A*30:02~B*57:03 | 0.0298 | A*03:01~B*47:01 | 0.0003 | A*24:02~B*15:10 | 0.0008 |
|  |  |  |  | A*26:01~B*81:01 | 0.0052 | A*66:02~B*81:01 | 0.0022 | A*30:02~B*58:01 | 0.0106 | A*03:01~B*49:01 | 0.0004 | A*24:02~B*18:01 | 0.0014 |
|  |  |  |  | A*26:01~B*81:03 | 0.0003 | A*68:01~B*08:01 | 0.0043 | A*30:02~B*81:01 | 0.0034 | A*03:01~B*50:01 | 0.0009 | A*24:02~B*35:08 | 0.0008 |
|  |  |  |  | A*26:12~B*41:01 | 0.0015 | A*68:01~B*15:17 | 0.0022 | A*30:04~B*15:03 | 0.0009 | A*03:01~B*51:01 | 0.0050 | A*24:02~B*40:02 | 0.0015 |
|  |  |  |  | A*26:12~B*51:01 | 0.0003 | A*68:01~B*18:01 | 0.0022 | A*30:04~B*15:16 | 0.0009 | A*03:01~B*55:01 | 0.0008 | A*24:02~B*40:06 | 0.0008 |
|  |  |  |  | A*26:121~B*15:10 | 0.0006 | A*68:02~B*07:02 | 0.0280 | A*30:04~B*15:83 | 0.0009 | A*03:01~B*57:01 | 0.0056 | A*24:02~B*41:02 | 0.0015 |
|  |  |  |  | A*29:01~B*14:02 | 0.0003 | A*68:02~B*13:02 | 0.0045 | A*30:04~B*53:01 | 0.0009 | A*03:01~B*57:02 | 0.0003 | A*24:02~B*44:02 | 0.0008 |
|  |  |  |  | A*29:01~B*15:03 | 0.0004 | A*68:02~B*14:02 | 0.0070 | A*30:04~B*58:02 | 0.0027 | A*03:01~B*57:03 | 0.0004 | A*24:02~B*44:03 | 0.0009 |
|  |  |  |  | A*29:01~B*15:22 | 0.0003 | A*68:02~B*15:10 | 0.0151 | A*30:04~B*73:01 | 0.0018 | A*03:02~B*08:01 | 0.0011 | A*24:02~B*45:01 | 0.0008 |
|  |  |  |  | A*29:01~B*18:01 | 0.0024 | A*68:02~B*15:16 | 0.0022 | A*30:09~B*15:10 | 0.0009 | A*03:02~B*35:01 | 0.0003 | A*24:02~B*50:01 | 0.0008 |
|  |  |  |  | A*29:01~B*44:03 | 0.0011 | A*68:02~B*27:03 | 0.0022 | A*30:09~B*18:01 | 0.0009 | A*03:02~B*51:01 | 0.0008 | A*24:02~B*52:01 | 0.0008 |
|  |  |  |  | A*29:01~B*58:01 | 0.0003 | A*68:02~B*39:10 | 0.0016 | A*30:09~B*45:01 | 0.0035 | A*03:02~B*55:01 | 0.0003 | A*24:02~B*56:01 | 0.0008 |
|  |  |  |  | A*29:02~B*07:02 | 0.0015 | A*68:02~B*44:03 | 0.0022 | A*30:09~B*53:01 | 0.0010 | A*03:05~B*07:02 | 0.0003 | A*24:02~B*57:03 | 0.0008 |
|  |  |  |  | A*29:02~B*07:05 | 0.0004 | A*68:02~B*45:01 | 0.0027 | A*30:09~B*81:01 | 0.0017 | A*03:21N~B*57:02 | 0.0003 | A*24:02~B*81:01 | 0.0037 |
|  |  |  |  | A*29:02~B*08:01 | 0.0004 | A*68:02~B*53:01 | 0.0190 | A*31:01~B*18:01 | 0.0009 | A*03:49~B*27:05 | 0.0003 | A*24:03~B*07:02 | 0.0008 |
|  |  |  |  | A*29:02~B*13:02 | 0.0015 | A*68:02~B*57:02 | 0.0043 | A*31:01~B*41:02 | 0.0009 | A*11:01~B*07:02 | 0.0035 | A*24:03~B*38:01 | 0.0008 |
|  |  |  |  | A*29:02~B*14:02 | 0.0003 | A*68:02~B*57:03 | 0.0043 | A*31:04~B*44:03 | 0.0009 | A*11:01~B*07:05 | 0.0003 | A*24:03~B*44:10 | 0.0008 |
|  |  |  |  | A*29:02~B*15:03 | 0.0055 | A*68:02~B*58:01 | 0.0043 | A*32:01~B*15:16 | 0.0009 | A*11:01~B*14:02 | 0.0036 | A*24:07~B*35:05 | 0.0008 |
|  |  |  |  | A*29:02~B*15:10 | 0.0055 | A*74:01~B*14:01 | 0.0022 | A*32:01~B*39:10 | 0.0018 | A*11:01~B*15:01 | 0.0031 | A*25:01~B*18:01 | 0.0030 |
|  |  |  |  | A*29:02~B*15:220 | 0.0003 | A*74:01~B*15:03 | 0.0173 | A*32:01~B*45:01 | 0.0009 | A*11:01~B*15:02 | 0.0006 | A*25:01~B*27:03 | 0.0008 |
|  |  |  |  | A*29:02~B*35:01 | 0.0003 | A*74:01~B*35:01 | 0.0031 | A*33:01~B*14:01 | 0.0000 | A*11:01~B*15:03 | 0.0003 | A*25:01~B*39:01 | 0.0008 |
|  |  |  |  | A*29:02~B*39:10 | 0.0017 | A*74:01~B*49:01 | 0.0240 | A*33:01~B*14:02 | 0.0009 | A*11:01~B*15:17 | 0.0003 | A*25:01~B*44:02 | 0.0008 |
|  |  |  |  | A*29:02~B*41:01 | 0.0000 | A*74:01~B*53:01 | 0.0056 | A*33:01~B*15:10 | 0.0018 | A*11:01~B*15:18 | 0.0003 | A*25:01~B*44:03 | 0.0008 |
|  |  |  |  | A*29:02~B*42:01 | 0.0149 | A*74:01~B*57:03 | 0.0095 | A*33:01~B*15:16 | 0.0009 | A*11:01~B*18:01 | 0.0021 | A*26:01~B*08:01 | 0.0023 |
|  |  |  |  | A*29:02~B*44:03 | 0.0285 | A*74:01~B*58:01 | 0.0075 | A*33:01~B*35:01 | 0.0008 | A*11:01~B*27:02 | 0.0002 | A*26:01~B*15:01 | 0.0008 |
|  |  |  |  | A*29:02~B*45:01 | 0.0043 | A*74:01~B*58:02 | 0.0152 | A*33:01~B*42:01 | 0.0026 | A*11:01~B*27:05 | 0.0059 | A*26:01~B*15:17 | 0.0008 |
|  |  |  |  | A*29:02~B*58:01 | 0.0012 | A*74:03~B*57:02 | 0.0022 | A*33:01~B*45:01 | 0.0009 | A*11:01~B*27:13 | 0.0003 | A*26:01~B*27:02 | 0.0008 |
|  |  |  |  | A*29:02~B*58:02 | 0.0025 | A*80:01~B*18:01 | 0.0022 | A*33:01~B*53:01 | 0.0017 | A*11:01~B*35:01 | 0.0127 | A*26:01~B*27:05 | 0.0015 |
|  |  |  |  | A*29:02~B*81:01 | 0.0002 | A*80:01~B*58:01 | 0.0043 | A*33:01~B*57:03 | 0.0010 | A*11:01~B*35:02 | 0.0007 | A*26:01~B*37:01 | 0.0008 |
|  |  |  |  | A*29:11~B*13:02 | 0.0122 |  |  | A*33:01~B*58:01 | 0.0018 | A*11:01~B*35:03 | 0.0021 | A*26:01~B*39:10 | 0.0008 |
|  |  |  |  | A*29:11~B*15:10 | 0.0009 |  |  | A*33:03~B*15:03 | 0.0010 | A*11:01~B*35:08 | 0.0009 | A*26:01~B*44:03 | 0.0008 |
|  |  |  |  | A*29:11~B*41:01 | 0.0003 |  |  | A*33:03~B*15:10 | 0.0026 | A*11:01~B*35:42 | 0.0003 | A*26:01~B*48:01 | 0.0008 |
|  |  |  |  | A*29:11~B*44:03 | 0.0003 |  |  | A*33:03~B*15:16 | 0.0009 | A*11:01~B*37:01 | 0.0012 | A*26:01~B*57:02 | 0.0008 |
|  |  |  |  | A*29:11~B*57:03 | 0.0003 |  |  | A*33:03~B*15:17 | 0.0018 | A*11:01~B*38:01 | 0.0017 | A*26:01~B*58:01 | 0.0008 |
|  |  |  |  | A*30:01~B*07:02 | 0.0015 |  |  | A*33:03~B*37:01 | 0.0009 | A*11:01~B*39:01 | 0.0007 | A*26:01~B*81:01 | 0.0015 |
|  |  |  |  | A*30:01~B*08:01 | 0.0021 |  |  | A*33:03~B*45:01 | 0.0009 | A*11:01~B*39:02 | 0.0003 | A*29:01~B*55:01 | 0.0008 |
|  |  |  |  | A*30:01~B*13:02 | 0.0002 |  |  | A*33:03~B*50:01 | 0.0018 | A*11:01~B*39:10 | 0.0003 | A*29:02~B*07:02 | 0.0028 |
|  |  |  |  | A*30:01~B*15:03 | 0.0087 |  |  | A*33:03~B*53:01 | 0.0016 | A*11:01~B*39:24 | 0.0003 | A*29:02~B*15:01 | 0.0010 |
|  |  |  |  | A*30:01~B*15:16 | 0.0003 |  |  | A*33:03~B*58:01 | 0.0009 | A*11:01~B*40:01 | 0.0009 | A*29:02~B*15:03 | 0.0017 |
|  |  |  |  | A*30:01~B*15:17 | 0.0009 |  |  | A*34:02~B*07:05 | 0.0009 | A*11:01~B*40:02 | 0.0009 | A*29:02~B*15:16 | 0.0008 |
|  |  |  |  | A*30:01~B*18:01 | 0.0058 |  |  | A*34:02~B*08:01 | 0.0020 | A*11:01~B*41:02 | 0.0003 | A*29:02~B*15:31 | 0.0008 |
|  |  |  |  | A*30:01~B*39:10 | 0.0011 |  |  | A*34:02~B*15:03 | 0.0025 | A*11:01~B*44:02 | 0.0044 | A*29:02~B*27:03 | 0.0015 |
|  |  |  |  | A*30:01~B*42:01 | 0.0465 |  |  | A*34:02~B*15:17 | 0.0035 | A*11:01~B*44:03 | 0.0013 | A*29:02~B*35:01 | 0.0016 |
|  |  |  |  | A*30:01~B*42:02 | 0.0146 |  |  | A*34:02~B*35:01 | 0.0034 | A*11:01~B*49:01 | 0.0008 | A*29:02~B*35:43 | 0.0008 |
|  |  |  |  | A*30:01~B*44:03 | 0.0033 |  |  | A*34:02~B*40:16 | 0.0009 | A*11:01~B*51:01 | 0.0038 | A*29:02~B*39:10 | 0.0009 |
|  |  |  |  | A*30:01~B*45:01 | 0.0012 |  |  | A*34:02~B*42:01 | 0.0006 | A*11:01~B*51:07 | 0.0004 | A*29:02~B*41:01 | 0.0015 |
|  |  |  |  | A*30:01~B*53:01 | 0.0007 |  |  | A*34:02~B*44:03 | 0.0210 | A*11:01~B*52:01 | 0.0040 | A*29:02~B*44:03 | 0.0017 |
|  |  |  |  | A*30:01~B*57:03 | 0.0019 |  |  | A*34:02~B*53:01 | 0.0026 | A*11:01~B*55:01 | 0.0020 | A*29:02~B*49:01 | 0.0044 |
|  |  |  |  | A*30:01~B*58:01 | 0.0041 |  |  | A*34:02~B*58:01 | 0.0013 | A*11:01~B*56:01 | 0.0009 | A*29:02~B*50:01 | 0.0008 |
|  |  |  |  | A*30:01~B*58:02 | 0.0031 |  |  | A*34:02~B*58:02 | 0.0014 | A*11:01~B*57:01 | 0.0022 | A*29:02~B*52:01 | 0.0015 |
|  |  |  |  | A*30:01~B*81:00 | 0.0006 |  |  | A*34:02~B*58:15 | 0.0009 | A*11:01~B*58:01 | 0.0008 | A*29:02~B*53:01 | 0.0024 |
|  |  |  |  | A*30:01~B*81:01 | 0.0031 |  |  | A*34:02~B*73:01 | 0.0009 | A*11:04~B*15:01 | 0.0003 | A*29:02~B*57:02 | 0.0013 |
|  |  |  |  | A*30:01~B*82:02 | 0.0000 |  |  | A*34:02~B*81:01 | 0.0007 | A*11:04~B*35:01 | 0.0003 | A*29:02~B*81:01 | 0.0064 |
|  |  |  |  | A*30:02~B*07:02 | 0.0034 |  |  | A*36:01~B*07:02 | 0.0017 | A*11:67~B*08:01 | 0.0003 | A*30:01~B*07:02 | 0.0055 |
|  |  |  |  | A*30:02~B*08:01 | 0.0089 |  |  | A*36:01~B*14:01 | 0.0009 | A*23:01~B*07:02 | 0.0009 | A*30:01~B*07:05 | 0.0008 |
|  |  |  |  | A*30:02~B*14:01 | 0.0003 |  |  | A*36:01~B*15:10 | 0.0022 | A*23:01~B*07:05 | 0.0003 | A*30:01~B*14:02 | 0.0018 |
|  |  |  |  | A*30:02~B*14:02 | 0.0024 |  |  | A*36:01~B*15:220 | 0.0009 | A*23:01~B*08:01 | 0.0009 | A*30:01~B*15:16 | 0.0010 |
|  |  |  |  | A*30:02~B*15:03 | 0.0028 |  |  | A*36:01~B*35:01 | 0.0010 | A*23:01~B*14:02 | 0.0003 | A*30:01~B*18:01 | 0.0012 |
|  |  |  |  | A*30:02~B*15:10 | 0.0051 |  |  | A*36:01~B*42:01 | 0.0026 | A*23:01~B*15:03 | 0.0006 | A*30:01~B*35:01 | 0.0021 |
|  |  |  |  | A*30:02~B*18:01 | 0.0055 |  |  | A*36:01~B*44:03 | 0.0010 | A*23:01~B*15:24 | 0.0003 | A*30:01~B*42:01 | 0.0169 |
|  |  |  |  | A*30:02~B*39:10 | 0.0077 |  |  | A*36:01~B*45:01 | 0.0019 | A*23:01~B*18:01 | 0.0003 | A*30:01~B*42:02 | 0.0030 |
|  |  |  |  | A*30:02~B*42:01 | 0.0092 |  |  | A*36:01~B*45:07 | 0.0009 | A*23:01~B*35:01 | 0.0006 | A*30:01~B*44:03 | 0.0032 |
|  |  |  |  | A*30:02~B*42:02 | 0.0003 |  |  | A*36:01~B*49:01 | 0.0009 | A*23:01~B*35:12 | 0.0003 | A*30:01~B*45:01 | 0.0038 |
|  |  |  |  | A*30:02~B*44:03 | 0.0007 |  |  | A*36:01~B*51:01 | 0.0017 | A*23:01~B*37:01 | 0.0003 | A*30:01~B*49:01 | 0.0008 |
|  |  |  |  | A*30:02~B*45:01 | 0.0093 |  |  | A*36:01~B*53:01 | 0.0272 | A*23:01~B*38:01 | 0.0003 | A*30:01~B*52:01 | 0.0015 |
|  |  |  |  | A*30:02~B*53:01 | 0.0008 |  |  | A*36:01~B*58:02 | 0.0023 | A*23:01~B*39:01 | 0.0003 | A*30:01~B*53:01 | 0.0027 |
|  |  |  |  | A*30:02~B*57:02 | 0.0009 |  |  | A*36:01~B*81:01 | 0.0009 | A*23:01~B*41:01 | 0.0003 | A*30:01~B*57:03 | 0.0081 |
|  |  |  |  | A*30:02~B*57:03 | 0.0028 |  |  | A*43:01~B*14:02 | 0.0009 | A*23:01~B*41:02 | 0.0008 | A*30:02~B*07:02 | 0.0009 |
|  |  |  |  | A*30:02~B*58:01 | 0.0004 |  |  | A*43:01~B*42:01 | 0.0009 | A*23:01~B*42:01 | 0.0003 | A*30:02~B*08:01 | 0.0065 |
|  |  |  |  | A*30:02~B*58:02 | 0.0114 |  |  | A*66:01~B*15:10 | 0.0011 | A*23:01~B*44:03 | 0.0053 | A*30:02~B*14:01 | 0.0008 |
|  |  |  |  | A*30:02~B*82:02 | 0.0003 |  |  | A*66:01~B*18:01 | 0.0009 | A*23:01~B*49:01 | 0.0028 | A*30:02~B*14:02 | 0.0032 |
|  |  |  |  | A*30:03~B*15:03 | 0.0003 |  |  | A*66:01~B*39:10 | 0.0041 | A*23:01~B*50:01 | 0.0011 | A*30:02~B*15:03 | 0.0054 |
|  |  |  |  | A*30:04~B*07:02 | 0.0021 |  |  | A*66:01~B*41:01 | 0.0009 | A*23:01~B*50:02 | 0.0003 | A*30:02~B*15:10 | 0.0016 |
|  |  |  |  | A*30:04~B*07:05 | 0.0006 |  |  | A*66:01~B*44:03 | 0.0035 | A*23:01~B*51:01 | 0.0003 | A*30:02~B*15:17 | 0.0008 |
|  |  |  |  | A*30:04~B*08:01 | 0.0026 |  |  | A*66:01~B*45:01 | 0.0020 | A*23:01~B*53:01 | 0.0008 | A*30:02~B*18:01 | 0.0034 |
|  |  |  |  | A*30:04~B*14:01 | 0.0016 |  |  | A*66:01~B*49:01 | 0.0009 | A*23:01~B*57:03 | 0.0003 | A*30:02~B*27:03 | 0.0015 |
|  |  |  |  | A*30:04~B*15:03 | 0.0002 |  |  | A*66:01~B*51:01 | 0.0025 | A*23:01~B*58:01 | 0.0005 | A*30:02~B*27:05 | 0.0008 |
|  |  |  |  | A*30:04~B*15:16 | 0.0006 |  |  | A*66:01~B*53:01 | 0.0055 | A*24:02~B*07:02 | 0.0077 | A*30:02~B*35:01 | 0.0040 |
|  |  |  |  | A*30:04~B*18:01 | 0.0003 |  |  | A*66:01~B*58:01 | 0.0011 | A*24:02~B*07:05 | 0.0003 | A*30:02~B*39:10 | 0.0027 |
|  |  |  |  | A*30:04~B*27:05 | 0.0012 |  |  | A*66:01~B*58:02 | 0.0103 | A*24:02~B*08:01 | 0.0030 | A*30:02~B*40:16 | 0.0008 |
|  |  |  |  | A*30:04~B*39:10 | 0.0003 |  |  | A*66:02~B*18:01 | 0.0009 | A*24:02~B*13:02 | 0.0025 | A*30:02~B*42:01 | 0.0008 |
|  |  |  |  | A*30:04~B*41:01 | 0.0018 |  |  | A*66:02~B*58:01 | 0.0018 | A*24:02~B*14:02 | 0.0019 | A*30:02~B*44:03 | 0.0013 |
|  |  |  |  | A*30:04~B*41:02 | 0.0006 |  |  | A*66:03~B*15:10 | 0.0018 | A*24:02~B*15:01 | 0.0046 | A*30:02~B*45:01 | 0.0054 |
|  |  |  |  | A*30:04~B*42:01 | 0.0003 |  |  | A*68:01~B*08:01 | 0.0009 | A*24:02~B*15:03 | 0.0003 | A*30:02~B*50:01 | 0.0008 |
|  |  |  |  | A*30:04~B*44:03 | 0.0025 |  |  | A*68:01~B*14:02 | 0.0018 | A*24:02~B*15:07 | 0.0006 | A*30:02~B*53:01 | 0.0062 |
|  |  |  |  | A*30:04~B*45:01 | 0.0000 |  |  | A*68:01~B*14:03 | 0.0009 | A*24:02~B*15:17 | 0.0023 | A*30:02~B*57:01 | 0.0014 |
|  |  |  |  | A*30:04~B*51:01 | 0.0006 |  |  | A*68:01~B*15:03 | 0.0009 | A*24:02~B*15:24 | 0.0003 | A*30:02~B*57:02 | 0.0015 |
|  |  |  |  | A*30:04~B*53:01 | 0.0003 |  |  | A*68:01~B*15:10 | 0.0009 | A*24:02~B*18:01 | 0.0040 | A*30:02~B*57:03 | 0.0267 |
|  |  |  |  | A*30:04~B*58:02 | 0.0029 |  |  | A*68:01~B*35:01 | 0.0035 | A*24:02~B*27:02 | 0.0014 | A*30:02~B*58:01 | 0.0002 |
|  |  |  |  | A*30:04~B*82:02 | 0.0027 |  |  | A*68:01~B*40:16 | 0.0027 | A*24:02~B*27:05 | 0.0034 | A*30:02~B*58:02 | 0.0034 |
|  |  |  |  | A*30:09~B*42:01 | 0.0003 |  |  | A*68:01~B*58:02 | 0.0009 | A*24:02~B*27:07 | 0.0008 | A*30:02~B*78:01 | 0.0027 |
|  |  |  |  | A*30:09~B*57:03 | 0.0003 |  |  | A*68:02~B*07:02 | 0.0113 | A*24:02~B*27:12 | 0.0003 | A*30:02~B*81:01 | 0.0030 |
|  |  |  |  | A*30:09~B*81:01 | 0.0040 |  |  | A*68:02~B*07:51 | 0.0009 | A*24:02~B*35:01 | 0.0027 | A*30:03~B*57:03 | 0.0008 |
|  |  |  |  | A*31:01~B*07:05 | 0.0003 |  |  | A*68:02~B*13:02 | 0.0011 | A*24:02~B*35:02 | 0.0030 | A*30:04~B*15:03 | 0.0008 |
|  |  |  |  | A*31:01~B*15:03 | 0.0009 |  |  | A*68:02~B*14:01 | 0.0157 | A*24:02~B*35:03 | 0.0043 | A*30:04~B*58:02 | 0.0008 |
|  |  |  |  | A*32:01~B*07:02 | 0.0013 |  |  | A*68:02~B*15:03 | 0.0037 | A*24:02~B*35:08 | 0.0009 | A*30:04~B*73:01 | 0.0008 |
|  |  |  |  | A*32:01~B*07:05 | 0.0006 |  |  | A*68:02~B*15:10 | 0.0275 | A*24:02~B*35:43 | 0.0006 | A*30:09~B*45:01 | 0.0008 |
|  |  |  |  | A*32:01~B*15:01 | 0.0009 |  |  | A*68:02~B*18:01 | 0.0033 | A*24:02~B*37:01 | 0.0013 | A*30:10~B*13:02 | 0.0008 |
|  |  |  |  | A*32:01~B*15:03 | 0.0003 |  |  | A*68:02~B*35:01 | 0.0014 | A*24:02~B*38:01 | 0.0037 | A*30:10~B*44:15 | 0.0008 |
|  |  |  |  | A*32:01~B*42:01 | 0.0005 |  |  | A*68:02~B*39:10 | 0.0010 | A*24:02~B*39:01 | 0.0014 | A*30:151~B*42:02 | 0.0008 |
|  |  |  |  | A*32:01~B*44:03 | 0.0006 |  |  | A*68:02~B*42:01 | 0.0082 | A*24:02~B*39:06 | 0.0017 | A*31:01~B*15:01 | 0.0008 |
|  |  |  |  | A*32:106~B*07:02 | 0.0007 |  |  | A*68:02~B*44:03 | 0.0021 | A*24:02~B*40:01 | 0.0030 | A*31:01~B*40:01 | 0.0053 |
|  |  |  |  | A*32:106~B*07:05 | 0.0006 |  |  | A*68:02~B*53:01 | 0.0102 | A*24:02~B*40:02 | 0.0036 | A*31:01~B*44:03 | 0.0015 |
|  |  |  |  | A*32:106~B*44:03 | 0.0003 |  |  | A*68:02~B*57:02 | 0.0027 | A*24:02~B*40:06 | 0.0003 | A*31:01~B*51:01 | 0.0023 |
|  |  |  |  | A*32:106~B*58:02 | 0.0003 |  |  | A*68:02~B*57:03 | 0.0012 | A*24:02~B*41:01 | 0.0003 | A*31:01~B*57:03 | 0.0098 |
|  |  |  |  | A*33:01~B*07:02 | 0.0001 |  |  | A*68:02~B*58:01 | 0.0068 | A*24:02~B*44:02 | 0.0020 | A*32:01~B*08:01 | 0.0015 |
|  |  |  |  | A*33:01~B*15:03 | 0.0007 |  |  | A*68:02~B*58:02 | 0.0010 | A*24:02~B*44:03 | 0.0022 | A*32:01~B*14:01 | 0.0015 |
|  |  |  |  | A*33:01~B*15:220 | 0.0003 |  |  | A*68:02~B*81:01 | 0.0019 | A*24:02~B*44:05 | 0.0003 | A*32:01~B*15:16 | 0.0008 |
|  |  |  |  | A*33:01~B*41:02 | 0.0003 |  |  | A*74:01~B*07:02 | 0.0011 | A*24:02~B*45:01 | 0.0001 | A*32:01~B*15:18 | 0.0008 |
|  |  |  |  | A*33:01~B*42:01 | 0.0053 |  |  | A*74:01~B*14:01 | 0.0031 | A*24:02~B*48:01 | 0.0006 | A*32:01~B*18:01 | 0.0023 |
|  |  |  |  | A*33:01~B*57:03 | 0.0001 |  |  | A*74:01~B*14:02 | 0.0012 | A*24:02~B*50:01 | 0.0004 | A*32:01~B*39:06 | 0.0008 |
|  |  |  |  | A*33:01~B*58:02 | 0.0001 |  |  | A*74:01~B*15:03 | 0.0273 | A*24:02~B*51:01 | 0.0039 | A*32:01~B*39:10 | 0.0006 |
|  |  |  |  | A*33:01~B*81:01 | 0.0003 |  |  | A*74:01~B*15:10 | 0.0012 | A*24:02~B*51:09 | 0.0008 | A*32:01~B*40:01 | 0.0008 |
|  |  |  |  | A*33:03~B*07:02 | 0.0049 |  |  | A*74:01~B*15:16 | 0.0009 | A*24:02~B*51:22 | 0.0003 | A*32:01~B*40:02 | 0.0015 |
|  |  |  |  | A*33:03~B*15:16 | 0.0003 |  |  | A*74:01~B*15:83 | 0.0009 | A*24:02~B*52:01 | 0.0006 | A*32:01~B*42:02 | 0.0008 |
|  |  |  |  | A*33:03~B*42:01 | 0.0034 |  |  | A*74:01~B*35:01 | 0.0146 | A*24:02~B*54:01 | 0.0003 | A*32:01~B*44:02 | 0.0015 |
|  |  |  |  | A*33:03~B*44:03 | 0.0004 |  |  | A*74:01~B*42:01 | 0.0040 | A*24:02~B*55:01 | 0.0021 | A*32:01~B*53:01 | 0.0012 |
|  |  |  |  | A*33:03~B*53:01 | 0.0054 |  |  | A*74:01~B*44:03 | 0.0026 | A*24:02~B*57:01 | 0.0057 | A*32:01~B*57:03 | 0.0020 |
|  |  |  |  | A*33:03~B*58:01 | 0.0003 |  |  | A*74:01~B*50:01 | 0.0026 | A*24:02~B*57:03 | 0.0005 | A*32:01~B*81:01 | 0.0008 |
|  |  |  |  | A*33:03~B*58:02 | 0.0003 |  |  | A*74:01~B*53:01 | 0.0041 | A*24:02~B*58:01 | 0.0003 | A*33:01~B*07:05 | 0.0008 |
|  |  |  |  | A*34:01~B*57:01 | 0.0003 |  |  | A*74:01~B*57:03 | 0.0008 | A*24:02~B*73:01 | 0.0003 | A*33:01~B*08:01 | 0.0005 |
|  |  |  |  | A*34:02~B*07:02 | 0.0003 |  |  | A*74:01~B*58:02 | 0.0028 | A*24:02~B*81:01 | 0.0003 | A*33:01~B*14:02 | 0.0043 |
|  |  |  |  | A*34:02~B*08:01 | 0.0041 |  |  | A*74:01~B*81:01 | 0.0018 | A*24:03~B*14:01 | 0.0003 | A*33:01~B*35:01 | 0.0023 |
|  |  |  |  | A*34:02~B*14:02 | 0.0003 |  |  | A*80:01~B*15:03 | 0.0009 | A*24:03~B*15:17 | 0.0006 | A*33:01~B*39:10 | 0.0008 |
|  |  |  |  | A*34:02~B*15:03 | 0.0055 |  |  | A*80:01~B*18:01 | 0.0009 | A*24:03~B*18:01 | 0.0006 | A*33:01~B*42:01 | 0.0023 |
|  |  |  |  | A*34:02~B*39:10 | 0.0004 |  |  | A*80:01~B*53:01 | 0.0018 | A*24:03~B*27:05 | 0.0003 | A*33:01~B*57:03 | 0.0010 |
|  |  |  |  | A*34:02~B*42:01 | 0.0012 |  |  | A*80:01~B*58:01 | 0.0009 | A*24:03~B*35:43 | 0.0006 | A*33:01~B*58:01 | 0.0023 |
|  |  |  |  | A*34:02~B*44:03 | 0.0239 |  |  |  |  | A*24:03~B*44:03 | 0.0003 | A*33:01~B*78:01 | 0.0023 |
|  |  |  |  | A*34:02~B*45:01 | 0.0004 |  |  |  |  | A*24:03~B*49:01 | 0.0003 | A*33:01~B*81:01 | 0.0009 |
|  |  |  |  | A*34:02~B*53:01 | 0.0005 |  |  |  |  | A*24:03~B*50:01 | 0.0003 | A*33:03~B*07:02 | 0.0010 |
|  |  |  |  | A*34:02~B*57:03 | 0.0003 |  |  |  |  | A*24:03~B*56:01 | 0.0003 | A*33:03~B*07:05 | 0.0015 |
|  |  |  |  | A*34:02~B*58:02 | 0.0040 |  |  |  |  | A*24:03~B*58:01 | 0.0003 | A*33:03~B*08:01 | 0.0015 |
|  |  |  |  | A*34:02~B*81:00 | 0.0006 |  |  |  |  | A*24:05~B*40:08 | 0.0003 | A*33:03~B*13:02 | 0.0030 |
|  |  |  |  | A*34:02~B*81:01 | 0.0021 |  |  |  |  | A*24:23~B*51:01 | 0.0003 | A*33:03~B*14:03 | 0.0008 |
|  |  |  |  | A*34:02~B*81:03 | 0.0003 |  |  |  |  | A*24:314~B*14:02 | 0.0003 | A*33:03~B*15:03 | 0.0023 |
|  |  |  |  | A*36:01~B*15:10 | 0.0007 |  |  |  |  | A*24:95~B*39:01 | 0.0003 | A*33:03~B*15:10 | 0.0039 |
|  |  |  |  | A*36:01~B*18:01 | 0.0003 |  |  |  |  | A*25:01~B*07:02 | 0.0015 | A*33:03~B*15:16 | 0.0056 |
|  |  |  |  | A*36:01~B*42:01 | 0.0007 |  |  |  |  | A*25:01~B*08:01 | 0.0031 | A*33:03~B*39:10 | 0.0023 |
|  |  |  |  | A*36:01~B*53:01 | 0.0024 |  |  |  |  | A*25:01~B*14:02 | 0.0013 | A*33:03~B*40:12 | 0.0008 |
|  |  |  |  | A*36:01~B*58:02 | 0.0001 |  |  |  |  | A*25:01~B*15:01 | 0.0016 | A*33:03~B*42:01 | 0.0038 |
|  |  |  |  | A*43:01~B*07:02 | 0.0005 |  |  |  |  | A*25:01~B*18:01 | 0.0114 | A*33:03~B*44:03 | 0.0010 |
|  |  |  |  | A*43:01~B*07:05 | 0.0006 |  |  |  |  | A*25:01~B*27:05 | 0.0016 | A*33:03~B*49:01 | 0.0031 |
|  |  |  |  | A*43:01~B*08:01 | 0.0003 |  |  |  |  | A*25:01~B*35:01 | 0.0007 | A*33:03~B*51:01 | 0.0009 |
|  |  |  |  | A*43:01~B*13:02 | 0.0003 |  |  |  |  | A*25:01~B*35:41 | 0.0003 | A*33:03~B*53:01 | 0.0172 |
|  |  |  |  | A*43:01~B*14:01 | 0.0012 |  |  |  |  | A*25:01~B*39:01 | 0.0018 | A*33:03~B*56:01 | 0.0008 |
|  |  |  |  | A*43:01~B*15:01 | 0.0003 |  |  |  |  | A*25:01~B*39:11 | 0.0003 | A*33:03~B*57:02 | 0.0007 |
|  |  |  |  | A*43:01~B*15:03 | 0.0080 |  |  |  |  | A*25:01~B*40:02 | 0.0006 | A*33:03~B*58:01 | 0.0090 |
|  |  |  |  | A*43:01~B*15:10 | 0.0012 |  |  |  |  | A*25:01~B*44:02 | 0.0014 | A*33:05~B*07:02 | 0.0008 |
|  |  |  |  | A*43:01~B*35:02 | 0.0006 |  |  |  |  | A*25:01~B*44:03 | 0.0003 | A*34:02~B*07:02 | 0.0016 |
|  |  |  |  | A*43:01~B*39:10 | 0.0007 |  |  |  |  | A*25:01~B*53:01 | 0.0003 | A*34:02~B*07:05 | 0.0008 |
|  |  |  |  | A*43:01~B*41:01 | 0.0005 |  |  |  |  | A*25:01~B*55:01 | 0.0003 | A*34:02~B*13:02 | 0.0015 |
|  |  |  |  | A*43:01~B*44:03 | 0.0019 |  |  |  |  | A*25:01~B*57:01 | 0.0008 | A*34:02~B*14:02 | 0.0008 |
|  |  |  |  | A*43:01~B*57:03 | 0.0005 |  |  |  |  | A*25:01~B*58:01 | 0.0020 | A*34:02~B*15:03 | 0.0030 |
|  |  |  |  | A*43:01~B*58:02 | 0.0105 |  |  |  |  | A*26:01~B*07:02 | 0.0015 | A*34:02~B*15:17 | 0.0008 |
|  |  |  |  | A*66:01~B*08:01 | 0.0018 |  |  |  |  | A*26:01~B*07:05 | 0.0003 | A*34:02~B*15:18 | 0.0008 |
|  |  |  |  | A*66:01~B*13:02 | 0.0004 |  |  |  |  | A*26:01~B*08:01 | 0.0003 | A*34:02~B*18:01 | 0.0022 |
|  |  |  |  | A*66:01~B*15:03 | 0.0014 |  |  |  |  | A*26:01~B*13:02 | 0.0012 | A*34:02~B*27:12 | 0.0008 |
|  |  |  |  | A*66:01~B*15:10 | 0.0003 |  |  |  |  | A*26:01~B*14:01 | 0.0010 | A*34:02~B*39:10 | 0.0016 |
|  |  |  |  | A*66:01~B*15:18 | 0.0003 |  |  |  |  | A*26:01~B*15:03 | 0.0002 | A*34:02~B*41:03 | 0.0008 |
|  |  |  |  | A*66:01~B*35:01 | 0.0008 |  |  |  |  | A*26:01~B*15:17 | 0.0003 | A*34:02~B*42:01 | 0.0004 |
|  |  |  |  | A*66:01~B*39:10 | 0.0045 |  |  |  |  | A*26:01~B*18:01 | 0.0021 | A*34:02~B*44:03 | 0.0109 |
|  |  |  |  | A*66:01~B*42:02 | 0.0003 |  |  |  |  | A*26:01~B*27:02 | 0.0006 | A*34:02~B*49:01 | 0.0015 |
|  |  |  |  | A*66:01~B*44:03 | 0.0015 |  |  |  |  | A*26:01~B*27:05 | 0.0030 | A*34:02~B*52:01 | 0.0009 |
|  |  |  |  | A*66:01~B*57:02 | 0.0003 |  |  |  |  | A*26:01~B*35:01 | 0.0023 | A*34:02~B*53:01 | 0.0045 |
|  |  |  |  | A*66:01~B*58:01 | 0.0003 |  |  |  |  | A*26:01~B*35:02 | 0.0003 | A*34:02~B*58:01 | 0.0009 |
|  |  |  |  | A*66:01~B*58:02 | 0.0270 |  |  |  |  | A*26:01~B*35:03 | 0.0003 | A*34:02~B*81:01 | 0.0026 |
|  |  |  |  | A*66:01~B*81:01 | 0.0006 |  |  |  |  | A*26:01~B*37:01 | 0.0002 | A*36:01~B*15:10 | 0.0011 |
|  |  |  |  | A*66:01~B*82:02 | 0.0003 |  |  |  |  | A*26:01~B*38:01 | 0.0070 | A*36:01~B*15:16 | 0.0014 |
|  |  |  |  | A*66:02~B*15:03 | 0.0006 |  |  |  |  | A*26:01~B*39:01 | 0.0002 | A*36:01~B*18:01 | 0.0008 |
|  |  |  |  | A*66:02~B*42:01 | 0.0090 |  |  |  |  | A*26:01~B*39:24 | 0.0003 | A*36:01~B*27:05 | 0.0008 |
|  |  |  |  | A*66:02~B*44:03 | 0.0008 |  |  |  |  | A*26:01~B*40:04 | 0.0003 | A*36:01~B*39:10 | 0.0013 |
|  |  |  |  | A*66:03~B*35:01 | 0.0003 |  |  |  |  | A*26:01~B*44:02 | 0.0022 | A*36:01~B*44:03 | 0.0020 |
|  |  |  |  | A*66:03~B*44:03 | 0.0003 |  |  |  |  | A*26:01~B*45:01 | 0.0010 | A*36:01~B*51:01 | 0.0010 |
|  |  |  |  | A*66:03~B*57:03 | 0.0003 |  |  |  |  | A*26:01~B*49:01 | 0.0025 | A*36:01~B*52:01 | 0.0008 |
|  |  |  |  | A*68:01~B*07:02 | 0.0004 |  |  |  |  | A*26:01~B*51:01 | 0.0017 | A*36:01~B*53:01 | 0.0079 |
|  |  |  |  | A*68:01~B*07:05 | 0.0003 |  |  |  |  | A*26:01~B*52:01 | 0.0002 | A*36:01~B*57:03 | 0.0017 |
|  |  |  |  | A*68:01~B*08:01 | 0.0005 |  |  |  |  | A*26:01~B*55:01 | 0.0002 | A*36:01~B*58:02 | 0.0025 |
|  |  |  |  | A*68:01~B*15:03 | 0.0008 |  |  |  |  | A*26:01~B*58:01 | 0.0005 | A*66:01~B*08:01 | 0.0008 |
|  |  |  |  | A*68:01~B*27:05 | 0.0003 |  |  |  |  | A*26:08~B*27:02 | 0.0003 | A*66:01~B*13:02 | 0.0008 |
|  |  |  |  | A*68:01~B*35:01 | 0.0021 |  |  |  |  | A*26:08~B*39:01 | 0.0008 | A*66:01~B*15:03 | 0.0016 |
|  |  |  |  | A*68:01~B*41:01 | 0.0032 |  |  |  |  | A*26:08~B*45:01 | 0.0003 | A*66:01~B*15:10 | 0.0008 |
|  |  |  |  | A*68:01~B*42:01 | 0.0006 |  |  |  |  | A*26:08~B*51:01 | 0.0003 | A*66:01~B*35:01 | 0.0020 |
|  |  |  |  | A*68:01~B*51:01 | 0.0003 |  |  |  |  | A*29:01~B*07:02 | 0.0006 | A*66:01~B*39:10 | 0.0026 |
|  |  |  |  | A*68:01~B*58:01 | 0.0030 |  |  |  |  | A*29:01~B*07:05 | 0.0014 | A*66:01~B*42:01 | 0.0023 |
|  |  |  |  | A*68:01~B*58:02 | 0.0153 |  |  |  |  | A*29:01~B*14:02 | 0.0001 | A*66:01~B*44:03 | 0.0015 |
|  |  |  |  | A*68:01~B*82:02 | 0.0015 |  |  |  |  | A*29:01~B*44:02 | 0.0001 | A*66:01~B*45:01 | 0.0015 |
|  |  |  |  | A*68:02~B*07:02 | 0.0149 |  |  |  |  | A*29:01~B*51:01 | 0.0003 | A*66:01~B*51:07 | 0.0008 |
|  |  |  |  | A*68:02~B*08:01 | 0.0008 |  |  |  |  | A*29:01~B*52:01 | 0.0006 | A*66:01~B*52:01 | 0.0008 |
|  |  |  |  | A*68:02~B*14:01 | 0.0048 |  |  |  |  | A*29:01~B*53:01 | 0.0003 | A*66:01~B*53:01 | 0.0030 |
|  |  |  |  | A*68:02~B*14:02 | 0.0031 |  |  |  |  | A*29:01~B*57:01 | 0.0003 | A*66:01~B*58:02 | 0.0029 |
|  |  |  |  | A*68:02~B*14:06 | 0.0003 |  |  |  |  | A*29:02~B*07:02 | 0.0016 | A*66:02~B*39:01 | 0.0008 |
|  |  |  |  | A*68:02~B*15:03 | 0.0034 |  |  |  |  | A*29:02~B*07:08 | 0.0003 | A*66:02~B*53:01 | 0.0008 |
|  |  |  |  | A*68:02~B*15:10 | 0.0328 |  |  |  |  | A*29:02~B*08:01 | 0.0003 | A*66:02~B*58:01 | 0.0068 |
|  |  |  |  | A*68:02~B*15:16 | 0.0033 |  |  |  |  | A*29:02~B*14:01 | 0.0003 | A*66:03~B*41:02 | 0.0008 |
|  |  |  |  | A*68:02~B*15:24 | 0.0003 |  |  |  |  | A*29:02~B*14:02 | 0.0007 | A*66:03~B*51:01 | 0.0008 |
|  |  |  |  | A*68:02~B*18:01 | 0.0039 |  |  |  |  | A*29:02~B*27:02 | 0.0005 | A*68:01~B*07:02 | 0.0046 |
|  |  |  |  | A*68:02~B*35:02 | 0.0000 |  |  |  |  | A*29:02~B*27:05 | 0.0006 | A*68:01~B*08:01 | 0.0010 |
|  |  |  |  | A*68:02~B*42:01 | 0.0029 |  |  |  |  | A*29:02~B*35:01 | 0.0008 | A*68:01~B*15:01 | 0.0008 |
|  |  |  |  | A*68:02~B*44:03 | 0.0041 |  |  |  |  | A*29:02~B*35:12 | 0.0003 | A*68:01~B*15:03 | 0.0020 |
|  |  |  |  | A*68:02~B*45:01 | 0.0007 |  |  |  |  | A*29:02~B*39:11 | 0.0003 | A*68:01~B*15:17 | 0.0008 |
|  |  |  |  | A*68:02~B*51:01 | 0.0003 |  |  |  |  | A*29:02~B*41:02 | 0.0003 | A*68:01~B*35:01 | 0.0030 |
|  |  |  |  | A*68:02~B*53:01 | 0.0024 |  |  |  |  | A*29:02~B*42:01 | 0.0003 | A*68:01~B*35:03 | 0.0008 |
|  |  |  |  | A*68:02~B*57:02 | 0.0038 |  |  |  |  | A*29:02~B*44:02 | 0.0007 | A*68:01~B*39:10 | 0.0008 |
|  |  |  |  | A*68:02~B*57:03 | 0.0007 |  |  |  |  | A*29:02~B*44:03 | 0.0230 | A*68:01~B*40:02 | 0.0008 |
|  |  |  |  | A*68:02~B*58:01 | 0.0042 |  |  |  |  | A*29:02~B*44:04 | 0.0006 | A*68:01~B*42:01 | 0.0008 |
|  |  |  |  | A*68:02~B*81:01 | 0.0008 |  |  |  |  | A*29:02~B*45:01 | 0.0018 | A*68:01~B*44:03 | 0.0015 |
|  |  |  |  | A*68:04~B*58:02 | 0.0003 |  |  |  |  | A*29:02~B*48:01 | 0.0003 | A*68:01~B*51:02 | 0.0008 |
|  |  |  |  | A*68:27~B*07:02 | 0.0003 |  |  |  |  | A*29:02~B*50:01 | 0.0005 | A*68:01~B*57:02 | 0.0015 |
|  |  |  |  | A*68:27~B*08:01 | 0.0001 |  |  |  |  | A*29:02~B*51:01 | 0.0014 | A*68:01~B*57:03 | 0.0015 |
|  |  |  |  | A*68:27~B*41:02 | 0.0003 |  |  |  |  | A*29:02~B*55:01 | 0.0005 | A*68:01~B*58:02 | 0.0081 |
|  |  |  |  | A*68:27~B*44:03 | 0.0015 |  |  |  |  | A*29:02~B*57:02 | 0.0003 | A*68:02~B*07:02 | 0.0042 |
|  |  |  |  | A*68:27~B*57:03 | 0.0003 |  |  |  |  | A*29:02~B*57:03 | 0.0003 | A*68:02~B*07:05 | 0.0015 |
|  |  |  |  | A*69:02~B*45:07 | 0.0000 |  |  |  |  | A*29:02~B*58:01 | 0.0010 | A*68:02~B*13:02 | 0.0008 |
|  |  |  |  | A*69:02~B*58:02 | 0.0003 |  |  |  |  | A*29:10~B*14:02 | 0.0006 | A*68:02~B*14:01 | 0.0011 |
|  |  |  |  | A*74:00~B*15:03 | 0.0075 |  |  |  |  | A*30:01~B*08:01 | 0.0003 | A*68:02~B*15:10 | 0.0065 |
|  |  |  |  | A*74:00~B*15:10 | 0.0007 |  |  |  |  | A*30:01~B*13:02 | 0.0122 | A*68:02~B*15:16 | 0.0013 |
|  |  |  |  | A*74:00~B*18:01 | 0.0003 |  |  |  |  | A*30:01~B*15:01 | 0.0009 | A*68:02~B*15:220 | 0.0008 |
|  |  |  |  | A*74:00~B*35:01 | 0.0057 |  |  |  |  | A*30:01~B*18:01 | 0.0011 | A*68:02~B*18:01 | 0.0008 |
|  |  |  |  | A*74:00~B*42:01 | 0.0004 |  |  |  |  | A*30:01~B*27:02 | 0.0003 | A*68:02~B*39:01 | 0.0008 |
|  |  |  |  | A*74:00~B*44:03 | 0.0006 |  |  |  |  | A*30:01~B*35:01 | 0.0003 | A*68:02~B*39:10 | 0.0015 |
|  |  |  |  | A*74:00~B*49:01 | 0.0009 |  |  |  |  | A*30:01~B*35:14 | 0.0003 | A*68:02~B*44:03 | 0.0010 |
|  |  |  |  | A*74:00~B*51:01 | 0.0003 |  |  |  |  | A*30:01~B*37:01 | 0.0003 | A*68:02~B*45:01 | 0.0011 |
|  |  |  |  | A*74:00~B*57:03 | 0.0052 |  |  |  |  | A*30:01~B*40:02 | 0.0003 | A*68:02~B*47:01 | 0.0008 |
|  |  |  |  | A*74:00~B*58:01 | 0.0006 |  |  |  |  | A*30:01~B*42:01 | 0.0003 | A*68:02~B*49:01 | 0.0016 |
|  |  |  |  | A*74:00~B*81:00 | 0.0002 |  |  |  |  | A*30:01~B*42:02 | 0.0003 | A*68:02~B*51:01 | 0.0022 |
|  |  |  |  | A*74:01~B*07:02 | 0.0006 |  |  |  |  | A*30:01~B*49:01 | 0.0003 | A*68:02~B*53:01 | 0.0133 |
|  |  |  |  | A*74:01~B*08:01 | 0.0004 |  |  |  |  | A*30:01~B*50:01 | 0.0003 | A*68:02~B*57:03 | 0.0097 |
|  |  |  |  | A*74:01~B*14:01 | 0.0003 |  |  |  |  | A*30:01~B*51:01 | 0.0003 | A*68:02~B*58:01 | 0.0015 |
|  |  |  |  | A*74:01~B*15:03 | 0.0190 |  |  |  |  | A*30:01~B*53:01 | 0.0009 | A*68:02~B*58:02 | 0.0014 |
|  |  |  |  | A*74:01~B*15:10 | 0.0003 |  |  |  |  | A*30:01~B*56:01 | 0.0003 | A*68:02~B*81:01 | 0.0080 |
|  |  |  |  | A*74:01~B*18:01 | 0.0003 |  |  |  |  | A*30:01~B*57:03 | 0.0005 | A*68:10~B*44:02 | 0.0008 |
|  |  |  |  | A*74:01~B*35:01 | 0.0070 |  |  |  |  | A*30:01~B*58:02 | 0.0003 | A*69:01~B*15:01 | 0.0008 |
|  |  |  |  | A*74:01~B*42:01 | 0.0012 |  |  |  |  | A*30:02~B*08:01 | 0.0008 | A*74:00~B*07:05 | 0.0008 |
|  |  |  |  | A*74:01~B*44:03 | 0.0004 |  |  |  |  | A*30:02~B*14:02 | 0.0003 | A*74:00~B*13:02 | 0.0008 |
|  |  |  |  | A*74:01~B*49:01 | 0.0003 |  |  |  |  | A*30:02~B*15:03 | 0.0003 | A*74:00~B*15:03 | 0.0023 |
|  |  |  |  | A*74:01~B*50:01 | 0.0003 |  |  |  |  | A*30:02~B*18:01 | 0.0031 | A*74:00~B*35:02 | 0.0008 |
|  |  |  |  | A*74:01~B*57:03 | 0.0049 |  |  |  |  | A*30:02~B*27:05 | 0.0003 | A*74:00~B*42:01 | 0.0008 |
|  |  |  |  | A*74:01~B*58:01 | 0.0012 |  |  |  |  | A*30:02~B*35:01 | 0.0004 | A*74:00~B*49:01 | 0.0023 |
|  |  |  |  | A*74:01~B*81:01 | 0.0003 |  |  |  |  | A*30:02~B*40:16 | 0.0003 | A*74:00~B*50:01 | 0.0008 |
|  |  |  |  | A*80:01~B*18:01 | 0.0082 |  |  |  |  | A*30:02~B*42:01 | 0.0003 | A*74:00~B*53:01 | 0.0030 |
|  |  |  |  | A*80:01~B*39:10 | 0.0003 |  |  |  |  | A*30:02~B*44:02 | 0.0005 | A*74:00~B*57:02 | 0.0012 |
|  |  |  |  | A*80:01~B*42:01 | 0.0003 |  |  |  |  | A*30:02~B*44:03 | 0.0008 | A*74:00~B*57:03 | 0.0018 |
|  |  |  |  |  |  |  |  |  |  | A*30:02~B*49:01 | 0.0003 | A*74:00~B*78:01 | 0.0008 |
|  |  |  |  |  |  |  |  |  |  | A*30:02~B*53:01 | 0.0014 | A*74:01~B*07:02 | 0.0038 |
|  |  |  |  |  |  |  |  |  |  | A*30:02~B*57:03 | 0.0011 | A*74:01~B*07:05 | 0.0015 |
|  |  |  |  |  |  |  |  |  |  | A*30:02~B*58:01 | 0.0003 | A*74:01~B*13:02 | 0.0008 |
|  |  |  |  |  |  |  |  |  |  | A*30:02~B*81:01 | 0.0003 | A*74:01~B*14:02 | 0.0024 |
|  |  |  |  |  |  |  |  |  |  | A*30:04~B*14:01 | 0.0006 | A*74:01~B*15:03 | 0.0135 |
|  |  |  |  |  |  |  |  |  |  | A*30:04~B*15:17 | 0.0003 | A*74:01~B*15:10 | 0.0015 |
|  |  |  |  |  |  |  |  |  |  | A*30:04~B*27:02 | 0.0003 | A*74:01~B*15:17 | 0.0008 |
|  |  |  |  |  |  |  |  |  |  | A*30:04~B*39:08 | 0.0003 | A*74:01~B*35:01 | 0.0020 |
|  |  |  |  |  |  |  |  |  |  | A*30:04~B*41:01 | 0.0003 | A*74:01~B*35:02 | 0.0008 |
|  |  |  |  |  |  |  |  |  |  | A*30:04~B*44:03 | 0.0006 | A*74:01~B*44:03 | 0.0015 |
|  |  |  |  |  |  |  |  |  |  | A*30:04~B*49:01 | 0.0006 | A*74:01~B*45:01 | 0.0006 |
|  |  |  |  |  |  |  |  |  |  | A*30:04~B*52:01 | 0.0003 | A*74:01~B*49:01 | 0.0025 |
|  |  |  |  |  |  |  |  |  |  | A*30:04~B*58:02 | 0.0003 | A*74:01~B*52:01 | 0.0031 |
|  |  |  |  |  |  |  |  |  |  | A*30:10~B*41:01 | 0.0006 | A*74:01~B*53:01 | 0.0032 |
|  |  |  |  |  |  |  |  |  |  | A*31:01~B*07:02 | 0.0012 | A*74:01~B*55:01 | 0.0008 |
|  |  |  |  |  |  |  |  |  |  | A*31:01~B*08:01 | 0.0011 | A*74:01~B*57:02 | 0.0009 |
|  |  |  |  |  |  |  |  |  |  | A*31:01~B*08:02 | 0.0003 | A*74:01~B*57:03 | 0.0115 |
|  |  |  |  |  |  |  |  |  |  | A*31:01~B*13:02 | 0.0007 | A*74:01~B*58:02 | 0.0001 |
|  |  |  |  |  |  |  |  |  |  | A*31:01~B*14:01 | 0.0006 | A*74:01~B*81:01 | 0.0055 |
|  |  |  |  |  |  |  |  |  |  | A*31:01~B*14:02 | 0.0010 | A*74:01~B*82:01 | 0.0008 |
|  |  |  |  |  |  |  |  |  |  | A*31:01~B*15:01 | 0.0017 | A*74:03~B*41:01 | 0.0008 |
|  |  |  |  |  |  |  |  |  |  | A*31:01~B*15:220 | 0.0003 | A*74:03~B*81:01 | 0.0023 |
|  |  |  |  |  |  |  |  |  |  | A*31:01~B*18:01 | 0.0007 | A*74:03~B*82:02 | 0.0008 |
|  |  |  |  |  |  |  |  |  |  | A*31:01~B*27:05 | 0.0025 | A*74:11~B*15:03 | 0.0008 |
|  |  |  |  |  |  |  |  |  |  | A*31:01~B*35:01 | 0.0015 | A*80:01~B*15:03 | 0.0008 |
|  |  |  |  |  |  |  |  |  |  | A*31:01~B*35:02 | 0.0005 | A*80:01~B*18:01 | 0.0015 |
|  |  |  |  |  |  |  |  |  |  | A*31:01~B*35:03 | 0.0003 | A*80:01~B*35:01 | 0.0015 |
|  |  |  |  |  |  |  |  |  |  | A*31:01~B*35:08 | 0.0007 | A*80:01~B*44:03 | 0.0008 |
|  |  |  |  |  |  |  |  |  |  | A*31:01~B*38:01 | 0.0002 | A*80:01~B*52:01 | 0.0008 |
|  |  |  |  |  |  |  |  |  |  | A*31:01~B*39:01 | 0.0009 | A*80:01~B*57:03 | 0.0008 |
|  |  |  |  |  |  |  |  |  |  | A*31:01~B*39:12 | 0.0003 | A*80:01~B*81:01 | 0.0008 |
|  |  |  |  |  |  |  |  |  |  | A*31:01~B*39:24 | 0.0003 |  |  |
|  |  |  |  |  |  |  |  |  |  | A*31:01~B*40:01 | 0.0075 |  |  |
|  |  |  |  |  |  |  |  |  |  | A*31:01~B*40:02 | 0.0011 |  |  |
|  |  |  |  |  |  |  |  |  |  | A*31:01~B*40:31 | 0.0003 |  |  |
|  |  |  |  |  |  |  |  |  |  | A*31:01~B*41:02 | 0.0006 |  |  |
|  |  |  |  |  |  |  |  |  |  | A*31:01~B*44:02 | 0.0009 |  |  |
|  |  |  |  |  |  |  |  |  |  | A*31:01~B*44:03 | 0.0003 |  |  |
|  |  |  |  |  |  |  |  |  |  | A*31:01~B*44:04 | 0.0003 |  |  |
|  |  |  |  |  |  |  |  |  |  | A*31:01~B*45:01 | 0.0003 |  |  |
|  |  |  |  |  |  |  |  |  |  | A*31:01~B*49:01 | 0.0008 |  |  |
|  |  |  |  |  |  |  |  |  |  | A*31:01~B*51:01 | 0.0044 |  |  |
|  |  |  |  |  |  |  |  |  |  | A*31:01~B*51:07 | 0.0002 |  |  |
|  |  |  |  |  |  |  |  |  |  | A*31:01~B*51:09 | 0.0003 |  |  |
|  |  |  |  |  |  |  |  |  |  | A*31:01~B*53:01 | 0.0003 |  |  |
|  |  |  |  |  |  |  |  |  |  | A*31:01~B*55:01 | 0.0006 |  |  |
|  |  |  |  |  |  |  |  |  |  | A*31:01~B*56:01 | 0.0003 |  |  |
|  |  |  |  |  |  |  |  |  |  | A*31:01~B*57:01 | 0.0027 |  |  |
|  |  |  |  |  |  |  |  |  |  | A*31:02~B*49:01 | 0.0003 |  |  |
|  |  |  |  |  |  |  |  |  |  | A*32:01~B*07:02 | 0.0005 |  |  |
|  |  |  |  |  |  |  |  |  |  | A*32:01~B*07:05 | 0.0006 |  |  |
|  |  |  |  |  |  |  |  |  |  | A*32:01~B*08:01 | 0.0018 |  |  |
|  |  |  |  |  |  |  |  |  |  | A*32:01~B*14:01 | 0.0050 |  |  |
|  |  |  |  |  |  |  |  |  |  | A*32:01~B*14:02 | 0.0013 |  |  |
|  |  |  |  |  |  |  |  |  |  | A*32:01~B*15:01 | 0.0026 |  |  |
|  |  |  |  |  |  |  |  |  |  | A*32:01~B*15:03 | 0.0003 |  |  |
|  |  |  |  |  |  |  |  |  |  | A*32:01~B*15:17 | 0.0003 |  |  |
|  |  |  |  |  |  |  |  |  |  | A*32:01~B*18:01 | 0.0019 |  |  |
|  |  |  |  |  |  |  |  |  |  | A*32:01~B*27:02 | 0.0007 |  |  |
|  |  |  |  |  |  |  |  |  |  | A*32:01~B*27:05 | 0.0042 |  |  |
|  |  |  |  |  |  |  |  |  |  | A*32:01~B*27:09 | 0.0003 |  |  |
|  |  |  |  |  |  |  |  |  |  | A*32:01~B*35:01 | 0.0023 |  |  |
|  |  |  |  |  |  |  |  |  |  | A*32:01~B*35:03 | 0.0006 |  |  |
|  |  |  |  |  |  |  |  |  |  | A*32:01~B*35:32 | 0.0003 |  |  |
|  |  |  |  |  |  |  |  |  |  | A*32:01~B*38:01 | 0.0003 |  |  |
|  |  |  |  |  |  |  |  |  |  | A*32:01~B*39:01 | 0.0002 |  |  |
|  |  |  |  |  |  |  |  |  |  | A*32:01~B*39:06 | 0.0003 |  |  |
|  |  |  |  |  |  |  |  |  |  | A*32:01~B*40:01 | 0.0032 |  |  |
|  |  |  |  |  |  |  |  |  |  | A*32:01~B*40:02 | 0.0051 |  |  |
|  |  |  |  |  |  |  |  |  |  | A*32:01~B*41:01 | 0.0002 |  |  |
|  |  |  |  |  |  |  |  |  |  | A*32:01~B*44:02 | 0.0064 |  |  |
|  |  |  |  |  |  |  |  |  |  | A*32:01~B*44:03 | 0.0005 |  |  |
|  |  |  |  |  |  |  |  |  |  | A*32:01~B*45:01 | 0.0003 |  |  |
|  |  |  |  |  |  |  |  |  |  | A*32:01~B*47:01 | 0.0003 |  |  |
|  |  |  |  |  |  |  |  |  |  | A*32:01~B*50:01 | 0.0003 |  |  |
|  |  |  |  |  |  |  |  |  |  | A*32:01~B*50:02 | 0.0003 |  |  |
|  |  |  |  |  |  |  |  |  |  | A*32:01~B*51:01 | 0.0011 |  |  |
|  |  |  |  |  |  |  |  |  |  | A*32:01~B*51:07 | 0.0003 |  |  |
|  |  |  |  |  |  |  |  |  |  | A*32:01~B*53:01 | 0.0003 |  |  |
|  |  |  |  |  |  |  |  |  |  | A*32:01~B*55:01 | 0.0017 |  |  |
|  |  |  |  |  |  |  |  |  |  | A*32:01~B*57:01 | 0.0027 |  |  |
|  |  |  |  |  |  |  |  |  |  | A*32:01~B*73:01 | 0.0003 |  |  |
|  |  |  |  |  |  |  |  |  |  | A*32:114~B*39:01 | 0.0003 |  |  |
|  |  |  |  |  |  |  |  |  |  | A*33:01~B*14:02 | 0.0079 |  |  |
|  |  |  |  |  |  |  |  |  |  | A*33:01~B*15:03 | 0.0003 |  |  |
|  |  |  |  |  |  |  |  |  |  | A*33:01~B*35:02 | 0.0003 |  |  |
|  |  |  |  |  |  |  |  |  |  | A*33:01~B*44:02 | 0.0003 |  |  |
|  |  |  |  |  |  |  |  |  |  | A*33:01~B*44:03 | 0.0004 |  |  |
|  |  |  |  |  |  |  |  |  |  | A*33:01~B*58:01 | 0.0002 |  |  |
|  |  |  |  |  |  |  |  |  |  | A*33:03~B*07:05 | 0.0002 |  |  |
|  |  |  |  |  |  |  |  |  |  | A*33:03~B*15:16 | 0.0003 |  |  |
|  |  |  |  |  |  |  |  |  |  | A*33:03~B*15:17 | 0.0004 |  |  |
|  |  |  |  |  |  |  |  |  |  | A*33:03~B*27:05 | 0.0005 |  |  |
|  |  |  |  |  |  |  |  |  |  | A*33:03~B*35:02 | 0.0003 |  |  |
|  |  |  |  |  |  |  |  |  |  | A*33:03~B*35:08 | 0.0006 |  |  |
|  |  |  |  |  |  |  |  |  |  | A*33:03~B*39:01 | 0.0003 |  |  |
|  |  |  |  |  |  |  |  |  |  | A*33:03~B*39:05 | 0.0003 |  |  |
|  |  |  |  |  |  |  |  |  |  | A*33:03~B*50:01 | 0.0008 |  |  |
|  |  |  |  |  |  |  |  |  |  | A*33:03~B*57:01 | 0.0006 |  |  |
|  |  |  |  |  |  |  |  |  |  | A*33:03~B*58:01 | 0.0008 |  |  |
|  |  |  |  |  |  |  |  |  |  | A*33:05~B*14:02 | 0.0003 |  |  |
|  |  |  |  |  |  |  |  |  |  | A*34:02~B*08:01 | 0.0014 |  |  |
|  |  |  |  |  |  |  |  |  |  | A*34:02~B*35:03 | 0.0003 |  |  |
|  |  |  |  |  |  |  |  |  |  | A*34:02~B*39:10 | 0.0003 |  |  |
|  |  |  |  |  |  |  |  |  |  | A*34:02~B*53:01 | 0.0003 |  |  |
|  |  |  |  |  |  |  |  |  |  | A*36:01~B*44:02 | 0.0006 |  |  |
|  |  |  |  |  |  |  |  |  |  | A*36:01~B*53:01 | 0.0008 |  |  |
|  |  |  |  |  |  |  |  |  |  | A*66:01~B*07:05 | 0.0003 |  |  |
|  |  |  |  |  |  |  |  |  |  | A*66:01~B*14:02 | 0.0003 |  |  |
|  |  |  |  |  |  |  |  |  |  | A*66:01~B*15:220 | 0.0003 |  |  |
|  |  |  |  |  |  |  |  |  |  | A*66:01~B*27:02 | 0.0003 |  |  |
|  |  |  |  |  |  |  |  |  |  | A*66:01~B*35:02 | 0.0003 |  |  |
|  |  |  |  |  |  |  |  |  |  | A*66:01~B*38:01 | 0.0008 |  |  |
|  |  |  |  |  |  |  |  |  |  | A*66:01~B*41:01 | 0.0003 |  |  |
|  |  |  |  |  |  |  |  |  |  | A*66:01~B*41:02 | 0.0011 |  |  |
|  |  |  |  |  |  |  |  |  |  | A*66:01~B*53:01 | 0.0003 |  |  |
|  |  |  |  |  |  |  |  |  |  | A*68:01~B*07:02 | 0.0018 |  |  |
|  |  |  |  |  |  |  |  |  |  | A*68:01~B*08:01 | 0.0014 |  |  |
|  |  |  |  |  |  |  |  |  |  | A*68:01~B*13:02 | 0.0006 |  |  |
|  |  |  |  |  |  |  |  |  |  | A*68:01~B*14:01 | 0.0005 |  |  |
|  |  |  |  |  |  |  |  |  |  | A*68:01~B*15:01 | 0.0005 |  |  |
|  |  |  |  |  |  |  |  |  |  | A*68:01~B*15:16 | 0.0003 |  |  |
|  |  |  |  |  |  |  |  |  |  | A*68:01~B*15:18 | 0.0005 |  |  |
|  |  |  |  |  |  |  |  |  |  | A*68:01~B*15:31 | 0.0003 |  |  |
|  |  |  |  |  |  |  |  |  |  | A*68:01~B*18:01 | 0.0006 |  |  |
|  |  |  |  |  |  |  |  |  |  | A*68:01~B*27:05 | 0.0019 |  |  |
|  |  |  |  |  |  |  |  |  |  | A*68:01~B*35:01 | 0.0009 |  |  |
|  |  |  |  |  |  |  |  |  |  | A*68:01~B*35:02 | 0.0003 |  |  |
|  |  |  |  |  |  |  |  |  |  | A*68:01~B*35:03 | 0.0022 |  |  |
|  |  |  |  |  |  |  |  |  |  | A*68:01~B*35:05 | 0.0003 |  |  |
|  |  |  |  |  |  |  |  |  |  | A*68:01~B*35:08 | 0.0003 |  |  |
|  |  |  |  |  |  |  |  |  |  | A*68:01~B*38:01 | 0.0011 |  |  |
|  |  |  |  |  |  |  |  |  |  | A*68:01~B*39:01 | 0.0003 |  |  |
|  |  |  |  |  |  |  |  |  |  | A*68:01~B*40:01 | 0.0009 |  |  |
|  |  |  |  |  |  |  |  |  |  | A*68:01~B*40:02 | 0.0003 |  |  |
|  |  |  |  |  |  |  |  |  |  | A*68:01~B*44:02 | 0.0047 |  |  |
|  |  |  |  |  |  |  |  |  |  | A*68:01~B*44:03 | 0.0008 |  |  |
|  |  |  |  |  |  |  |  |  |  | A*68:01~B*47:01 | 0.0005 |  |  |
|  |  |  |  |  |  |  |  |  |  | A*68:01~B*51:01 | 0.0038 |  |  |
|  |  |  |  |  |  |  |  |  |  | A*68:01~B*52:01 | 0.0012 |  |  |
|  |  |  |  |  |  |  |  |  |  | A*68:01~B*55:01 | 0.0003 |  |  |
|  |  |  |  |  |  |  |  |  |  | A*68:01~B*57:01 | 0.0012 |  |  |
|  |  |  |  |  |  |  |  |  |  | A*68:01~B*57:03 | 0.0002 |  |  |
|  |  |  |  |  |  |  |  |  |  | A*68:02~B*14:02 | 0.0056 |  |  |
|  |  |  |  |  |  |  |  |  |  | A*68:02~B*14:06 | 0.0003 |  |  |
|  |  |  |  |  |  |  |  |  |  | A*68:02~B*15:10 | 0.0004 |  |  |
|  |  |  |  |  |  |  |  |  |  | A*68:02~B*18:01 | 0.0003 |  |  |
|  |  |  |  |  |  |  |  |  |  | A*68:02~B*27:05 | 0.0004 |  |  |
|  |  |  |  |  |  |  |  |  |  | A*68:02~B*38:01 | 0.0003 |  |  |
|  |  |  |  |  |  |  |  |  |  | A*68:02~B*40:02 | 0.0002 |  |  |
|  |  |  |  |  |  |  |  |  |  | A*68:02~B*41:01 | 0.0003 |  |  |
|  |  |  |  |  |  |  |  |  |  | A*68:02~B*49:01 | 0.0003 |  |  |
|  |  |  |  |  |  |  |  |  |  | A*68:02~B*53:01 | 0.0030 |  |  |
|  |  |  |  |  |  |  |  |  |  | A*68:02~B*57:01 | 0.0003 |  |  |
|  |  |  |  |  |  |  |  |  |  | A*68:02~B*57:03 | 0.0031 |  |  |
|  |  |  |  |  |  |  |  |  |  | A*68:03~B*35:43 | 0.0003 |  |  |
|  |  |  |  |  |  |  |  |  |  | A*68:03~B*44:03 | 0.0003 |  |  |
|  |  |  |  |  |  |  |  |  |  | A*68:03~B*44:05 | 0.0003 |  |  |
|  |  |  |  |  |  |  |  |  |  | A*68:07~B*39:08 | 0.0003 |  |  |
|  |  |  |  |  |  |  |  |  |  | A*68:07~B*40:02 | 0.0003 |  |  |
|  |  |  |  |  |  |  |  |  |  | A*68:15~B*57:03 | 0.0003 |  |  |
|  |  |  |  |  |  |  |  |  |  | A*69:01~B*07:05 | 0.0003 |  |  |
|  |  |  |  |  |  |  |  |  |  | A*69:01~B*39:01 | 0.0003 |  |  |
|  |  |  |  |  |  |  |  |  |  | A*69:01~B*44:02 | 0.0003 |  |  |
|  |  |  |  |  |  |  |  |  |  | A*69:01~B*52:01 | 0.0006 |  |  |
|  |  |  |  |  |  |  |  |  |  | A*69:01~B*55:01 | 0.0011 |  |  |
|  |  |  |  |  |  |  |  |  |  | A*69:01~B*57:03 | 0.0003 |  |  |
|  |  |  |  |  |  |  |  |  |  | A*74:00~B*15:03 | 0.0003 |  |  |
|  |  |  |  |  |  |  |  |  |  | A*74:00~B*15:10 | 0.0003 |  |  |
|  |  |  |  |  |  |  |  |  |  | A*74:01~B*18:01 | 0.0003 |  |  |
|  |  |  |  |  |  |  |  |  |  | A*74:01~B*35:01 | 0.0003 |  |  |
|  |  |  |  |  |  |  |  |  |  | A*74:03~B*51:01 | 0.0003 |  |  |
|  |  |  |  |  |  |  |  |  |  | A*80:01~B*44:02 | 0.0003 |  |  |

**Supplementary Table 5|** full list of haplotype A~C for all populations

| **KENYA** | | **RWANDA** | | **SOUTH AFRICA** | | **UGANDA** | | **ZAMBIA** | | **EUAM** | | **AFAM** | |
| --- | --- | --- | --- | --- | --- | --- | --- | --- | --- | --- | --- | --- | --- |
| A~C | HF | A~C | HF | A~C | HF | A~C | HF | A~C | HF | A~C | HF | A~C | HF |
| A*01:01~C*04:07 | 0.0046 | A*01:01~C*03:02 | 0.0029 | A*01:01~C*01:02 | 0.0003 | A*01:01~C*04:01 | 0.0025 | A*01:01~C*03:04 | 0.0027 | A*01:01~C*01:02 | 0.0023 | A*01:01~C*01:02 | 0.0010 |
| A*01:01~C*06:02 | 0.0092 | A*01:01~C*04:01 | 0.0049 | A*01:01~C*04:01 | 0.0010 | A*01:01~C*04:07 | 0.0346 | A*01:01~C*04:01 | 0.0008 | A*01:01~C*02:02 | 0.0022 | A*01:01~C*02:02 | 0.0008 |
| A*01:01~C*07:01 | 0.0229 | A*01:01~C*04:07 | 0.0257 | A*01:01~C*06:02 | 0.0025 | A*01:01~C*06:02 | 0.0099 | A*01:01~C*06:02 | 0.0019 | A*01:01~C*03:02 | 0.0007 | A*01:01~C*03:04 | 0.0024 |
| A*01:01~C*07:04 | 0.0092 | A*01:01~C*06:02 | 0.0439 | A*01:01~C*07:01 | 0.0016 | A*01:01~C*07:01 | 0.0038 | A*01:01~C*08:02 | 0.0009 | A*01:01~C*03:03 | 0.0012 | A*01:01~C*06:02 | 0.0082 |
| A*01:01~C*14:02 | 0.0046 | A*01:01~C*07:01 | 0.0138 | A*01:01~C*07:02 | 0.0006 | A*01:01~C*07:02 | 0.0022 | A*01:01~C*12:03 | 0.0009 | A*01:01~C*03:04 | 0.0029 | A*01:01~C*07:01 | 0.0177 |
| A*01:01~C*18:01 | 0.0138 | A*01:01~C*15:02 | 0.0087 | A*01:01~C*08:04 | 0.0003 | A*01:01~C*08:02 | 0.0032 | A*01:01~C*17:01 | 0.0009 | A*01:01~C*04:01 | 0.0053 | A*01:01~C*07:06 | 0.0008 |
| A*01:03~C*07:01 | 0.0046 | A*01:01~C*18:01 | 0.0157 | A*01:01~C*16:01 | 0.0005 | A*01:01~C*15:02 | 0.0022 | A*01:01~C*18:01 | 0.0079 | A*01:01~C*05:01 | 0.0005 | A*01:01~C*12:02 | 0.0011 |
| A*01:03~C*17:01 | 0.0046 | A*01:02~C*03:02 | 0.0029 | A*01:01~C*18:00 | 0.0198 | A*01:01~C*17:01 | 0.0022 | A*01:02~C*06:02 | 0.0009 | A*01:01~C*06:02 | 0.0426 | A*01:01~C*12:03 | 0.0035 |
| A*02:01~C*02:10 | 0.0279 | A*01:03~C*07:01 | 0.0050 | A*01:01~C*18:01 | 0.0052 | A*01:01~C*18:01 | 0.0108 | A*01:03~C*14:02 | 0.0009 | A*01:01~C*07:01 | 0.0587 | A*01:01~C*15:02 | 0.0023 |
| A*02:01~C*03:02 | 0.0092 | A*01:03~C*07:02 | 0.0037 | A*01:02~C*18:00 | 0.0003 | A*01:02~C*03:02 | 0.0043 | A*01:03~C*16:01 | 0.0009 | A*01:01~C*07:02 | 0.0079 | A*01:01~C*16:02 | 0.0008 |
| A*02:01~C*04:01 | 0.0166 | A*01:03~C*16:01 | 0.0000 | A*01:23~C*06:02 | 0.0024 | A*01:02~C*04:01 | 0.0043 | A*01:03~C*18:01 | 0.0009 | A*01:01~C*08:02 | 0.0012 | A*01:01~C*18:01 | 0.0024 |
| A*02:01~C*07:01 | 0.0138 | A*01:09~C*17:01 | 0.0029 | A*02:01~C*02:02 | 0.0003 | A*01:02~C*07:01 | 0.0022 | A*02:01~C*02:02 | 0.0009 | A*01:01~C*12:02 | 0.0053 | A*01:02~C*03:02 | 0.0008 |
| A*02:01~C*07:04 | 0.0134 | A*02:01~C*02:10 | 0.0477 | A*02:01~C*02:10 | 0.0037 | A*01:03~C*18:01 | 0.0043 | A*02:01~C*02:10 | 0.0242 | A*01:01~C*12:03 | 0.0011 | A*01:02~C*07:01 | 0.0023 |
| A*02:01~C*08:02 | 0.0138 | A*02:01~C*03:04 | 0.0029 | A*02:01~C*03:02 | 0.0035 | A*02:01~C*02:10 | 0.0388 | A*02:01~C*03:02 | 0.0027 | A*01:01~C*14:02 | 0.0009 | A*01:02~C*15:05 | 0.0008 |
| A*02:01~C*16:01 | 0.0247 | A*02:01~C*04:01 | 0.0270 | A*02:01~C*03:04 | 0.0008 | A*02:01~C*03:02 | 0.0092 | A*02:01~C*03:03 | 0.0009 | A*01:01~C*15:02 | 0.0001 | A*01:03~C*17:01 | 0.0008 |
| A*02:01~C*16:02 | 0.0046 | A*02:01~C*06:02 | 0.0127 | A*02:01~C*04:01 | 0.0044 | A*02:01~C*04:01 | 0.0194 | A*02:01~C*04:01 | 0.0042 | A*01:01~C*15:05 | 0.0003 | A*01:09~C*04:07 | 0.0008 |
| A*02:02~C*02:10 | 0.0092 | A*02:01~C*07:01 | 0.0128 | A*02:01~C*06:02 | 0.0034 | A*02:01~C*07:01 | 0.0238 | A*02:01~C*06:02 | 0.0049 | A*01:01~C*15:13 | 0.0004 | A*01:09~C*18:00 | 0.0008 |
| A*02:02~C*04:01 | 0.0046 | A*02:01~C*07:02 | 0.0092 | A*02:01~C*07:01 | 0.0063 | A*02:01~C*08:02 | 0.0061 | A*02:01~C*07:01 | 0.0061 | A*01:01~C*16:01 | 0.0010 | A*01:22N~C*12:02 | 0.0008 |
| A*02:02~C*06:02 | 0.0092 | A*02:01~C*08:02 | 0.0092 | A*02:01~C*07:04 | 0.0014 | A*02:01~C*16:01 | 0.0368 | A*02:01~C*07:02 | 0.0019 | A*01:01~C*16:02 | 0.0012 | A*02:01~C*01:02 | 0.0051 |
| A*02:02~C*07:01 | 0.0046 | A*02:01~C*14:02 | 0.0029 | A*02:01~C*08:01 | 0.0003 | A*02:01~C*17:01 | 0.0022 | A*02:01~C*08:02 | 0.0022 | A*01:01~C*16:04 | 0.0003 | A*02:01~C*02:02 | 0.0054 |
| A*02:02~C*17:01 | 0.0183 | A*02:01~C*15:02 | 0.0029 | A*02:01~C*08:02 | 0.0004 | A*02:02~C*03:04 | 0.0033 | A*02:01~C*08:04 | 0.0018 | A*01:01~C*17:01 | 0.0010 | A*02:01~C*02:10 | 0.0043 |
| A*02:05~C*02:02 | 0.0046 | A*02:01~C*16:01 | 0.0086 | A*02:01~C*08:04 | 0.0025 | A*02:02~C*06:02 | 0.0335 | A*02:01~C*15:25 | 0.0009 | A*01:02~C*03:04 | 0.0003 | A*02:01~C*03:02 | 0.0018 |
| A*02:05~C*04:01 | 0.0046 | A*02:01~C*16:02 | 0.0029 | A*02:01~C*12:03 | 0.0004 | A*02:02~C*07:01 | 0.0022 | A*02:01~C*16:01 | 0.0305 | A*01:02~C*16:02 | 0.0003 | A*02:01~C*03:03 | 0.0015 |
| A*02:05~C*07:01 | 0.0229 | A*02:02~C*02:10 | 0.0050 | A*02:01~C*15:05 | 0.0004 | A*02:02~C*07:02 | 0.0022 | A*02:01~C*17:01 | 0.0062 | A*01:03~C*15:05 | 0.0003 | A*02:01~C*03:04 | 0.0040 |
| A*02:05~C*16:01 | 0.0138 | A*02:02~C*06:02 | 0.0571 | A*02:01~C*16:01 | 0.0196 | A*02:02~C*16:01 | 0.0043 | A*02:01~C*18:01 | 0.0011 | A*01:03~C*18:02 | 0.0003 | A*02:01~C*04:01 | 0.0100 |
| A*02:14~C*15:02 | 0.0046 | A*02:02~C*07:02 | 0.0034 | A*02:01~C*17:00 | 0.0007 | A*02:04~C*04:01 | 0.0022 | A*02:01~C*18:02 | 0.0028 | A*02:01~C*01:02 | 0.0155 | A*02:01~C*05:01 | 0.0037 |
| A*03:01~C*02:10 | 0.0046 | A*02:02~C*08:02 | 0.0029 | A*02:01~C*17:01 | 0.0020 | A*02:05~C*07:01 | 0.0043 | A*02:02~C*02:10 | 0.0019 | A*02:01~C*02:02 | 0.0152 | A*02:01~C*06:02 | 0.0106 |
| A*03:01~C*07:01 | 0.0183 | A*02:02~C*14:02 | 0.0058 | A*02:01~C*18:00 | 0.0009 | A*02:14~C*04:07 | 0.0022 | A*02:02~C*04:01 | 0.0104 | A*02:01~C*02:29 | 0.0003 | A*02:01~C*07:01 | 0.0071 |
| A*11:01~C*04:01 | 0.0046 | A*02:02~C*16:01 | 0.0054 | A*02:02~C*02:10 | 0.0014 | A*02:14~C*08:02 | 0.0022 | A*02:02~C*06:02 | 0.0084 | A*02:01~C*03:02 | 0.0003 | A*02:01~C*07:02 | 0.0072 |
| A*11:01~C*07:01 | 0.0046 | A*02:02~C*17:01 | 0.0015 | A*02:02~C*03:02 | 0.0003 | A*02:14~C*15:02 | 0.0022 | A*02:02~C*07:01 | 0.0011 | A*02:01~C*03:03 | 0.0106 | A*02:01~C*07:04 | 0.0015 |
| A*23:01~C*03:04 | 0.0046 | A*02:05~C*02:02 | 0.0058 | A*02:02~C*04:01 | 0.0007 | A*03:01~C*03:04 | 0.0049 | A*02:02~C*07:02 | 0.0010 | A*02:01~C*03:04 | 0.0295 | A*02:01~C*08:01 | 0.0008 |
| A*23:01~C*04:01 | 0.0046 | A*02:05~C*06:02 | 0.0029 | A*02:02~C*05:01 | 0.0024 | A*03:01~C*04:01 | 0.0153 | A*02:02~C*08:02 | 0.0040 | A*02:01~C*04:01 | 0.0132 | A*02:01~C*08:02 | 0.0019 |
| A*23:01~C*05:01 | 0.0046 | A*02:05~C*07:01 | 0.0033 | A*02:02~C*06:02 | 0.0003 | A*03:01~C*06:02 | 0.0116 | A*02:02~C*14:02 | 0.0018 | A*02:01~C*04:07 | 0.0003 | A*02:01~C*08:04 | 0.0013 |
| A*23:01~C*06:02 | 0.0046 | A*02:05~C*07:02 | 0.0025 | A*02:02~C*07:01 | 0.0038 | A*03:01~C*07:01 | 0.0158 | A*02:02~C*14:03 | 0.0009 | A*02:01~C*05:01 | 0.0423 | A*02:01~C*12:03 | 0.0054 |
| A*23:01~C*07:01 | 0.0046 | A*02:14~C*04:01 | 0.0058 | A*02:02~C*08:04 | 0.0012 | A*03:01~C*07:04 | 0.0065 | A*02:02~C*16:01 | 0.0057 | A*02:01~C*06:02 | 0.0300 | A*02:01~C*14:02 | 0.0032 |
| A*23:01~C*08:02 | 0.0046 | A*03:01~C*06:02 | 0.0329 | A*02:02~C*14:02 | 0.0034 | A*03:01~C*08:02 | 0.0022 | A*02:02~C*17:01 | 0.0020 | A*02:01~C*07:01 | 0.0188 | A*02:01~C*15:05 | 0.0012 |
| A*23:01~C*16:04 | 0.0046 | A*03:01~C*07:01 | 0.0169 | A*02:02~C*17:01 | 0.0003 | A*23:01~C*02:10 | 0.0023 | A*02:02~C*18:02 | 0.0009 | A*02:01~C*07:02 | 0.0269 | A*02:01~C*16:01 | 0.0234 |
| A*23:02~C*08:02 | 0.0046 | A*03:01~C*08:02 | 0.0109 | A*02:05~C*02:02 | 0.0006 | A*23:01~C*03:02 | 0.0065 | A*02:04~C*02:10 | 0.0009 | A*02:01~C*07:04 | 0.0019 | A*02:01~C*17:01 | 0.0055 |
| A*24:02~C*07:01 | 0.0046 | A*03:01~C*18:01 | 0.0029 | A*02:05~C*02:10 | 0.0006 | A*23:01~C*03:04 | 0.0043 | A*02:05~C*02:02 | 0.0020 | A*02:01~C*08:02 | 0.0095 | A*02:01~C*18:00 | 0.0022 |
| A*24:02~C*07:02 | 0.0092 | A*23:01~C*02:10 | 0.0033 | A*02:05~C*02:17 | 0.0024 | A*23:01~C*04:01 | 0.0142 | A*02:05~C*02:10 | 0.0007 | A*02:01~C*08:03 | 0.0003 | A*02:01~C*18:02 | 0.0009 |
| A*26:01~C*02:10 | 0.0092 | A*23:01~C*03:04 | 0.0141 | A*02:05~C*03:04 | 0.0010 | A*23:01~C*05:01 | 0.0022 | A*02:05~C*03:02 | 0.0009 | A*02:01~C*12:02 | 0.0037 | A*02:02~C*02:10 | 0.0010 |
| A*26:01~C*04:01 | 0.0092 | A*23:01~C*04:01 | 0.0084 | A*02:05~C*04:01 | 0.0013 | A*23:01~C*06:02 | 0.0221 | A*02:05~C*04:01 | 0.0026 | A*02:01~C*12:03 | 0.0112 | A*02:02~C*03:02 | 0.0023 |
| A*26:01~C*04:07 | 0.0046 | A*23:01~C*07:01 | 0.0291 | A*02:05~C*06:02 | 0.0067 | A*23:01~C*07:01 | 0.0025 | A*02:05~C*07:01 | 0.0080 | A*02:01~C*14:02 | 0.0075 | A*02:02~C*03:04 | 0.0009 |
| A*29:02~C*04:01 | 0.0046 | A*23:01~C*07:02 | 0.0033 | A*02:05~C*07:01 | 0.0191 | A*23:01~C*07:02 | 0.0130 | A*02:05~C*07:02 | 0.0018 | A*02:01~C*15:02 | 0.0064 | A*02:02~C*04:01 | 0.0078 |
| A*29:02~C*06:02 | 0.0275 | A*23:01~C*07:04 | 0.0029 | A*02:05~C*07:02 | 0.0009 | A*23:01~C*16:02 | 0.0022 | A*02:05~C*08:02 | 0.0015 | A*02:01~C*15:06 | 0.0003 | A*02:02~C*05:01 | 0.0036 |
| A*29:02~C*07:01 | 0.0046 | A*23:01~C*08:02 | 0.0054 | A*02:05~C*07:18 | 0.0006 | A*23:01~C*17:01 | 0.0023 | A*02:05~C*16:01 | 0.0012 | A*02:01~C*15:13 | 0.0001 | A*02:02~C*06:02 | 0.0048 |
| A*29:02~C*17:01 | 0.0138 | A*23:01~C*18:01 | 0.0173 | A*02:05~C*08:02 | 0.0003 | A*26:01~C*03:02 | 0.0022 | A*02:14~C*04:01 | 0.0009 | A*02:01~C*16:01 | 0.0059 | A*02:02~C*07:01 | 0.0018 |
| A*30:01~C*03:04 | 0.0046 | A*24:02~C*03:04 | 0.0058 | A*02:05~C*08:04 | 0.0135 | A*26:01~C*03:04 | 0.0022 | A*02:14~C*07:02 | 0.0009 | A*02:01~C*16:02 | 0.0011 | A*02:02~C*07:05 | 0.0008 |
| A*30:01~C*04:07 | 0.0046 | A*24:02~C*04:01 | 0.0029 | A*02:05~C*16:01 | 0.0011 | A*26:01~C*06:02 | 0.0050 | A*03new~C*03:03 | 0.0009 | A*02:01~C*16:04 | 0.0008 | A*02:02~C*08:02 | 0.0032 |
| A*30:01~C*08:02 | 0.0048 | A*24:02~C*07:01 | 0.0058 | A*02:05~C*17:00 | 0.0010 | A*26:01~C*07:01 | 0.0080 | A*03:01~C*02:02 | 0.0018 | A*02:01~C*17:00 | 0.0009 | A*02:02~C*08:04 | 0.0016 |
| A*30:01~C*17:01 | 0.0496 | A*26:01~C*06:02 | 0.0029 | A*02:05~C*17:01 | 0.0017 | A*29:01~C*18:01 | 0.0022 | A*03:01~C*02:10 | 0.0092 | A*02:01~C*17:01 | 0.0006 | A*02:02~C*14:02 | 0.0021 |
| A*30:01~C*18:01 | 0.0051 | A*26:01~C*07:04 | 0.0029 | A*02:05~C*18:00 | 0.0004 | A*29:02~C*02:02 | 0.0022 | A*03:01~C*03:02 | 0.0017 | A*02:01~C*17:03 | 0.0003 | A*02:02~C*16:01 | 0.0040 |
| A*30:02~C*02:10 | 0.0088 | A*26:03~C*01:02 | 0.0029 | A*02:11~C*07:04 | 0.0003 | A*29:02~C*04:01 | 0.0047 | A*03:01~C*03:04 | 0.0045 | A*02:01~C*18:00 | 0.0003 | A*02:02~C*17:01 | 0.0015 |
| A*30:02~C*03:02 | 0.0046 | A*26:12~C*06:02 | 0.0087 | A*02:11~C*15:02 | 0.0003 | A*29:02~C*06:02 | 0.0062 | A*03:01~C*04:01 | 0.0010 | A*02:02~C*01:02 | 0.0003 | A*02:02~C*18:01 | 0.0009 |
| A*30:02~C*04:01 | 0.0110 | A*29:02~C*04:01 | 0.0055 | A*02:14~C*04:01 | 0.0027 | A*29:02~C*07:01 | 0.0024 | A*03:01~C*06:02 | 0.0181 | A*02:02~C*02:02 | 0.0003 | A*02:04~C*06:02 | 0.0008 |
| A*30:02~C*06:02 | 0.0046 | A*29:02~C*06:02 | 0.0092 | A*02:14~C*06:02 | 0.0003 | A*29:02~C*07:02 | 0.0087 | A*03:01~C*06:03 | 0.0009 | A*02:02~C*07:01 | 0.0006 | A*02:04~C*15:02 | 0.0015 |
| A*30:02~C*07:04 | 0.0004 | A*29:02~C*07:02 | 0.0027 | A*02:14~C*08:04 | 0.0003 | A*29:02~C*07:04 | 0.0022 | A*03:01~C*07:01 | 0.0015 | A*02:02~C*17:00 | 0.0003 | A*02:05~C*02:02 | 0.0009 |
| A*30:02~C*08:02 | 0.0089 | A*29:02~C*08:02 | 0.0029 | A*03:01~C*02:10 | 0.0032 | A*29:02~C*08:04 | 0.0022 | A*03:01~C*08:02 | 0.0014 | A*02:02~C*17:01 | 0.0003 | A*02:05~C*04:01 | 0.0034 |
| A*30:02~C*12:02 | 0.0046 | A*29:02~C*15:02 | 0.0029 | A*03:01~C*03:02 | 0.0006 | A*29:02~C*14:02 | 0.0022 | A*03:01~C*14:03 | 0.0009 | A*02:04~C*03:04 | 0.0003 | A*02:05~C*07:01 | 0.0083 |
| A*30:02~C*12:03 | 0.0046 | A*29:02~C*16:01 | 0.0029 | A*03:01~C*03:04 | 0.0040 | A*29:02~C*15:02 | 0.0022 | A*03:01~C*16:01 | 0.0026 | A*02:04~C*15:02 | 0.0014 | A*02:05~C*08:02 | 0.0008 |
| A*30:02~C*16:01 | 0.0257 | A*29:02~C*17:01 | 0.0058 | A*03:01~C*04:01 | 0.0074 | A*29:02~C*16:01 | 0.0022 | A*03:01~C*18:02 | 0.0025 | A*02:05~C*01:02 | 0.0007 | A*02:05~C*16:01 | 0.0008 |
| A*30:02~C*17:01 | 0.0054 | A*30:01~C*02:02 | 0.0029 | A*03:01~C*06:02 | 0.0219 | A*29:02~C*17:01 | 0.0017 | A*23:01~C*02:10 | 0.0153 | A*02:05~C*03:09 | 0.0003 | A*02:05~C*18:00 | 0.0017 |
| A*30:02~C*18:01 | 0.0040 | A*30:01~C*02:10 | 0.0054 | A*03:01~C*07:01 | 0.0045 | A*29:02~C*18:01 | 0.0022 | A*23:01~C*03:03 | 0.0062 | A*02:05~C*04:01 | 0.0003 | A*02:05~C*18:02 | 0.0015 |
| A*30:04~C*06:02 | 0.0092 | A*30:01~C*03:04 | 0.0032 | A*03:01~C*07:02 | 0.0125 | A*30:01~C*03:04 | 0.0060 | A*23:01~C*03:04 | 0.0017 | A*02:05~C*06:02 | 0.0019 | A*02:06~C*02:02 | 0.0023 |
| A*30:04~C*07:01 | 0.0092 | A*30:01~C*05:01 | 0.0029 | A*03:01~C*07:04 | 0.0006 | A*30:01~C*04:01 | 0.0097 | A*23:01~C*04:01 | 0.0118 | A*02:05~C*07:01 | 0.0042 | A*02:06~C*03:03 | 0.0008 |
| A*30:04~C*18:01 | 0.0092 | A*30:01~C*06:02 | 0.0029 | A*03:01~C*08:04 | 0.0005 | A*30:01~C*06:02 | 0.0156 | A*23:01~C*04:07 | 0.0018 | A*02:05~C*08:02 | 0.0008 | A*02:06~C*03:04 | 0.0008 |
| A*31:04~C*07:02 | 0.0046 | A*30:01~C*08:02 | 0.0029 | A*03:01~C*12:03 | 0.0010 | A*30:01~C*07:02 | 0.0022 | A*23:01~C*06:02 | 0.0227 | A*02:05~C*15:05 | 0.0003 | A*02:11~C*15:02 | 0.0008 |
| A*33:01~C*08:02 | 0.0046 | A*30:01~C*12:03 | 0.0058 | A*03:01~C*16:01 | 0.0007 | A*30:01~C*07:04 | 0.0043 | A*23:01~C*07:01 | 0.0163 | A*02:05~C*16:01 | 0.0004 | A*02:16~C*15:02 | 0.0008 |
| A*33:01~C*14:02 | 0.0046 | A*30:01~C*17:01 | 0.0202 | A*03:01~C*17:00 | 0.0016 | A*30:01~C*08:02 | 0.0037 | A*23:01~C*07:02 | 0.0047 | A*02:05~C*16:02 | 0.0003 | A*03:01~C*01:02 | 0.0008 |
| A*33:01~C*17:01 | 0.0046 | A*30:02~C*02:10 | 0.0116 | A*03:01~C*17:01 | 0.0007 | A*30:01~C*17:01 | 0.0343 | A*23:01~C*07:04 | 0.0010 | A*02:05~C*17:03 | 0.0003 | A*03:01~C*02:02 | 0.0025 |
| A*33:03~C*08:02 | 0.0046 | A*30:02~C*04:01 | 0.0069 | A*03:01~C*18:01 | 0.0006 | A*30:02~C*02:10 | 0.0052 | A*23:01~C*08:02 | 0.0143 | A*02:05~C*18:00 | 0.0003 | A*03:01~C*02:10 | 0.0022 |
| A*34:02~C*02:10 | 0.0046 | A*30:02~C*06:02 | 0.0099 | A*03:01~C*18:02 | 0.0004 | A*30:02~C*03:04 | 0.0108 | A*23:01~C*08:04 | 0.0012 | A*02:06~C*02:02 | 0.0001 | A*03:01~C*03:02 | 0.0008 |
| A*34:02~C*04:01 | 0.0046 | A*30:02~C*07:01 | 0.0186 | A*03:02~C*07:02 | 0.0003 | A*30:02~C*04:01 | 0.0100 | A*23:01~C*16:01 | 0.0091 | A*02:06~C*03:03 | 0.0016 | A*03:01~C*03:03 | 0.0015 |
| A*34:02~C*07:01 | 0.0092 | A*30:02~C*07:02 | 0.0034 | A*03:21~C*17:01 | 0.0003 | A*30:02~C*07:01 | 0.0110 | A*23:01~C*17:01 | 0.0054 | A*02:06~C*03:04 | 0.0007 | A*03:01~C*03:04 | 0.0049 |
| A*36:01~C*04:01 | 0.0275 | A*30:02~C*07:04 | 0.0058 | A*11:01~C*04:01 | 0.0003 | A*30:02~C*07:04 | 0.0105 | A*23:01~C*18:01 | 0.0028 | A*02:06~C*03:05 | 0.0003 | A*03:01~C*04:01 | 0.0115 |
| A*66:01~C*06:02 | 0.0229 | A*30:02~C*12:03 | 0.0029 | A*11:01~C*04:03 | 0.0003 | A*30:02~C*08:02 | 0.0088 | A*23:02~C*12:03 | 0.0009 | A*02:06~C*05:01 | 0.0003 | A*03:01~C*05:01 | 0.0037 |
| A*68:01~C*04:01 | 0.0057 | A*30:02~C*14:02 | 0.0029 | A*11:01~C*05:01 | 0.0003 | A*30:02~C*12:03 | 0.0065 | A*23:02~C*17:01 | 0.0009 | A*02:06~C*06:02 | 0.0005 | A*03:01~C*06:02 | 0.0069 |
| A*68:01~C*06:02 | 0.0092 | A*30:02~C*16:01 | 0.0029 | A*11:01~C*12:02 | 0.0003 | A*30:02~C*16:01 | 0.0065 | A*24:02~C*01:02 | 0.0009 | A*02:06~C*07:02 | 0.0003 | A*03:01~C*07:01 | 0.0222 |
| A*68:01~C*07:01 | 0.0046 | A*30:02~C*17:01 | 0.0058 | A*23new~C*03:03 | 0.0003 | A*30:02~C*17:01 | 0.0022 | A*24:02~C*02:02 | 0.0009 | A*02:06~C*08:01 | 0.0006 | A*03:01~C*07:02 | 0.0079 |
| A*68:01~C*07:02 | 0.0183 | A*30:02~C*18:01 | 0.0045 | A*23:01~C*02:02 | 0.0006 | A*30:02~C*18:01 | 0.0043 | A*24:02~C*04:01 | 0.0037 | A*02:06~C*08:03 | 0.0003 | A*03:01~C*08:02 | 0.0062 |
| A*68:01~C*08:02 | 0.0081 | A*30:04~C*07:01 | 0.0029 | A*23:01~C*02:10 | 0.0119 | A*30:04~C*04:01 | 0.0022 | A*24:02~C*06:02 | 0.0018 | A*02:06~C*12:03 | 0.0003 | A*03:01~C*12:02 | 0.0012 |
| A*68:01~C*18:01 | 0.0046 | A*30:04~C*07:02 | 0.0029 | A*23:01~C*02:17 | 0.0015 | A*30:04~C*06:02 | 0.0022 | A*24:02~C*07:01 | 0.0016 | A*02:06~C*15:02 | 0.0005 | A*03:01~C*12:03 | 0.0053 |
| A*68:02~C*02:02 | 0.0046 | A*30:04~C*14:03 | 0.0029 | A*23:01~C*03:02 | 0.0029 | A*30:04~C*08:02 | 0.0022 | A*24:02~C*16:01 | 0.0018 | A*02:09~C*03:02 | 0.0003 | A*03:01~C*14:02 | 0.0031 |
| A*68:02~C*02:10 | 0.0000 | A*30:09~C*17:01 | 0.0029 | A*23:01~C*03:03 | 0.0049 | A*30:04~C*17:01 | 0.0022 | A*24:31~C*03:04 | 0.0009 | A*02:09~C*04:01 | 0.0003 | A*03:01~C*15:02 | 0.0012 |
| A*68:02~C*03:04 | 0.0596 | A*31:01~C*04:01 | 0.0029 | A*23:01~C*03:04 | 0.0084 | A*30:09~C*04:01 | 0.0065 | A*26:01~C*04:01 | 0.0009 | A*02:11~C*04:01 | 0.0003 | A*03:01~C*15:05 | 0.0013 |
| A*68:02~C*04:01 | 0.0092 | A*32:01~C*04:01 | 0.0029 | A*23:01~C*04:01 | 0.0065 | A*30:09~C*04:07 | 0.0022 | A*26:01~C*07:02 | 0.0018 | A*02:11~C*12:02 | 0.0005 | A*03:01~C*16:01 | 0.0026 |
| A*68:02~C*06:02 | 0.0183 | A*33:01~C*04:01 | 0.0029 | A*23:01~C*06:02 | 0.0129 | A*31:01~C*17:01 | 0.0022 | A*26:01~C*17:01 | 0.0035 | A*02:11~C*15:02 | 0.0011 | A*03:01~C*17:01 | 0.0016 |
| A*68:02~C*07:02 | 0.0413 | A*33:03~C*04:07 | 0.0058 | A*23:01~C*07:01 | 0.0125 | A*31:03~C*08:02 | 0.0022 | A*26:12~C*07:01 | 0.0009 | A*02:11~C*16:01 | 0.0003 | A*03:01~C*18:00 | 0.0049 |
| A*68:02~C*08:02 | 0.0046 | A*33:03~C*16:01 | 0.0029 | A*23:01~C*07:02 | 0.0025 | A*31:04~C*03:04 | 0.0022 | A*29:01~C*02:10 | 0.0012 | A*02:17~C*07:01 | 0.0006 | A*03:01~C*18:02 | 0.0023 |
| A*68:02~C*15:02 | 0.0092 | A*34:02~C*04:01 | 0.0171 | A*23:01~C*07:04 | 0.0006 | A*31:04~C*16:01 | 0.0022 | A*29:01~C*04:07 | 0.0009 | A*02:17~C*15:02 | 0.0006 | A*03:02~C*04:29 | 0.0008 |
| A*68:02~C*16:01 | 0.0046 | A*34:02~C*07:01 | 0.0031 | A*23:01~C*08:02 | 0.0065 | A*32:01~C*04:01 | 0.0023 | A*29:01~C*06:02 | 0.0006 | A*02:20~C*01:02 | 0.0003 | A*11:01~C*02:02 | 0.0034 |
| A*74:01~C*03:02 | 0.0046 | A*36:01~C*04:01 | 0.0145 | A*23:01~C*08:04 | 0.0004 | A*32:01~C*07:02 | 0.0022 | A*29:02~C*02:10 | 0.0059 | A*02:22~C*01:02 | 0.0003 | A*11:01~C*03:03 | 0.0008 |
| A*74:01~C*04:01 | 0.0127 | A*36:01~C*06:02 | 0.0087 | A*23:01~C*12:03 | 0.0006 | A*32:01~C*08:02 | 0.0063 | A*29:02~C*03:04 | 0.0033 | A*02:274~C*06:02 | 0.0003 | A*11:01~C*04:01 | 0.0045 |
| A*74:01~C*06:02 | 0.0321 | A*36:01~C*07:01 | 0.0029 | A*23:01~C*15:05 | 0.0021 | A*32:01~C*12:03 | 0.0022 | A*29:02~C*04:01 | 0.0063 | A*02:30~C*12:03 | 0.0003 | A*11:01~C*05:01 | 0.0041 |
| A*74:01~C*08:02 | 0.0057 | A*36:01~C*17:01 | 0.0058 | A*23:01~C*16:01 | 0.0214 | A*32:01~C*15:02 | 0.0022 | A*29:02~C*06:02 | 0.0201 | A*02:724~C*02:10 | 0.0003 | A*11:01~C*07:02 | 0.0015 |
| A*74:01~C*14:02 | 0.0046 | A*66:01~C*04:01 | 0.0031 | A*23:01~C*17:00 | 0.0017 | A*33:01~C*03:04 | 0.0022 | A*29:02~C*07:01 | 0.0064 | A*03:01~C*01:02 | 0.0064 | A*11:01~C*16:02 | 0.0008 |
| A*74:01~C*14:03 | 0.0046 | A*66:01~C*06:02 | 0.0230 | A*23:01~C*17:01 | 0.0052 | A*33:01~C*07:01 | 0.0043 | A*29:02~C*07:06 | 0.0009 | A*03:01~C*02:02 | 0.0061 | A*11:01~C*17:00 | 0.0008 |
| A*74:01~C*16:01 | 0.0092 | A*68:01~C*07:01 | 0.0029 | A*23:01~C*18:00 | 0.0006 | A*33:01~C*14:02 | 0.0022 | A*29:02~C*12:03 | 0.0009 | A*03:01~C*03:03 | 0.0047 | A*11:02~C*07:02 | 0.0008 |
| A*74:01~C*17:01 | 0.0046 | A*68:01~C*08:02 | 0.0029 | A*24:02~C*01:02 | 0.0006 | A*33:01~C*17:01 | 0.0065 | A*29:02~C*16:01 | 0.0017 | A*03:01~C*03:04 | 0.0063 | A*23:01~C*02:10 | 0.0075 |
|  |  | A*68:01~C*14:02 | 0.0029 | A*24:02~C*04:01 | 0.0009 | A*33:01~C*18:01 | 0.0022 | A*29:02~C*17:01 | 0.0138 | A*03:01~C*04:01 | 0.0157 | A*23:01~C*03:02 | 0.0051 |
|  |  | A*68:02~C*03:02 | 0.0029 | A*24:02~C*04:03 | 0.0003 | A*33:03~C*02:10 | 0.0043 | A*29:02~C*18:01 | 0.0009 | A*03:01~C*05:01 | 0.0051 | A*23:01~C*03:04 | 0.0024 |
|  |  | A*68:02~C*03:04 | 0.0116 | A*24:02~C*07:01 | 0.0004 | A*33:03~C*03:02 | 0.0038 | A*29:02~C*18:02 | 0.0027 | A*03:01~C*06:02 | 0.0094 | A*23:01~C*04:01 | 0.0100 |
|  |  | A*68:02~C*04:01 | 0.0062 | A*24:02~C*07:02 | 0.0205 | A*33:03~C*04:01 | 0.0027 | A*30:01~C*02:10 | 0.0055 | A*03:01~C*07:01 | 0.0045 | A*23:01~C*06:02 | 0.0080 |
|  |  | A*68:02~C*04:07 | 0.0032 | A*24:02~C*07:04 | 0.0009 | A*33:03~C*07:01 | 0.0022 | A*30:01~C*03:04 | 0.0070 | A*03:01~C*07:02 | 0.0453 | A*23:01~C*07:01 | 0.0075 |
|  |  | A*68:02~C*07:01 | 0.0152 | A*24:02~C*08:01 | 0.0003 | A*34:02~C*02:02 | 0.0022 | A*30:01~C*04:01 | 0.0073 | A*03:01~C*07:04 | 0.0008 | A*23:01~C*07:02 | 0.0085 |
|  |  | A*68:02~C*07:02 | 0.0297 | A*24:02~C*14:02 | 0.0006 | A*34:02~C*02:10 | 0.0022 | A*30:01~C*06:02 | 0.0029 | A*03:01~C*08:02 | 0.0134 | A*23:01~C*08:02 | 0.0097 |
|  |  | A*68:02~C*08:02 | 0.0063 | A*24:02~C*15:25 | 0.0006 | A*34:02~C*03:02 | 0.0022 | A*30:01~C*07:01 | 0.0039 | A*03:01~C*12:03 | 0.0029 | A*23:01~C*08:04 | 0.0039 |
|  |  | A*68:02~C*12:03 | 0.0029 | A*24:02~C*16:01 | 0.0006 | A*34:02~C*04:01 | 0.0149 | A*30:01~C*07:02 | 0.0033 | A*03:01~C*14:02 | 0.0012 | A*23:01~C*08:43 | 0.0008 |
|  |  | A*68:02~C*17:01 | 0.0029 | A*24:02~C*17:01 | 0.0008 | A*34:02~C*07:01 | 0.0022 | A*30:01~C*12:03 | 0.0009 | A*03:01~C*15:02 | 0.0012 | A*23:01~C*15:05 | 0.0025 |
|  |  | A*68:02~C*18:01 | 0.0029 | A*24:02~C*18:00 | 0.0003 | A*34:02~C*07:04 | 0.0025 | A*30:01~C*14:03 | 0.0009 | A*03:01~C*15:05 | 0.0008 | A*23:01~C*16:01 | 0.0073 |
|  |  | A*74:01~C*02:10 | 0.0253 | A*24:03~C*07:02 | 0.0003 | A*34:02~C*14:02 | 0.0022 | A*30:01~C*17:01 | 0.0557 | A*03:01~C*17:03 | 0.0004 | A*23:01~C*17:01 | 0.0015 |
|  |  | A*74:01~C*03:04 | 0.0029 | A*24:07~C*07:01 | 0.0003 | A*36:01~C*04:01 | 0.0327 | A*30:01~C*18:01 | 0.0038 | A*03:02~C*03:03 | 0.0003 | A*23:01~C*18:00 | 0.0038 |
|  |  | A*74:01~C*04:01 | 0.0133 | A*26:01~C*02:10 | 0.0015 | A*36:01~C*07:01 | 0.0025 | A*30:01~C*18:02 | 0.0009 | A*03:02~C*06:02 | 0.0007 | A*24:02~C*01:02 | 0.0007 |
|  |  | A*74:01~C*06:02 | 0.0138 | A*26:01~C*04:01 | 0.0058 | A*36:01~C*08:02 | 0.0081 | A*30:02~C*02:10 | 0.0013 | A*03:02~C*07:01 | 0.0004 | A*24:02~C*02:10 | 0.0008 |
|  |  | A*74:01~C*07:01 | 0.0238 | A*26:01~C*06:02 | 0.0017 | A*66:01~C*02:10 | 0.0065 | A*30:02~C*03:04 | 0.0041 | A*03:02~C*15:02 | 0.0008 | A*24:02~C*03:03 | 0.0008 |
|  |  | A*74:01~C*16:01 | 0.0034 | A*26:01~C*07:01 | 0.0062 | A*66:01~C*03:04 | 0.0000 | A*30:02~C*04:01 | 0.0102 | A*03:02~C*17:00 | 0.0003 | A*24:02~C*03:04 | 0.0013 |
|  |  | A*74:01~C*17:01 | 0.0072 | A*26:01~C*07:02 | 0.0023 | A*66:01~C*06:02 | 0.0130 | A*30:02~C*06:02 | 0.0012 | A*03:05~C*07:02 | 0.0003 | A*24:02~C*03:05 | 0.0008 |
|  |  | A*74:02~C*16:02 | 0.0029 | A*26:01~C*08:02 | 0.0004 | A*66:01~C*07:01 | 0.0022 | A*30:02~C*07:01 | 0.0290 | A*03:21N~C*02:02 | 0.0003 | A*24:02~C*05:01 | 0.0008 |
|  |  | A*74:05~C*02:10 | 0.0029 | A*26:01~C*08:04 | 0.0007 | A*66:01~C*07:04 | 0.0043 | A*30:02~C*07:02 | 0.0017 | A*03:49~C*03:03 | 0.0003 | A*24:02~C*07:01 | 0.0008 |
|  |  | A*80:01~C*03:02 | 0.0029 | A*26:01~C*12:03 | 0.0005 | A*66:01~C*12:03 | 0.0022 | A*30:02~C*07:04 | 0.0104 | A*11:01~C*01:02 | 0.0061 | A*24:02~C*07:02 | 0.0014 |
|  |  |  |  | A*26:01~C*17:01 | 0.0008 | A*66:01~C*14:02 | 0.0022 | A*30:02~C*08:02 | 0.0186 | A*11:01~C*02:02 | 0.0030 | A*24:02~C*08:01 | 0.0015 |
|  |  |  |  | A*26:12~C*07:01 | 0.0015 | A*66:01~C*14:03 | 0.0022 | A*30:02~C*12:03 | 0.0032 | A*11:01~C*02:10 | 0.0006 | A*24:02~C*08:02 | 0.0010 |
|  |  |  |  | A*26:12~C*07:02 | 0.0000 | A*66:01~C*18:01 | 0.0022 | A*30:02~C*15:02 | 0.0009 | A*11:01~C*03:03 | 0.0037 | A*24:02~C*08:04 | 0.0043 |
|  |  |  |  | A*26:12~C*17:01 | 0.0003 | A*66:02~C*08:02 | 0.0043 | A*30:02~C*16:01 | 0.0089 | A*11:01~C*04:01 | 0.0162 | A*24:02~C*12:03 | 0.0023 |
|  |  |  |  | A*26:121~C*04:01 | 0.0006 | A*68:01~C*03:04 | 0.0022 | A*30:02~C*17:01 | 0.0049 | A*11:01~C*05:01 | 0.0039 | A*24:02~C*14:03 | 0.0008 |
|  |  |  |  | A*29:01~C*04:01 | 0.0008 | A*68:01~C*07:01 | 0.0022 | A*30:02~C*18:01 | 0.0236 | A*11:01~C*06:02 | 0.0029 | A*24:02~C*17:01 | 0.0010 |
|  |  |  |  | A*29:01~C*06:02 | 0.0005 | A*68:01~C*07:04 | 0.0043 | A*30:02~C*18:02 | 0.0132 | A*11:01~C*07:01 | 0.0039 | A*24:02~C*18:01 | 0.0008 |
|  |  |  |  | A*29:01~C*07:04 | 0.0024 | A*68:02~C*03:04 | 0.0183 | A*30:04~C*02:10 | 0.0009 | A*11:01~C*07:02 | 0.0041 | A*24:03~C*01:02 | 0.0008 |
|  |  |  |  | A*29:01~C*08:02 | 0.0003 | A*68:02~C*04:01 | 0.0187 | A*30:04~C*06:02 | 0.0027 | A*11:01~C*07:04 | 0.0009 | A*24:03~C*02:10 | 0.0008 |
|  |  |  |  | A*29:01~C*08:04 | 0.0009 | A*68:02~C*06:02 | 0.0047 | A*30:04~C*07:04 | 0.0009 | A*11:01~C*08:01 | 0.0011 | A*24:03~C*04:01 | 0.0008 |
|  |  |  |  | A*29:02~C*02:10 | 0.0042 | A*68:02~C*07:01 | 0.0134 | A*30:04~C*15:05 | 0.0018 | A*11:01~C*08:02 | 0.0034 | A*24:07~C*04:01 | 0.0008 |
|  |  |  |  | A*29:02~C*03:04 | 0.0013 | A*68:02~C*07:02 | 0.0302 | A*30:04~C*15:25 | 0.0009 | A*11:01~C*12:02 | 0.0046 | A*25:01~C*03:04 | 0.0009 |
|  |  |  |  | A*29:02~C*04:01 | 0.0016 | A*68:02~C*08:02 | 0.0050 | A*30:04~C*17new | 0.0009 | A*11:01~C*12:03 | 0.0034 | A*25:01~C*07:01 | 0.0008 |
|  |  |  |  | A*29:02~C*06:02 | 0.0079 | A*68:02~C*12:03 | 0.0022 | A*30:09~C*03:04 | 0.0009 | A*11:01~C*14:02 | 0.0023 | A*25:01~C*12:03 | 0.0044 |
|  |  |  |  | A*29:02~C*07:01 | 0.0260 | A*68:02~C*14:02 | 0.0022 | A*30:09~C*06:02 | 0.0053 | A*11:01~C*15:02 | 0.0028 | A*26:01~C*02:02 | 0.0008 |
|  |  |  |  | A*29:02~C*07:02 | 0.0026 | A*68:02~C*17:01 | 0.0028 | A*30:09~C*07:01 | 0.0009 | A*11:01~C*15:05 | 0.0003 | A*26:01~C*03:02 | 0.0007 |
|  |  |  |  | A*29:02~C*07:06 | 0.0033 | A*74:01~C*02:02 | 0.0022 | A*30:09~C*07:02 | 0.0009 | A*11:01~C*16:01 | 0.0005 | A*26:01~C*03:03 | 0.0008 |
|  |  |  |  | A*29:02~C*08:02 | 0.0004 | A*74:01~C*02:10 | 0.0165 | A*31:01~C*04:07 | 0.0009 | A*11:01~C*17:01 | 0.0002 | A*26:01~C*03:04 | 0.0022 |
|  |  |  |  | A*29:02~C*08:04 | 0.0010 | A*74:01~C*03:04 | 0.0022 | A*31:01~C*06:02 | 0.0009 | A*11:01~C*17:03 | 0.0003 | A*26:01~C*06:02 | 0.0008 |
|  |  |  |  | A*29:02~C*12:03 | 0.0009 | A*74:01~C*06:02 | 0.0278 | A*31:04~C*04:01 | 0.0009 | A*11:04~C*04:01 | 0.0006 | A*26:01~C*06:08 | 0.0015 |
|  |  |  |  | A*29:02~C*14:03 | 0.0001 | A*74:01~C*07:01 | 0.0185 | A*32:01~C*06:02 | 0.0009 | A*11:67~C*07:01 | 0.0003 | A*26:01~C*07:01 | 0.0008 |
|  |  |  |  | A*29:02~C*15:05 | 0.0003 | A*74:01~C*08:02 | 0.0173 | A*32:01~C*12:03 | 0.0018 | A*23:01~C*02:02 | 0.0007 | A*26:01~C*08:04 | 0.0008 |
|  |  |  |  | A*29:02~C*16:01 | 0.0031 | A*74:03~C*04:01 | 0.0022 | A*32:01~C*14:02 | 0.0009 | A*23:01~C*02:10 | 0.0006 | A*26:01~C*12:03 | 0.0015 |
|  |  |  |  | A*29:02~C*17:00 | 0.0061 | A*80:01~C*02:02 | 0.0022 | A*33:01~C*02:10 | 0.0026 | A*23:01~C*04:01 | 0.0064 | A*26:01~C*18:00 | 0.0008 |
|  |  |  |  | A*29:02~C*17:01 | 0.0097 | A*80:01~C*03:02 | 0.0022 | A*33:01~C*03:02 | 0.0009 | A*23:01~C*05:01 | 0.0007 | A*26:01~C*18:02 | 0.0015 |
|  |  |  |  | A*29:02~C*18:00 | 0.0005 | A*80:01~C*06:02 | 0.0022 | A*33:01~C*04:01 | 0.0009 | A*23:01~C*06:02 | 0.0007 | A*29:01~C*03:03 | 0.0008 |
|  |  |  |  | A*29:11~C*04:01 | 0.0010 |  |  | A*33:01~C*07:01 | 0.0025 | A*23:01~C*07:01 | 0.0056 | A*29:02~C*02:02 | 0.0023 |
|  |  |  |  | A*29:11~C*06:02 | 0.0118 |  |  | A*33:01~C*08:02 | 0.0018 | A*23:01~C*07:02 | 0.0010 | A*29:02~C*02:10 | 0.0015 |
|  |  |  |  | A*29:11~C*07:01 | 0.0008 |  |  | A*33:01~C*14:02 | 0.0009 | A*23:01~C*08:02 | 0.0003 | A*29:02~C*04:01 | 0.0053 |
|  |  |  |  | A*29:11~C*17:01 | 0.0003 |  |  | A*33:01~C*16:01 | 0.0002 | A*23:01~C*12:03 | 0.0008 | A*29:02~C*04:27 | 0.0008 |
|  |  |  |  | A*30:01~C*02:02 | 0.0020 |  |  | A*33:01~C*17:01 | 0.0026 | A*23:01~C*15:05 | 0.0003 | A*29:02~C*06:02 | 0.0025 |
|  |  |  |  | A*30:01~C*02:10 | 0.0088 |  |  | A*33:03~C*02:10 | 0.0023 | A*23:01~C*17:01 | 0.0005 | A*29:02~C*07:01 | 0.0026 |
|  |  |  |  | A*30:01~C*03:02 | 0.0029 |  |  | A*33:03~C*03:02 | 0.0009 | A*23:01~C*17:03 | 0.0006 | A*29:02~C*07:02 | 0.0011 |
|  |  |  |  | A*30:01~C*03:04 | 0.0013 |  |  | A*33:03~C*03:04 | 0.0027 | A*24:02~C*01:02 | 0.0033 | A*29:02~C*08:02 | 0.0013 |
|  |  |  |  | A*30:01~C*04:01 | 0.0053 |  |  | A*33:03~C*04:01 | 0.0021 | A*24:02~C*02:02 | 0.0097 | A*29:02~C*08:04 | 0.0027 |
|  |  |  |  | A*30:01~C*05:01 | 0.0009 |  |  | A*33:03~C*05:01 | 0.0018 | A*24:02~C*02:10 | 0.0003 | A*29:02~C*12:03 | 0.0008 |
|  |  |  |  | A*30:01~C*06:02 | 0.0040 |  |  | A*33:03~C*07:04 | 0.0009 | A*24:02~C*03:02 | 0.0002 | A*29:02~C*14:02 | 0.0008 |
|  |  |  |  | A*30:01~C*07:01 | 0.0033 |  |  | A*33:03~C*14:02 | 0.0009 | A*24:02~C*03:03 | 0.0076 | A*29:02~C*15:02 | 0.0015 |
|  |  |  |  | A*30:01~C*07:02 | 0.0026 |  |  | A*33:03~C*16:01 | 0.0009 | A*24:02~C*03:04 | 0.0040 | A*29:02~C*15:05 | 0.0023 |
|  |  |  |  | A*30:01~C*07:04 | 0.0035 |  |  | A*34:02~C*02:10 | 0.0015 | A*24:02~C*03:05 | 0.0003 | A*29:02~C*16:01 | 0.0016 |
|  |  |  |  | A*30:01~C*07:06 | 0.0003 |  |  | A*34:02~C*03:02 | 0.0018 | A*24:02~C*03:06 | 0.0003 | A*29:02~C*18:00 | 0.0012 |
|  |  |  |  | A*30:01~C*08:04 | 0.0000 |  |  | A*34:02~C*04:01 | 0.0149 | A*24:02~C*04:01 | 0.0123 | A*29:02~C*18:01 | 0.0027 |
|  |  |  |  | A*30:01~C*12:03 | 0.0010 |  |  | A*34:02~C*05:01 | 0.0040 | A*24:02~C*05:01 | 0.0015 | A*29:02~C*18:02 | 0.0008 |
|  |  |  |  | A*30:01~C*16:01 | 0.0010 |  |  | A*34:02~C*06:02 | 0.0062 | A*24:02~C*06:02 | 0.0088 | A*30:01~C*02:10 | 0.0019 |
|  |  |  |  | A*30:01~C*17:00 | 0.0260 |  |  | A*34:02~C*07:01 | 0.0082 | A*24:02~C*07:01 | 0.0064 | A*30:01~C*03:04 | 0.0015 |
|  |  |  |  | A*30:01~C*17:01 | 0.0355 |  |  | A*34:02~C*07:04 | 0.0009 | A*24:02~C*07:02 | 0.0110 | A*30:01~C*04:01 | 0.0015 |
|  |  |  |  | A*30:01~C*18:00 | 0.0008 |  |  | A*34:02~C*07:06 | 0.0035 | A*24:02~C*08:02 | 0.0011 | A*30:01~C*04:07 | 0.0008 |
|  |  |  |  | A*30:01~C*18:02 | 0.0005 |  |  | A*34:02~C*08:04 | 0.0006 | A*24:02~C*12:02 | 0.0004 | A*30:01~C*06:02 | 0.0008 |
|  |  |  |  | A*30:02~C*02:10 | 0.0040 |  |  | A*34:02~C*15:05 | 0.0009 | A*24:02~C*12:03 | 0.0083 | A*30:01~C*07:01 | 0.0009 |
|  |  |  |  | A*30:02~C*03:04 | 0.0037 |  |  | A*36:01~C*02:10 | 0.0010 | A*24:02~C*14:02 | 0.0008 | A*30:01~C*07:02 | 0.0058 |
|  |  |  |  | A*30:02~C*05:01 | 0.0003 |  |  | A*36:01~C*03:04 | 0.0021 | A*24:02~C*15:02 | 0.0015 | A*30:01~C*08:02 | 0.0024 |
|  |  |  |  | A*30:02~C*06:02 | 0.0118 |  |  | A*36:01~C*04:01 | 0.0300 | A*24:02~C*15:05 | 0.0003 | A*30:01~C*14:02 | 0.0008 |
|  |  |  |  | A*30:02~C*07:01 | 0.0108 |  |  | A*36:01~C*06:02 | 0.0020 | A*24:02~C*16:01 | 0.0017 | A*30:01~C*16:01 | 0.0041 |
|  |  |  |  | A*30:02~C*07:02 | 0.0036 |  |  | A*36:01~C*07:01 | 0.0015 | A*24:02~C*16:04 | 0.0003 | A*30:01~C*17:00 | 0.0061 |
|  |  |  |  | A*30:02~C*07:04 | 0.0054 |  |  | A*36:01~C*07:02 | 0.0024 | A*24:02~C*17:00 | 0.0005 | A*30:01~C*17:01 | 0.0156 |
|  |  |  |  | A*30:02~C*08:02 | 0.0031 |  |  | A*36:01~C*07:04 | 0.0018 | A*24:03~C*01:02 | 0.0008 | A*30:01~C*18:00 | 0.0041 |
|  |  |  |  | A*30:02~C*12:03 | 0.0079 |  |  | A*36:01~C*16:01 | 0.0018 | A*24:03~C*02:02 | 0.0006 | A*30:01~C*18:02 | 0.0060 |
|  |  |  |  | A*30:02~C*16:01 | 0.0094 |  |  | A*36:01~C*17:01 | 0.0026 | A*24:03~C*06:02 | 0.0003 | A*30:02~C*02:02 | 0.0015 |
|  |  |  |  | A*30:02~C*17:00 | 0.0040 |  |  | A*36:01~C*18:01 | 0.0010 | A*24:03~C*07:01 | 0.0013 | A*30:02~C*02:10 | 0.0052 |
|  |  |  |  | A*30:02~C*17:01 | 0.0052 |  |  | A*43:01~C*08:02 | 0.0009 | A*24:03~C*08:02 | 0.0003 | A*30:02~C*03:04 | 0.0012 |
|  |  |  |  | A*30:02~C*18:00 | 0.0021 |  |  | A*43:01~C*17:01 | 0.0009 | A*24:03~C*12:03 | 0.0003 | A*30:02~C*04:01 | 0.0128 |
|  |  |  |  | A*30:02~C*18:01 | 0.0003 |  |  | A*66:01~C*03:02 | 0.0010 | A*24:03~C*16:01 | 0.0002 | A*30:02~C*05:01 | 0.0030 |
|  |  |  |  | A*30:02~C*18:02 | 0.0009 |  |  | A*66:01~C*03:04 | 0.0027 | A*24:05~C*03:04 | 0.0003 | A*30:02~C*06:02 | 0.0083 |
|  |  |  |  | A*30:03~C*02:10 | 0.0003 |  |  | A*66:01~C*04:01 | 0.0053 | A*24:23~C*14:02 | 0.0003 | A*30:02~C*07:01 | 0.0147 |
|  |  |  |  | A*30:04~C*02:10 | 0.0029 |  |  | A*66:01~C*06:02 | 0.0128 | A*24:314~C*08:02 | 0.0003 | A*30:02~C*07:04 | 0.0015 |
|  |  |  |  | A*30:04~C*02:17 | 0.0024 |  |  | A*66:01~C*07:01 | 0.0000 | A*24:95~C*12:03 | 0.0003 | A*30:02~C*08:02 | 0.0034 |
|  |  |  |  | A*30:04~C*03:02 | 0.0024 |  |  | A*66:01~C*08:02 | 0.0009 | A*25:01~C*01:02 | 0.0004 | A*30:02~C*08:04 | 0.0033 |
|  |  |  |  | A*30:04~C*04:01 | 0.0026 |  |  | A*66:01~C*12:03 | 0.0022 | A*25:01~C*02:02 | 0.0005 | A*30:02~C*12:03 | 0.0019 |
|  |  |  |  | A*30:04~C*06:02 | 0.0022 |  |  | A*66:01~C*14:02 | 0.0044 | A*25:01~C*03:02 | 0.0003 | A*30:02~C*14:03 | 0.0023 |
|  |  |  |  | A*30:04~C*07:01 | 0.0004 |  |  | A*66:01~C*14:03 | 0.0009 | A*25:01~C*03:03 | 0.0014 | A*30:02~C*16:01 | 0.0055 |
|  |  |  |  | A*30:04~C*07:02 | 0.0020 |  |  | A*66:01~C*16:01 | 0.0013 | A*25:01~C*03:04 | 0.0009 | A*30:02~C*17:01 | 0.0009 |
|  |  |  |  | A*30:04~C*07:04 | 0.0003 |  |  | A*66:01~C*17:01 | 0.0013 | A*25:01~C*04:01 | 0.0013 | A*30:02~C*18:00 | 0.0103 |
|  |  |  |  | A*30:04~C*08:02 | 0.0007 |  |  | A*66:02~C*06:02 | 0.0009 | A*25:01~C*05:01 | 0.0018 | A*30:02~C*18:02 | 0.0095 |
|  |  |  |  | A*30:04~C*08:04 | 0.0008 |  |  | A*66:02~C*07:01 | 0.0018 | A*25:01~C*06:02 | 0.0010 | A*30:03~C*07:01 | 0.0008 |
|  |  |  |  | A*30:04~C*14:02 | 0.0006 |  |  | A*66:03~C*18:01 | 0.0018 | A*25:01~C*07:01 | 0.0043 | A*30:04~C*02:10 | 0.0008 |
|  |  |  |  | A*30:04~C*15:05 | 0.0003 |  |  | A*68:01~C*02:10 | 0.0003 | A*25:01~C*07:02 | 0.0013 | A*30:04~C*06:02 | 0.0008 |
|  |  |  |  | A*30:04~C*16:01 | 0.0008 |  |  | A*68:01~C*03:04 | 0.0009 | A*25:01~C*07:06 | 0.0003 | A*30:04~C*15:05 | 0.0008 |
|  |  |  |  | A*30:04~C*17:00 | 0.0008 |  |  | A*68:01~C*04:01 | 0.0048 | A*25:01~C*08:02 | 0.0005 | A*30:09~C*06:02 | 0.0008 |
|  |  |  |  | A*30:04~C*17:01 | 0.0019 |  |  | A*68:01~C*06:02 | 0.0009 | A*25:01~C*12:03 | 0.0140 | A*30:10~C*04:07 | 0.0008 |
|  |  |  |  | A*30:04~C*18:01 | 0.0003 |  |  | A*68:01~C*07:01 | 0.0010 | A*25:01~C*15:02 | 0.0003 | A*30:10~C*06:02 | 0.0008 |
|  |  |  |  | A*30:09~C*04:01 | 0.0043 |  |  | A*68:01~C*08:02 | 0.0045 | A*25:01~C*16:01 | 0.0007 | A*30:151~C*18:00 | 0.0008 |
|  |  |  |  | A*30:09~C*17:00 | 0.0003 |  |  | A*68:02~C*02:10 | 0.0025 | A*26:01~C*01:02 | 0.0019 | A*31:01~C*01:02 | 0.0008 |
|  |  |  |  | A*31:01~C*07:02 | 0.0003 |  |  | A*68:02~C*03:02 | 0.0027 | A*26:01~C*02:02 | 0.0025 | A*31:01~C*03:04 | 0.0054 |
|  |  |  |  | A*31:01~C*18:00 | 0.0009 |  |  | A*68:02~C*03:04 | 0.0304 | A*26:01~C*04:01 | 0.0027 | A*31:01~C*04:01 | 0.0008 |
|  |  |  |  | A*32:01~C*01:02 | 0.0003 |  |  | A*68:02~C*04:01 | 0.0142 | A*26:01~C*05:01 | 0.0020 | A*31:01~C*07:01 | 0.0097 |
|  |  |  |  | A*32:01~C*02:10 | 0.0015 |  |  | A*68:02~C*05:01 | 0.0030 | A*26:01~C*06:02 | 0.0038 | A*31:01~C*07:621 | 0.0008 |
|  |  |  |  | A*32:01~C*04:01 | 0.0012 |  |  | A*68:02~C*06:02 | 0.0040 | A*26:01~C*07:01 | 0.0035 | A*31:01~C*14:03 | 0.0008 |
|  |  |  |  | A*32:01~C*07:02 | 0.0006 |  |  | A*68:02~C*07:01 | 0.0039 | A*26:01~C*07:02 | 0.0011 | A*31:01~C*15:02 | 0.0008 |
|  |  |  |  | A*32:01~C*17:00 | 0.0003 |  |  | A*68:02~C*07:02 | 0.0107 | A*26:01~C*08:01 | 0.0004 | A*31:01~C*16:01 | 0.0008 |
|  |  |  |  | A*32:01~C*17:01 | 0.0003 |  |  | A*68:02~C*07:04 | 0.0010 | A*26:01~C*08:02 | 0.0014 | A*32:01~C*02:02 | 0.0036 |
|  |  |  |  | A*32:106~C*02:10 | 0.0009 |  |  | A*68:02~C*08:02 | 0.0160 | A*26:01~C*12:02 | 0.0002 | A*32:01~C*03:04 | 0.0008 |
|  |  |  |  | A*32:106~C*06:02 | 0.0003 |  |  | A*68:02~C*12:03 | 0.0025 | A*26:01~C*12:03 | 0.0079 | A*32:01~C*04:01 | 0.0009 |
|  |  |  |  | A*32:106~C*07:02 | 0.0006 |  |  | A*68:02~C*16:01 | 0.0025 | A*26:01~C*14:02 | 0.0004 | A*32:01~C*06:02 | 0.0011 |
|  |  |  |  | A*33:01~C*02:10 | 0.0009 |  |  | A*68:02~C*17:01 | 0.0041 | A*26:01~C*15:02 | 0.0014 | A*32:01~C*07:02 | 0.0023 |
|  |  |  |  | A*33:01~C*07:02 | 0.0001 |  |  | A*68:02~C*18:01 | 0.0024 | A*26:01~C*15:05 | 0.0006 | A*32:01~C*07:04 | 0.0015 |
|  |  |  |  | A*33:01~C*08:02 | 0.0003 |  |  | A*74:01~C*02:02 | 0.0015 | A*26:08~C*02:02 | 0.0003 | A*32:01~C*08:02 | 0.0015 |
|  |  |  |  | A*33:01~C*17:00 | 0.0027 |  |  | A*74:01~C*02:10 | 0.0292 | A*26:08~C*03:04 | 0.0003 | A*32:01~C*08:04 | 0.0008 |
|  |  |  |  | A*33:01~C*17:01 | 0.0030 |  |  | A*74:01~C*04:01 | 0.0191 | A*26:08~C*12:03 | 0.0008 | A*32:01~C*08:13 | 0.0008 |
|  |  |  |  | A*33:01~C*18:01 | 0.0003 |  |  | A*74:01~C*06:02 | 0.0068 | A*26:08~C*15:02 | 0.0003 | A*32:01~C*12:03 | 0.0008 |
|  |  |  |  | A*33:03~C*03:02 | 0.0003 |  |  | A*74:01~C*07:01 | 0.0021 | A*29:01~C*04:01 | 0.0003 | A*32:01~C*14:03 | 0.0008 |
|  |  |  |  | A*33:03~C*04:01 | 0.0056 |  |  | A*74:01~C*08:02 | 0.0020 | A*29:01~C*05:01 | 0.0002 | A*32:01~C*18:00 | 0.0020 |
|  |  |  |  | A*33:03~C*06:02 | 0.0003 |  |  | A*74:01~C*15:02 | 0.0009 | A*29:01~C*06:02 | 0.0003 | A*33:01~C*03:02 | 0.0008 |
|  |  |  |  | A*33:03~C*07:02 | 0.0054 |  |  | A*74:01~C*17:01 | 0.0050 | A*29:01~C*07:02 | 0.0004 | A*33:01~C*04:01 | 0.0010 |
|  |  |  |  | A*33:03~C*14:02 | 0.0003 |  |  | A*74:01~C*18:01 | 0.0025 | A*29:01~C*08:02 | 0.0002 | A*33:01~C*07:01 | 0.0029 |
|  |  |  |  | A*33:03~C*17:00 | 0.0006 |  |  | A*80:01~C*02:02 | 0.0009 | A*29:01~C*12:02 | 0.0005 | A*33:01~C*08:02 | 0.0050 |
|  |  |  |  | A*33:03~C*17:01 | 0.0023 |  |  | A*80:01~C*02:10 | 0.0009 | A*29:01~C*15:02 | 0.0003 | A*33:01~C*12:03 | 0.0009 |
|  |  |  |  | A*34:01~C*06:02 | 0.0003 |  |  | A*80:01~C*03:02 | 0.0009 | A*29:01~C*15:05 | 0.0014 | A*33:01~C*16:01 | 0.0040 |
|  |  |  |  | A*34:02~C*02:10 | 0.0045 |  |  | A*80:01~C*04:01 | 0.0018 | A*29:02~C*01:02 | 0.0014 | A*33:01~C*17:01 | 0.0015 |
|  |  |  |  | A*34:02~C*04:01 | 0.0214 |  |  |  |  | A*29:02~C*02:02 | 0.0009 | A*33:01~C*18:00 | 0.0013 |
|  |  |  |  | A*34:02~C*06:02 | 0.0032 |  |  |  |  | A*29:02~C*04:01 | 0.0007 | A*33:03~C*01:02 | 0.0008 |
|  |  |  |  | A*34:02~C*07:01 | 0.0080 |  |  |  |  | A*29:02~C*05:01 | 0.0017 | A*33:03~C*03:02 | 0.0058 |
|  |  |  |  | A*34:02~C*07:04 | 0.0003 |  |  |  |  | A*29:02~C*06:02 | 0.0013 | A*33:03~C*03:04 | 0.0027 |
|  |  |  |  | A*34:02~C*08:02 | 0.0003 |  |  |  |  | A*29:02~C*07:01 | 0.0017 | A*33:03~C*04:01 | 0.0190 |
|  |  |  |  | A*34:02~C*08:04 | 0.0035 |  |  |  |  | A*29:02~C*07:02 | 0.0025 | A*33:03~C*04:13 | 0.0015 |
|  |  |  |  | A*34:02~C*12:03 | 0.0009 |  |  |  |  | A*29:02~C*08:02 | 0.0013 | A*33:03~C*05:01 | 0.0008 |
|  |  |  |  | A*34:02~C*17:00 | 0.0008 |  |  |  |  | A*29:02~C*15:02 | 0.0003 | A*33:03~C*07:01 | 0.0093 |
|  |  |  |  | A*34:02~C*17:01 | 0.0007 |  |  |  |  | A*29:02~C*16:01 | 0.0236 | A*33:03~C*07:02 | 0.0023 |
|  |  |  |  | A*34:02~C*18:02 | 0.0003 |  |  |  |  | A*29:02~C*17:00 | 0.0006 | A*33:03~C*12:03 | 0.0016 |
|  |  |  |  | A*36:01~C*04:01 | 0.0034 |  |  |  |  | A*29:02~C*17:03 | 0.0002 | A*33:03~C*14:02 | 0.0058 |
|  |  |  |  | A*36:01~C*07:04 | 0.0003 |  |  |  |  | A*29:02~C*18:00 | 0.0004 | A*33:03~C*15:05 | 0.0020 |
|  |  |  |  | A*36:01~C*17:00 | 0.0006 |  |  |  |  | A*29:10~C*08:02 | 0.0006 | A*33:03~C*16:01 | 0.0011 |
|  |  |  |  | A*43:01~C*02:10 | 0.0022 |  |  |  |  | A*30:01~C*03:03 | 0.0006 | A*33:03~C*17:00 | 0.0008 |
|  |  |  |  | A*43:01~C*03:04 | 0.0003 |  |  |  |  | A*30:01~C*04:01 | 0.0016 | A*33:03~C*17:01 | 0.0014 |
|  |  |  |  | A*43:01~C*04:01 | 0.0028 |  |  |  |  | A*30:01~C*06:02 | 0.0136 | A*33:03~C*18:00 | 0.0041 |
|  |  |  |  | A*43:01~C*06:02 | 0.0106 |  |  |  |  | A*30:01~C*07:01 | 0.0007 | A*33:05~C*07:02 | 0.0008 |
|  |  |  |  | A*43:01~C*07:01 | 0.0009 |  |  |  |  | A*30:01~C*07:02 | 0.0003 | A*34:02~C*02:10 | 0.0031 |
|  |  |  |  | A*43:01~C*07:02 | 0.0005 |  |  |  |  | A*30:01~C*12:03 | 0.0016 | A*34:02~C*03:02 | 0.0009 |
|  |  |  |  | A*43:01~C*08:04 | 0.0013 |  |  |  |  | A*30:01~C*14:02 | 0.0001 | A*34:02~C*03:03 | 0.0008 |
|  |  |  |  | A*43:01~C*12:03 | 0.0006 |  |  |  |  | A*30:01~C*17:01 | 0.0006 | A*34:02~C*03:04 | 0.0008 |
|  |  |  |  | A*43:01~C*18:00 | 0.0058 |  |  |  |  | A*30:01~C*18:00 | 0.0003 | A*34:02~C*04:01 | 0.0110 |
|  |  |  |  | A*43:01~C*18:01 | 0.0003 |  |  |  |  | A*30:02~C*01:02 | 0.0003 | A*34:02~C*06:02 | 0.0023 |
|  |  |  |  | A*43:01~C*18:02 | 0.0018 |  |  |  |  | A*30:02~C*03:02 | 0.0006 | A*34:02~C*07:01 | 0.0042 |
|  |  |  |  | A*66:01~C*02:17 | 0.0003 |  |  |  |  | A*30:02~C*04:01 | 0.0019 | A*34:02~C*07:02 | 0.0031 |
|  |  |  |  | A*66:01~C*03:02 | 0.0007 |  |  |  |  | A*30:02~C*05:01 | 0.0031 | A*34:02~C*08:02 | 0.0011 |
|  |  |  |  | A*66:01~C*03:04 | 0.0007 |  |  |  |  | A*30:02~C*06:02 | 0.0004 | A*34:02~C*08:04 | 0.0027 |
|  |  |  |  | A*66:01~C*04:01 | 0.0024 |  |  |  |  | A*30:02~C*07:01 | 0.0009 | A*34:02~C*12:03 | 0.0017 |
|  |  |  |  | A*66:01~C*06:02 | 0.0277 |  |  |  |  | A*30:02~C*08:02 | 0.0004 | A*34:02~C*16:01 | 0.0019 |
|  |  |  |  | A*66:01~C*07:01 | 0.0009 |  |  |  |  | A*30:02~C*12:02 | 0.0003 | A*34:02~C*17:03 | 0.0008 |
|  |  |  |  | A*66:01~C*08:04 | 0.0005 |  |  |  |  | A*30:02~C*16:01 | 0.0011 | A*34:02~C*18:00 | 0.0020 |
|  |  |  |  | A*66:01~C*12:03 | 0.0043 |  |  |  |  | A*30:02~C*17:01 | 0.0003 | A*36:01~C*03:04 | 0.0017 |
|  |  |  |  | A*66:01~C*17:00 | 0.0009 |  |  |  |  | A*30:02~C*18:00 | 0.0010 | A*36:01~C*04:01 | 0.0119 |
|  |  |  |  | A*66:01~C*18:00 | 0.0015 |  |  |  |  | A*30:02~C*18:02 | 0.0003 | A*36:01~C*06:02 | 0.0009 |
|  |  |  |  | A*66:02~C*02:10 | 0.0004 |  |  |  |  | A*30:04~C*03:04 | 0.0003 | A*36:01~C*12:03 | 0.0008 |
|  |  |  |  | A*66:02~C*04:01 | 0.0004 |  |  |  |  | A*30:04~C*07:01 | 0.0015 | A*36:01~C*14:02 | 0.0013 |
|  |  |  |  | A*66:02~C*17:00 | 0.0038 |  |  |  |  | A*30:04~C*07:02 | 0.0003 | A*36:01~C*15:02 | 0.0014 |
|  |  |  |  | A*66:02~C*17:01 | 0.0058 |  |  |  |  | A*30:04~C*08:02 | 0.0007 | A*36:01~C*16:01 | 0.0015 |
|  |  |  |  | A*66:03~C*04:01 | 0.0006 |  |  |  |  | A*30:04~C*12:02 | 0.0003 | A*36:01~C*17:00 | 0.0015 |
|  |  |  |  | A*66:03~C*07:01 | 0.0003 |  |  |  |  | A*30:04~C*17:01 | 0.0003 | A*66:01~C*03:04 | 0.0006 |
|  |  |  |  | A*68:01~C*02:02 | 0.0003 |  |  |  |  | A*30:10~C*06:02 | 0.0006 | A*66:01~C*04:01 | 0.0021 |
|  |  |  |  | A*68:01~C*02:10 | 0.0012 |  |  |  |  | A*31:01~C*01:02 | 0.0007 | A*66:01~C*04:07 | 0.0008 |
|  |  |  |  | A*68:01~C*03:02 | 0.0013 |  |  |  |  | A*31:01~C*01:127 | 0.0006 | A*66:01~C*06:02 | 0.0067 |
|  |  |  |  | A*68:01~C*04:01 | 0.0013 |  |  |  |  | A*31:01~C*02:02 | 0.0021 | A*66:01~C*07:02 | 0.0015 |
|  |  |  |  | A*68:01~C*06:02 | 0.0196 |  |  |  |  | A*31:01~C*03:03 | 0.0012 | A*66:01~C*12:03 | 0.0028 |
|  |  |  |  | A*68:01~C*16:01 | 0.0004 |  |  |  |  | A*31:01~C*03:04 | 0.0090 | A*66:01~C*14:03 | 0.0008 |
|  |  |  |  | A*68:01~C*16:04 | 0.0003 |  |  |  |  | A*31:01~C*04:01 | 0.0052 | A*66:01~C*16:01 | 0.0015 |
|  |  |  |  | A*68:01~C*17:00 | 0.0010 |  |  |  |  | A*31:01~C*04:08 | 0.0003 | A*66:01~C*17:01 | 0.0014 |
|  |  |  |  | A*68:01~C*17:01 | 0.0026 |  |  |  |  | A*31:01~C*05:01 | 0.0014 | A*66:01~C*17:03 | 0.0008 |
|  |  |  |  | A*68:01~C*18:00 | 0.0003 |  |  |  |  | A*31:01~C*06:02 | 0.0030 | A*66:01~C*18:00 | 0.0013 |
|  |  |  |  | A*68:02~C*02:02 | 0.0010 |  |  |  |  | A*31:01~C*07:01 | 0.0016 | A*66:01~C*18:02 | 0.0009 |
|  |  |  |  | A*68:02~C*02:05 | 0.0003 |  |  |  |  | A*31:01~C*07:02 | 0.0020 | A*66:02~C*04:01 | 0.0008 |
|  |  |  |  | A*68:02~C*02:10 | 0.0014 |  |  |  |  | A*31:01~C*08:01 | 0.0003 | A*66:02~C*07:01 | 0.0075 |
|  |  |  |  | A*68:02~C*03:02 | 0.0016 |  |  |  |  | A*31:01~C*08:02 | 0.0005 | A*66:03~C*03:03 | 0.0008 |
|  |  |  |  | A*68:02~C*03:04 | 0.0269 |  |  |  |  | A*31:01~C*12:03 | 0.0020 | A*66:03~C*07:06 | 0.0008 |
|  |  |  |  | A*68:02~C*04:01 | 0.0043 |  |  |  |  | A*31:01~C*14:02 | 0.0013 | A*68:01~C*02:10 | 0.0016 |
|  |  |  |  | A*68:02~C*04:04 | 0.0003 |  |  |  |  | A*31:01~C*15:02 | 0.0045 | A*68:01~C*03:04 | 0.0023 |
|  |  |  |  | A*68:02~C*04:226 | 0.0003 |  |  |  |  | A*31:01~C*16:01 | 0.0002 | A*68:01~C*04:01 | 0.0041 |
|  |  |  |  | A*68:02~C*05:01 | 0.0009 |  |  |  |  | A*31:02~C*07:01 | 0.0003 | A*68:01~C*06:02 | 0.0050 |
|  |  |  |  | A*68:02~C*07:01 | 0.0047 |  |  |  |  | A*32:01~C*01:02 | 0.0047 | A*68:01~C*07:02 | 0.0023 |
|  |  |  |  | A*68:02~C*07:02 | 0.0139 |  |  |  |  | A*32:01~C*02:02 | 0.0064 | A*68:01~C*07:04 | 0.0008 |
|  |  |  |  | A*68:02~C*07:04 | 0.0006 |  |  |  |  | A*32:01~C*03:03 | 0.0038 | A*68:01~C*08:02 | 0.0032 |
|  |  |  |  | A*68:02~C*07:06 | 0.0012 |  |  |  |  | A*32:01~C*03:04 | 0.0033 | A*68:01~C*12:03 | 0.0018 |
|  |  |  |  | A*68:02~C*08:02 | 0.0073 |  |  |  |  | A*32:01~C*04:01 | 0.0028 | A*68:01~C*15:02 | 0.0012 |
|  |  |  |  | A*68:02~C*08:04 | 0.0126 |  |  |  |  | A*32:01~C*04:07 | 0.0003 | A*68:01~C*15:05 | 0.0013 |
|  |  |  |  | A*68:02~C*14:02 | 0.0003 |  |  |  |  | A*32:01~C*05:01 | 0.0075 | A*68:01~C*16:01 | 0.0015 |
|  |  |  |  | A*68:02~C*14:03 | 0.0005 |  |  |  |  | A*32:01~C*06:02 | 0.0034 | A*68:01~C*18:00 | 0.0037 |
|  |  |  |  | A*68:02~C*15:05 | 0.0023 |  |  |  |  | A*32:01~C*07:01 | 0.0040 | A*68:02~C*02:10 | 0.0010 |
|  |  |  |  | A*68:02~C*17:00 | 0.0016 |  |  |  |  | A*32:01~C*07:02 | 0.0009 | A*68:02~C*03:02 | 0.0015 |
|  |  |  |  | A*68:02~C*17:01 | 0.0018 |  |  |  |  | A*32:01~C*08:02 | 0.0059 | A*68:02~C*03:04 | 0.0071 |
|  |  |  |  | A*68:02~C*18:00 | 0.0020 |  |  |  |  | A*32:01~C*08:04 | 0.0003 | A*68:02~C*04:01 | 0.0136 |
|  |  |  |  | A*68:02~C*18:02 | 0.0016 |  |  |  |  | A*32:01~C*12:03 | 0.0004 | A*68:02~C*05:01 | 0.0011 |
|  |  |  |  | A*68:04~C*06:02 | 0.0003 |  |  |  |  | A*32:01~C*14:02 | 0.0004 | A*68:02~C*06:02 | 0.0023 |
|  |  |  |  | A*68:27~C*04:01 | 0.0013 |  |  |  |  | A*32:01~C*15:02 | 0.0009 | A*68:02~C*07:01 | 0.0105 |
|  |  |  |  | A*68:27~C*07:01 | 0.0005 |  |  |  |  | A*32:01~C*15:05 | 0.0005 | A*68:02~C*07:02 | 0.0070 |
|  |  |  |  | A*68:27~C*08:04 | 0.0003 |  |  |  |  | A*32:01~C*16:01 | 0.0006 | A*68:02~C*07:05 | 0.0008 |
|  |  |  |  | A*68:27~C*17:01 | 0.0003 |  |  |  |  | A*32:01~C*17:01 | 0.0002 | A*68:02~C*08:02 | 0.0035 |
|  |  |  |  | A*69:02~C*06:02 | 0.0003 |  |  |  |  | A*32:114~C*08:02 | 0.0003 | A*68:02~C*08:04 | 0.0022 |
|  |  |  |  | A*74:00~C*02:10 | 0.0083 |  |  |  |  | A*33:01~C*02:02 | 0.0006 | A*68:02~C*14:02 | 0.0018 |
|  |  |  |  | A*74:00~C*03:02 | 0.0003 |  |  |  |  | A*33:01~C*02:10 | 0.0003 | A*68:02~C*16:01 | 0.0024 |
|  |  |  |  | A*74:00~C*04:01 | 0.0064 |  |  |  |  | A*33:01~C*05:01 | 0.0003 | A*68:02~C*17:00 | 0.0010 |
|  |  |  |  | A*74:00~C*07:01 | 0.0069 |  |  |  |  | A*33:01~C*07:04 | 0.0004 | A*68:02~C*18:00 | 0.0026 |
|  |  |  |  | A*74:00~C*17:00 | 0.0006 |  |  |  |  | A*33:01~C*08:02 | 0.0078 | A*68:02~C*18:01 | 0.0015 |
|  |  |  |  | A*74:01~C*02:10 | 0.0212 |  |  |  |  | A*33:03~C*01:02 | 0.0003 | A*68:10~C*05:01 | 0.0008 |
|  |  |  |  | A*74:01~C*03:02 | 0.0006 |  |  |  |  | A*33:03~C*03:02 | 0.0011 | A*69:01~C*07:04 | 0.0008 |
|  |  |  |  | A*74:01~C*03:04 | 0.0003 |  |  |  |  | A*33:03~C*04:01 | 0.0013 | A*74:00~C*02:10 | 0.0020 |
|  |  |  |  | A*74:01~C*04:01 | 0.0066 |  |  |  |  | A*33:03~C*06:02 | 0.0010 | A*74:00~C*04:01 | 0.0045 |
|  |  |  |  | A*74:01~C*07:01 | 0.0055 |  |  |  |  | A*33:03~C*07:01 | 0.0004 | A*74:00~C*07:01 | 0.0051 |
|  |  |  |  | A*74:01~C*07:02 | 0.0004 |  |  |  |  | A*33:03~C*08:01 | 0.0003 | A*74:00~C*08:04 | 0.0008 |
|  |  |  |  | A*74:01~C*07:04 | 0.0003 |  |  |  |  | A*33:03~C*14:02 | 0.0001 | A*74:00~C*17:00 | 0.0013 |
|  |  |  |  | A*74:01~C*07:06 | 0.0003 |  |  |  |  | A*33:03~C*15:05 | 0.0003 | A*74:00~C*18:00 | 0.0015 |
|  |  |  |  | A*74:01~C*17:01 | 0.0014 |  |  |  |  | A*33:03~C*18:01 | 0.0003 | A*74:01~C*02:10 | 0.0147 |
|  |  |  |  | A*80:01~C*02:02 | 0.0085 |  |  |  |  | A*33:05~C*08:02 | 0.0003 | A*74:01~C*04:01 | 0.0091 |
|  |  |  |  | A*80:01~C*12:03 | 0.0003 |  |  |  |  | A*34:02~C*04:01 | 0.0006 | A*74:01~C*05:01 | 0.0011 |
|  |  |  |  |  |  |  |  |  |  | A*34:02~C*07:01 | 0.0014 | A*74:01~C*06:02 | 0.0020 |
|  |  |  |  |  |  |  |  |  |  | A*34:02~C*16:01 | 0.0003 | A*74:01~C*07:01 | 0.0092 |
|  |  |  |  |  |  |  |  |  |  | A*36:01~C*04:01 | 0.0006 | A*74:01~C*08:02 | 0.0020 |
|  |  |  |  |  |  |  |  |  |  | A*36:01~C*05:01 | 0.0006 | A*74:01~C*08:04 | 0.0029 |
|  |  |  |  |  |  |  |  |  |  | A*36:01~C*16:02 | 0.0002 | A*74:01~C*14:02 | 0.0008 |
|  |  |  |  |  |  |  |  |  |  | A*66:01~C*06:02 | 0.0003 | A*74:01~C*15:05 | 0.0023 |
|  |  |  |  |  |  |  |  |  |  | A*66:01~C*08:02 | 0.0003 | A*74:01~C*16:01 | 0.0054 |
|  |  |  |  |  |  |  |  |  |  | A*66:01~C*12:03 | 0.0011 | A*74:01~C*17:00 | 0.0008 |
|  |  |  |  |  |  |  |  |  |  | A*66:01~C*15:05 | 0.0003 | A*74:01~C*17:01 | 0.0014 |
|  |  |  |  |  |  |  |  |  |  | A*66:01~C*16:02 | 0.0003 | A*74:01~C*18:00 | 0.0043 |
|  |  |  |  |  |  |  |  |  |  | A*66:01~C*17:00 | 0.0006 | A*74:01~C*18:02 | 0.0017 |
|  |  |  |  |  |  |  |  |  |  | A*66:01~C*17:03 | 0.0011 | A*74:03~C*03:02 | 0.0008 |
|  |  |  |  |  |  |  |  |  |  | A*68:01~C*01:02 | 0.0006 | A*74:03~C*07:04 | 0.0008 |
|  |  |  |  |  |  |  |  |  |  | A*68:01~C*02:02 | 0.0014 | A*74:03~C*08:04 | 0.0015 |
|  |  |  |  |  |  |  |  |  |  | A*68:01~C*03:03 | 0.0016 | A*74:03~C*17:00 | 0.0008 |
|  |  |  |  |  |  |  |  |  |  | A*68:01~C*03:04 | 0.0024 | A*74:11~C*02:10 | 0.0008 |
|  |  |  |  |  |  |  |  |  |  | A*68:01~C*04:01 | 0.0054 | A*80:01~C*02:02 | 0.0008 |
|  |  |  |  |  |  |  |  |  |  | A*68:01~C*05:01 | 0.0011 | A*80:01~C*02:10 | 0.0015 |
|  |  |  |  |  |  |  |  |  |  | A*68:01~C*06:02 | 0.0017 | A*80:01~C*04:01 | 0.0025 |
|  |  |  |  |  |  |  |  |  |  | A*68:01~C*07:01 | 0.0011 | A*80:01~C*07:01 | 0.0009 |
|  |  |  |  |  |  |  |  |  |  | A*68:01~C*07:02 | 0.0019 | A*80:01~C*18:00 | 0.0012 |
|  |  |  |  |  |  |  |  |  |  | A*68:01~C*07:04 | 0.0050 |  |  |
|  |  |  |  |  |  |  |  |  |  | A*68:01~C*12:02 | 0.0006 |  |  |
|  |  |  |  |  |  |  |  |  |  | A*68:01~C*12:03 | 0.0014 |  |  |
|  |  |  |  |  |  |  |  |  |  | A*68:01~C*14:02 | 0.0011 |  |  |
|  |  |  |  |  |  |  |  |  |  | A*68:01~C*15:02 | 0.0017 |  |  |
|  |  |  |  |  |  |  |  |  |  | A*68:01~C*16:01 | 0.0006 |  |  |
|  |  |  |  |  |  |  |  |  |  | A*68:02~C*01:02 | 0.0004 |  |  |
|  |  |  |  |  |  |  |  |  |  | A*68:02~C*04:01 | 0.0027 |  |  |
|  |  |  |  |  |  |  |  |  |  | A*68:02~C*05:01 | 0.0007 |  |  |
|  |  |  |  |  |  |  |  |  |  | A*68:02~C*07:01 | 0.0035 |  |  |
|  |  |  |  |  |  |  |  |  |  | A*68:02~C*08:02 | 0.0064 |  |  |
|  |  |  |  |  |  |  |  |  |  | A*68:02~C*12:03 | 0.0004 |  |  |
|  |  |  |  |  |  |  |  |  |  | A*68:02~C*17:00 | 0.0003 |  |  |
|  |  |  |  |  |  |  |  |  |  | A*68:03~C*01:02 | 0.0003 |  |  |
|  |  |  |  |  |  |  |  |  |  | A*68:03~C*03:05 | 0.0003 |  |  |
|  |  |  |  |  |  |  |  |  |  | A*68:03~C*15:02 | 0.0003 |  |  |
|  |  |  |  |  |  |  |  |  |  | A*68:07~C*07:02 | 0.0003 |  |  |
|  |  |  |  |  |  |  |  |  |  | A*68:07~C*15:02 | 0.0003 |  |  |
|  |  |  |  |  |  |  |  |  |  | A*68:15~C*03:04 | 0.0003 |  |  |
|  |  |  |  |  |  |  |  |  |  | A*69:01~C*01:02 | 0.0007 |  |  |
|  |  |  |  |  |  |  |  |  |  | A*69:01~C*03:03 | 0.0003 |  |  |
|  |  |  |  |  |  |  |  |  |  | A*69:01~C*05:01 | 0.0003 |  |  |
|  |  |  |  |  |  |  |  |  |  | A*69:01~C*12:02 | 0.0006 |  |  |
|  |  |  |  |  |  |  |  |  |  | A*69:01~C*12:03 | 0.0005 |  |  |
|  |  |  |  |  |  |  |  |  |  | A*69:01~C*14:02 | 0.0003 |  |  |
|  |  |  |  |  |  |  |  |  |  | A*69:01~C*15:05 | 0.0003 |  |  |
|  |  |  |  |  |  |  |  |  |  | A*74:00~C*02:10 | 0.0003 |  |  |
|  |  |  |  |  |  |  |  |  |  | A*74:00~C*06:02 | 0.0003 |  |  |
|  |  |  |  |  |  |  |  |  |  | A*74:01~C*02:10 | 0.0003 |  |  |
|  |  |  |  |  |  |  |  |  |  | A*74:01~C*16:01 | 0.0003 |  |  |
|  |  |  |  |  |  |  |  |  |  | A*74:03~C*14:02 | 0.0003 |  |  |
|  |  |  |  |  |  |  |  |  |  | A*80:01~C*05:01 | 0.0003 |  |  |

**Supplementary Table 6|** full list of haplotype B~C for all populations

| **KENYA** | | **RWANDA** | | **SOUTH AFRICA** | | **UGANDA** | | **ZAMBIA** | | **EUAM** | | **AFAM** | |
| --- | --- | --- | --- | --- | --- | --- | --- | --- | --- | --- | --- | --- | --- |
| B~C | HF | B~C | HF | B~C | HF | B~C | HF | B~C | HF | B~C | HF | B~C | HF |
| B*07:02~C*07:02 | 0.0688 | B*07:02~C*07:02 | 0.0549 | B*07:02~C*01:02 | 0.0003 | B*07:02~C*07:02 | 0.0541 | B*07:02~C*04:01 | 0.0009 | B*07:02~C*03:04 | 0.0003 | B*07:02~C*02:02 | 0.0008 |
| B*07:02~C*15:02 | 0.0046 | B*07:05~C*07:01 | 0.0029 | B*07:02~C*02:02 | 0.0003 | B*07:02~C*15:02 | 0.0022 | B*07:02~C*06:02 | 0.0009 | B*07:02~C*07:01 | 0.0009 | B*07:02~C*04:01 | 0.0015 |
| B*08:01~C*07:01 | 0.0046 | B*07:05~C*07:02 | 0.0029 | B*07:02~C*02:10 | 0.0037 | B*07:05~C*07:02 | 0.0022 | B*07:02~C*07:02 | 0.0292 | B*07:02~C*07:02 | 0.0929 | B*07:02~C*05:01 | 0.0008 |
| B*13:02~C*06:02 | 0.0092 | B*07:05~C*15:02 | 0.0087 | B*07:02~C*03:04 | 0.0009 | B*07:05~C*15:02 | 0.0022 | B*07:02~C*07:06 | 0.0009 | B*07:02~C*07:04 | 0.0003 | B*07:02~C*07:01 | 0.0023 |
| B*13:02~C*16:04 | 0.0046 | B*08:01~C*03:04 | 0.0058 | B*07:02~C*07:01 | 0.0009 | B*08:01~C*03:04 | 0.0043 | B*07:02~C*15:02 | 0.0018 | B*07:02~C*12:03 | 0.0003 | B*07:02~C*07:02 | 0.0406 |
| B*14:01~C*08:02 | 0.0046 | B*08:01~C*07:01 | 0.0058 | B*07:02~C*07:02 | 0.0490 | B*08:01~C*07:01 | 0.0087 | B*07:05~C*07:01 | 0.0018 | B*07:02~C*15:02 | 0.0003 | B*07:02~C*15:05 | 0.0093 |
| B*14:02~C*08:02 | 0.0321 | B*08:01~C*07:02 | 0.0029 | B*07:02~C*15:05 | 0.0003 | B*08:01~C*07:02 | 0.0043 | B*07:51~C*07:02 | 0.0009 | B*07:02~C*15:05 | 0.0003 | B*07:05~C*07:02 | 0.0055 |
| B*15:03~C*02:10 | 0.0642 | B*08:01~C*07:04 | 0.0029 | B*07:02~C*15:25 | 0.0006 | B*08:01~C*07:04 | 0.0065 | B*08:01~C*03:04 | 0.0035 | B*07:04~C*07:02 | 0.0003 | B*07:05~C*15:05 | 0.0036 |
| B*15:03~C*04:01 | 0.0046 | B*13:02~C*06:02 | 0.0145 | B*07:04~C*07:02 | 0.0003 | B*13:02~C*06:02 | 0.0108 | B*08:01~C*07:01 | 0.0257 | B*07:05~C*02:02 | 0.0003 | B*07:09~C*07:02 | 0.0008 |
| B*15:03~C*08:02 | 0.0138 | B*14:02~C*08:02 | 0.0318 | B*07:05~C*07:01 | 0.0003 | B*14:01~C*08:02 | 0.0022 | B*08:01~C*08:02 | 0.0009 | B*07:05~C*15:05 | 0.0045 | B*08:01~C*01:02 | 0.0015 |
| B*15:10~C*03:04 | 0.0688 | B*14:03~C*08:02 | 0.0029 | B*07:05~C*07:02 | 0.0064 | B*14:02~C*03:04 | 0.0022 | B*13:02~C*03:04 | 0.0009 | B*07:08~C*07:02 | 0.0003 | B*08:01~C*03:04 | 0.0053 |
| B*15:10~C*04:01 | 0.0046 | B*15:03~C*02:10 | 0.0983 | B*07:05~C*08:04 | 0.0003 | B*14:02~C*08:02 | 0.0325 | B*13:02~C*04:01 | 0.0009 | B*08:01~C*03:04 | 0.0006 | B*08:01~C*04:01 | 0.0008 |
| B*15:16~C*14:02 | 0.0138 | B*15:03~C*04:01 | 0.0029 | B*08:01~C*02:05 | 0.0003 | B*14:03~C*08:02 | 0.0022 | B*13:02~C*06:02 | 0.0088 | B*08:01~C*04:01 | 0.0003 | B*08:01~C*05:01 | 0.0008 |
| B*15:17~C*05:01 | 0.0046 | B*15:03~C*07:01 | 0.0029 | B*08:01~C*02:10 | 0.0024 | B*15:03~C*02:10 | 0.0758 | B*13:03~C*06:02 | 0.0009 | B*08:01~C*06:02 | 0.0003 | B*08:01~C*07:01 | 0.0257 |
| B*15:17~C*07:01 | 0.0046 | B*15:03~C*18:01 | 0.0058 | B*08:01~C*02:17 | 0.0067 | B*15:03~C*04:01 | 0.0022 | B*14:01~C*02:10 | 0.0027 | B*08:01~C*07:01 | 0.0717 | B*08:01~C*07:02 | 0.0030 |
| B*15:31~C*04:01 | 0.0046 | B*15:10~C*03:02 | 0.0058 | B*08:01~C*03:04 | 0.0069 | B*15:03~C*06:02 | 0.0021 | B*14:01~C*04:01 | 0.0009 | B*08:01~C*07:02 | 0.0028 | B*13:02~C*05:01 | 0.0015 |
| B*15:31~C*04:07 | 0.0046 | B*15:10~C*03:04 | 0.0289 | B*08:01~C*06:02 | 0.0006 | B*15:03~C*08:02 | 0.0022 | B*14:01~C*07:01 | 0.0008 | B*08:02~C*07:01 | 0.0003 | B*13:02~C*06:02 | 0.0091 |
| B*18:01~C*07:01 | 0.0046 | B*15:16~C*03:04 | 0.0029 | B*08:01~C*07:01 | 0.0281 | B*15:10~C*03:04 | 0.0476 | B*14:01~C*07:02 | 0.0009 | B*13:02~C*04:01 | 0.0003 | B*13:02~C*08:02 | 0.0008 |
| B*18:01~C*07:04 | 0.0138 | B*15:16~C*14:02 | 0.0087 | B*08:01~C*07:02 | 0.0160 | B*15:10~C*04:01 | 0.0022 | B*14:01~C*08:02 | 0.0230 | B*13:02~C*06:02 | 0.0275 | B*13:02~C*08:04 | 0.0008 |
| B*18:03~C*04:01 | 0.0046 | B*15:16~C*16:01 | 0.0029 | B*08:01~C*16:01 | 0.0003 | B*15:10~C*08:04 | 0.0022 | B*14:02~C*08:02 | 0.0327 | B*13:02~C*12:03 | 0.0005 | B*13:02~C*18:00 | 0.0008 |
| B*27:03~C*02:02 | 0.0092 | B*15:17~C*05:01 | 0.0029 | B*08:01~C*17:01 | 0.0003 | B*15:16~C*08:02 | 0.0022 | B*14:03~C*08:02 | 0.0027 | B*14:01~C*08:02 | 0.0122 | B*14:01~C*02:10 | 0.0008 |
| B*35:01~C*04:01 | 0.0229 | B*15:17~C*07:01 | 0.0029 | B*08:01~C*18:00 | 0.0000 | B*15:16~C*14:02 | 0.0087 | B*15:01~C*03:03 | 0.0009 | B*14:02~C*01:02 | 0.0003 | B*14:01~C*06:02 | 0.0008 |
| B*35:01~C*07:04 | 0.0092 | B*15:17~C*17:01 | 0.0029 | B*13:01~C*04:03 | 0.0003 | B*15:17~C*05:01 | 0.0022 | B*15:03~C*02:02 | 0.0027 | B*14:02~C*02:02 | 0.0008 | B*14:01~C*08:02 | 0.0076 |
| B*37:01~C*07:01 | 0.0046 | B*18:01~C*04:01 | 0.0058 | B*13:02~C*06:02 | 0.0162 | B*15:17~C*07:01 | 0.0065 | B*15:03~C*02:10 | 0.0937 | B*14:02~C*08:02 | 0.0439 | B*14:02~C*08:02 | 0.0250 |
| B*39:10~C*12:03 | 0.0046 | B*18:01~C*07:01 | 0.0029 | B*13:03~C*06:02 | 0.0012 | B*15:17~C*17:01 | 0.0043 | B*15:03~C*03:04 | 0.0009 | B*14:02~C*12:03 | 0.0003 | B*14:02~C*08:43 | 0.0008 |
| B*40:12~C*04:01 | 0.0046 | B*18:01~C*07:04 | 0.0087 | B*14:01~C*02:10 | 0.0017 | B*15:31~C*04:01 | 0.0022 | B*15:03~C*04:01 | 0.0018 | B*14:02~C*15:05 | 0.0003 | B*14:02~C*18:00 | 0.0015 |
| B*40:16~C*08:02 | 0.0092 | B*18:01~C*18:01 | 0.0116 | B*14:01~C*08:02 | 0.0055 | B*15:31~C*16:01 | 0.0022 | B*15:03~C*07:01 | 0.0018 | B*14:06~C*08:02 | 0.0003 | B*14:03~C*08:02 | 0.0023 |
| B*41:01~C*07:01 | 0.0092 | B*18:03~C*04:01 | 0.0029 | B*14:01~C*08:04 | 0.0155 | B*15:37~C*03:04 | 0.0022 | B*15:03~C*12:03 | 0.0009 | B*15:01~C*01:02 | 0.0029 | B*15:01~C*01:02 | 0.0015 |
| B*41:01~C*17:01 | 0.0046 | B*27:03~C*02:02 | 0.0087 | B*14:01~C*18:00 | 0.0001 | B*18:01~C*02:02 | 0.0043 | B*15:03~C*16:01 | 0.0009 | B*15:01~C*02:02 | 0.0003 | B*15:01~C*02:02 | 0.0008 |
| B*41:02~C*17:01 | 0.0092 | B*35:01~C*04:01 | 0.0231 | B*14:02~C*08:02 | 0.0125 | B*18:01~C*07:01 | 0.0022 | B*15:03~C*18:01 | 0.0009 | B*15:01~C*03:03 | 0.0241 | B*15:01~C*03:03 | 0.0038 |
| B*42:01~C*17:01 | 0.0734 | B*35:01~C*06:02 | 0.0058 | B*14:06~C*08:02 | 0.0003 | B*18:01~C*07:04 | 0.0281 | B*15:10~C*03:04 | 0.0522 | B*15:01~C*03:04 | 0.0156 | B*15:01~C*03:04 | 0.0030 |
| B*42:02~C*17:01 | 0.0046 | B*35:01~C*15:02 | 0.0029 | B*15:01~C*04:01 | 0.0021 | B*18:03~C*04:01 | 0.0043 | B*15:10~C*08:02 | 0.0018 | B*15:01~C*04:01 | 0.0045 | B*15:01~C*04:01 | 0.0023 |
| B*44:03~C*04:01 | 0.0138 | B*35:02~C*04:01 | 0.0029 | B*15:03~C*02:10 | 0.0699 | B*27:03~C*02:02 | 0.0022 | B*15:10~C*16:01 | 0.0053 | B*15:01~C*07:02 | 0.0003 | B*15:01~C*07:04 | 0.0008 |
| B*44:03~C*07:01 | 0.0046 | B*37:01~C*06:02 | 0.0116 | B*15:03~C*03:03 | 0.0000 | B*27:03~C*04:01 | 0.0022 | B*15:10~C*17:01 | 0.0009 | B*15:01~C*07:04 | 0.0006 | B*15:03~C*02:10 | 0.0461 |
| B*44:03~C*07:02 | 0.0046 | B*39:10~C*12:03 | 0.0116 | B*15:03~C*04:01 | 0.0021 | B*35:01~C*04:01 | 0.0235 | B*15:16~C*14:02 | 0.0053 | B*15:01~C*12:03 | 0.0006 | B*15:03~C*04:01 | 0.0008 |
| B*44:03~C*14:03 | 0.0046 | B*39:10~C*17:01 | 0.0029 | B*15:03~C*04:04 | 0.0003 | B*35:01~C*06:02 | 0.0003 | B*15:16~C*16:01 | 0.0018 | B*15:02~C*08:01 | 0.0006 | B*15:03~C*12:03 | 0.0008 |
| B*44:15~C*04:07 | 0.0092 | B*40:12~C*04:01 | 0.0029 | B*15:03~C*07:01 | 0.0006 | B*35:02~C*16:01 | 0.0022 | B*15:17~C*05:01 | 0.0062 | B*15:03~C*02:10 | 0.0020 | B*15:03~C*18:00 | 0.0015 |
| B*45:01~C*06:02 | 0.0367 | B*40:16~C*08:02 | 0.0058 | B*15:03~C*17:00 | 0.0006 | B*37:01~C*06:02 | 0.0022 | B*15:220~C*04:01 | 0.0009 | B*15:03~C*12:03 | 0.0003 | B*15:07~C*03:03 | 0.0008 |
| B*45:01~C*16:01 | 0.0642 | B*41:01~C*07:01 | 0.0058 | B*15:03~C*17:01 | 0.0003 | B*39:10~C*12:03 | 0.0130 | B*15:220~C*17:01 | 0.0027 | B*15:03~C*16:01 | 0.0006 | B*15:10~C*03:04 | 0.0204 |
| B*47:03~C*03:02 | 0.0046 | B*41:01~C*17:01 | 0.0058 | B*15:03~C*18:00 | 0.0072 | B*40:12~C*04:01 | 0.0065 | B*15:31~C*04:07 | 0.0009 | B*15:07~C*03:03 | 0.0006 | B*15:10~C*04:01 | 0.0023 |
| B*49:01~C*07:01 | 0.0459 | B*42:01~C*03:04 | 0.0029 | B*15:03~C*18:01 | 0.0003 | B*40:16~C*07:01 | 0.0022 | B*15:47~C*02:10 | 0.0027 | B*15:10~C*03:04 | 0.0011 | B*15:10~C*08:04 | 0.0015 |
| B*50:01~C*06:02 | 0.0046 | B*42:01~C*17:01 | 0.0376 | B*15:03~C*18:02 | 0.0018 | B*41:01~C*07:01 | 0.0065 | B*15:83~C*15:25 | 0.0018 | B*15:11~C*03:03 | 0.0003 | B*15:10~C*16:01 | 0.0008 |
| B*51:01~C*16:01 | 0.0046 | B*44:03~C*04:01 | 0.0202 | B*15:08~C*01:02 | 0.0003 | B*41:01~C*17:01 | 0.0022 | B*18:01~C*02:02 | 0.0035 | B*15:16~C*14:02 | 0.0008 | B*15:16~C*14:02 | 0.0151 |
| B*51:01~C*16:02 | 0.0046 | B*44:03~C*07:01 | 0.0116 | B*15:10~C*02:10 | 0.0003 | B*42:01~C*17:01 | 0.0433 | B*18:01~C*02:10 | 0.0044 | B*15:17~C*03:02 | 0.0003 | B*15:16~C*16:01 | 0.0023 |
| B*52:01~C*12:02 | 0.0046 | B*44:03~C*14:03 | 0.0029 | B*15:10~C*03:04 | 0.0326 | B*42:02~C*17:01 | 0.0043 | B*18:01~C*03:04 | 0.0009 | B*15:17~C*05:01 | 0.0003 | B*15:17~C*05:01 | 0.0023 |
| B*53:01~C*04:01 | 0.0505 | B*44:15~C*04:01 | 0.0029 | B*15:10~C*04:01 | 0.0094 | B*44:03~C*04:01 | 0.0130 | B*18:01~C*04:01 | 0.0027 | B*15:17~C*07:01 | 0.0059 | B*15:17~C*07:01 | 0.0015 |
| B*53:01~C*06:02 | 0.0229 | B*44:15~C*04:07 | 0.0347 | B*15:10~C*07:01 | 0.0003 | B*44:03~C*07:01 | 0.0043 | B*18:01~C*05:01 | 0.0027 | B*15:18~C*07:04 | 0.0011 | B*15:18~C*07:01 | 0.0008 |
| B*53:01~C*07:01 | 0.0046 | B*45:01~C*06:02 | 0.0347 | B*15:10~C*08:02 | 0.0006 | B*44:03~C*14:02 | 0.0022 | B*18:01~C*06:02 | 0.0018 | B*15:220~C*12:03 | 0.0006 | B*15:18~C*07:04 | 0.0008 |
| B*53:01~C*16:01 | 0.0092 | B*45:01~C*16:01 | 0.0145 | B*15:10~C*08:04 | 0.0189 | B*44:03~C*14:03 | 0.0022 | B*18:01~C*07:04 | 0.0159 | B*15:24~C*03:03 | 0.0008 | B*15:220~C*07:01 | 0.0008 |
| B*57:02~C*07:01 | 0.0046 | B*47:01~C*06:02 | 0.0058 | B*15:10~C*16:01 | 0.0267 | B*44:15~C*04:07 | 0.0390 | B*18:03~C*04:01 | 0.0009 | B*15:31~C*04:07 | 0.0006 | B*15:31~C*04:27 | 0.0008 |
| B*57:02~C*18:01 | 0.0046 | B*47:01~C*07:01 | 0.0029 | B*15:13~C*08:01 | 0.0006 | B*45:01~C*06:02 | 0.0281 | B*35:01~C*02:10 | 0.0009 | B*15:40~C*03:03 | 0.0003 | B*18:01~C*02:02 | 0.0030 |
| B*57:03~C*04:01 | 0.0046 | B*49:01~C*07:01 | 0.0838 | B*15:16~C*03:04 | 0.0037 | B*45:01~C*07:01 | 0.0022 | B*35:01~C*04:01 | 0.0264 | B*18:01~C*01:02 | 0.0003 | B*18:01~C*02:10 | 0.0030 |
| B*57:03~C*07:01 | 0.0183 | B*51:01~C*14:02 | 0.0029 | B*15:16~C*14:02 | 0.0049 | B*45:01~C*16:01 | 0.0325 | B*35:01~C*06:02 | 0.0037 | B*18:01~C*02:02 | 0.0011 | B*18:01~C*04:01 | 0.0008 |
| B*57:03~C*18:01 | 0.0183 | B*51:01~C*16:01 | 0.0087 | B*15:16~C*16:01 | 0.0046 | B*47:01~C*06:02 | 0.0022 | B*35:01~C*16:01 | 0.0018 | B*18:01~C*02:10 | 0.0003 | B*18:01~C*05:01 | 0.0083 |
| B*58:01~C*03:02 | 0.0138 | B*51:01~C*16:02 | 0.0058 | B*15:17~C*05:01 | 0.0009 | B*47:01~C*07:01 | 0.0043 | B*35:01~C*17:01 | 0.0027 | B*18:01~C*05:01 | 0.0068 | B*18:01~C*07:04 | 0.0023 |
| B*58:01~C*06:02 | 0.0229 | B*53:01~C*04:01 | 0.0578 | B*15:18~C*03:04 | 0.0003 | B*47:03~C*07:01 | 0.0022 | B*35:02~C*04:01 | 0.0009 | B*18:01~C*07:01 | 0.0153 | B*18:01~C*08:02 | 0.0007 |
| B*58:01~C*07:01 | 0.0229 | B*53:01~C*06:02 | 0.0058 | B*15:18~C*07:04 | 0.0003 | B*49:01~C*04:01 | 0.0022 | B*37:01~C*02:10 | 0.0009 | B*18:01~C*12:03 | 0.0190 | B*18:01~C*12:03 | 0.0053 |
| B*58:01~C*17:01 | 0.0092 | B*56:01~C*01:02 | 0.0029 | B*15:18~C*18:00 | 0.0003 | B*49:01~C*06:02 | 0.0022 | B*39:01~C*12:03 | 0.0009 | B*18:05~C*12:03 | 0.0003 | B*18:01~C*18:00 | 0.0008 |
| B*58:02~C*04:01 | 0.0046 | B*57:03~C*07:01 | 0.0231 | B*15:22~C*04:01 | 0.0009 | B*49:01~C*07:01 | 0.0584 | B*39:10~C*12:03 | 0.0115 | B*18:20~C*07:01 | 0.0003 | B*27:02~C*02:02 | 0.0008 |
| B*58:02~C*06:02 | 0.0505 | B*57:03~C*08:02 | 0.0029 | B*15:22~C*17:01 | 0.0003 | B*49:01~C*15:02 | 0.0022 | B*39:10~C*17:01 | 0.0009 | B*27:02~C*02:02 | 0.0057 | B*27:03~C*02:02 | 0.0038 |
| B*73:01~C*15:02 | 0.0092 | B*57:03~C*17:01 | 0.0058 | B*15:220~C*04:01 | 0.0009 | B*51:01~C*16:01 | 0.0130 | B*40:16~C*08:02 | 0.0044 | B*27:02~C*05:01 | 0.0006 | B*27:03~C*07:01 | 0.0008 |
| B*81:01~C*08:02 | 0.0046 | B*57:03~C*18:01 | 0.0029 | B*15:24~C*03:04 | 0.0003 | B*51:01~C*16:02 | 0.0022 | B*40:16~C*17:01 | 0.0018 | B*27:02~C*07:02 | 0.0003 | B*27:05~C*01:02 | 0.0038 |
| B*81:01~C*18:01 | 0.0138 | B*58:01~C*03:02 | 0.0058 | B*18:01~C*02:02 | 0.0119 | B*53:01~C*02:02 | 0.0022 | B*41:01~C*01:02 | 0.0009 | B*27:02~C*15:02 | 0.0003 | B*27:05~C*02:02 | 0.0076 |
|  |  | B*58:01~C*06:02 | 0.0405 | B*18:01~C*02:10 | 0.0003 | B*53:01~C*03:04 | 0.0022 | B*41:01~C*07:01 | 0.0009 | B*27:03~C*02:02 | 0.0003 | B*27:05~C*03:03 | 0.0008 |
|  |  | B*58:01~C*07:01 | 0.0116 | B*18:01~C*04:03 | 0.0003 | B*53:01~C*04:01 | 0.0824 | B*41:01~C*08:02 | 0.0009 | B*27:05~C*01:02 | 0.0241 | B*27:05~C*06:08 | 0.0015 |
|  |  | B*58:01~C*14:02 | 0.0029 | B*18:01~C*05:01 | 0.0037 | B*53:01~C*06:02 | 0.0085 | B*41:01~C*16:01 | 0.0009 | B*27:05~C*01:127 | 0.0006 | B*27:05~C*15:02 | 0.0008 |
|  |  | B*58:02~C*02:10 | 0.0029 | B*18:01~C*07:01 | 0.0003 | B*53:01~C*16:01 | 0.0022 | B*41:01~C*17:01 | 0.0009 | B*27:05~C*02:02 | 0.0220 | B*27:06~C*03:04 | 0.0008 |
|  |  | B*58:02~C*06:02 | 0.1098 | B*18:01~C*07:04 | 0.0159 | B*57:01~C*07:01 | 0.0022 | B*41:02~C*04:07 | 0.0009 | B*27:05~C*03:03 | 0.0017 | B*27:12~C*02:02 | 0.0008 |
|  |  | B*73:01~C*15:02 | 0.0029 | B*18:01~C*12:03 | 0.0003 | B*57:02~C*04:01 | 0.0022 | B*42:01~C*03:04 | 0.0009 | B*27:05~C*04:01 | 0.0002 | B*35:01~C*04:01 | 0.0271 |
|  |  | B*81:01~C*04:01 | 0.0029 | B*18:01~C*15:05 | 0.0024 | B*57:02~C*18:01 | 0.0108 | B*42:01~C*07:01 | 0.0018 | B*27:05~C*05:01 | 0.0006 | B*35:01~C*06:02 | 0.0026 |
|  |  | B*81:01~C*18:01 | 0.0231 | B*18:01~C*17:00 | 0.0003 | B*57:03~C*07:01 | 0.0130 | B*42:01~C*17:01 | 0.0885 | B*27:05~C*07:04 | 0.0006 | B*35:01~C*07:02 | 0.0008 |
|  |  |  |  | B*27:05~C*02:02 | 0.0009 | B*57:03~C*08:02 | 0.0173 | B*42:02~C*17:01 | 0.0097 | B*27:05~C*15:02 | 0.0004 | B*35:01~C*07:05 | 0.0015 |
|  |  |  |  | B*27:05~C*02:10 | 0.0006 | B*57:03~C*17:01 | 0.0043 | B*44:03~C*03:03 | 0.0071 | B*27:07~C*15:02 | 0.0008 | B*35:01~C*14:02 | 0.0008 |
|  |  |  |  | B*35:01~C*04:01 | 0.0174 | B*57:03~C*18:01 | 0.0043 | B*44:03~C*04:01 | 0.0336 | B*27:09~C*18:02 | 0.0003 | B*35:01~C*16:01 | 0.0051 |
|  |  |  |  | B*35:01~C*07:02 | 0.0003 | B*58:01~C*03:02 | 0.0260 | B*44:03~C*07:01 | 0.0151 | B*27:12~C*02:02 | 0.0003 | B*35:01~C*18:00 | 0.0008 |
|  |  |  |  | B*35:02~C*04:01 | 0.0015 | B*58:01~C*04:01 | 0.0022 | B*44:03~C*07:06 | 0.0035 | B*27:13~C*01:02 | 0.0003 | B*35:02~C*04:01 | 0.0015 |
|  |  |  |  | B*35:03~C*02:02 | 0.0003 | B*58:01~C*06:02 | 0.0216 | B*44:03~C*14:02 | 0.0009 | B*35:01~C*01:02 | 0.0003 | B*35:03~C*04:01 | 0.0008 |
|  |  |  |  | B*38:02~C*07:02 | 0.0003 | B*58:01~C*07:01 | 0.0087 | B*44:03~C*14:03 | 0.0035 | B*35:01~C*02:02 | 0.0003 | B*35:03~C*04:29 | 0.0008 |
|  |  |  |  | B*39:10~C*12:03 | 0.0177 | B*58:02~C*04:01 | 0.0022 | B*44:03~C*17:01 | 0.0009 | B*35:01~C*03:03 | 0.0006 | B*35:03~C*12:03 | 0.0008 |
|  |  |  |  | B*39:10~C*15:05 | 0.0027 | B*58:02~C*06:02 | 0.0757 | B*45:01~C*02:10 | 0.0009 | B*35:01~C*03:04 | 0.0006 | B*35:04~C*04:01 | 0.0008 |
|  |  |  |  | B*39:10~C*17:01 | 0.0006 | B*58:02~C*07:01 | 0.0022 | B*45:01~C*06:02 | 0.0318 | B*35:01~C*04:01 | 0.0433 | B*35:05~C*04:01 | 0.0008 |
|  |  |  |  | B*40:01~C*03:04 | 0.0006 | B*73:01~C*15:02 | 0.0022 | B*45:01~C*16:01 | 0.0398 | B*35:01~C*14:02 | 0.0003 | B*35:08~C*04:01 | 0.0008 |
|  |  |  |  | B*40:01~C*12:03 | 0.0003 | B*81:01~C*04:01 | 0.0173 | B*45:01~C*17:01 | 0.0009 | B*35:01~C*16:01 | 0.0003 | B*35:43~C*01:02 | 0.0008 |
|  |  |  |  | B*40:06~C*07:04 | 0.0006 | B*81:01~C*08:02 | 0.0130 | B*45:07~C*16:01 | 0.0009 | B*35:02~C*04:01 | 0.0071 | B*37:01~C*06:02 | 0.0030 |
|  |  |  |  | B*40:06~C*15:02 | 0.0003 | B*81:01~C*18:01 | 0.0130 | B*49:01~C*07:01 | 0.0115 | B*35:02~C*04:08 | 0.0003 | B*37:01~C*07:01 | 0.0008 |
|  |  |  |  | B*41:01~C*02:10 | 0.0000 | B*82:02~C*03:02 | 0.0043 | B*50:01~C*06:02 | 0.0053 | B*35:02~C*06:02 | 0.0003 | B*38:01~C*12:03 | 0.0023 |
|  |  |  |  | B*41:01~C*07:01 | 0.0018 |  |  | B*51:01~C*04:07 | 0.0009 | B*35:02~C*17:03 | 0.0003 | B*39:01~C*02:10 | 0.0008 |
|  |  |  |  | B*41:01~C*17:00 | 0.0027 |  |  | B*51:01~C*14:02 | 0.0035 | B*35:03~C*04:01 | 0.0122 | B*39:01~C*07:01 | 0.0009 |
|  |  |  |  | B*41:01~C*17:01 | 0.0076 |  |  | B*51:01~C*16:01 | 0.0159 | B*35:03~C*12:03 | 0.0040 | B*39:01~C*07:02 | 0.0006 |
|  |  |  |  | B*41:02~C*17:00 | 0.0006 |  |  | B*53:01~C*02:02 | 0.0009 | B*35:05~C*04:01 | 0.0003 | B*39:01~C*12:03 | 0.0015 |
|  |  |  |  | B*41:02~C*17:01 | 0.0006 |  |  | B*53:01~C*03:04 | 0.0044 | B*35:08~C*04:01 | 0.0037 | B*39:01~C*15:02 | 0.0008 |
|  |  |  |  | B*42:01~C*04:01 | 0.0003 |  |  | B*53:01~C*04:01 | 0.0806 | B*35:12~C*03:03 | 0.0003 | B*39:05~C*08:01 | 0.0008 |
|  |  |  |  | B*42:01~C*06:02 | 0.0003 |  |  | B*53:01~C*06:02 | 0.0088 | B*35:12~C*04:01 | 0.0003 | B*39:06~C*07:02 | 0.0008 |
|  |  |  |  | B*42:01~C*17:00 | 0.0436 |  |  | B*53:01~C*06:03 | 0.0009 | B*35:14~C*04:01 | 0.0003 | B*39:10~C*06:02 | 0.0008 |
|  |  |  |  | B*42:01~C*17:01 | 0.0637 |  |  | B*53:01~C*16:01 | 0.0018 | B*35:17~C*04:01 | 0.0003 | B*39:10~C*12:03 | 0.0234 |
|  |  |  |  | B*42:02~C*17:00 | 0.0073 |  |  | B*57:01~C*04:01 | 0.0009 | B*35:187~C*04:01 | 0.0003 | B*39:10~C*14:03 | 0.0008 |
|  |  |  |  | B*42:02~C*17:01 | 0.0088 |  |  | B*57:01~C*06:02 | 0.0009 | B*35:32~C*01:02 | 0.0003 | B*39:20~C*12:03 | 0.0008 |
|  |  |  |  | B*44:02~C*05:01 | 0.0003 |  |  | B*57:02~C*02:02 | 0.0009 | B*35:41~C*04:01 | 0.0003 | B*39:24~C*03:02 | 0.0008 |
|  |  |  |  | B*44:03~C*02:10 | 0.0058 |  |  | B*57:02~C*02:10 | 0.0009 | B*35:42~C*02:10 | 0.0003 | B*40:01~C*03:04 | 0.0121 |
|  |  |  |  | B*44:03~C*03:03 | 0.0052 |  |  | B*57:02~C*18:01 | 0.0053 | B*35:43~C*01:02 | 0.0023 | B*40:01~C*07:02 | 0.0008 |
|  |  |  |  | B*44:03~C*04:01 | 0.0382 |  |  | B*57:02~C*18:02 | 0.0027 | B*37:01~C*04:01 | 0.0003 | B*40:02~C*02:02 | 0.0045 |
|  |  |  |  | B*44:03~C*07:01 | 0.0341 |  |  | B*57:03~C*06:02 | 0.0018 | B*37:01~C*06:02 | 0.0110 | B*40:02~C*03:04 | 0.0008 |
|  |  |  |  | B*44:03~C*07:02 | 0.0003 |  |  | B*57:03~C*07:01 | 0.0053 | B*37:01~C*07:02 | 0.0006 | B*40:02~C*03:05 | 0.0008 |
|  |  |  |  | B*44:03~C*07:06 | 0.0052 |  |  | B*57:03~C*08:02 | 0.0009 | B*38:01~C*01:02 | 0.0003 | B*40:06~C*08:01 | 0.0008 |
|  |  |  |  | B*44:03~C*08:04 | 0.0018 |  |  | B*57:03~C*18:01 | 0.0248 | B*38:01~C*04:01 | 0.0003 | B*40:06~C*15:02 | 0.0015 |
|  |  |  |  | B*44:03~C*14:03 | 0.0005 |  |  | B*57:03~C*18:02 | 0.0204 | B*38:01~C*06:02 | 0.0003 | B*40:12~C*15:05 | 0.0008 |
|  |  |  |  | B*44:03~C*17:01 | 0.0006 |  |  | B*58:01~C*03:02 | 0.0133 | B*38:01~C*07:02 | 0.0006 | B*40:16~C*08:02 | 0.0008 |
|  |  |  |  | B*45:00~C*04:01 | 0.0001 |  |  | B*58:01~C*04:01 | 0.0009 | B*38:01~C*12:03 | 0.0207 | B*41:01~C*06:02 | 0.0008 |
|  |  |  |  | B*45:00~C*16:01 | 0.0005 |  |  | B*58:01~C*06:02 | 0.0097 | B*39:01~C*07:02 | 0.0014 | B*41:01~C*07:01 | 0.0015 |
|  |  |  |  | B*45:01~C*06:02 | 0.0091 |  |  | B*58:01~C*07:01 | 0.0301 | B*39:01~C*12:03 | 0.0088 | B*41:01~C*17:00 | 0.0008 |
|  |  |  |  | B*45:01~C*16:01 | 0.0207 |  |  | B*58:01~C*07:04 | 0.0009 | B*39:01~C*17:03 | 0.0003 | B*41:02~C*12:02 | 0.0008 |
|  |  |  |  | B*45:07~C*16:01 | 0.0046 |  |  | B*58:02~C*06:02 | 0.0513 | B*39:02~C*07:02 | 0.0003 | B*41:02~C*17:00 | 0.0008 |
|  |  |  |  | B*47:01~C*06:02 | 0.0006 |  |  | B*58:02~C*08:02 | 0.0009 | B*39:05~C*12:03 | 0.0003 | B*41:02~C*17:01 | 0.0030 |
|  |  |  |  | B*49:01~C*07:01 | 0.0070 |  |  | B*58:02~C*17 new | 0.0009 | B*39:05~C*18:01 | 0.0003 | B*41:02~C*17:03 | 0.0008 |
|  |  |  |  | B*49:01~C*07:04 | 0.0003 |  |  | B*58:15~C*06:02 | 0.0009 | B*39:06~C*07:02 | 0.0037 | B*41:03~C*17:03 | 0.0008 |
|  |  |  |  | B*50:01~C*06:02 | 0.0003 |  |  | B*73:01~C*15:05 | 0.0027 | B*39:06~C*12:03 | 0.0003 | B*42:01~C*03:02 | 0.0008 |
|  |  |  |  | B*51:01~C*07:01 | 0.0063 |  |  | B*81:01~C*04:01 | 0.0018 | B*39:06~C*16:01 | 0.0003 | B*42:01~C*06:02 | 0.0008 |
|  |  |  |  | B*51:01~C*14:02 | 0.0003 |  |  | B*81:01~C*07:01 | 0.0018 | B*39:08~C*07:02 | 0.0008 | B*42:01~C*07:01 | 0.0008 |
|  |  |  |  | B*51:01~C*16:01 | 0.0012 |  |  | B*81:01~C*08:02 | 0.0009 | B*39:10~C*06:02 | 0.0003 | B*42:01~C*17:00 | 0.0091 |
|  |  |  |  | B*51:01~C*16:04 | 0.0003 |  |  | B*81:01~C*08:04 | 0.0035 | B*39:10~C*12:03 | 0.0008 | B*42:01~C*17:01 | 0.0265 |
|  |  |  |  | B*51:01~C*18:00 | 0.0004 |  |  | B*81:01~C*18:01 | 0.0177 | B*39:11~C*07:02 | 0.0006 | B*42:02~C*17:00 | 0.0023 |
|  |  |  |  | B*52:01~C*12:02 | 0.0003 |  |  | B*82:01~C*04:07 | 0.0009 | B*39:12~C*07:02 | 0.0003 | B*42:02~C*17:01 | 0.0023 |
|  |  |  |  | B*53:01~C*03:04 | 0.0019 |  |  |  |  | B*39:24~C*07:01 | 0.0008 | B*44:02~C*02:02 | 0.0008 |
|  |  |  |  | B*53:01~C*04:01 | 0.0151 |  |  |  |  | B*40:01~C*03:03 | 0.0006 | B*44:02~C*05:01 | 0.0091 |
|  |  |  |  | B*53:01~C*04:226 | 0.0003 |  |  |  |  | B*40:01~C*03:04 | 0.0368 | B*44:02~C*06:02 | 0.0008 |
|  |  |  |  | B*53:01~C*06:02 | 0.0006 |  |  |  |  | B*40:01~C*03:09 | 0.0003 | B*44:02~C*07:04 | 0.0015 |
|  |  |  |  | B*53:01~C*08:02 | 0.0003 |  |  |  |  | B*40:01~C*05:01 | 0.0003 | B*44:03~C*03:03 | 0.0008 |
|  |  |  |  | B*53:01~C*14:03 | 0.0001 |  |  |  |  | B*40:01~C*07:02 | 0.0006 | B*44:03~C*04:01 | 0.0281 |
|  |  |  |  | B*55:01~C*01:02 | 0.0006 |  |  |  |  | B*40:02~C*02:02 | 0.0147 | B*44:03~C*07:01 | 0.0059 |
|  |  |  |  | B*57:01~C*06:02 | 0.0003 |  |  |  |  | B*40:02~C*03:04 | 0.0017 | B*44:03~C*07:02 | 0.0009 |
|  |  |  |  | B*57:02~C*07:01 | 0.0028 |  |  |  |  | B*40:02~C*03:05 | 0.0003 | B*44:03~C*07:06 | 0.0015 |
|  |  |  |  | B*57:02~C*18:00 | 0.0034 |  |  |  |  | B*40:02~C*03:06 | 0.0003 | B*44:03~C*14:03 | 0.0045 |
|  |  |  |  | B*57:02~C*18:02 | 0.0018 |  |  |  |  | B*40:02~C*04:01 | 0.0006 | B*44:03~C*16:01 | 0.0060 |
|  |  |  |  | B*57:03~C*07:01 | 0.0177 |  |  |  |  | B*40:02~C*15:02 | 0.0014 | B*44:03~C*18:00 | 0.0008 |
|  |  |  |  | B*57:03~C*08:02 | 0.0006 |  |  |  |  | B*40:04~C*03:04 | 0.0006 | B*44:05~C*02:02 | 0.0008 |
|  |  |  |  | B*57:03~C*18:00 | 0.0020 |  |  |  |  | B*40:06~C*08:01 | 0.0003 | B*44:10~C*04:01 | 0.0015 |
|  |  |  |  | B*57:03~C*18:01 | 0.0006 |  |  |  |  | B*40:08~C*03:04 | 0.0006 | B*44:15~C*04:07 | 0.0015 |
|  |  |  |  | B*57:03~C*18:02 | 0.0019 |  |  |  |  | B*40:16~C*08:02 | 0.0003 | B*45:01~C*04:07 | 0.0015 |
|  |  |  |  | B*58:01~C*02:10 | 0.0003 |  |  |  |  | B*40:31~C*03:04 | 0.0003 | B*45:01~C*06:02 | 0.0083 |
|  |  |  |  | B*58:01~C*03:02 | 0.0122 |  |  |  |  | B*41:01~C*06:02 | 0.0006 | B*45:01~C*16:01 | 0.0204 |
|  |  |  |  | B*58:01~C*03:04 | 0.0016 |  |  |  |  | B*41:01~C*07:01 | 0.0006 | B*47:01~C*06:02 | 0.0008 |
|  |  |  |  | B*58:01~C*04:01 | 0.0006 |  |  |  |  | B*41:01~C*16:02 | 0.0003 | B*47:01~C*18:02 | 0.0008 |
|  |  |  |  | B*58:01~C*06:02 | 0.0101 |  |  |  |  | B*41:01~C*17:00 | 0.0020 | B*48:01~C*12:03 | 0.0008 |
|  |  |  |  | B*58:01~C*07:01 | 0.0246 |  |  |  |  | B*41:01~C*17:01 | 0.0028 | B*49:01~C*03:04 | 0.0008 |
|  |  |  |  | B*58:01~C*07:18 | 0.0006 |  |  |  |  | B*41:02~C*17:00 | 0.0011 | B*49:01~C*07:01 | 0.0250 |
|  |  |  |  | B*58:01~C*18:00 | 0.0003 |  |  |  |  | B*41:02~C*17:03 | 0.0025 | B*49:01~C*08:02 | 0.0008 |
|  |  |  |  | B*58:02~C*04:01 | 0.0003 |  |  |  |  | B*42:01~C*17:00 | 0.0003 | B*50:01~C*04:01 | 0.0015 |
|  |  |  |  | B*58:02~C*06:02 | 0.1134 |  |  |  |  | B*42:01~C*17:01 | 0.0008 | B*50:01~C*06:02 | 0.0038 |
|  |  |  |  | B*58:02~C*07:01 | 0.0003 |  |  |  |  | B*42:02~C*17:01 | 0.0003 | B*51:01~C*01:02 | 0.0008 |
|  |  |  |  | B*58:02~C*18:00 | 0.0003 |  |  |  |  | B*44:02~C*01:02 | 0.0003 | B*51:01~C*02:02 | 0.0008 |
|  |  |  |  | B*58:10~C*03:02 | 0.0003 |  |  |  |  | B*44:02~C*02:02 | 0.0006 | B*51:01~C*03:04 | 0.0008 |
|  |  |  |  | B*81:00~C*04:01 | 0.0015 |  |  |  |  | B*44:02~C*03:03 | 0.0006 | B*51:01~C*06:02 | 0.0008 |
|  |  |  |  | B*81:00~C*08:04 | 0.0006 |  |  |  |  | B*44:02~C*04:01 | 0.0003 | B*51:01~C*08:01 | 0.0008 |
|  |  |  |  | B*81:00~C*18:00 | 0.0037 |  |  |  |  | B*44:02~C*05:01 | 0.0657 | B*51:01~C*14:02 | 0.0030 |
|  |  |  |  | B*81:01~C*04:01 | 0.0140 |  |  |  |  | B*44:02~C*07:04 | 0.0062 | B*51:01~C*15:02 | 0.0061 |
|  |  |  |  | B*81:01~C*06:02 | 0.0003 |  |  |  |  | B*44:02~C*12:03 | 0.0003 | B*51:01~C*16:01 | 0.0106 |
|  |  |  |  | B*81:01~C*08:04 | 0.0028 |  |  |  |  | B*44:02~C*15:02 | 0.0003 | B*51:01~C*16:02 | 0.0008 |
|  |  |  |  | B*81:01~C*18:00 | 0.0186 |  |  |  |  | B*44:02~C*16:04 | 0.0011 | B*51:01~C*18:02 | 0.0008 |
|  |  |  |  | B*81:01~C*18:01 | 0.0061 |  |  |  |  | B*44:03~C*02:02 | 0.0013 | B*51:02~C*16:01 | 0.0008 |
|  |  |  |  | B*81:03~C*04:01 | 0.0003 |  |  |  |  | B*44:03~C*03:04 | 0.0006 | B*51:07~C*14:02 | 0.0008 |
|  |  |  |  | B*81:03~C*08:04 | 0.0003 |  |  |  |  | B*44:03~C*04:01 | 0.0113 | B*51:08~C*16:02 | 0.0008 |
|  |  |  |  | B*82:02~C*03:02 | 0.0049 |  |  |  |  | B*44:03~C*06:02 | 0.0003 | B*51:64~C*16:01 | 0.0008 |
|  |  |  |  |  |  |  |  |  |  | B*44:03~C*07:01 | 0.0009 | B*52:01~C*04:01 | 0.0008 |
|  |  |  |  |  |  |  |  |  |  | B*44:03~C*07:02 | 0.0006 | B*52:01~C*12:02 | 0.0023 |
|  |  |  |  |  |  |  |  |  |  | B*44:03~C*07:06 | 0.0003 | B*52:01~C*15:02 | 0.0023 |
|  |  |  |  |  |  |  |  |  |  | B*44:03~C*12:03 | 0.0003 | B*52:01~C*16:01 | 0.0144 |
|  |  |  |  |  |  |  |  |  |  | B*44:03~C*16:01 | 0.0310 | B*53:01~C*03:03 | 0.0008 |
|  |  |  |  |  |  |  |  |  |  | B*44:03~C*16:02 | 0.0009 | B*53:01~C*04:01 | 0.0763 |
|  |  |  |  |  |  |  |  |  |  | B*44:04~C*16:01 | 0.0008 | B*53:01~C*04:13 | 0.0015 |
|  |  |  |  |  |  |  |  |  |  | B*44:05~C*02:02 | 0.0017 | B*53:01~C*06:02 | 0.0080 |
|  |  |  |  |  |  |  |  |  |  | B*44:05~C*03:05 | 0.0002 | B*53:01~C*07:01 | 0.0008 |
|  |  |  |  |  |  |  |  |  |  | B*44:27~C*07:04 | 0.0003 | B*53:01~C*08:02 | 0.0023 |
|  |  |  |  |  |  |  |  |  |  | B*45:01~C*02:02 | 0.0002 | B*53:01~C*16:01 | 0.0026 |
|  |  |  |  |  |  |  |  |  |  | B*45:01~C*06:02 | 0.0045 | B*53:01~C*17:01 | 0.0008 |
|  |  |  |  |  |  |  |  |  |  | B*45:01~C*16:01 | 0.0030 | B*55:01~C*03:03 | 0.0015 |
|  |  |  |  |  |  |  |  |  |  | B*46:01~C*01:02 | 0.0003 | B*56:01~C*01:02 | 0.0015 |
|  |  |  |  |  |  |  |  |  |  | B*47:01~C*03:04 | 0.0003 | B*57:01~C*06:02 | 0.0098 |
|  |  |  |  |  |  |  |  |  |  | B*47:01~C*06:02 | 0.0008 | B*57:01~C*07:01 | 0.0008 |
|  |  |  |  |  |  |  |  |  |  | B*48:01~C*08:01 | 0.0017 | B*57:02~C*08:02 | 0.0015 |
|  |  |  |  |  |  |  |  |  |  | B*48:01~C*08:03 | 0.0006 | B*57:02~C*18:00 | 0.0076 |
|  |  |  |  |  |  |  |  |  |  | B*48:02~C*04:01 | 0.0006 | B*57:02~C*18:02 | 0.0038 |
|  |  |  |  |  |  |  |  |  |  | B*49:01~C*06:02 | 0.0003 | B*57:03~C*04:01 | 0.0008 |
|  |  |  |  |  |  |  |  |  |  | B*49:01~C*07:01 | 0.0136 | B*57:03~C*07:01 | 0.0560 |
|  |  |  |  |  |  |  |  |  |  | B*49:01~C*07:02 | 0.0006 | B*57:03~C*07:621 | 0.0008 |
|  |  |  |  |  |  |  |  |  |  | B*50:01~C*04:01 | 0.0003 | B*57:03~C*08:02 | 0.0045 |
|  |  |  |  |  |  |  |  |  |  | B*50:01~C*05:01 | 0.0006 | B*57:03~C*17:01 | 0.0015 |
|  |  |  |  |  |  |  |  |  |  | B*50:01~C*06:02 | 0.0057 | B*57:03~C*18:00 | 0.0250 |
|  |  |  |  |  |  |  |  |  |  | B*50:02~C*06:02 | 0.0003 | B*57:03~C*18:02 | 0.0197 |
|  |  |  |  |  |  |  |  |  |  | B*50:02~C*16:02 | 0.0003 | B*58:01~C*03:02 | 0.0166 |
|  |  |  |  |  |  |  |  |  |  | B*51:01~C*01:02 | 0.0062 | B*58:01~C*06:02 | 0.0015 |
|  |  |  |  |  |  |  |  |  |  | B*51:01~C*02:02 | 0.0028 | B*58:01~C*07:01 | 0.0227 |
|  |  |  |  |  |  |  |  |  |  | B*51:01~C*02:29 | 0.0003 | B*58:01~C*16:01 | 0.0008 |
|  |  |  |  |  |  |  |  |  |  | B*51:01~C*03:04 | 0.0006 | B*58:02~C*04:01 | 0.0008 |
|  |  |  |  |  |  |  |  |  |  | B*51:01~C*04:01 | 0.0024 | B*58:02~C*06:02 | 0.0227 |
|  |  |  |  |  |  |  |  |  |  | B*51:01~C*05:01 | 0.0006 | B*58:11~C*03:02 | 0.0008 |
|  |  |  |  |  |  |  |  |  |  | B*51:01~C*07:02 | 0.0003 | B*58:11~C*07:01 | 0.0008 |
|  |  |  |  |  |  |  |  |  |  | B*51:01~C*12:03 | 0.0003 | B*73:01~C*15:05 | 0.0008 |
|  |  |  |  |  |  |  |  |  |  | B*51:01~C*14:02 | 0.0147 | B*78:01~C*07:04 | 0.0008 |
|  |  |  |  |  |  |  |  |  |  | B*51:01~C*15:02 | 0.0215 | B*78:01~C*16:01 | 0.0053 |
|  |  |  |  |  |  |  |  |  |  | B*51:01~C*15:06 | 0.0003 | B*81:01~C*07:04 | 0.0008 |
|  |  |  |  |  |  |  |  |  |  | B*51:01~C*15:13 | 0.0006 | B*81:01~C*08:04 | 0.0265 |
|  |  |  |  |  |  |  |  |  |  | B*51:01~C*16:01 | 0.0006 | B*81:01~C*08:13 | 0.0008 |
|  |  |  |  |  |  |  |  |  |  | B*51:01~C*16:02 | 0.0017 | B*81:01~C*18:00 | 0.0159 |
|  |  |  |  |  |  |  |  |  |  | B*51:05~C*04:01 | 0.0003 | B*81:01~C*18:01 | 0.0083 |
|  |  |  |  |  |  |  |  |  |  | B*51:07~C*14:02 | 0.0008 | B*82:01~C*03:02 | 0.0015 |
|  |  |  |  |  |  |  |  |  |  | B*51:08~C*16:02 | 0.0003 | B*82:02~C*03:02 | 0.0008 |
|  |  |  |  |  |  |  |  |  |  | B*51:09~C*01:02 | 0.0011 |  |  |
|  |  |  |  |  |  |  |  |  |  | B*51:22~C*15:02 | 0.0003 |  |  |
|  |  |  |  |  |  |  |  |  |  | B*52:01~C*02:02 | 0.0003 |  |  |
|  |  |  |  |  |  |  |  |  |  | B*52:01~C*03:04 | 0.0006 |  |  |
|  |  |  |  |  |  |  |  |  |  | B*52:01~C*12:02 | 0.0167 |  |  |
|  |  |  |  |  |  |  |  |  |  | B*52:01~C*15:02 | 0.0008 |  |  |
|  |  |  |  |  |  |  |  |  |  | B*52:01~C*16:01 | 0.0006 |  |  |
|  |  |  |  |  |  |  |  |  |  | B*52:01~C*16:04 | 0.0003 |  |  |
|  |  |  |  |  |  |  |  |  |  | B*52:02~C*15:02 | 0.0003 |  |  |
|  |  |  |  |  |  |  |  |  |  | B*53:01~C*01:02 | 0.0003 |  |  |
|  |  |  |  |  |  |  |  |  |  | B*53:01~C*04:01 | 0.0080 |  |  |
|  |  |  |  |  |  |  |  |  |  | B*53:01~C*06:02 | 0.0008 |  |  |
|  |  |  |  |  |  |  |  |  |  | B*53:01~C*12:03 | 0.0003 |  |  |
|  |  |  |  |  |  |  |  |  |  | B*53:01~C*15:05 | 0.0003 |  |  |
|  |  |  |  |  |  |  |  |  |  | B*54:01~C*01:02 | 0.0003 |  |  |
|  |  |  |  |  |  |  |  |  |  | B*55:01~C*01:02 | 0.0023 |  |  |
|  |  |  |  |  |  |  |  |  |  | B*55:01~C*03:03 | 0.0088 |  |  |
|  |  |  |  |  |  |  |  |  |  | B*55:01~C*03:04 | 0.0003 |  |  |
|  |  |  |  |  |  |  |  |  |  | B*55:01~C*03:05 | 0.0003 |  |  |
|  |  |  |  |  |  |  |  |  |  | B*55:01~C*12:03 | 0.0003 |  |  |
|  |  |  |  |  |  |  |  |  |  | B*56:01~C*01:02 | 0.0051 |  |  |
|  |  |  |  |  |  |  |  |  |  | B*57:01~C*01:02 | 0.0006 |  |  |
|  |  |  |  |  |  |  |  |  |  | B*57:01~C*03:04 | 0.0003 |  |  |
|  |  |  |  |  |  |  |  |  |  | B*57:01~C*06:02 | 0.0742 |  |  |
|  |  |  |  |  |  |  |  |  |  | B*57:01~C*07:01 | 0.0031 |  |  |
|  |  |  |  |  |  |  |  |  |  | B*57:01~C*07:02 | 0.0003 |  |  |
|  |  |  |  |  |  |  |  |  |  | B*57:01~C*12:03 | 0.0003 |  |  |
|  |  |  |  |  |  |  |  |  |  | B*57:01~C*15:02 | 0.0003 |  |  |
|  |  |  |  |  |  |  |  |  |  | B*57:01~C*16:01 | 0.0003 |  |  |
|  |  |  |  |  |  |  |  |  |  | B*57:02~C*01:02 | 0.0003 |  |  |
|  |  |  |  |  |  |  |  |  |  | B*57:02~C*04:01 | 0.0003 |  |  |
|  |  |  |  |  |  |  |  |  |  | B*57:02~C*07:01 | 0.0006 |  |  |
|  |  |  |  |  |  |  |  |  |  | B*57:02~C*18:00 | 0.0003 |  |  |
|  |  |  |  |  |  |  |  |  |  | B*57:03~C*06:02 | 0.0003 |  |  |
|  |  |  |  |  |  |  |  |  |  | B*57:03~C*07:01 | 0.0074 |  |  |
|  |  |  |  |  |  |  |  |  |  | B*57:03~C*08:02 | 0.0003 |  |  |
|  |  |  |  |  |  |  |  |  |  | B*57:03~C*12:02 | 0.0003 |  |  |
|  |  |  |  |  |  |  |  |  |  | B*57:03~C*18:00 | 0.0020 |  |  |
|  |  |  |  |  |  |  |  |  |  | B*57:03~C*18:02 | 0.0003 |  |  |
|  |  |  |  |  |  |  |  |  |  | B*58:01~C*03:02 | 0.0031 |  |  |
|  |  |  |  |  |  |  |  |  |  | B*58:01~C*03:03 | 0.0003 |  |  |
|  |  |  |  |  |  |  |  |  |  | B*58:01~C*06:02 | 0.0006 |  |  |
|  |  |  |  |  |  |  |  |  |  | B*58:01~C*07:01 | 0.0088 |  |  |
|  |  |  |  |  |  |  |  |  |  | B*58:01~C*12:03 | 0.0003 |  |  |
|  |  |  |  |  |  |  |  |  |  | B*58:01~C*14:02 | 0.0003 |  |  |
|  |  |  |  |  |  |  |  |  |  | B*58:02~C*06:02 | 0.0006 |  |  |
|  |  |  |  |  |  |  |  |  |  | B*67:01~C*12:03 | 0.0003 |  |  |
|  |  |  |  |  |  |  |  |  |  | B*73:01~C*15:05 | 0.0006 |  |  |
|  |  |  |  |  |  |  |  |  |  | B*81:01~C*07:02 | 0.0003 |  |  |
|  |  |  |  |  |  |  |  |  |  | B*81:01~C*08:04 | 0.0003 |  |  |

**Supplementary Table 7|** full list of haplotype A~B~C for all populations

| **KENYA** | | **RWANDA** | | **SOUTH AFRICA** | | **UGANDA** | | **ZAMBIA** | | **EUAM** | | **AFAM** | |
| --- | --- | --- | --- | --- | --- | --- | --- | --- | --- | --- | --- | --- | --- |
| A~B~C | HF | A~B~C | HF | A~B~C | HF | A~B~C | HF | A~B~C | HF | A~B~C | HF | A~B~C | HF |
| A*01:01~B*07:02~C*07:02 | 0.0092 | A*01:01~B*07:05~C*07:01 | 0.0029 | A*01:01~B*07:02~C*07:02 | 0.0006 | A*01:01~B*07:05~C*07:02 | 0.0022 | A*01:01~B*08:01~C*07:01 | 0.0009 | A*01:01~B*07:02~C*07:01 | 0.0009 | A*01:01~B*08:01~C*01:02 | 0.0008 |
| A*01:01~B*15:16~C*14:02 | 0.0046 | A*01:01~B*07:05~C*15:02 | 0.0087 | A*01:01~B*08:01~C*07:01 | 0.0006 | A*01:01~B*07:05~C*15:02 | 0.0022 | A*01:01~B*15:10~C*03:04 | 0.0009 | A*01:01~B*07:02~C*07:02 | 0.0071 | A*01:01~B*08:01~C*07:01 | 0.0143 |
| A*01:01~B*35:01~C*07:04 | 0.0092 | A*01:01~B*37:01~C*06:02 | 0.0116 | A*01:01~B*15:03~C*04:01 | 0.0003 | A*01:01~B*08:01~C*07:02 | 0.0022 | A*01:01~B*39:10~C*12:03 | 0.0009 | A*01:01~B*07:05~C*15:05 | 0.0003 | A*01:01~B*15:10~C*03:04 | 0.0023 |
| A*01:01~B*41:01~C*07:01 | 0.0092 | A*01:01~B*41:01~C*07:01 | 0.0029 | A*01:01~B*15:08~C*01:02 | 0.0003 | A*01:01~B*15:03~C*06:02 | 0.0022 | A*01:01~B*49:01~C*07:01 | 0.0018 | A*01:01~B*08:01~C*07:01 | 0.0503 | A*01:01~B*15:10~C*04:01 | 0.0015 |
| A*01:01~B*44:15~C*04:01 | 0.0046 | A*01:01~B*44:03~C*04:01 | 0.0029 | A*01:01~B*15:10~C*04:01 | 0.0003 | A*01:01~B*18:03~C*04:01 | 0.0022 | A*01:01~B*53:01~C*04:01 | 0.0009 | A*01:01~B*13:02~C*06:02 | 0.0008 | A*01:01~B*37:01~C*06:02 | 0.0015 |
| A*01:01~B*45:01~C*06:02 | 0.0046 | A*01:01~B*44:15~C*04:01 | 0.0029 | A*01:01~B*15:16~C*08:04 | 0.0003 | A*01:01~B*37:01~C*06:02 | 0.0022 | A*01:01~B*53:01~C*06:02 | 0.0009 | A*01:01~B*13:02~C*07:01 | 0.0003 | A*01:01~B*38:01~C*12:03 | 0.0015 |
| A*01:01~B*49:01~C*07:01 | 0.0092 | A*01:01~B*44:15~C*04:07 | 0.0260 | A*01:01~B*15:22~C*04:01 | 0.0003 | A*01:01~B*42:01~C*17:01 | 0.0022 | A*01:01~B*57:01~C*06:02 | 0.0009 | A*01:01~B*14:02~C*08:02 | 0.0018 | A*01:01~B*39:10~C*12:03 | 0.0008 |
| A*01:01~B*58:01~C*07:01 | 0.0046 | A*01:01~B*45:01~C*06:02 | 0.0196 | A*01:01~B*15:220~C*04:01 | 0.0003 | A*01:01~B*44:15~C*04:07 | 0.0346 | A*01:01~B*81:01~C*08:02 | 0.0009 | A*01:01~B*15:01~C*01:02 | 0.0003 | A*01:01~B*40:06~C*15:02 | 0.0008 |
| A*01:01~B*81:01~C*18:01 | 0.0092 | A*01:01~B*49:01~C*07:01 | 0.0068 | A*01:01~B*47:01~C*06:02 | 0.0006 | A*01:01~B*45:01~C*06:02 | 0.0043 | A*01:01~B*81:01~C*18:01 | 0.0080 | A*01:01~B*15:01~C*03:04 | 0.0005 | A*01:01~B*41:02~C*12:02 | 0.0008 |
| A*01:03~B*41:02~C*17:01 | 0.0046 | A*01:01~B*57:03~C*07:01 | 0.0029 | A*01:01~B*51:01~C*16:01 | 0.0003 | A*01:01~B*51:01~C*16:01 | 0.0022 | A*01:02~B*35:01~C*06:02 | 0.0009 | A*01:01~B*15:01~C*04:01 | 0.0003 | A*01:01~B*44:03~C*07:06 | 0.0008 |
| A*01:03~B*53:01~C*06:02 | 0.0046 | A*01:01~B*58:01~C*03:02 | 0.0029 | A*01:01~B*57:03~C*07:01 | 0.0003 | A*01:01~B*57:02~C*18:01 | 0.0022 | A*01:03~B*15:03~C*18:01 | 0.0009 | A*01:01~B*15:17~C*07:01 | 0.0015 | A*01:01~B*45:01~C*06:02 | 0.0015 |
| A*02:01~B*07:02~C*07:02 | 0.0046 | A*01:01~B*58:01~C*06:02 | 0.0029 | A*01:01~B*58:01~C*07:01 | 0.0003 | A*01:01~B*57:03~C*08:02 | 0.0022 | A*01:03~B*15:16~C*14:02 | 0.0009 | A*01:01~B*18:01~C*05:01 | 0.0003 | A*01:01~B*51:01~C*12:02 | 0.0008 |
| A*02:01~B*15:03~C*02:10 | 0.0229 | A*01:01~B*58:02~C*06:02 | 0.0083 | A*01:01~B*58:02~C*06:02 | 0.0021 | A*01:01~B*58:01~C*07:01 | 0.0022 | A*01:03~B*51:01~C*16:01 | 0.0009 | A*01:01~B*18:01~C*07:01 | 0.0012 | A*01:01~B*51:01~C*15:02 | 0.0008 |
| A*02:01~B*15:03~C*03:04 | 0.0046 | A*01:01~B*81:01~C*18:01 | 0.0145 | A*01:01~B*81:00~C*18:00 | 0.0030 | A*01:01~B*81:01~C*18:01 | 0.0087 | A*02:01~B*14:01~C*02:10 | 0.0009 | A*01:01~B*27:02~C*02:02 | 0.0006 | A*01:01~B*53:01~C*06:02 | 0.0008 |
| A*02:01~B*18:01~C*07:04 | 0.0138 | A*01:02~B*58:01~C*03:02 | 0.0029 | A*01:01~B*81:01~C*18:00 | 0.0168 | A*01:02~B*44:03~C*07:01 | 0.0022 | A*02:01~B*14:02~C*08:02 | 0.0019 | A*01:01~B*27:05~C*01:02 | 0.0008 | A*01:01~B*57:01~C*06:02 | 0.0038 |
| A*02:01~B*40:12~C*04:07 | 0.0046 | A*01:03~B*07:02~C*07:02 | 0.0038 | A*01:01~B*81:01~C*18:01 | 0.0052 | A*01:02~B*49:01~C*04:01 | 0.0022 | A*02:01~B*15:03~C*02:10 | 0.0228 | A*01:01~B*27:05~C*02:02 | 0.0013 | A*01:01~B*57:03~C*07:01 | 0.0038 |
| A*02:01~B*40:16~C*08:02 | 0.0092 | A*01:03~B*49:01~C*07:01 | 0.0029 | A*01:02~B*81:01~C*18:00 | 0.0003 | A*01:02~B*53:01~C*04:01 | 0.0022 | A*02:01~B*15:10~C*03:04 | 0.0027 | A*01:01~B*35:01~C*04:01 | 0.0010 | A*01:01~B*58:02~C*06:02 | 0.0015 |
| A*02:01~B*41:02~C*17:01 | 0.0046 | A*01:03~B*58:01~C*07:01 | 0.0019 | A*01:23~B*07:05~C*07:02 | 0.0003 | A*01:02~B*58:01~C*03:02 | 0.0043 | A*02:01~B*18:01~C*07:04 | 0.0019 | A*01:01~B*35:02~C*04:01 | 0.0017 | A*01:01~B*81:01~C*18:01 | 0.0023 |
| A*02:01~B*45:01~C*16:01 | 0.0321 | A*01:09~B*57:03~C*17:01 | 0.0029 | A*01:23~B*58:01~C*06:02 | 0.0018 | A*01:03~B*57:02~C*18:01 | 0.0043 | A*02:01~B*42:01~C*17:01 | 0.0046 | A*01:01~B*35:02~C*06:02 | 0.0003 | A*01:01~B*82:01~C*03:02 | 0.0008 |
| A*02:01~B*49:01~C*07:01 | 0.0092 | A*02:01~B*07:02~C*07:02 | 0.0087 | A*01:23~B*58:02~C*06:02 | 0.0003 | A*02:01~B*15:03~C*02:10 | 0.0405 | A*02:01~B*42:02~C*17:01 | 0.0017 | A*01:01~B*35:03~C*04:01 | 0.0012 | A*01:02~B*07:02~C*15:05 | 0.0008 |
| A*02:01~B*53:01~C*04:01 | 0.0046 | A*02:01~B*14:02~C*08:02 | 0.0145 | A*02:01~B*08:01~C*02:10 | 0.0003 | A*02:01~B*15:17~C*17:01 | 0.0022 | A*02:01~B*45:01~C*06:02 | 0.0014 | A*01:01~B*35:03~C*12:03 | 0.0004 | A*01:02~B*49:01~C*07:01 | 0.0015 |
| A*02:01~B*58:01~C*03:02 | 0.0092 | A*02:01~B*15:03~C*02:10 | 0.0484 | A*02:01~B*08:01~C*07:01 | 0.0030 | A*02:01~B*15:31~C*16:01 | 0.0022 | A*02:01~B*45:01~C*16:01 | 0.0224 | A*01:01~B*35:08~C*04:01 | 0.0003 | A*01:02~B*58:01~C*03:02 | 0.0008 |
| A*02:01~B*58:02~C*06:02 | 0.0046 | A*02:01~B*15:03~C*04:01 | 0.0029 | A*02:01~B*15:03~C*02:10 | 0.0033 | A*02:01~B*18:01~C*07:04 | 0.0022 | A*02:01~B*49:01~C*03:02 | 0.0009 | A*01:01~B*37:01~C*06:02 | 0.0062 | A*01:02~B*58:01~C*18:00 | 0.0008 |
| A*02:02~B*15:03~C*02:10 | 0.0092 | A*02:01~B*15:03~C*07:01 | 0.0029 | A*02:01~B*15:03~C*04:01 | 0.0003 | A*02:01~B*35:01~C*04:01 | 0.0043 | A*02:01~B*49:01~C*07:01 | 0.0044 | A*01:01~B*38:01~C*06:02 | 0.0003 | A*01:03~B*41:02~C*17:01 | 0.0008 |
| A*02:02~B*41:01~C*17:01 | 0.0046 | A*02:01~B*15:10~C*03:04 | 0.0029 | A*02:01~B*15:10~C*03:04 | 0.0003 | A*02:01~B*35:02~C*16:01 | 0.0022 | A*02:01~B*51:01~C*16:01 | 0.0070 | A*01:01~B*38:01~C*07:02 | 0.0003 | A*01:09~B*15:03~C*18:00 | 0.0008 |
| A*02:02~B*53:01~C*04:01 | 0.0046 | A*02:01~B*18:01~C*07:01 | 0.0029 | A*02:01~B*15:10~C*08:04 | 0.0026 | A*02:01~B*40:12~C*04:01 | 0.0043 | A*02:01~B*53:01~C*04:01 | 0.0080 | A*01:01~B*38:01~C*12:03 | 0.0004 | A*01:09~B*44:15~C*04:07 | 0.0008 |
| A*02:02~B*53:01~C*06:02 | 0.0092 | A*02:01~B*35:01~C*04:01 | 0.0058 | A*02:01~B*15:10~C*16:01 | 0.0006 | A*02:01~B*40:16~C*08:02 | 0.0022 | A*02:01~B*53:01~C*06:02 | 0.0010 | A*01:01~B*39:06~C*07:02 | 0.0008 | A*01:22N~B*52:01~C*12:02 | 0.0008 |
| A*02:02~B*57:03~C*07:01 | 0.0092 | A*02:01~B*45:01~C*16:01 | 0.0087 | A*02:01~B*15:13~C*08:01 | 0.0003 | A*02:01~B*41:01~C*07:01 | 0.0043 | A*02:01~B*57:02~C*02:02 | 0.0009 | A*01:01~B*40:01~C*03:04 | 0.0027 | A*02:01~B*07:02~C*02:02 | 0.0008 |
| A*02:02~B*58:01~C*17:01 | 0.0092 | A*02:01~B*47:01~C*07:01 | 0.0029 | A*02:01~B*15:16~C*16:01 | 0.0046 | A*02:01~B*45:01~C*16:01 | 0.0238 | A*02:01~B*57:03~C*08:02 | 0.0009 | A*01:01~B*40:02~C*02:02 | 0.0004 | A*02:01~B*07:02~C*07:02 | 0.0060 |
| A*02:05~B*07:02~C*15:02 | 0.0046 | A*02:01~B*49:01~C*07:01 | 0.0029 | A*02:01~B*15:22~C*04:01 | 0.0003 | A*02:01~B*49:01~C*07:01 | 0.0087 | A*02:01~B*57:03~C*18:01 | 0.0009 | A*01:01~B*41:01~C*16:02 | 0.0003 | A*02:01~B*07:02~C*15:05 | 0.0008 |
| A*02:05~B*27:03~C*02:02 | 0.0046 | A*02:01~B*51:01~C*12:03 | 0.0029 | A*02:01~B*18:01~C*02:02 | 0.0001 | A*02:01~B*51:01~C*16:01 | 0.0065 | A*02:01~B*57:03~C*18:02 | 0.0029 | A*01:01~B*41:01~C*17:01 | 0.0012 | A*02:01~B*07:09~C*07:02 | 0.0008 |
| A*02:05~B*35:01~C*04:01 | 0.0046 | A*02:01~B*51:01~C*16:02 | 0.0029 | A*02:01~B*18:01~C*07:04 | 0.0016 | A*02:01~B*53:01~C*04:01 | 0.0093 | A*02:01~B*58:01~C*03:02 | 0.0018 | A*01:01~B*44:02~C*05:01 | 0.0004 | A*02:01~B*08:01~C*07:02 | 0.0008 |
| A*02:05~B*42:01~C*16:01 | 0.0092 | A*02:01~B*53:01~C*04:01 | 0.0145 | A*02:01~B*18:01~C*12:03 | 0.0003 | A*02:01~B*53:01~C*16:01 | 0.0022 | A*02:01~B*58:02~C*06:02 | 0.0007 | A*01:01~B*44:03~C*06:02 | 0.0003 | A*02:01~B*08:01~C*18:00 | 0.0008 |
| A*02:05~B*44:03~C*04:01 | 0.0046 | A*02:01~B*53:01~C*06:02 | 0.0029 | A*02:01~B*18:01~C*15:05 | 0.0004 | A*02:01~B*57:03~C*07:01 | 0.0065 | A*02:01~B*81:01~C*08:04 | 0.0018 | A*01:01~B*44:03~C*16:01 | 0.0010 | A*02:01~B*08:01~C*18:02 | 0.0008 |
| A*02:05~B*45:01~C*16:01 | 0.0046 | A*02:01~B*57:03~C*08:02 | 0.0029 | A*02:01~B*35:01~C*04:01 | 0.0007 | A*02:01~B*57:03~C*08:02 | 0.0043 | A*02:02~B*07:02~C*07:02 | 0.0009 | A*01:01~B*44:03~C*16:02 | 0.0008 | A*02:01~B*13:02~C*06:02 | 0.0030 |
| A*02:05~B*49:01~C*07:01 | 0.0138 | A*02:01~B*58:02~C*06:02 | 0.0065 | A*02:01~B*40:01~C*03:04 | 0.0006 | A*02:01~B*58:01~C*03:02 | 0.0108 | A*02:02~B*08:01~C*07:01 | 0.0009 | A*01:01~B*45:01~C*06:02 | 0.0005 | A*02:01~B*14:01~C*01:02 | 0.0008 |
| A*02:05~B*58:01~C*07:01 | 0.0046 | A*02:01~B*73:01~C*15:02 | 0.0029 | A*02:01~B*42:01~C*17:01 | 0.0024 | A*02:02~B*07:02~C*07:02 | 0.0022 | A*02:02~B*14:02~C*08:02 | 0.0019 | A*01:01~B*46:01~C*01:02 | 0.0003 | A*02:01~B*14:02~C*08:02 | 0.0010 |
| A*02:14~B*73:01~C*15:02 | 0.0046 | A*02:02~B*07:02~C*07:02 | 0.0029 | A*02:01~B*42:02~C*17:00 | 0.0003 | A*02:02~B*14:02~C*08:02 | 0.0022 | A*02:02~B*15:03~C*02:10 | 0.0022 | A*01:01~B*49:01~C*07:01 | 0.0009 | A*02:01~B*15:01~C*02:02 | 0.0008 |
| A*03:01~B*15:03~C*02:10 | 0.0092 | A*02:02~B*15:03~C*02:10 | 0.0031 | A*02:01~B*44:03~C*04:01 | 0.0007 | A*02:02~B*35:01~C*06:02 | 0.0043 | A*02:02~B*15:16~C*14:02 | 0.0018 | A*01:01~B*50:01~C*06:02 | 0.0003 | A*02:01~B*15:01~C*03:03 | 0.0015 |
| A*03:01~B*47:03~C*07:01 | 0.0046 | A*02:02~B*15:16~C*14:02 | 0.0058 | A*02:01~B*44:03~C*07:01 | 0.0016 | A*02:02~B*51:01~C*16:01 | 0.0043 | A*02:02~B*18:01~C*02:10 | 0.0009 | A*01:01~B*51:01~C*01:02 | 0.0005 | A*02:01~B*15:01~C*03:04 | 0.0008 |
| A*03:01~B*49:01~C*07:01 | 0.0046 | A*02:02~B*18:01~C*04:01 | 0.0029 | A*02:01~B*45:00~C*16:01 | 0.0003 | A*02:02~B*53:01~C*03:04 | 0.0022 | A*02:02~B*18:01~C*05:01 | 0.0008 | A*01:01~B*51:01~C*04:01 | 0.0003 | A*02:01~B*15:01~C*04:01 | 0.0015 |
| A*03:01~B*58:02~C*06:02 | 0.0046 | A*02:02~B*41:01~C*17:01 | 0.0029 | A*02:01~B*45:01~C*16:01 | 0.0096 | A*02:02~B*58:02~C*06:02 | 0.0303 | A*02:02~B*40:16~C*08:02 | 0.0009 | A*01:01~B*51:01~C*14:02 | 0.0006 | A*02:01~B*15:01~C*08:02 | 0.0008 |
| A*11:01~B*35:01~C*04:01 | 0.0046 | A*02:02~B*45:01~C*06:02 | 0.0029 | A*02:01~B*45:07~C*16:01 | 0.0034 | A*02:04~B*53:01~C*04:01 | 0.0022 | A*02:02~B*41:01~C*07:01 | 0.0009 | A*01:01~B*51:01~C*15:02 | 0.0006 | A*02:01~B*15:03~C*02:10 | 0.0034 |
| A*11:01~B*44:03~C*07:01 | 0.0046 | A*02:02~B*51:01~C*16:01 | 0.0058 | A*02:01~B*49:01~C*07:01 | 0.0018 | A*02:05~B*49:01~C*07:01 | 0.0022 | A*02:02~B*42:01~C*17:01 | 0.0007 | A*01:01~B*51:01~C*15:13 | 0.0003 | A*02:01~B*15:10~C*03:04 | 0.0023 |
| A*23:01~B*13:02~C*16:04 | 0.0046 | A*02:02~B*58:01~C*06:02 | 0.0115 | A*02:01~B*51:01~C*16:01 | 0.0006 | A*02:05~B*58:01~C*07:01 | 0.0022 | A*02:02~B*44:03~C*03:03 | 0.0009 | A*01:01~B*52:01~C*12:02 | 0.0050 | A*02:01~B*15:16~C*14:02 | 0.0015 |
| A*23:01~B*14:02~C*08:02 | 0.0046 | A*02:02~B*58:02~C*06:02 | 0.0433 | A*02:01~B*53:01~C*04:01 | 0.0020 | A*02:14~B*14:02~C*08:02 | 0.0022 | A*02:02~B*44:03~C*04:01 | 0.0018 | A*01:01~B*52:01~C*16:04 | 0.0003 | A*02:01~B*18:01~C*07:04 | 0.0008 |
| A*23:01~B*15:10~C*03:04 | 0.0046 | A*02:05~B*07:05~C*07:02 | 0.0029 | A*02:01~B*57:02~C*18:00 | 0.0003 | A*02:14~B*44:15~C*04:07 | 0.0022 | A*02:02~B*44:03~C*14:03 | 0.0009 | A*01:01~B*53:01~C*12:02 | 0.0003 | A*02:01~B*18:01~C*12:03 | 0.0008 |
| A*23:01~B*15:17~C*05:01 | 0.0046 | A*02:05~B*27:03~C*02:02 | 0.0058 | A*02:01~B*57:03~C*07:01 | 0.0003 | A*02:14~B*49:01~C*15:02 | 0.0022 | A*02:02~B*51:01~C*16:01 | 0.0044 | A*01:01~B*55:01~C*03:03 | 0.0006 | A*02:01~B*27:05~C*01:02 | 0.0030 |
| A*23:01~B*44:03~C*04:01 | 0.0046 | A*02:05~B*58:01~C*06:02 | 0.0029 | A*02:01~B*57:03~C*08:02 | 0.0003 | A*03:01~B*14:02~C*08:02 | 0.0043 | A*02:02~B*53:01~C*04:01 | 0.0061 | A*01:01~B*56:01~C*01:02 | 0.0004 | A*02:01~B*27:05~C*02:02 | 0.0030 |
| A*23:01~B*57:02~C*07:01 | 0.0046 | A*02:05~B*58:01~C*07:01 | 0.0029 | A*02:01~B*58:01~C*03:02 | 0.0036 | A*03:01~B*15:03~C*06:02 | 0.0022 | A*02:02~B*53:01~C*16:01 | 0.0009 | A*01:01~B*57:01~C*06:02 | 0.0325 | A*02:01~B*35:01~C*04:01 | 0.0009 |
| A*23:01~B*58:02~C*06:02 | 0.0046 | A*02:14~B*18:01~C*04:01 | 0.0029 | A*02:01~B*58:01~C*07:01 | 0.0003 | A*03:01~B*15:10~C*03:04 | 0.0082 | A*02:02~B*57:01~C*04:01 | 0.0009 | A*01:01~B*57:01~C*07:01 | 0.0022 | A*02:01~B*35:01~C*16:01 | 0.0008 |
| A*23:02~B*14:02~C*08:02 | 0.0046 | A*02:14~B*18:03~C*04:01 | 0.0029 | A*02:01~B*58:02~C*06:02 | 0.0036 | A*03:01~B*18:01~C*07:04 | 0.0065 | A*02:02~B*57:02~C*02:10 | 0.0009 | A*01:01~B*57:02~C*07:01 | 0.0003 | A*02:01~B*35:43~C*07:02 | 0.0008 |
| A*24:02~B*18:01~C*07:01 | 0.0046 | A*03:01~B*14:02~C*08:02 | 0.0116 | A*02:01~B*58:10~C*03:02 | 0.0003 | A*03:01~B*35:01~C*04:01 | 0.0065 | A*02:02~B*57:03~C*18:02 | 0.0008 | A*01:01~B*57:03~C*06:02 | 0.0003 | A*02:01~B*39:01~C*12:03 | 0.0008 |
| A*24:02~B*53:01~C*07:01 | 0.0046 | A*03:01~B*15:03~C*18:01 | 0.0029 | A*02:01~B*81:00~C*18:00 | 0.0003 | A*03:01~B*47:01~C*07:01 | 0.0022 | A*02:02~B*58:01~C*06:02 | 0.0018 | A*01:01~B*57:03~C*07:01 | 0.0008 | A*02:01~B*39:10~C*06:02 | 0.0008 |
| A*24:02~B*57:03~C*18:01 | 0.0092 | A*03:01~B*35:01~C*06:02 | 0.0058 | A*02:02~B*14:01~C*08:04 | 0.0009 | A*03:01~B*49:01~C*07:01 | 0.0108 | A*02:02~B*58:02~C*06:02 | 0.0061 | A*01:01~B*58:01~C*03:02 | 0.0007 | A*02:01~B*39:10~C*12:03 | 0.0059 |
| A*26:01~B*15:03~C*02:10 | 0.0046 | A*03:01~B*44:03~C*07:01 | 0.0029 | A*02:02~B*15:03~C*02:10 | 0.0013 | A*03:01~B*53:01~C*04:01 | 0.0026 | A*02:02~B*58:02~C*08:02 | 0.0009 | A*01:01~B*58:01~C*06:02 | 0.0003 | A*02:01~B*39:24~C*07:01 | 0.0008 |
| A*26:01~B*15:03~C*04:01 | 0.0046 | A*03:01~B*45:01~C*06:02 | 0.0065 | A*02:02~B*15:16~C*14:02 | 0.0034 | A*03:01~B*57:03~C*07:01 | 0.0022 | A*02:04~B*51:01~C*16:01 | 0.0009 | A*01:01~B*58:01~C*07:01 | 0.0010 | A*02:01~B*40:01~C*03:04 | 0.0015 |
| A*26:01~B*50:01~C*06:02 | 0.0046 | A*03:01~B*47:01~C*06:02 | 0.0058 | A*02:02~B*18:01~C*05:01 | 0.0024 | A*03:01~B*58:01~C*06:02 | 0.0065 | A*02:05~B*07:02~C*07:02 | 0.0017 | A*01:01~B*67:01~C*12:03 | 0.0003 | A*02:01~B*42:01~C*17:01 | 0.0054 |
| A*26:01~B*52:01~C*12:02 | 0.0092 | A*03:01~B*49:01~C*07:01 | 0.0134 | A*02:02~B*42:01~C*17:01 | 0.0003 | A*03:01~B*58:02~C*06:02 | 0.0043 | A*02:05~B*15:03~C*02:10 | 0.0009 | A*01:02~B*15:01~C*07:01 | 0.0003 | A*02:01~B*44:02~C*05:01 | 0.0038 |
| A*29:02~B*13:02~C*06:02 | 0.0138 | A*03:01~B*53:01~C*06:02 | 0.0029 | A*02:02~B*44:03~C*08:04 | 0.0003 | A*23:01~B*07:02~C*07:02 | 0.0108 | A*02:05~B*15:03~C*04:01 | 0.0009 | A*01:02~B*51:08~C*16:02 | 0.0003 | A*02:01~B*44:02~C*06:02 | 0.0008 |
| A*29:02~B*42:01~C*17:01 | 0.0046 | A*03:01~B*58:01~C*06:02 | 0.0029 | A*02:02~B*53:01~C*04:01 | 0.0006 | A*23:01~B*08:01~C*03:04 | 0.0022 | A*02:05~B*18:01~C*02:10 | 0.0009 | A*01:03~B*57:02~C*01:02 | 0.0003 | A*02:01~B*44:02~C*07:04 | 0.0008 |
| A*29:02~B*44:03~C*07:02 | 0.0138 | A*03:01~B*58:02~C*06:02 | 0.0090 | A*02:02~B*57:03~C*07:01 | 0.0039 | A*23:01~B*08:01~C*07:01 | 0.0022 | A*02:05~B*35:01~C*04:01 | 0.0009 | A*01:03~B*73:01~C*04:01 | 0.0003 | A*02:01~B*44:03~C*03:03 | 0.0008 |
| A*29:02~B*45:01~C*06:02 | 0.0046 | A*23:01~B*07:02~C*07:02 | 0.0029 | A*02:02~B*58:01~C*06:02 | 0.0003 | A*23:01~B*08:01~C*07:02 | 0.0022 | A*02:05~B*42:01~C*17:01 | 0.0009 | A*02:01~B*07:02~C*07:02 | 0.0241 | A*02:01~B*44:03~C*04:01 | 0.0008 |
| A*29:02~B*57:03~C*04:01 | 0.0046 | A*23:01~B*08:01~C*03:04 | 0.0058 | A*02:02~B*82:02~C*18:00 | 0.0003 | A*23:01~B*15:03~C*02:10 | 0.0028 | A*02:05~B*45:01~C*16:01 | 0.0010 | A*02:01~B*08:01~C*03:04 | 0.0003 | A*02:01~B*44:03~C*16:01 | 0.0015 |
| A*29:02~B*58:02~C*06:02 | 0.0046 | A*23:01~B*08:01~C*07:04 | 0.0029 | A*02:05~B*07:02~C*02:10 | 0.0003 | A*23:01~B*15:10~C*03:04 | 0.0022 | A*02:05~B*58:01~C*03:02 | 0.0009 | A*02:01~B*08:01~C*07:01 | 0.0057 | A*02:01~B*45:01~C*06:02 | 0.0009 |
| A*30:01~B*15:10~C*08:02 | 0.0459 | A*23:01~B*14:03~C*08:02 | 0.0029 | A*02:05~B*07:02~C*03:04 | 0.0003 | A*23:01~B*15:10~C*04:01 | 0.0022 | A*02:05~B*58:01~C*06:02 | 0.0009 | A*02:01~B*13:02~C*06:02 | 0.0080 | A*02:01~B*45:01~C*16:01 | 0.0080 |
| A*30:01~B*42:01~C*17:01 | 0.0046 | A*23:01~B*15:03~C*02:10 | 0.0058 | A*02:05~B*07:02~C*07:02 | 0.0006 | A*23:01~B*15:17~C*05:01 | 0.0022 | A*02:05~B*58:01~C*07:01 | 0.0097 | A*02:01~B*14:01~C*08:02 | 0.0031 | A*02:01~B*48:01~C*12:03 | 0.0008 |
| A*30:01~B*44:03~C*04:07 | 0.0092 | A*23:01~B*15:17~C*05:01 | 0.0029 | A*02:05~B*08:01~C*02:17 | 0.0024 | A*23:01~B*40:12~C*15:02 | 0.0022 | A*02:14~B*14:01~C*07:02 | 0.0009 | A*02:01~B*14:02~C*08:02 | 0.0070 | A*02:01~B*49:01~C*07:01 | 0.0029 |
| A*30:01~B*57:03~C*18:01 | 0.0046 | A*23:01~B*18:01~C*18:01 | 0.0116 | A*02:05~B*13:02~C*06:02 | 0.0006 | A*23:01~B*42:01~C*17:01 | 0.0043 | A*02:14~B*18:03~C*04:01 | 0.0009 | A*02:01~B*15:01~C*01:02 | 0.0011 | A*02:01~B*50:01~C*06:02 | 0.0008 |
| A*30:01~B*81:01~C*08:02 | 0.0046 | A*23:01~B*42:01~C*03:04 | 0.0029 | A*02:05~B*14:01~C*08:04 | 0.0104 | A*23:01~B*44:03~C*04:01 | 0.0065 | A*03new~B*15:10~C*03:04 | 0.0009 | A*02:01~B*15:01~C*02:02 | 0.0003 | A*02:01~B*51:01~C*01:02 | 0.0008 |
| A*30:02~B*14:02~C*08:02 | 0.0092 | A*23:01~B*44:03~C*04:01 | 0.0029 | A*02:05~B*15:01~C*04:01 | 0.0003 | A*23:01~B*45:01~C*06:02 | 0.0152 | A*03:01~B*08:01~C*07:01 | 0.0009 | A*02:01~B*15:01~C*03:03 | 0.0087 | A*02:01~B*51:01~C*14:02 | 0.0008 |
| A*30:02~B*15:03~C*02:10 | 0.0092 | A*23:01~B*44:03~C*07:01 | 0.0058 | A*02:05~B*15:03~C*02:10 | 0.0007 | A*23:01~B*51:01~C*16:01 | 0.0000 | A*03:01~B*13:03~C*06:02 | 0.0009 | A*02:01~B*15:01~C*03:04 | 0.0120 | A*02:01~B*51:01~C*15:02 | 0.0015 |
| A*30:02~B*15:10~C*04:01 | 0.0046 | A*23:01~B*49:01~C*07:01 | 0.0145 | A*02:05~B*15:03~C*04:01 | 0.0003 | A*23:01~B*51:01~C*16:02 | 0.0022 | A*03:01~B*14:01~C*08:02 | 0.0027 | A*02:01~B*15:01~C*04:01 | 0.0027 | A*02:01~B*51:01~C*16:01 | 0.0030 |
| A*30:02~B*15:31~C*04:07 | 0.0000 | A*23:01~B*53:01~C*04:01 | 0.0087 | A*02:05~B*15:10~C*03:04 | 0.0006 | A*23:01~B*53:01~C*04:01 | 0.0015 | A*03:01~B*15:03~C*02:02 | 0.0009 | A*02:01~B*15:10~C*03:04 | 0.0002 | A*02:01~B*51:01~C*16:02 | 0.0008 |
| A*30:02~B*18:01~C*07:04 | 0.0046 | A*23:01~B*57:03~C*07:01 | 0.0087 | A*02:05~B*15:10~C*07:02 | 0.0003 | A*23:01~B*53:01~C*06:02 | 0.0022 | A*03:01~B*15:03~C*02:10 | 0.0079 | A*02:01~B*15:16~C*14:02 | 0.0003 | A*02:01~B*52:01~C*16:01 | 0.0048 |
| A*30:02~B*18:03~C*04:01 | 0.0046 | A*23:01~B*81:01~C*18:01 | 0.0058 | A*02:05~B*15:10~C*08:02 | 0.0003 | A*23:01~B*58:01~C*03:02 | 0.0043 | A*03:01~B*15:10~C*03:04 | 0.0035 | A*02:01~B*15:17~C*05:01 | 0.0003 | A*02:01~B*53:01~C*04:01 | 0.0074 |
| A*30:02~B*39:10~C*12:03 | 0.0046 | A*24:02~B*15:03~C*02:10 | 0.0029 | A*02:05~B*15:10~C*08:04 | 0.0027 | A*23:01~B*58:02~C*04:01 | 0.0022 | A*03:01~B*15:16~C*16:01 | 0.0009 | A*02:01~B*15:17~C*07:01 | 0.0003 | A*02:01~B*53:01~C*16:01 | 0.0008 |
| A*30:02~B*42:02~C*17:01 | 0.0229 | A*24:02~B*15:10~C*03:04 | 0.0029 | A*02:05~B*15:10~C*16:01 | 0.0003 | A*23:01~B*58:02~C*06:02 | 0.0043 | A*03:01~B*18:01~C*02:02 | 0.0009 | A*02:01~B*15:31~C*04:07 | 0.0003 | A*02:01~B*57:02~C*18:00 | 0.0023 |
| A*30:02~B*44:03~C*04:01 | 0.0046 | A*24:02~B*35:01~C*04:01 | 0.0029 | A*02:05~B*15:18~C*03:04 | 0.0003 | A*26:01~B*15:10~C*03:04 | 0.0022 | A*03:01~B*35:01~C*04:01 | 0.0009 | A*02:01~B*18:01~C*02:02 | 0.0003 | A*02:01~B*57:03~C*07:01 | 0.0054 |
| A*30:02~B*45:01~C*06:02 | 0.0046 | A*24:02~B*41:01~C*07:01 | 0.0029 | A*02:05~B*18:01~C*02:02 | 0.0006 | A*26:01~B*15:17~C*17:01 | 0.0022 | A*03:01~B*44:03~C*03:04 | 0.0009 | A*02:01~B*18:01~C*05:01 | 0.0007 | A*02:01~B*57:03~C*18:00 | 0.0008 |
| A*30:02~B*45:01~C*16:01 | 0.0046 | A*24:02~B*57:03~C*07:01 | 0.0029 | A*02:05~B*35:01~C*08:04 | 0.0003 | A*26:01~B*41:01~C*07:01 | 0.0022 | A*03:01~B*44:03~C*07:01 | 0.0009 | A*02:01~B*18:01~C*07:01 | 0.0052 | A*02:01~B*58:01~C*03:02 | 0.0008 |
| A*30:02~B*53:01~C*04:01 | 0.0046 | A*26:01~B*18:01~C*07:04 | 0.0029 | A*02:05~B*41:01~C*17:00 | 0.0003 | A*26:01~B*49:01~C*07:01 | 0.0065 | A*03:01~B*44:03~C*14:03 | 0.0009 | A*02:01~B*18:01~C*12:03 | 0.0018 | A*02:01~B*58:02~C*06:02 | 0.0018 |
| A*30:02~B*58:01~C*03:02 | 0.0046 | A*26:01~B*58:02~C*06:02 | 0.0029 | A*02:05~B*41:01~C*17:01 | 0.0009 | A*26:01~B*58:01~C*03:02 | 0.0022 | A*03:01~B*45:01~C*06:02 | 0.0004 | A*02:01~B*18:20~C*07:01 | 0.0003 | A*02:01~B*78:01~C*16:01 | 0.0015 |
| A*30:04~B*45:01~C*06:02 | 0.0092 | A*26:03~B*56:01~C*01:02 | 0.0029 | A*02:05~B*42:01~C*17:00 | 0.0003 | A*26:01~B*81:01~C*04:01 | 0.0022 | A*03:01~B*45:01~C*16:01 | 0.0009 | A*02:01~B*27:02~C*02:02 | 0.0009 | A*02:01~B*81:01~C*08:04 | 0.0012 |
| A*30:04~B*57:02~C*18:01 | 0.0092 | A*26:12~B*58:01~C*06:02 | 0.0029 | A*02:05~B*42:02~C*17:01 | 0.0003 | A*29:01~B*42:01~C*17:01 | 0.0022 | A*03:01~B*49:01~C*07:01 | 0.0009 | A*02:01~B*27:02~C*02:29 | 0.0003 | A*02:02~B*07:02~C*04:01 | 0.0007 |
| A*30:04~B*57:03~C*07:01 | 0.0046 | A*26:12~B*58:02~C*06:02 | 0.0058 | A*02:05~B*44:03~C*04:01 | 0.0003 | A*29:02~B*07:02~C*07:02 | 0.0087 | A*03:01~B*53:01~C*06:03 | 0.0009 | A*02:01~B*27:03~C*02:02 | 0.0003 | A*02:02~B*13:02~C*05:01 | 0.0008 |
| A*30:04~B*57:03~C*18:01 | 0.0046 | A*29:02~B*07:02~C*07:02 | 0.0029 | A*02:05~B*44:03~C*07:01 | 0.0012 | A*29:02~B*15:10~C*08:04 | 0.0022 | A*03:01~B*57:03~C*18:02 | 0.0012 | A*02:01~B*27:05~C*01:02 | 0.0098 | A*02:02~B*14:03~C*08:02 | 0.0008 |
| A*31:04~B*07:02~C*07:02 | 0.0046 | A*29:02~B*13:02~C*06:02 | 0.0058 | A*02:05~B*45:00~C*17:00 | 0.0003 | A*29:02~B*15:16~C*14:02 | 0.0022 | A*03:01~B*58:01~C*03:02 | 0.0018 | A*02:01~B*27:05~C*02:02 | 0.0062 | A*02:02~B*15:03~C*02:10 | 0.0015 |
| A*33:01~B*14:02~C*08:02 | 0.0046 | A*29:02~B*14:02~C*08:02 | 0.0029 | A*02:05~B*45:07~C*16:01 | 0.0009 | A*29:02~B*18:01~C*02:02 | 0.0022 | A*03:01~B*58:01~C*06:02 | 0.0009 | A*02:01~B*35:01~C*01:02 | 0.0003 | A*02:02~B*15:10~C*03:04 | 0.0008 |
| A*33:01~B*15:16~C*14:02 | 0.0046 | A*29:02~B*15:03~C*18:01 | 0.0029 | A*02:05~B*53:01~C*04:01 | 0.0003 | A*29:02~B*18:01~C*07:04 | 0.0022 | A*03:01~B*58:02~C*06:02 | 0.0170 | A*02:01~B*35:01~C*04:01 | 0.0040 | A*02:02~B*15:16~C*14:02 | 0.0015 |
| A*33:01~B*53:01~C*04:01 | 0.0046 | A*29:02~B*35:01~C*04:01 | 0.0029 | A*02:05~B*58:01~C*04:01 | 0.0003 | A*29:02~B*18:03~C*04:01 | 0.0022 | A*23:01~B*07:02~C*07:02 | 0.0050 | A*02:01~B*35:03~C*04:01 | 0.0019 | A*02:02~B*18:01~C*05:01 | 0.0015 |
| A*33:03~B*14:02~C*08:02 | 0.0046 | A*29:02~B*35:01~C*15:02 | 0.0029 | A*02:05~B*58:01~C*06:02 | 0.0024 | A*29:02~B*42:01~C*17:01 | 0.0022 | A*23:01~B*08:01~C*03:04 | 0.0035 | A*02:01~B*35:03~C*12:03 | 0.0003 | A*02:02~B*35:01~C*04:01 | 0.0013 |
| A*34:02~B*08:01~C*07:01 | 0.0046 | A*29:02~B*41:01~C*17:01 | 0.0029 | A*02:05~B*58:01~C*07:01 | 0.0185 | A*29:02~B*44:03~C*04:01 | 0.0022 | A*23:01~B*08:01~C*07:01 | 0.0045 | A*02:01~B*35:12~C*03:03 | 0.0003 | A*02:02~B*35:01~C*06:02 | 0.0008 |
| A*34:02~B*15:03~C*02:10 | 0.0046 | A*29:02~B*42:01~C*17:01 | 0.0029 | A*02:05~B*58:01~C*07:18 | 0.0006 | A*29:02~B*45:01~C*07:01 | 0.0043 | A*23:01~B*08:01~C*08:02 | 0.0009 | A*02:01~B*35:43~C*01:02 | 0.0003 | A*02:02~B*35:01~C*07:05 | 0.0008 |
| A*34:02~B*53:01~C*04:01 | 0.0046 | A*29:02~B*45:01~C*16:01 | 0.0029 | A*02:05~B*58:02~C*06:02 | 0.0034 | A*29:02~B*45:01~C*16:01 | 0.0022 | A*23:01~B*13:02~C*06:02 | 0.0009 | A*02:01~B*37:01~C*06:02 | 0.0011 | A*02:02~B*35:01~C*14:02 | 0.0008 |
| A*34:02~B*81:01~C*18:01 | 0.0275 | A*29:02~B*58:02~C*06:02 | 0.0029 | A*02:11~B*15:18~C*07:04 | 0.0003 | A*29:02~B*53:01~C*06:02 | 0.0022 | A*23:01~B*14:01~C*02:10 | 0.0009 | A*02:01~B*38:01~C*07:02 | 0.0003 | A*02:02~B*42:01~C*17:01 | 0.0008 |
| A*36:01~B*53:01~C*04:01 | 0.0046 | A*30:01~B*15:10~C*03:04 | 0.0116 | A*02:11~B*40:06~C*14:02 | 0.0003 | A*29:02~B*57:03~C*18:01 | 0.0022 | A*23:01~B*14:01~C*08:02 | 0.0015 | A*02:01~B*38:01~C*12:03 | 0.0038 | A*02:02~B*45:01~C*16:01 | 0.0010 |
| A*66:01~B*35:01~C*04:01 | 0.0183 | A*30:01~B*27:03~C*02:02 | 0.0029 | A*02:14~B*14:01~C*06:02 | 0.0003 | A*29:02~B*58:01~C*06:02 | 0.0022 | A*23:01~B*14:02~C*08:02 | 0.0085 | A*02:01~B*39:01~C*07:02 | 0.0007 | A*02:02~B*49:01~C*07:01 | 0.0014 |
| A*66:01~B*58:02~C*06:02 | 0.0138 | A*30:01~B*39:10~C*12:03 | 0.0058 | A*02:14~B*44:03~C*04:01 | 0.0027 | A*29:02~B*73:01~C*15:02 | 0.0022 | A*23:01~B*14:03~C*08:02 | 0.0009 | A*02:01~B*39:01~C*12:03 | 0.0019 | A*02:02~B*51:01~C*16:01 | 0.0015 |
| A*68:01~B*07:02~C*07:02 | 0.0092 | A*30:01~B*39:10~C*17:01 | 0.0029 | A*02:14~B*58:02~C*08:04 | 0.0003 | A*30:01~B*07:02~C*07:02 | 0.0023 | A*23:01~B*15:03~C*02:10 | 0.0136 | A*02:01~B*39:01~C*17:03 | 0.0003 | A*02:02~B*53:01~C*04:01 | 0.0083 |
| A*68:01~B*15:03~C*08:02 | 0.0046 | A*30:01~B*40:16~C*08:02 | 0.0029 | A*03:01~B*07:02~C*07:02 | 0.0003 | A*30:01~B*08:01~C*17:01 | 0.0043 | A*23:01~B*15:10~C*08:02 | 0.0014 | A*02:01~B*39:02~C*07:02 | 0.0003 | A*02:02~B*53:01~C*06:02 | 0.0008 |
| A*68:01~B*15:17~C*07:01 | 0.0046 | A*30:01~B*42:01~C*17:01 | 0.0173 | A*03:01~B*08:01~C*02:10 | 0.0003 | A*30:01~B*13:02~C*06:02 | 0.0041 | A*23:01~B*15:10~C*16:01 | 0.0044 | A*02:01~B*39:06~C*07:02 | 0.0008 | A*02:02~B*53:01~C*08:02 | 0.0008 |
| A*68:01~B*37:01~C*07:01 | 0.0138 | A*30:01~B*58:02~C*06:02 | 0.0029 | A*03:01~B*08:01~C*03:04 | 0.0003 | A*30:01~B*14:01~C*04:01 | 0.0022 | A*23:01~B*15:17~C*05:01 | 0.0009 | A*02:01~B*39:10~C*06:02 | 0.0003 | A*02:02~B*57:03~C*04:01 | 0.0008 |
| A*68:01~B*58:02~C*04:01 | 0.0046 | A*30:02~B*07:02~C*07:02 | 0.0036 | A*03:01~B*08:01~C*07:01 | 0.0006 | A*30:01~B*14:02~C*08:02 | 0.0031 | A*23:01~B*18:01~C*07:04 | 0.0009 | A*02:01~B*39:10~C*12:03 | 0.0003 | A*02:02~B*57:03~C*07:01 | 0.0008 |
| A*68:01~B*58:02~C*06:02 | 0.0367 | A*30:02~B*08:01~C*07:01 | 0.0029 | A*03:01~B*08:01~C*07:02 | 0.0119 | A*30:01~B*15:10~C*03:04 | 0.0077 | A*23:01~B*35:01~C*17:01 | 0.0009 | A*02:01~B*39:24~C*07:01 | 0.0003 | A*02:02~B*57:03~C*08:02 | 0.0008 |
| A*68:02~B*07:02~C*07:02 | 0.0046 | A*30:02~B*13:02~C*06:02 | 0.0029 | A*03:01~B*13:02~C*06:02 | 0.0006 | A*30:01~B*15:37~C*03:04 | 0.0022 | A*23:01~B*41:01~C*08:02 | 0.0009 | A*02:01~B*40:01~C*03:04 | 0.0160 | A*02:02~B*57:03~C*18:00 | 0.0008 |
| A*68:02~B*14:01~C*08:02 | 0.0046 | A*30:02~B*14:02~C*08:02 | 0.0029 | A*03:01~B*13:03~C*06:02 | 0.0012 | A*30:01~B*18:01~C*07:04 | 0.0043 | A*23:01~B*42:01~C*17:01 | 0.0016 | A*02:01~B*40:01~C*07:02 | 0.0003 | A*02:02~B*58:01~C*03:02 | 0.0035 |
| A*68:02~B*15:10~C*03:04 | 0.0046 | A*30:02~B*15:03~C*02:10 | 0.0116 | A*03:01~B*15:03~C*02:10 | 0.0024 | A*30:01~B*42:01~C*17:01 | 0.0238 | A*23:01~B*44:03~C*03:03 | 0.0052 | A*02:01~B*40:02~C*02:02 | 0.0039 | A*02:02~B*81:01~C*08:04 | 0.0017 |
| A*68:02~B*27:03~C*02:02 | 0.0596 | A*30:02~B*18:01~C*07:04 | 0.0058 | A*03:01~B*15:10~C*03:04 | 0.0037 | A*30:01~B*42:02~C*17:01 | 0.0022 | A*23:01~B*44:03~C*04:01 | 0.0077 | A*02:01~B*40:02~C*03:04 | 0.0004 | A*02:02~B*81:01~C*18:00 | 0.0015 |
| A*68:02~B*42:01~C*17:01 | 0.0046 | A*30:02~B*35:02~C*04:01 | 0.0029 | A*03:01~B*15:10~C*04:01 | 0.0042 | A*30:01~B*45:01~C*06:02 | 0.0065 | A*23:01~B*45:01~C*06:02 | 0.0135 | A*02:01~B*40:02~C*15:02 | 0.0007 | A*02:02~B*81:01~C*18:01 | 0.0008 |
| A*68:02~B*45:01~C*16:01 | 0.0046 | A*30:02~B*39:10~C*12:03 | 0.0029 | A*03:01~B*18:01~C*07:04 | 0.0004 | A*30:01~B*47:03~C*07:01 | 0.0022 | A*23:01~B*45:01~C*16:01 | 0.0036 | A*02:01~B*40:04~C*03:04 | 0.0003 | A*02:04~B*41:01~C*06:02 | 0.0008 |
| A*68:02~B*49:01~C*07:01 | 0.0092 | A*30:02~B*42:01~C*17:01 | 0.0058 | A*03:01~B*39:10~C*12:03 | 0.0008 | A*30:01~B*57:03~C*17:01 | 0.0022 | A*23:01~B*53:01~C*04:01 | 0.0057 | A*02:01~B*41:01~C*07:01 | 0.0003 | A*02:04~B*51:01~C*15:02 | 0.0015 |
| A*68:02~B*53:01~C*06:02 | 0.0092 | A*30:02~B*45:01~C*16:01 | 0.0029 | A*03:01~B*40:01~C*12:03 | 0.0003 | A*30:01~B*58:01~C*04:01 | 0.0043 | A*23:01~B*53:01~C*06:02 | 0.0049 | A*02:01~B*41:01~C*17:00 | 0.0008 | A*02:05~B*07:05~C*07:02 | 0.0008 |
| A*68:02~B*73:01~C*15:02 | 0.0138 | A*30:02~B*49:01~C*07:01 | 0.0051 | A*03:01~B*42:01~C*17:00 | 0.0012 | A*30:01~B*58:02~C*06:02 | 0.0043 | A*23:01~B*53:01~C*16:01 | 0.0009 | A*02:01~B*41:01~C*17:01 | 0.0006 | A*02:05~B*08:01~C*04:01 | 0.0008 |
| A*74:01~B*14:02~C*08:02 | 0.0046 | A*30:02~B*53:01~C*04:01 | 0.0058 | A*03:01~B*42:01~C*17:01 | 0.0014 | A*30:02~B*08:01~C*07:01 | 0.0022 | A*23:01~B*57:03~C*07:01 | 0.0012 | A*02:01~B*44:02~C*02:02 | 0.0006 | A*02:05~B*14:02~C*08:02 | 0.0008 |
| A*74:01~B*15:16~C*14:02 | 0.0046 | A*30:02~B*57:03~C*07:01 | 0.0058 | A*03:01~B*44:03~C*04:01 | 0.0011 | A*30:02~B*08:01~C*07:04 | 0.0043 | A*23:01~B*57:03~C*18:01 | 0.0009 | A*02:01~B*44:02~C*05:01 | 0.0426 | A*02:05~B*27:03~C*02:02 | 0.0008 |
| A*74:01~B*15:31~C*04:01 | 0.0046 | A*30:02~B*57:03~C*18:01 | 0.0029 | A*03:01~B*44:03~C*08:04 | 0.0003 | A*30:02~B*13:02~C*06:02 | 0.0022 | A*23:01~B*57:03~C*18:02 | 0.0006 | A*02:01~B*44:02~C*07:02 | 0.0003 | A*02:05~B*35:01~C*18:00 | 0.0008 |
| A*74:01~B*35:01~C*04:01 | 0.0092 | A*30:02~B*58:01~C*06:02 | 0.0029 | A*03:01~B*44:03~C*14:03 | 0.0003 | A*30:02~B*14:02~C*03:04 | 0.0022 | A*23:01~B*58:01~C*06:02 | 0.0016 | A*02:01~B*44:02~C*07:04 | 0.0016 | A*02:05~B*37:01~C*06:02 | 0.0008 |
| A*74:01~B*44:15~C*14:03 | 0.0046 | A*30:02~B*58:01~C*14:02 | 0.0029 | A*03:01~B*45:01~C*16:01 | 0.0004 | A*30:02~B*14:02~C*08:02 | 0.0108 | A*23:01~B*58:01~C*07:01 | 0.0079 | A*02:01~B*44:02~C*16:04 | 0.0008 | A*02:05~B*44:03~C*04:01 | 0.0008 |
| A*74:01~B*45:01~C*06:02 | 0.0046 | A*30:02~B*58:02~C*06:02 | 0.0058 | A*03:01~B*49:01~C*07:01 | 0.0040 | A*30:02~B*15:03~C*02:10 | 0.0043 | A*23:01~B*58:02~C*06:02 | 0.0056 | A*02:01~B*44:03~C*03:04 | 0.0003 | A*02:05~B*45:01~C*16:01 | 0.0008 |
| A*74:01~B*51:01~C*16:02 | 0.0046 | A*30:04~B*07:02~C*07:02 | 0.0029 | A*03:01~B*49:01~C*07:04 | 0.0003 | A*30:02~B*15:10~C*03:04 | 0.0108 | A*23:01~B*81:01~C*08:04 | 0.0009 | A*02:01~B*44:03~C*04:01 | 0.0027 | A*02:05~B*49:01~C*07:01 | 0.0004 |
| A*74:01~B*51:01~C*17:01 | 0.0046 | A*30:04~B*08:01~C*07:01 | 0.0029 | A*03:01~B*57:03~C*18:01 | 0.0003 | A*30:02~B*18:01~C*07:04 | 0.0051 | A*23:01~B*81:01~C*18:01 | 0.0026 | A*02:01~B*44:03~C*07:02 | 0.0003 | A*02:05~B*53:01~C*04:01 | 0.0008 |
| A*74:01~B*53:01~C*16:01 | 0.0046 | A*30:04~B*44:03~C*14:03 | 0.0029 | A*03:01~B*57:03~C*18:02 | 0.0003 | A*30:02~B*39:10~C*12:03 | 0.0065 | A*23:02~B*39:01~C*12:03 | 0.0009 | A*02:01~B*44:03~C*16:01 | 0.0039 | A*02:05~B*57:03~C*07:01 | 0.0019 |
| A*74:01~B*58:01~C*03:02 | 0.0092 | A*30:09~B*81:01~C*04:01 | 0.0029 | A*03:01~B*58:01~C*03:02 | 0.0002 | A*30:02~B*42:01~C*17:01 | 0.0022 | A*23:02~B*42:01~C*17:01 | 0.0009 | A*02:01~B*44:03~C*16:02 | 0.0003 | A*02:05~B*57:03~C*18:00 | 0.0023 |
| A*74:01~B*58:01~C*06:02 | 0.0229 | A*31:01~B*53:01~C*04:01 | 0.0029 | A*03:01~B*58:01~C*07:01 | 0.0006 | A*30:02~B*45:01~C*16:01 | 0.0065 | A*24:02~B*13:02~C*06:02 | 0.0009 | A*02:01~B*44:05~C*02:02 | 0.0013 | A*02:05~B*57:03~C*18:02 | 0.0015 |
|  |  | A*32:01~B*53:01~C*04:01 | 0.0029 | A*03:01~B*58:02~C*06:02 | 0.0208 | A*30:02~B*49:01~C*07:01 | 0.0022 | A*24:02~B*15:03~C*02:10 | 0.0018 | A*02:01~B*44:27~C*07:04 | 0.0003 | A*02:05~B*58:01~C*07:01 | 0.0038 |
|  |  | A*33:01~B*53:01~C*04:01 | 0.0029 | A*03:01~B*81:00~C*04:01 | 0.0006 | A*30:02~B*53:01~C*04:01 | 0.0078 | A*24:02~B*15:16~C*16:01 | 0.0009 | A*02:01~B*45:01~C*05:01 | 0.0003 | A*02:05~B*58:11~C*07:01 | 0.0008 |
|  |  | A*33:03~B*15:16~C*16:01 | 0.0029 | A*03:01~B*81:01~C*04:01 | 0.0009 | A*30:02~B*57:03~C*07:01 | 0.0043 | A*24:02~B*18:01~C*07:04 | 0.0009 | A*02:01~B*45:01~C*16:01 | 0.0020 | A*02:06~B*27:05~C*02:02 | 0.0023 |
|  |  | A*33:03~B*44:15~C*04:07 | 0.0058 | A*03:01~B*81:01~C*18:01 | 0.0003 | A*30:02~B*57:03~C*18:01 | 0.0022 | A*24:02~B*35:02~C*04:01 | 0.0009 | A*02:01~B*48:01~C*08:01 | 0.0003 | A*02:06~B*27:05~C*03:03 | 0.0008 |
|  |  | A*34:02~B*40:12~C*04:01 | 0.0029 | A*03:02~B*08:01~C*07:02 | 0.0003 | A*30:02~B*81:01~C*04:01 | 0.0022 | A*24:02~B*41:01~C*01:02 | 0.0009 | A*02:01~B*48:01~C*08:03 | 0.0003 | A*02:06~B*27:06~C*03:04 | 0.0008 |
|  |  | A*34:02~B*44:03~C*04:01 | 0.0116 | A*03:21~B*42:01~C*17:01 | 0.0003 | A*30:04~B*14:02~C*08:02 | 0.0022 | A*24:02~B*44:03~C*04:01 | 0.0044 | A*02:01~B*48:02~C*04:01 | 0.0006 | A*02:11~B*52:01~C*08:01 | 0.0008 |
|  |  | A*34:02~B*53:01~C*04:01 | 0.0029 | A*11:01~B*13:01~C*04:03 | 0.0003 | A*30:04~B*41:01~C*17:01 | 0.0022 | A*24:31~B*15:01~C*03:03 | 0.0009 | A*02:01~B*49:01~C*07:01 | 0.0029 | A*02:16~B*27:05~C*15:02 | 0.0008 |
|  |  | A*34:02~B*57:03~C*07:01 | 0.0029 | A*11:01~B*35:03~C*04:01 | 0.0003 | A*30:04~B*44:03~C*07:01 | 0.0022 | A*26:01~B*07:02~C*07:02 | 0.0018 | A*02:01~B*51:01~C*01:02 | 0.0021 | A*03:01~B*07:02~C*07:01 | 0.0023 |
|  |  | A*36:01~B*13:02~C*06:02 | 0.0029 | A*11:01~B*44:02~C*05:01 | 0.0003 | A*30:04~B*53:01~C*06:02 | 0.0022 | A*26:01~B*15:220~C*17:01 | 0.0027 | A*02:01~B*51:01~C*02:02 | 0.0012 | A*03:01~B*07:02~C*07:02 | 0.0099 |
|  |  | A*36:01~B*15:17~C*17:01 | 0.0029 | A*11:01~B*52:01~C*12:02 | 0.0003 | A*30:09~B*81:01~C*04:01 | 0.0087 | A*26:01~B*51:01~C*04:07 | 0.0009 | A*02:01~B*51:01~C*04:01 | 0.0003 | A*03:01~B*07:02~C*15:05 | 0.0008 |
|  |  | A*36:01~B*42:01~C*17:01 | 0.0058 | A*23new~B*44:03~C*03:03 | 0.0003 | A*31:01~B*82:02~C*03:02 | 0.0022 | A*26:01~B*81:01~C*04:01 | 0.0009 | A*02:01~B*51:01~C*05:01 | 0.0003 | A*03:01~B*07:05~C*15:05 | 0.0008 |
|  |  | A*36:01~B*45:01~C*06:02 | 0.0058 | A*23:01~B*07:02~C*02:02 | 0.0003 | A*31:03~B*15:16~C*08:02 | 0.0022 | A*26:12~B*44:03~C*07:01 | 0.0003 | A*02:01~B*51:01~C*07:02 | 0.0003 | A*03:01~B*08:01~C*07:02 | 0.0008 |
|  |  | A*36:01~B*49:01~C*07:01 | 0.0029 | A*23:01~B*07:02~C*02:10 | 0.0005 | A*31:04~B*15:03~C*08:02 | 0.0022 | A*26:12~B*45:01~C*06:02 | 0.0006 | A*02:01~B*51:01~C*14:02 | 0.0076 | A*03:01~B*14:01~C*08:02 | 0.0030 |
|  |  | A*36:01~B*53:01~C*04:01 | 0.0116 | A*23:01~B*07:02~C*07:02 | 0.0020 | A*31:04~B*58:02~C*06:02 | 0.0022 | A*29:01~B*14:01~C*07:01 | 0.0009 | A*02:01~B*51:01~C*15:02 | 0.0042 | A*03:01~B*14:02~C*08:02 | 0.0035 |
|  |  | A*66:01~B*44:03~C*04:01 | 0.0029 | A*23:01~B*07:05~C*07:02 | 0.0012 | A*32:01~B*07:02~C*04:01 | 0.0022 | A*29:01~B*58:02~C*06:02 | 0.0009 | A*02:01~B*51:01~C*15:06 | 0.0003 | A*03:01~B*15:01~C*03:03 | 0.0008 |
|  |  | A*66:01~B*58:01~C*06:02 | 0.0116 | A*23:01~B*08:01~C*02:17 | 0.0011 | A*32:01~B*07:02~C*07:02 | 0.0022 | A*29:01~B*82:01~C*04:07 | 0.0009 | A*02:01~B*51:01~C*15:13 | 0.0002 | A*03:01~B*15:03~C*02:10 | 0.0034 |
|  |  | A*66:01~B*58:02~C*06:02 | 0.0116 | A*23:01~B*08:01~C*03:04 | 0.0062 | A*32:01~B*39:10~C*12:03 | 0.0022 | A*29:02~B*07:02~C*07:02 | 0.0011 | A*02:01~B*51:01~C*16:01 | 0.0004 | A*03:01~B*15:10~C*03:04 | 0.0023 |
|  |  | A*68:01~B*15:16~C*14:02 | 0.0029 | A*23:01~B*08:01~C*07:01 | 0.0082 | A*32:01~B*47:01~C*07:01 | 0.0022 | A*29:02~B*13:02~C*06:02 | 0.0071 | A*02:01~B*51:01~C*16:02 | 0.0011 | A*03:01~B*15:10~C*06:02 | 0.0008 |
|  |  | A*68:01~B*15:17~C*07:01 | 0.0029 | A*23:01~B*08:01~C*16:01 | 0.0003 | A*32:01~B*81:01~C*08:02 | 0.0065 | A*29:02~B*15:03~C*02:10 | 0.0046 | A*02:01~B*51:05~C*04:01 | 0.0003 | A*03:01~B*15:16~C*16:01 | 0.0008 |
|  |  | A*68:01~B*40:16~C*08:02 | 0.0029 | A*23:01~B*08:01~C*17:01 | 0.0003 | A*33:01~B*14:03~C*08:02 | 0.0022 | A*29:02~B*15:31~C*04:07 | 0.0009 | A*02:01~B*52:01~C*12:02 | 0.0034 | A*03:01~B*15:17~C*07:01 | 0.0008 |
|  |  | A*68:02~B*07:02~C*07:02 | 0.0273 | A*23:01~B*13:02~C*06:02 | 0.0003 | A*33:01~B*15:10~C*03:04 | 0.0022 | A*29:02~B*35:01~C*04:01 | 0.0028 | A*02:01~B*52:01~C*16:01 | 0.0003 | A*03:01~B*18:01~C*05:01 | 0.0030 |
|  |  | A*68:02~B*08:01~C*07:02 | 0.0029 | A*23:01~B*14:01~C*02:10 | 0.0016 | A*33:01~B*15:16~C*14:02 | 0.0022 | A*29:02~B*35:01~C*16:01 | 0.0018 | A*02:01~B*53:01~C*04:01 | 0.0003 | A*03:01~B*27:05~C*01:02 | 0.0008 |
|  |  | A*68:02~B*13:02~C*06:02 | 0.0029 | A*23:01~B*14:01~C*08:02 | 0.0003 | A*33:01~B*42:01~C*17:01 | 0.0065 | A*29:02~B*39:10~C*12:03 | 0.0007 | A*02:01~B*55:01~C*03:03 | 0.0019 | A*03:01~B*35:01~C*04:01 | 0.0057 |
|  |  | A*68:02~B*15:10~C*03:02 | 0.0029 | A*23:01~B*14:02~C*08:02 | 0.0053 | A*33:01~B*53:01~C*04:01 | 0.0022 | A*29:02~B*42:01~C*17:01 | 0.0131 | A*02:01~B*56:01~C*01:02 | 0.0023 | A*03:01~B*35:01~C*06:02 | 0.0008 |
|  |  | A*68:02~B*15:10~C*03:04 | 0.0087 | A*23:01~B*15:01~C*04:01 | 0.0006 | A*33:01~B*81:01~C*18:01 | 0.0022 | A*29:02~B*42:02~C*17:01 | 0.0009 | A*02:01~B*57:01~C*06:02 | 0.0223 | A*03:01~B*35:01~C*16:01 | 0.0008 |
|  |  | A*68:02~B*15:16~C*03:04 | 0.0029 | A*23:01~B*15:03~C*02:10 | 0.0093 | A*33:03~B*15:03~C*02:10 | 0.0043 | A*29:02~B*44:03~C*03:04 | 0.0009 | A*02:01~B*57:03~C*07:01 | 0.0006 | A*03:01~B*35:03~C*04:01 | 0.0008 |
|  |  | A*68:02~B*39:10~C*14:02 | 0.0029 | A*23:01~B*15:03~C*04:01 | 0.0003 | A*33:03~B*15:31~C*03:02 | 0.0022 | A*29:02~B*44:03~C*07:01 | 0.0085 | A*02:01~B*57:03~C*18:00 | 0.0003 | A*03:01~B*39:10~C*12:03 | 0.0033 |
|  |  | A*68:02~B*42:01~C*17:01 | 0.0029 | A*23:01~B*15:10~C*03:04 | 0.0007 | A*33:03~B*53:01~C*04:01 | 0.0065 | A*29:02~B*45:01~C*06:02 | 0.0085 | A*02:01~B*58:01~C*03:02 | 0.0003 | A*03:01~B*39:20~C*12:03 | 0.0008 |
|  |  | A*68:02~B*44:15~C*04:07 | 0.0029 | A*23:01~B*15:10~C*04:01 | 0.0012 | A*34:02~B*15:16~C*14:02 | 0.0022 | A*29:02~B*45:01~C*16:01 | 0.0009 | A*02:01~B*58:01~C*07:01 | 0.0021 | A*03:01~B*40:01~C*03:04 | 0.0038 |
|  |  | A*68:02~B*49:01~C*07:01 | 0.0123 | A*23:01~B*15:10~C*16:01 | 0.0215 | A*34:02~B*18:01~C*07:04 | 0.0035 | A*29:02~B*53:01~C*04:01 | 0.0023 | A*02:02~B*40:02~C*02:02 | 0.0003 | A*03:01~B*40:02~C*02:02 | 0.0015 |
|  |  | A*68:02~B*53:01~C*04:01 | 0.0058 | A*23:01~B*18:01~C*02:02 | 0.0003 | A*34:02~B*27:03~C*02:02 | 0.0022 | A*29:02~B*57:03~C*18:02 | 0.0027 | A*02:02~B*41:01~C*17:00 | 0.0003 | A*03:01~B*40:02~C*03:05 | 0.0008 |
|  |  | A*68:02~B*58:01~C*07:01 | 0.0067 | A*23:01~B*18:01~C*07:04 | 0.0004 | A*34:02~B*35:01~C*04:01 | 0.0043 | A*29:02~B*58:01~C*04:01 | 0.0009 | A*02:02~B*41:01~C*17:01 | 0.0003 | A*03:01~B*41:02~C*17:01 | 0.0008 |
|  |  | A*68:02~B*81:01~C*18:01 | 0.0029 | A*23:01~B*35:01~C*04:01 | 0.0002 | A*34:02~B*42:02~C*07:01 | 0.0022 | A*29:02~B*58:01~C*06:02 | 0.0018 | A*02:02~B*53:01~C*06:02 | 0.0003 | A*03:01~B*42:01~C*12:02 | 0.0008 |
|  |  | A*74:01~B*15:03~C*02:10 | 0.0266 | A*23:01~B*35:02~C*04:01 | 0.0004 | A*34:02~B*44:03~C*04:01 | 0.0022 | A*29:02~B*58:01~C*07:01 | 0.0018 | A*02:02~B*57:03~C*07:01 | 0.0006 | A*03:01~B*44:03~C*04:01 | 0.0058 |
|  |  | A*74:01~B*15:10~C*03:04 | 0.0029 | A*23:01~B*39:10~C*12:03 | 0.0006 | A*34:02~B*44:15~C*04:07 | 0.0022 | A*29:02~B*58:02~C*06:02 | 0.0009 | A*02:04~B*51:01~C*15:02 | 0.0014 | A*03:01~B*44:03~C*07:01 | 0.0013 |
|  |  | A*74:01~B*35:01~C*04:01 | 0.0116 | A*23:01~B*39:10~C*15:05 | 0.0021 | A*34:02~B*47:01~C*02:10 | 0.0022 | A*29:02~B*81:01~C*18:01 | 0.0009 | A*02:04~B*57:01~C*03:04 | 0.0003 | A*03:01~B*44:03~C*14:03 | 0.0008 |
|  |  | A*74:01~B*42:01~C*17:01 | 0.0029 | A*23:01~B*41:01~C*17:00 | 0.0012 | A*34:02~B*53:01~C*04:01 | 0.0032 | A*30:01~B*07:02~C*07:02 | 0.0034 | A*02:05~B*14:02~C*08:02 | 0.0006 | A*03:01~B*44:05~C*02:02 | 0.0008 |
|  |  | A*74:01~B*44:03~C*07:01 | 0.0029 | A*23:01~B*41:01~C*17:01 | 0.0023 | A*34:02~B*81:01~C*08:02 | 0.0019 | A*30:01~B*13:02~C*03:04 | 0.0009 | A*02:05~B*35:02~C*04:01 | 0.0003 | A*03:01~B*47:01~C*06:02 | 0.0008 |
|  |  | A*74:01~B*49:01~C*07:01 | 0.0231 | A*23:01~B*42:01~C*17:00 | 0.0012 | A*34:02~B*82:02~C*03:02 | 0.0022 | A*30:01~B*15:03~C*02:02 | 0.0009 | A*02:05~B*35:43~C*01:02 | 0.0006 | A*03:01~B*49:01~C*07:01 | 0.0042 |
|  |  | A*74:01~B*51:01~C*16:01 | 0.0029 | A*23:01~B*42:01~C*17:01 | 0.0022 | A*36:01~B*15:03~C*04:01 | 0.0022 | A*30:01~B*15:03~C*02:10 | 0.0055 | A*02:05~B*39:05~C*12:03 | 0.0003 | A*03:01~B*51:01~C*14:02 | 0.0023 |
|  |  | A*74:01~B*57:03~C*17:01 | 0.0029 | A*23:01~B*42:02~C*17:01 | 0.0003 | A*36:01~B*49:01~C*06:02 | 0.0022 | A*30:01~B*15:10~C*03:04 | 0.0053 | A*02:05~B*40:01~C*03:09 | 0.0003 | A*03:01~B*51:01~C*17:01 | 0.0008 |
|  |  | A*74:01~B*58:01~C*06:02 | 0.0030 | A*23:01~B*44:03~C*02:10 | 0.0010 | A*36:01~B*49:01~C*07:01 | 0.0065 | A*30:01~B*15:47~C*02:10 | 0.0027 | A*02:05~B*41:02~C*17:03 | 0.0003 | A*03:01~B*52:01~C*16:01 | 0.0023 |
|  |  | A*74:01~B*58:02~C*06:02 | 0.0109 | A*23:01~B*44:03~C*03:03 | 0.0046 | A*36:01~B*53:01~C*04:01 | 0.0300 | A*30:01~B*18:01~C*03:04 | 0.0009 | A*02:05~B*44:03~C*16:01 | 0.0006 | A*03:01~B*53:01~C*04:01 | 0.0025 |
|  |  | A*74:02~B*51:01~C*16:02 | 0.0029 | A*23:01~B*44:03~C*04:01 | 0.0027 | A*36:01~B*81:01~C*08:02 | 0.0024 | A*30:01~B*35:01~C*17:01 | 0.0009 | A*02:05~B*49:01~C*07:01 | 0.0017 | A*03:01~B*57:01~C*06:02 | 0.0053 |
|  |  | A*74:05~B*58:02~C*02:10 | 0.0029 | A*23:01~B*45:01~C*06:02 | 0.0046 | A*66:01~B*15:03~C*02:10 | 0.0043 | A*30:01~B*39:10~C*12:03 | 0.0009 | A*02:05~B*50:01~C*06:02 | 0.0017 | A*03:01~B*57:02~C*18:00 | 0.0026 |
|  |  | A*80:01~B*15:10~C*03:02 | 0.0029 | A*23:01~B*45:01~C*18:00 | 0.0003 | A*66:01~B*18:01~C*07:04 | 0.0043 | A*30:01~B*40:16~C*17:01 | 0.0018 | A*02:05~B*51:01~C*16:02 | 0.0003 | A*03:01~B*57:02~C*18:02 | 0.0008 |
|  |  |  |  | A*23:01~B*53:01~C*03:04 | 0.0012 | A*66:01~B*39:10~C*12:03 | 0.0022 | A*30:01~B*42:01~C*07:01 | 0.0018 | A*02:05~B*53:01~C*15:05 | 0.0003 | A*03:01~B*57:03~C*07:01 | 0.0057 |
|  |  |  |  | A*23:01~B*53:01~C*04:01 | 0.0005 | A*66:01~B*44:03~C*14:02 | 0.0022 | A*30:01~B*42:01~C*17:01 | 0.0463 | A*02:05~B*56:01~C*01:02 | 0.0003 | A*03:01~B*57:03~C*08:02 | 0.0004 |
|  |  |  |  | A*23:01~B*53:01~C*06:02 | 0.0006 | A*66:01~B*44:03~C*14:03 | 0.0022 | A*30:01~B*42:02~C*17:01 | 0.0062 | A*02:05~B*57:03~C*07:01 | 0.0003 | A*03:01~B*57:03~C*18:02 | 0.0009 |
|  |  |  |  | A*23:01~B*53:01~C*08:02 | 0.0003 | A*66:01~B*57:01~C*07:01 | 0.0022 | A*30:01~B*44:03~C*04:01 | 0.0011 | A*02:05~B*57:03~C*18:00 | 0.0003 | A*03:01~B*58:01~C*07:01 | 0.0017 |
|  |  |  |  | A*23:01~B*57:02~C*07:01 | 0.0027 | A*66:01~B*58:02~C*06:02 | 0.0130 | A*30:01~B*44:03~C*14:03 | 0.0009 | A*02:05~B*58:01~C*03:03 | 0.0003 | A*03:01~B*58:01~C*16:01 | 0.0008 |
|  |  |  |  | A*23:01~B*58:01~C*03:02 | 0.0027 | A*66:01~B*81:01~C*04:01 | 0.0022 | A*30:01~B*45:01~C*06:02 | 0.0021 | A*02:05~B*58:01~C*07:01 | 0.0017 | A*03:01~B*58:02~C*06:02 | 0.0008 |
|  |  |  |  | A*23:01~B*58:01~C*06:02 | 0.0015 | A*66:01~B*81:01~C*18:01 | 0.0022 | A*30:01~B*49:01~C*07:01 | 0.0009 | A*02:06~B*15:01~C*03:04 | 0.0003 | A*03:01~B*81:01~C*18:00 | 0.0013 |
|  |  |  |  | A*23:01~B*58:01~C*07:01 | 0.0017 | A*66:02~B*58:02~C*07:01 | 0.0022 | A*30:01~B*50:01~C*06:02 | 0.0009 | A*02:06~B*15:11~C*03:03 | 0.0003 | A*03:02~B*35:03~C*04:29 | 0.0008 |
|  |  |  |  | A*23:01~B*58:02~C*06:02 | 0.0054 | A*66:02~B*81:01~C*08:02 | 0.0022 | A*30:01~B*53:01~C*04:01 | 0.0027 | A*02:06~B*27:05~C*02:02 | 0.0003 | A*11:01~B*08:01~C*07:01 | 0.0008 |
|  |  |  |  | A*23:01~B*58:02~C*07:01 | 0.0003 | A*68:01~B*08:01~C*03:04 | 0.0022 | A*30:01~B*57:03~C*06:02 | 0.0009 | A*02:06~B*27:05~C*03:03 | 0.0011 | A*11:01~B*15:01~C*04:01 | 0.0008 |
|  |  |  |  | A*23:01~B*81:01~C*18:00 | 0.0006 | A*68:01~B*08:01~C*07:04 | 0.0022 | A*30:01~B*57:03~C*07:01 | 0.0009 | A*02:06~B*35:01~C*03:03 | 0.0003 | A*11:01~B*18:01~C*02:02 | 0.0015 |
|  |  |  |  | A*23:01~B*81:01~C*18:01 | 0.0003 | A*68:01~B*15:17~C*07:01 | 0.0043 | A*30:01~B*57:03~C*18:01 | 0.0017 | A*02:06~B*35:01~C*03:04 | 0.0003 | A*11:01~B*27:05~C*02:02 | 0.0008 |
|  |  |  |  | A*24:02~B*07:02~C*07:02 | 0.0182 | A*68:02~B*07:02~C*07:02 | 0.0280 | A*30:01~B*57:03~C*18:02 | 0.0009 | A*02:06~B*35:17~C*04:01 | 0.0003 | A*11:01~B*35:01~C*04:01 | 0.0038 |
|  |  |  |  | A*24:02~B*07:02~C*15:25 | 0.0006 | A*68:02~B*13:02~C*06:02 | 0.0045 | A*30:01~B*81:01~C*18:01 | 0.0016 | A*02:06~B*35:43~C*01:02 | 0.0003 | A*11:01~B*35:04~C*04:01 | 0.0008 |
|  |  |  |  | A*24:02~B*08:01~C*07:02 | 0.0015 | A*68:02~B*14:02~C*08:02 | 0.0077 | A*30:02~B*07:02~C*04:01 | 0.0009 | A*02:06~B*40:02~C*03:04 | 0.0003 | A*11:01~B*39:01~C*07:02 | 0.0008 |
|  |  |  |  | A*24:02~B*15:10~C*16:01 | 0.0006 | A*68:02~B*15:10~C*03:04 | 0.0144 | A*30:02~B*07:02~C*07:02 | 0.0018 | A*02:06~B*44:02~C*05:01 | 0.0003 | A*11:01~B*40:01~C*03:04 | 0.0008 |
|  |  |  |  | A*24:02~B*15:13~C*08:01 | 0.0003 | A*68:02~B*15:16~C*14:02 | 0.0022 | A*30:02~B*07:02~C*07:06 | 0.0009 | A*02:06~B*48:01~C*08:01 | 0.0006 | A*11:01~B*40:02~C*02:02 | 0.0008 |
|  |  |  |  | A*24:02~B*15:16~C*14:02 | 0.0003 | A*68:02~B*18:01~C*06:02 | 0.0022 | A*30:02~B*07:02~C*15:02 | 0.0009 | A*02:06~B*48:01~C*08:03 | 0.0003 | A*11:01~B*44:02~C*05:01 | 0.0030 |
|  |  |  |  | A*24:02~B*18:01~C*04:03 | 0.0003 | A*68:02~B*27:03~C*04:01 | 0.0022 | A*30:02~B*07:05~C*07:01 | 0.0009 | A*02:06~B*51:01~C*15:02 | 0.0006 | A*11:01~B*51:08~C*16:02 | 0.0008 |
|  |  |  |  | A*24:02~B*18:01~C*07:01 | 0.0003 | A*68:02~B*39:10~C*12:03 | 0.0022 | A*30:02~B*08:01~C*07:01 | 0.0150 | A*02:06~B*55:01~C*03:05 | 0.0003 | A*11:01~B*52:01~C*17:00 | 0.0008 |
|  |  |  |  | A*24:02~B*18:01~C*07:04 | 0.0003 | A*68:02~B*44:03~C*04:01 | 0.0022 | A*30:02~B*14:01~C*04:01 | 0.0009 | A*02:09~B*35:03~C*04:01 | 0.0003 | A*11:01~B*55:01~C*03:03 | 0.0008 |
|  |  |  |  | A*24:02~B*35:01~C*07:02 | 0.0003 | A*68:02~B*53:01~C*04:01 | 0.0169 | A*30:02~B*14:01~C*08:02 | 0.0018 | A*02:09~B*58:01~C*03:02 | 0.0003 | A*11:02~B*40:01~C*07:02 | 0.0008 |
|  |  |  |  | A*24:02~B*35:02~C*04:01 | 0.0003 | A*68:02~B*57:02~C*18:01 | 0.0043 | A*30:02~B*14:02~C*08:02 | 0.0176 | A*02:11~B*45:01~C*16:01 | 0.0003 | A*23:01~B*07:02~C*07:02 | 0.0054 |
|  |  |  |  | A*24:02~B*38:02~C*07:02 | 0.0003 | A*68:02~B*57:03~C*07:01 | 0.0043 | A*30:02~B*14:03~C*08:02 | 0.0009 | A*02:11~B*52:01~C*12:02 | 0.0006 | A*23:01~B*07:02~C*15:05 | 0.0008 |
|  |  |  |  | A*24:02~B*40:06~C*07:04 | 0.0006 | A*68:02~B*58:01~C*07:01 | 0.0043 | A*30:02~B*15:03~C*07:01 | 0.0009 | A*02:11~B*52:01~C*15:02 | 0.0008 | A*23:01~B*08:01~C*03:04 | 0.0015 |
|  |  |  |  | A*24:02~B*42:01~C*17:01 | 0.0006 | A*68:02~B*58:02~C*06:02 | 0.0022 | A*30:02~B*15:10~C*03:04 | 0.0018 | A*02:11~B*52:02~C*15:02 | 0.0003 | A*23:01~B*08:01~C*07:02 | 0.0008 |
|  |  |  |  | A*24:02~B*44:03~C*04:01 | 0.0006 | A*74:01~B*15:03~C*02:10 | 0.0173 | A*30:02~B*18:01~C*02:02 | 0.0018 | A*02:11~B*57:01~C*07:01 | 0.0003 | A*23:01~B*14:01~C*06:02 | 0.0015 |
|  |  |  |  | A*24:02~B*51:01~C*15:02 | 0.0003 | A*74:01~B*15:17~C*07:01 | 0.0022 | A*30:02~B*18:01~C*04:01 | 0.0009 | A*02:17~B*15:40~C*03:03 | 0.0003 | A*23:01~B*14:02~C*08:02 | 0.0077 |
|  |  |  |  | A*24:02~B*51:01~C*16:01 | 0.0003 | A*74:01~B*35:01~C*04:01 | 0.0043 | A*30:02~B*18:01~C*07:04 | 0.0104 | A*02:17~B*18:01~C*12:03 | 0.0003 | A*23:01~B*14:02~C*08:43 | 0.0008 |
|  |  |  |  | A*24:02~B*55:01~C*01:02 | 0.0006 | A*74:01~B*49:01~C*07:01 | 0.0216 | A*30:02~B*35:01~C*17:01 | 0.0008 | A*02:17~B*51:01~C*15:02 | 0.0006 | A*23:01~B*14:02~C*18:00 | 0.0015 |
|  |  |  |  | A*24:02~B*58:01~C*07:02 | 0.0003 | A*74:01~B*53:01~C*02:02 | 0.0022 | A*30:02~B*39:10~C*12:03 | 0.0029 | A*02:20~B*27:05~C*01:02 | 0.0003 | A*23:01~B*14:03~C*08:02 | 0.0015 |
|  |  |  |  | A*24:03~B*07:02~C*07:02 | 0.0003 | A*74:01~B*57:03~C*08:02 | 0.0087 | A*30:02~B*41:01~C*16:01 | 0.0009 | A*02:22~B*41:01~C*07:01 | 0.0003 | A*23:01~B*15:03~C*02:10 | 0.0075 |
|  |  |  |  | A*24:07~B*44:03~C*07:01 | 0.0003 | A*74:01~B*58:01~C*04:01 | 0.0022 | A*30:02~B*42:01~C*17:01 | 0.0026 | A*02:274~B*51:01~C*02:02 | 0.0003 | A*23:01~B*15:10~C*16:01 | 0.0015 |
|  |  |  |  | A*26:01~B*07:04~C*07:02 | 0.0003 | A*74:01~B*58:01~C*06:02 | 0.0087 | A*30:02~B*42:02~C*17:01 | 0.0010 | A*02:30~B*38:01~C*12:03 | 0.0003 | A*23:01~B*15:16~C*16:01 | 0.0008 |
|  |  |  |  | A*26:01~B*07:05~C*07:02 | 0.0021 | A*74:01~B*58:02~C*06:02 | 0.0152 | A*30:02~B*45:01~C*02:10 | 0.0009 | A*02:724~B*15:03~C*05:01 | 0.0003 | A*23:01~B*15:17~C*05:01 | 0.0008 |
|  |  |  |  | A*26:01~B*08:01~C*02:10 | 0.0015 | A*74:01~B*81:01~C*08:02 | 0.0022 | A*30:02~B*45:01~C*16:01 | 0.0075 | A*03:01~B*07:02~C*07:02 | 0.0423 | A*23:01~B*27:03~C*02:02 | 0.0012 |
|  |  |  |  | A*26:01~B*14:01~C*08:02 | 0.0003 | A*74:03~B*57:02~C*04:01 | 0.0022 | A*30:02~B*49:01~C*07:01 | 0.0009 | A*03:01~B*07:04~C*07:02 | 0.0003 | A*23:01~B*27:05~C*02:02 | 0.0008 |
|  |  |  |  | A*26:01~B*14:01~C*08:04 | 0.0006 | A*80:01~B*18:01~C*02:02 | 0.0022 | A*30:02~B*51:01~C*16:01 | 0.0009 | A*03:01~B*07:05~C*15:05 | 0.0006 | A*23:01~B*35:01~C*04:01 | 0.0010 |
|  |  |  |  | A*26:01~B*15:03~C*07:01 | 0.0006 | A*80:01~B*58:01~C*03:02 | 0.0022 | A*30:02~B*53:01~C*03:04 | 0.0033 | A*03:01~B*08:01~C*07:01 | 0.0026 | A*23:01~B*39:01~C*15:02 | 0.0008 |
|  |  |  |  | A*26:01~B*15:03~C*17:01 | 0.0003 | A*80:01~B*58:01~C*06:02 | 0.0022 | A*30:02~B*53:01~C*04:01 | 0.0042 | A*03:01~B*08:01~C*07:02 | 0.0014 | A*23:01~B*42:01~C*17:01 | 0.0015 |
|  |  |  |  | A*26:01~B*15:22~C*17:01 | 0.0003 |  |  | A*30:02~B*57:02~C*18:01 | 0.0035 | A*03:01~B*13:02~C*06:02 | 0.0015 | A*23:01~B*44:03~C*04:01 | 0.0027 |
|  |  |  |  | A*26:01~B*39:10~C*12:03 | 0.0006 |  |  | A*30:02~B*57:02~C*18:02 | 0.0027 | A*03:01~B*14:01~C*08:02 | 0.0009 | A*23:01~B*44:03~C*07:01 | 0.0008 |
|  |  |  |  | A*26:01~B*41:01~C*17:01 | 0.0003 |  |  | A*30:02~B*57:03~C*07:01 | 0.0027 | A*03:01~B*14:02~C*01:02 | 0.0003 | A*23:01~B*44:10~C*04:01 | 0.0008 |
|  |  |  |  | A*26:01~B*42:01~C*06:02 | 0.0003 |  |  | A*30:02~B*57:03~C*18:01 | 0.0166 | A*03:01~B*14:02~C*08:02 | 0.0114 | A*23:01~B*45:01~C*06:02 | 0.0030 |
|  |  |  |  | A*26:01~B*42:01~C*17:00 | 0.0002 |  |  | A*30:02~B*57:03~C*18:02 | 0.0112 | A*03:01~B*14:02~C*15:05 | 0.0003 | A*23:01~B*49:01~C*03:04 | 0.0008 |
|  |  |  |  | A*26:01~B*51:01~C*07:01 | 0.0052 |  |  | A*30:02~B*58:01~C*03:02 | 0.0009 | A*03:01~B*15:01~C*03:03 | 0.0039 | A*23:01~B*50:01~C*06:02 | 0.0015 |
|  |  |  |  | A*26:01~B*58:01~C*06:02 | 0.0003 |  |  | A*30:02~B*58:01~C*06:02 | 0.0010 | A*03:01~B*15:01~C*03:04 | 0.0019 | A*23:01~B*50:01~C*17:01 | 0.0008 |
|  |  |  |  | A*26:01~B*58:01~C*07:01 | 0.0003 |  |  | A*30:02~B*58:01~C*07:01 | 0.0063 | A*03:01~B*15:01~C*07:04 | 0.0006 | A*23:01~B*51:64~C*16:01 | 0.0008 |
|  |  |  |  | A*26:01~B*58:02~C*06:02 | 0.0007 |  |  | A*30:02~B*81:01~C*18:01 | 0.0029 | A*03:01~B*15:10~C*03:04 | 0.0003 | A*23:01~B*52:01~C*16:01 | 0.0018 |
|  |  |  |  | A*26:01~B*81:00~C*04:01 | 0.0003 |  |  | A*30:04~B*15:03~C*02:10 | 0.0009 | A*03:01~B*15:18~C*07:04 | 0.0003 | A*23:01~B*53:01~C*04:01 | 0.0061 |
|  |  |  |  | A*26:01~B*81:01~C*04:01 | 0.0052 |  |  | A*30:04~B*15:83~C*15:25 | 0.0009 | A*03:01~B*18:01~C*05:01 | 0.0009 | A*23:01~B*53:01~C*06:02 | 0.0009 |
|  |  |  |  | A*26:01~B*81:03~C*04:01 | 0.0003 |  |  | A*30:04~B*18:01~C*07:04 | 0.0009 | A*03:01~B*18:01~C*07:01 | 0.0025 | A*23:01~B*53:01~C*08:02 | 0.0015 |
|  |  |  |  | A*26:12~B*41:01~C*07:01 | 0.0015 |  |  | A*30:04~B*53:01~C*06:02 | 0.0009 | A*03:01~B*18:01~C*12:03 | 0.0013 | A*23:01~B*53:01~C*16:01 | 0.0008 |
|  |  |  |  | A*26:12~B*51:01~C*16:01 | 0.0003 |  |  | A*30:04~B*58:02~C*06:02 | 0.0018 | A*03:01~B*18:05~C*12:03 | 0.0003 | A*23:01~B*57:03~C*07:01 | 0.0072 |
|  |  |  |  | A*26:121~B*15:10~C*04:01 | 0.0006 |  |  | A*30:04~B*58:02~C*17new | 0.0009 | A*03:01~B*27:02~C*07:02 | 0.0003 | A*23:01~B*57:03~C*18:02 | 0.0006 |
|  |  |  |  | A*29:01~B*14:02~C*08:02 | 0.0003 |  |  | A*30:04~B*73:01~C*15:05 | 0.0018 | A*03:01~B*27:05~C*01:02 | 0.0040 | A*23:01~B*58:01~C*03:02 | 0.0053 |
|  |  |  |  | A*29:01~B*15:03~C*04:01 | 0.0003 |  |  | A*30:09~B*15:10~C*03:04 | 0.0009 | A*03:01~B*27:05~C*02:02 | 0.0029 | A*23:01~B*58:02~C*06:02 | 0.0008 |
|  |  |  |  | A*29:01~B*15:22~C*04:01 | 0.0003 |  |  | A*30:09~B*18:01~C*02:10 | 0.0009 | A*03:01~B*35:01~C*02:02 | 0.0003 | A*23:01~B*81:01~C*08:04 | 0.0031 |
|  |  |  |  | A*29:01~B*18:01~C*07:04 | 0.0024 |  |  | A*30:09~B*45:01~C*06:02 | 0.0035 | A*03:01~B*35:01~C*04:01 | 0.0134 | A*23:01~B*81:01~C*18:00 | 0.0024 |
|  |  |  |  | A*29:01~B*44:03~C*04:01 | 0.0003 |  |  | A*30:09~B*53:01~C*06:02 | 0.0009 | A*03:01~B*35:02~C*12:03 | 0.0003 | A*24:02~B*13:02~C*06:02 | 0.0008 |
|  |  |  |  | A*29:01~B*44:03~C*08:04 | 0.0009 |  |  | A*30:09~B*81:01~C*04:01 | 0.0009 | A*03:01~B*35:03~C*04:01 | 0.0010 | A*24:02~B*13:02~C*08:02 | 0.0008 |
|  |  |  |  | A*29:01~B*58:01~C*06:02 | 0.0003 |  |  | A*30:09~B*81:01~C*07:01 | 0.0009 | A*03:01~B*35:03~C*12:03 | 0.0001 | A*24:02~B*15:01~C*03:03 | 0.0008 |
|  |  |  |  | A*29:02~B*07:02~C*07:02 | 0.0018 |  |  | A*31:01~B*18:01~C*06:02 | 0.0009 | A*03:01~B*35:42~C*02:10 | 0.0003 | A*24:02~B*15:07~C*03:03 | 0.0008 |
|  |  |  |  | A*29:02~B*07:05~C*07:02 | 0.0003 |  |  | A*31:01~B*41:02~C*04:07 | 0.0009 | A*03:01~B*37:01~C*06:02 | 0.0009 | A*24:02~B*15:10~C*03:04 | 0.0008 |
|  |  |  |  | A*29:02~B*08:01~C*03:04 | 0.0003 |  |  | A*31:04~B*44:03~C*04:01 | 0.0009 | A*03:01~B*37:01~C*07:02 | 0.0003 | A*24:02~B*18:01~C*12:03 | 0.0008 |
|  |  |  |  | A*29:02~B*13:02~C*06:02 | 0.0015 |  |  | A*32:01~B*15:16~C*14:02 | 0.0009 | A*03:01~B*38:01~C*12:03 | 0.0016 | A*24:02~B*35:08~C*04:01 | 0.0008 |
|  |  |  |  | A*29:02~B*14:02~C*08:02 | 0.0003 |  |  | A*32:01~B*39:10~C*12:03 | 0.0018 | A*03:01~B*39:06~C*07:02 | 0.0004 | A*24:02~B*37:01~C*07:01 | 0.0008 |
|  |  |  |  | A*29:02~B*15:03~C*02:10 | 0.0047 |  |  | A*32:01~B*45:01~C*06:02 | 0.0009 | A*03:01~B*40:01~C*03:04 | 0.0037 | A*24:02~B*39:05~C*15:02 | 0.0008 |
|  |  |  |  | A*29:02~B*15:03~C*04:01 | 0.0003 |  |  | A*33:01~B*07:02~C*07:02 | 0.0009 | A*03:01~B*40:02~C*02:02 | 0.0012 | A*24:02~B*40:02~C*03:04 | 0.0008 |
|  |  |  |  | A*29:02~B*15:10~C*03:04 | 0.0015 |  |  | A*33:01~B*15:10~C*03:04 | 0.0018 | A*03:01~B*40:08~C*03:04 | 0.0003 | A*24:02~B*40:06~C*12:03 | 0.0008 |
|  |  |  |  | A*29:02~B*15:10~C*04:01 | 0.0003 |  |  | A*33:01~B*15:16~C*14:02 | 0.0009 | A*03:01~B*41:02~C*17:03 | 0.0003 | A*24:02~B*41:02~C*17:00 | 0.0008 |
|  |  |  |  | A*29:02~B*15:10~C*07:01 | 0.0006 |  |  | A*33:01~B*35:01~C*02:10 | 0.0009 | A*03:01~B*44:02~C*05:01 | 0.0046 | A*24:02~B*41:02~C*17:01 | 0.0008 |
|  |  |  |  | A*29:02~B*15:10~C*08:04 | 0.0009 |  |  | A*33:01~B*42:01~C*17:01 | 0.0026 | A*03:01~B*44:03~C*03:04 | 0.0003 | A*24:02~B*42:02~C*18:00 | 0.0008 |
|  |  |  |  | A*29:02~B*15:10~C*16:01 | 0.0025 |  |  | A*33:01~B*45:01~C*16:01 | 0.0009 | A*03:01~B*44:03~C*04:01 | 0.0003 | A*24:02~B*44:02~C*05:01 | 0.0008 |
|  |  |  |  | A*29:02~B*15:220~C*04:01 | 0.0003 |  |  | A*33:01~B*53:01~C*04:01 | 0.0017 | A*03:01~B*44:05~C*02:02 | 0.0002 | A*24:02~B*44:03~C*04:01 | 0.0009 |
|  |  |  |  | A*29:02~B*35:01~C*04:01 | 0.0003 |  |  | A*33:01~B*57:03~C*18:01 | 0.0010 | A*03:01~B*47:01~C*06:02 | 0.0004 | A*24:02~B*45:01~C*08:04 | 0.0008 |
|  |  |  |  | A*29:02~B*39:10~C*12:03 | 0.0014 |  |  | A*33:01~B*58:01~C*03:02 | 0.0009 | A*03:01~B*49:01~C*06:02 | 0.0003 | A*24:02~B*50:01~C*06:02 | 0.0008 |
|  |  |  |  | A*29:02~B*39:10~C*15:05 | 0.0003 |  |  | A*33:01~B*58:01~C*07:01 | 0.0009 | A*03:01~B*50:01~C*06:02 | 0.0009 | A*24:02~B*56:01~C*01:02 | 0.0008 |
|  |  |  |  | A*29:02~B*42:01~C*17:00 | 0.0059 |  |  | A*33:03~B*15:03~C*02:10 | 0.0011 | A*03:01~B*51:01~C*01:02 | 0.0014 | A*24:02~B*57:01~C*06:02 | 0.0008 |
|  |  |  |  | A*29:02~B*42:01~C*17:01 | 0.0082 |  |  | A*33:03~B*15:10~C*03:04 | 0.0027 | A*03:01~B*51:01~C*02:02 | 0.0006 | A*24:02~B*58:01~C*03:02 | 0.0010 |
|  |  |  |  | A*29:02~B*42:02~C*17:01 | 0.0004 |  |  | A*33:03~B*15:16~C*14:02 | 0.0009 | A*03:01~B*51:01~C*14:02 | 0.0012 | A*24:02~B*81:01~C*08:04 | 0.0011 |
|  |  |  |  | A*29:02~B*44:03~C*07:01 | 0.0251 |  |  | A*33:03~B*15:17~C*05:01 | 0.0018 | A*03:01~B*51:01~C*15:02 | 0.0017 | A*24:02~B*81:01~C*18:00 | 0.0008 |
|  |  |  |  | A*29:02~B*44:03~C*07:02 | 0.0003 |  |  | A*33:03~B*37:01~C*02:10 | 0.0009 | A*03:01~B*55:01~C*03:03 | 0.0002 | A*24:02~B*81:01~C*18:01 | 0.0008 |
|  |  |  |  | A*29:02~B*44:03~C*07:06 | 0.0033 |  |  | A*33:03~B*45:01~C*16:01 | 0.0009 | A*03:01~B*56:01~C*01:02 | 0.0007 | A*24:03~B*08:01~C*01:02 | 0.0008 |
|  |  |  |  | A*29:02~B*44:03~C*14:03 | 0.0003 |  |  | A*33:03~B*50:01~C*06:02 | 0.0018 | A*03:01~B*57:01~C*06:02 | 0.0062 | A*24:03~B*15:03~C*02:10 | 0.0008 |
|  |  |  |  | A*29:02~B*45:01~C*06:02 | 0.0036 |  |  | A*33:03~B*53:01~C*04:01 | 0.0015 | A*03:01~B*57:02~C*04:01 | 0.0003 | A*24:03~B*44:10~C*04:01 | 0.0008 |
|  |  |  |  | A*29:02~B*45:01~C*16:01 | 0.0007 |  |  | A*33:03~B*58:01~C*03:02 | 0.0009 | A*03:01~B*57:03~C*18:00 | 0.0003 | A*24:07~B*35:05~C*04:01 | 0.0008 |
|  |  |  |  | A*29:02~B*53:01~C*04:01 | 0.0003 |  |  | A*34:02~B*07:05~C*07:01 | 0.0009 | A*03:02~B*08:01~C*07:01 | 0.0008 | A*25:01~B*07:02~C*07:01 | 0.0008 |
|  |  |  |  | A*29:02~B*58:01~C*06:02 | 0.0004 |  |  | A*34:02~B*08:01~C*07:01 | 0.0017 | A*03:02~B*08:01~C*07:02 | 0.0003 | A*25:01~B*18:01~C*12:03 | 0.0030 |
|  |  |  |  | A*29:02~B*58:01~C*07:01 | 0.0003 |  |  | A*34:02~B*15:03~C*02:10 | 0.0015 | A*03:02~B*35:01~C*04:01 | 0.0003 | A*25:01~B*27:03~C*02:02 | 0.0008 |
|  |  |  |  | A*29:02~B*58:02~C*06:02 | 0.0023 |  |  | A*34:02~B*15:03~C*07:01 | 0.0009 | A*03:02~B*51:01~C*15:02 | 0.0008 | A*25:01~B*39:01~C*12:03 | 0.0008 |
|  |  |  |  | A*29:02~B*81:01~C*06:02 | 0.0003 |  |  | A*34:02~B*15:10~C*08:02 | 0.0003 | A*03:02~B*55:01~C*01:02 | 0.0003 | A*25:01~B*44:02~C*12:03 | 0.0008 |
|  |  |  |  | A*29:11~B*13:02~C*06:02 | 0.0122 |  |  | A*34:02~B*15:17~C*05:01 | 0.0035 | A*03:05~B*07:02~C*07:02 | 0.0003 | A*26:01~B*08:01~C*03:04 | 0.0023 |
|  |  |  |  | A*29:11~B*15:10~C*04:01 | 0.0009 |  |  | A*34:02~B*35:01~C*04:01 | 0.0013 | A*03:21N~B*57:02~C*02:02 | 0.0003 | A*26:01~B*15:01~C*03:03 | 0.0008 |
|  |  |  |  | A*29:11~B*41:01~C*17:01 | 0.0003 |  |  | A*34:02~B*35:01~C*06:02 | 0.0018 | A*03:49~B*27:05~C*03:03 | 0.0003 | A*26:01~B*27:02~C*02:02 | 0.0008 |
|  |  |  |  | A*29:11~B*44:03~C*07:01 | 0.0003 |  |  | A*34:02~B*40:16~C*08:02 | 0.0009 | A*11:01~B*07:02~C*07:02 | 0.0033 | A*26:01~B*27:05~C*06:08 | 0.0015 |
|  |  |  |  | A*29:11~B*57:03~C*03:04 | 0.0003 |  |  | A*34:02~B*42:01~C*17:01 | 0.0006 | A*11:01~B*07:05~C*15:05 | 0.0003 | A*26:01~B*37:01~C*06:02 | 0.0008 |
|  |  |  |  | A*30:01~B*07:02~C*07:02 | 0.0016 |  |  | A*34:02~B*44:03~C*04:01 | 0.0141 | A*11:01~B*14:02~C*08:02 | 0.0029 | A*26:01~B*39:10~C*12:03 | 0.0008 |
|  |  |  |  | A*30:01~B*08:01~C*07:01 | 0.0010 |  |  | A*34:02~B*44:03~C*07:01 | 0.0035 | A*11:01~B*15:01~C*01:02 | 0.0011 | A*26:01~B*44:03~C*18:00 | 0.0008 |
|  |  |  |  | A*30:01~B*08:01~C*07:02 | 0.0009 |  |  | A*34:02~B*44:03~C*07:06 | 0.0035 | A*11:01~B*15:01~C*04:01 | 0.0009 | A*26:01~B*52:01~C*04:01 | 0.0008 |
|  |  |  |  | A*30:01~B*13:02~C*06:02 | 0.0003 |  |  | A*34:02~B*53:01~C*04:01 | 0.0027 | A*11:01~B*15:01~C*12:03 | 0.0006 | A*26:01~B*57:02~C*18:02 | 0.0008 |
|  |  |  |  | A*30:01~B*14:01~C*08:04 | 0.0003 |  |  | A*34:02~B*58:01~C*03:02 | 0.0009 | A*11:01~B*15:02~C*08:01 | 0.0006 | A*26:01~B*57:03~C*08:13 | 0.0008 |
|  |  |  |  | A*30:01~B*15:03~C*02:10 | 0.0086 |  |  | A*34:02~B*58:02~C*06:02 | 0.0018 | A*11:01~B*15:03~C*02:10 | 0.0003 | A*26:01~B*58:01~C*07:01 | 0.0008 |
|  |  |  |  | A*30:01~B*15:03~C*18:00 | 0.0003 |  |  | A*34:02~B*58:15~C*06:02 | 0.0009 | A*11:01~B*15:17~C*07:01 | 0.0003 | A*26:01~B*58:11~C*03:02 | 0.0008 |
|  |  |  |  | A*30:01~B*15:10~C*16:01 | 0.0006 |  |  | A*34:02~B*73:01~C*15:05 | 0.0009 | A*11:01~B*15:18~C*07:04 | 0.0003 | A*26:01~B*81:01~C*01:02 | 0.0008 |
|  |  |  |  | A*30:01~B*15:16~C*03:04 | 0.0003 |  |  | A*34:02~B*81:01~C*08:04 | 0.0009 | A*11:01~B*18:01~C*05:01 | 0.0007 | A*29:01~B*55:01~C*03:03 | 0.0008 |
|  |  |  |  | A*30:01~B*15:17~C*05:01 | 0.0009 |  |  | A*36:01~B*07:02~C*07:02 | 0.0015 | A*11:01~B*18:01~C*07:01 | 0.0022 | A*29:02~B*07:02~C*07:02 | 0.0008 |
|  |  |  |  | A*30:01~B*18:01~C*02:02 | 0.0022 |  |  | A*36:01~B*14:01~C*02:10 | 0.0009 | A*11:01~B*27:02~C*15:02 | 0.0003 | A*29:02~B*07:02~C*15:05 | 0.0021 |
|  |  |  |  | A*30:01~B*18:01~C*07:04 | 0.0035 |  |  | A*36:01~B*15:10~C*03:04 | 0.0021 | A*11:01~B*27:05~C*01:02 | 0.0023 | A*29:02~B*15:01~C*03:04 | 0.0015 |
|  |  |  |  | A*30:01~B*18:01~C*17:00 | 0.0003 |  |  | A*36:01~B*15:220~C*04:01 | 0.0009 | A*11:01~B*27:05~C*02:02 | 0.0026 | A*29:02~B*15:03~C*02:10 | 0.0019 |
|  |  |  |  | A*30:01~B*39:10~C*12:03 | 0.0007 |  |  | A*36:01~B*35:01~C*04:01 | 0.0013 | A*11:01~B*27:13~C*01:02 | 0.0003 | A*29:02~B*15:16~C*14:02 | 0.0008 |
|  |  |  |  | A*30:01~B*39:10~C*17:01 | 0.0006 |  |  | A*36:01~B*42:01~C*17:01 | 0.0025 | A*11:01~B*35:01~C*03:03 | 0.0003 | A*29:02~B*15:31~C*04:27 | 0.0008 |
|  |  |  |  | A*30:01~B*42:01~C*17:00 | 0.0190 |  |  | A*36:01~B*44:03~C*03:03 | 0.0010 | A*11:01~B*35:01~C*04:01 | 0.0124 | A*29:02~B*35:01~C*01:02 | 0.0008 |
|  |  |  |  | A*30:01~B*42:01~C*17:01 | 0.0275 |  |  | A*36:01~B*45:01~C*06:02 | 0.0009 | A*11:01~B*35:02~C*04:08 | 0.0003 | A*29:02~B*35:01~C*04:01 | 0.0017 |
|  |  |  |  | A*30:01~B*42:02~C*17:00 | 0.0070 |  |  | A*36:01~B*45:01~C*16:01 | 0.0009 | A*11:01~B*35:03~C*04:01 | 0.0016 | A*29:02~B*39:10~C*12:03 | 0.0015 |
|  |  |  |  | A*30:01~B*42:02~C*17:01 | 0.0075 |  |  | A*36:01~B*45:07~C*16:01 | 0.0009 | A*11:01~B*35:03~C*12:03 | 0.0010 | A*29:02~B*41:01~C*07:01 | 0.0015 |
|  |  |  |  | A*30:01~B*44:03~C*04:01 | 0.0016 |  |  | A*36:01~B*49:01~C*07:01 | 0.0009 | A*11:01~B*35:08~C*04:01 | 0.0009 | A*29:02~B*44:03~C*14:03 | 0.0008 |
|  |  |  |  | A*30:01~B*44:03~C*07:01 | 0.0011 |  |  | A*36:01~B*51:01~C*16:01 | 0.0018 | A*11:01~B*37:01~C*06:02 | 0.0008 | A*29:02~B*44:03~C*16:01 | 0.0045 |
|  |  |  |  | A*30:01~B*44:03~C*07:06 | 0.0003 |  |  | A*36:01~B*53:01~C*04:01 | 0.0278 | A*11:01~B*38:01~C*12:03 | 0.0017 | A*29:02~B*49:01~C*07:01 | 0.0038 |
|  |  |  |  | A*30:01~B*44:03~C*17:01 | 0.0003 |  |  | A*36:01~B*58:02~C*06:02 | 0.0017 | A*11:01~B*39:01~C*12:03 | 0.0008 | A*29:02~B*50:01~C*04:01 | 0.0008 |
|  |  |  |  | A*30:01~B*45:01~C*06:02 | 0.0009 |  |  | A*36:01~B*81:01~C*18:01 | 0.0009 | A*11:01~B*39:10~C*12:03 | 0.0003 | A*29:02~B*52:01~C*02:02 | 0.0008 |
|  |  |  |  | A*30:01~B*53:01~C*03:04 | 0.0003 |  |  | A*43:01~B*42:01~C*17:01 | 0.0009 | A*11:01~B*39:24~C*07:01 | 0.0003 | A*29:02~B*53:01~C*16:01 | 0.0008 |
|  |  |  |  | A*30:01~B*53:01~C*04:01 | 0.0003 |  |  | A*43:01~B*57:03~C*06:02 | 0.0009 | A*11:01~B*40:01~C*03:04 | 0.0004 | A*29:02~B*57:02~C*18:02 | 0.0008 |
|  |  |  |  | A*30:01~B*57:03~C*07:01 | 0.0012 |  |  | A*66:01~B*15:10~C*03:04 | 0.0009 | A*11:01~B*40:01~C*07:02 | 0.0003 | A*29:02~B*57:03~C*07:01 | 0.0008 |
|  |  |  |  | A*30:01~B*57:03~C*18:00 | 0.0003 |  |  | A*66:01~B*18:01~C*05:01 | 0.0009 | A*11:01~B*40:02~C*01:02 | 0.0003 | A*29:02~B*81:01~C*08:04 | 0.0023 |
|  |  |  |  | A*30:01~B*57:03~C*18:02 | 0.0006 |  |  | A*66:01~B*39:10~C*12:03 | 0.0024 | A*11:01~B*40:02~C*02:02 | 0.0005 | A*29:02~B*81:01~C*18:00 | 0.0004 |
|  |  |  |  | A*30:01~B*58:01~C*03:02 | 0.0023 |  |  | A*66:01~B*39:10~C*17:01 | 0.0009 | A*11:01~B*41:02~C*17:03 | 0.0003 | A*29:02~B*81:01~C*18:01 | 0.0030 |
|  |  |  |  | A*30:01~B*58:01~C*07:01 | 0.0009 |  |  | A*66:01~B*41:01~C*17:01 | 0.0009 | A*11:01~B*44:02~C*03:03 | 0.0006 | A*30:01~B*07:02~C*04:01 | 0.0008 |
|  |  |  |  | A*30:01~B*58:02~C*06:02 | 0.0026 |  |  | A*66:01~B*44:03~C*04:01 | 0.0026 | A*11:01~B*44:02~C*04:01 | 0.0003 | A*30:01~B*07:02~C*07:02 | 0.0045 |
|  |  |  |  | A*30:01~B*81:00~C*04:01 | 0.0006 |  |  | A*66:01~B*44:03~C*14:02 | 0.0009 | A*11:01~B*44:02~C*05:01 | 0.0026 | A*30:01~B*07:05~C*07:02 | 0.0008 |
|  |  |  |  | A*30:01~B*81:01~C*04:01 | 0.0027 |  |  | A*66:01~B*44:03~C*14:03 | 0.0009 | A*11:01~B*44:02~C*07:04 | 0.0004 | A*30:01~B*08:01~C*03:04 | 0.0015 |
|  |  |  |  | A*30:01~B*81:01~C*18:00 | 0.0003 |  |  | A*66:01~B*45:01~C*16:01 | 0.0009 | A*11:01~B*44:03~C*02:02 | 0.0003 | A*30:01~B*13:02~C*05:01 | 0.0008 |
|  |  |  |  | A*30:01~B*82:02~C*03:02 | 0.0001 |  |  | A*66:01~B*45:01~C*17:01 | 0.0009 | A*11:01~B*44:03~C*04:01 | 0.0003 | A*30:01~B*13:02~C*06:02 | 0.0008 |
|  |  |  |  | A*30:02~B*07:02~C*07:01 | 0.0006 |  |  | A*66:01~B*49:01~C*07:01 | 0.0009 | A*11:01~B*44:03~C*07:01 | 0.0006 | A*30:01~B*14:02~C*08:02 | 0.0008 |
|  |  |  |  | A*30:02~B*07:02~C*07:02 | 0.0029 |  |  | A*66:01~B*51:01~C*14:02 | 0.0035 | A*11:01~B*44:03~C*12:03 | 0.0003 | A*30:01~B*15:03~C*02:10 | 0.0012 |
|  |  |  |  | A*30:02~B*08:01~C*02:17 | 0.0003 |  |  | A*66:01~B*53:01~C*03:04 | 0.0012 | A*11:01~B*44:03~C*16:01 | 0.0003 | A*30:01~B*15:16~C*14:02 | 0.0008 |
|  |  |  |  | A*30:02~B*08:01~C*07:01 | 0.0083 |  |  | A*66:01~B*53:01~C*04:01 | 0.0027 | A*11:01~B*49:01~C*07:01 | 0.0008 | A*30:01~B*18:01~C*18:00 | 0.0008 |
|  |  |  |  | A*30:02~B*08:01~C*07:02 | 0.0003 |  |  | A*66:01~B*53:01~C*06:02 | 0.0012 | A*11:01~B*50:02~C*06:02 | 0.0003 | A*30:01~B*35:01~C*04:01 | 0.0008 |
|  |  |  |  | A*30:02~B*14:01~C*08:02 | 0.0003 |  |  | A*66:01~B*57:03~C*07:01 | 0.0006 | A*11:01~B*51:01~C*03:03 | 0.0003 | A*30:01~B*42:01~C*17:00 | 0.0045 |
|  |  |  |  | A*30:02~B*14:02~C*08:02 | 0.0029 |  |  | A*66:01~B*58:01~C*03:02 | 0.0009 | A*11:01~B*51:01~C*04:01 | 0.0006 | A*30:01~B*42:01~C*17:01 | 0.0127 |
|  |  |  |  | A*30:02~B*15:03~C*02:10 | 0.0034 |  |  | A*66:01~B*58:02~C*06:02 | 0.0097 | A*11:01~B*51:01~C*14:02 | 0.0012 | A*30:01~B*42:02~C*17:00 | 0.0015 |
|  |  |  |  | A*30:02~B*15:10~C*02:10 | 0.0003 |  |  | A*66:02~B*18:01~C*02:10 | 0.0009 | A*11:01~B*51:01~C*15:02 | 0.0025 | A*30:01~B*42:02~C*17:01 | 0.0015 |
|  |  |  |  | A*30:02~B*15:10~C*03:04 | 0.0032 |  |  | A*66:02~B*58:01~C*07:01 | 0.0018 | A*11:01~B*51:07~C*14:02 | 0.0006 | A*30:01~B*44:03~C*04:01 | 0.0008 |
|  |  |  |  | A*30:02~B*15:10~C*08:02 | 0.0003 |  |  | A*66:03~B*57:03~C*18:01 | 0.0018 | A*11:01~B*52:01~C*12:02 | 0.0045 | A*30:01~B*44:03~C*07:02 | 0.0008 |
|  |  |  |  | A*30:02~B*18:01~C*05:01 | 0.0003 |  |  | A*68:01~B*08:01~C*07:01 | 0.0009 | A*11:01~B*53:01~C*04:01 | 0.0006 | A*30:01~B*45:01~C*04:07 | 0.0008 |
|  |  |  |  | A*30:02~B*18:01~C*07:04 | 0.0054 |  |  | A*68:01~B*14:02~C*08:02 | 0.0018 | A*11:01~B*55:01~C*03:03 | 0.0016 | A*30:01~B*45:01~C*16:01 | 0.0018 |
|  |  |  |  | A*30:02~B*39:10~C*12:03 | 0.0077 |  |  | A*68:01~B*14:03~C*08:02 | 0.0009 | A*11:01~B*56:01~C*01:02 | 0.0006 | A*30:01~B*52:01~C*16:01 | 0.0016 |
|  |  |  |  | A*30:02~B*42:01~C*17:00 | 0.0043 |  |  | A*68:01~B*15:03~C*02:10 | 0.0009 | A*11:01~B*57:01~C*01:02 | 0.0006 | A*30:01~B*57:03~C*04:01 | 0.0008 |
|  |  |  |  | A*30:02~B*42:01~C*17:01 | 0.0051 |  |  | A*68:01~B*15:10~C*03:04 | 0.0009 | A*11:01~B*57:01~C*06:02 | 0.0021 | A*30:01~B*57:03~C*18:00 | 0.0045 |
|  |  |  |  | A*30:02~B*42:02~C*17:01 | 0.0003 |  |  | A*68:01~B*35:01~C*04:01 | 0.0035 | A*11:01~B*57:01~C*16:01 | 0.0003 | A*30:01~B*57:03~C*18:02 | 0.0060 |
|  |  |  |  | A*30:02~B*44:03~C*02:10 | 0.0003 |  |  | A*68:01~B*40:16~C*08:02 | 0.0027 | A*11:01~B*58:01~C*07:01 | 0.0006 | A*30:01~B*58:01~C*07:01 | 0.0008 |
|  |  |  |  | A*30:02~B*44:03~C*03:03 | 0.0003 |  |  | A*68:01~B*58:02~C*06:02 | 0.0009 | A*11:01~B*58:01~C*12:03 | 0.0003 | A*30:01~B*81:01~C*08:04 | 0.0008 |
|  |  |  |  | A*30:02~B*44:03~C*07:01 | 0.0006 |  |  | A*68:02~B*07:02~C*06:02 | 0.0009 | A*11:04~B*15:01~C*04:01 | 0.0003 | A*30:02~B*07:02~C*07:02 | 0.0020 |
|  |  |  |  | A*30:02~B*45:01~C*16:01 | 0.0087 |  |  | A*68:02~B*07:02~C*07:02 | 0.0111 | A*11:04~B*35:01~C*04:01 | 0.0003 | A*30:02~B*07:02~C*15:05 | 0.0009 |
|  |  |  |  | A*30:02~B*53:01~C*03:04 | 0.0005 |  |  | A*68:02~B*07:51~C*07:02 | 0.0009 | A*11:67~B*08:01~C*07:01 | 0.0003 | A*30:02~B*08:01~C*07:01 | 0.0044 |
|  |  |  |  | A*30:02~B*53:01~C*04:01 | 0.0002 |  |  | A*68:02~B*13:02~C*04:01 | 0.0009 | A*23:01~B*07:02~C*07:02 | 0.0006 | A*30:02~B*14:01~C*05:01 | 0.0008 |
|  |  |  |  | A*30:02~B*57:02~C*18:00 | 0.0006 |  |  | A*68:02~B*14:01~C*08:02 | 0.0159 | A*23:01~B*07:02~C*12:03 | 0.0003 | A*30:02~B*14:02~C*08:02 | 0.0029 |
|  |  |  |  | A*30:02~B*57:02~C*18:02 | 0.0003 |  |  | A*68:02~B*15:03~C*02:10 | 0.0016 | A*23:01~B*07:05~C*15:05 | 0.0003 | A*30:02~B*15:03~C*02:10 | 0.0053 |
|  |  |  |  | A*30:02~B*57:03~C*18:00 | 0.0015 |  |  | A*68:02~B*15:03~C*04:01 | 0.0018 | A*23:01~B*08:01~C*07:01 | 0.0003 | A*30:02~B*18:01~C*05:01 | 0.0023 |
|  |  |  |  | A*30:02~B*57:03~C*18:01 | 0.0003 |  |  | A*68:02~B*15:03~C*12:03 | 0.0009 | A*23:01~B*14:02~C*08:02 | 0.0003 | A*30:02~B*18:01~C*07:04 | 0.0015 |
|  |  |  |  | A*30:02~B*57:03~C*18:02 | 0.0006 |  |  | A*68:02~B*15:03~C*16:01 | 0.0009 | A*23:01~B*15:03~C*02:10 | 0.0006 | A*30:02~B*27:03~C*02:02 | 0.0003 |
|  |  |  |  | A*30:02~B*58:01~C*07:01 | 0.0007 |  |  | A*68:02~B*15:10~C*03:04 | 0.0270 | A*23:01~B*15:24~C*03:03 | 0.0003 | A*30:02~B*27:03~C*07:01 | 0.0008 |
|  |  |  |  | A*30:02~B*58:02~C*06:02 | 0.0116 |  |  | A*68:02~B*15:10~C*16:01 | 0.0009 | A*23:01~B*18:01~C*12:03 | 0.0003 | A*30:02~B*35:01~C*04:01 | 0.0029 |
|  |  |  |  | A*30:02~B*81:00~C*03:02 | 0.0003 |  |  | A*68:02~B*18:01~C*04:01 | 0.0018 | A*23:01~B*27:05~C*05:01 | 0.0003 | A*30:02~B*35:01~C*16:01 | 0.0015 |
|  |  |  |  | A*30:03~B*44:03~C*07:01 | 0.0003 |  |  | A*68:02~B*18:01~C*05:01 | 0.0010 | A*23:01~B*35:01~C*04:01 | 0.0006 | A*30:02~B*39:10~C*12:03 | 0.0027 |
|  |  |  |  | A*30:04~B*07:02~C*02:10 | 0.0006 |  |  | A*68:02~B*18:01~C*07:04 | 0.0009 | A*23:01~B*37:01~C*04:01 | 0.0003 | A*30:02~B*40:16~C*08:02 | 0.0008 |
|  |  |  |  | A*30:04~B*07:02~C*07:02 | 0.0015 |  |  | A*68:02~B*35:01~C*04:01 | 0.0018 | A*23:01~B*38:01~C*04:01 | 0.0003 | A*30:02~B*42:01~C*07:01 | 0.0008 |
|  |  |  |  | A*30:04~B*07:05~C*07:02 | 0.0006 |  |  | A*68:02~B*39:10~C*12:03 | 0.0020 | A*23:01~B*39:01~C*07:02 | 0.0003 | A*30:02~B*42:01~C*17:01 | 0.0008 |
|  |  |  |  | A*30:04~B*08:01~C*02:05 | 0.0003 |  |  | A*68:02~B*42:01~C*03:04 | 0.0009 | A*23:01~B*41:01~C*17:01 | 0.0003 | A*30:02~B*44:03~C*06:02 | 0.0008 |
|  |  |  |  | A*30:04~B*08:01~C*02:17 | 0.0024 |  |  | A*68:02~B*42:01~C*17:01 | 0.0074 | A*23:01~B*41:02~C*17:00 | 0.0003 | A*30:02~B*44:03~C*14:03 | 0.0023 |
|  |  |  |  | A*30:04~B*14:01~C*08:02 | 0.0006 |  |  | A*68:02~B*53:01~C*04:01 | 0.0091 | A*23:01~B*41:02~C*17:03 | 0.0006 | A*30:02~B*45:01~C*16:01 | 0.0059 |
|  |  |  |  | A*30:04~B*14:01~C*08:04 | 0.0009 |  |  | A*68:02~B*57:02~C*18:01 | 0.0018 | A*23:01~B*42:01~C*17:01 | 0.0003 | A*30:02~B*50:01~C*06:02 | 0.0008 |
|  |  |  |  | A*30:04~B*15:03~C*02:10 | 0.0003 |  |  | A*68:02~B*57:03~C*18:01 | 0.0010 | A*23:01~B*44:03~C*04:01 | 0.0054 | A*30:02~B*53:01~C*04:01 | 0.0062 |
|  |  |  |  | A*30:04~B*15:16~C*14:02 | 0.0006 |  |  | A*68:02~B*58:01~C*03:02 | 0.0026 | A*23:01~B*45:01~C*06:02 | 0.0003 | A*30:02~B*53:01~C*06:02 | 0.0024 |
|  |  |  |  | A*30:04~B*18:01~C*07:04 | 0.0003 |  |  | A*68:02~B*58:01~C*06:02 | 0.0018 | A*23:01~B*49:01~C*07:01 | 0.0028 | A*30:02~B*57:02~C*18:00 | 0.0010 |
|  |  |  |  | A*30:04~B*27:05~C*02:10 | 0.0006 |  |  | A*68:02~B*58:01~C*07:01 | 0.0027 | A*23:01~B*50:01~C*06:02 | 0.0011 | A*30:02~B*57:02~C*18:02 | 0.0008 |
|  |  |  |  | A*30:04~B*39:10~C*15:05 | 0.0003 |  |  | A*68:02~B*58:02~C*06:02 | 0.0009 | A*23:01~B*50:02~C*04:01 | 0.0003 | A*30:02~B*57:03~C*07:01 | 0.0064 |
|  |  |  |  | A*30:04~B*41:01~C*17:00 | 0.0006 |  |  | A*68:02~B*81:01~C*07:01 | 0.0009 | A*23:01~B*51:01~C*14:02 | 0.0003 | A*30:02~B*57:03~C*08:02 | 0.0015 |
|  |  |  |  | A*30:04~B*41:01~C*17:01 | 0.0012 |  |  | A*68:02~B*81:01~C*18:01 | 0.0009 | A*23:01~B*52:01~C*02:02 | 0.0003 | A*30:02~B*57:03~C*18:00 | 0.0088 |
|  |  |  |  | A*30:04~B*41:02~C*16:01 | 0.0003 |  |  | A*74:01~B*07:02~C*15:02 | 0.0009 | A*23:01~B*53:01~C*04:01 | 0.0008 | A*30:02~B*57:03~C*18:02 | 0.0090 |
|  |  |  |  | A*30:04~B*41:02~C*17:01 | 0.0003 |  |  | A*74:01~B*08:01~C*07:01 | 0.0009 | A*23:01~B*57:03~C*07:01 | 0.0006 | A*30:02~B*58:01~C*07:01 | 0.0015 |
|  |  |  |  | A*30:04~B*42:01~C*17:01 | 0.0003 |  |  | A*74:01~B*14:01~C*08:02 | 0.0011 | A*23:01~B*58:01~C*07:01 | 0.0003 | A*30:02~B*58:02~C*06:02 | 0.0024 |
|  |  |  |  | A*30:04~B*44:03~C*02:10 | 0.0010 |  |  | A*74:01~B*14:02~C*08:02 | 0.0011 | A*24:02~B*07:02~C*07:02 | 0.0075 | A*30:02~B*78:01~C*16:01 | 0.0008 |
|  |  |  |  | A*30:04~B*44:03~C*04:01 | 0.0015 |  |  | A*74:01~B*15:03~C*02:02 | 0.0009 | A*24:02~B*07:02~C*15:02 | 0.0003 | A*30:02~B*81:01~C*08:04 | 0.0045 |
|  |  |  |  | A*30:04~B*45:01~C*16:01 | 0.0007 |  |  | A*74:01~B*15:03~C*02:10 | 0.0277 | A*24:02~B*07:05~C*02:02 | 0.0003 | A*30:03~B*51:01~C*06:02 | 0.0008 |
|  |  |  |  | A*30:04~B*51:01~C*07:01 | 0.0006 |  |  | A*74:01~B*15:10~C*17:01 | 0.0018 | A*24:02~B*08:01~C*03:04 | 0.0002 | A*30:04~B*57:01~C*07:01 | 0.0008 |
|  |  |  |  | A*30:04~B*53:01~C*04:01 | 0.0003 |  |  | A*74:01~B*15:83~C*15:25 | 0.0009 | A*24:02~B*08:01~C*07:01 | 0.0016 | A*30:04~B*58:02~C*06:02 | 0.0008 |
|  |  |  |  | A*30:04~B*58:02~C*06:02 | 0.0027 |  |  | A*74:01~B*18:01~C*02:10 | 0.0009 | A*24:02~B*08:01~C*07:02 | 0.0009 | A*30:04~B*73:01~C*15:05 | 0.0008 |
|  |  |  |  | A*30:04~B*82:02~C*03:02 | 0.0027 |  |  | A*74:01~B*18:01~C*06:02 | 0.0009 | A*24:02~B*13:02~C*06:02 | 0.0028 | A*30:09~B*45:01~C*06:02 | 0.0008 |
|  |  |  |  | A*30:09~B*42:01~C*17:00 | 0.0003 |  |  | A*74:01~B*35:01~C*04:01 | 0.0149 | A*24:02~B*14:02~C*02:02 | 0.0008 | A*30:10~B*13:02~C*06:02 | 0.0015 |
|  |  |  |  | A*30:09~B*81:01~C*04:01 | 0.0043 |  |  | A*74:01~B*42:01~C*17:01 | 0.0038 | A*24:02~B*14:02~C*08:02 | 0.0004 | A*30:151~B*57:03~C*17:01 | 0.0008 |
|  |  |  |  | A*31:01~B*07:05~C*07:02 | 0.0003 |  |  | A*74:01~B*44:03~C*04:01 | 0.0001 | A*24:02~B*15:01~C*03:03 | 0.0056 | A*31:01~B*15:01~C*01:02 | 0.0008 |
|  |  |  |  | A*31:01~B*15:03~C*18:00 | 0.0009 |  |  | A*74:01~B*44:03~C*07:01 | 0.0018 | A*24:02~B*15:03~C*02:10 | 0.0003 | A*31:01~B*35:01~C*04:01 | 0.0008 |
|  |  |  |  | A*32:01~B*07:02~C*01:02 | 0.0003 |  |  | A*74:01~B*50:01~C*06:02 | 0.0026 | A*24:02~B*15:03~C*15:05 | 0.0003 | A*31:01~B*40:01~C*03:04 | 0.0053 |
|  |  |  |  | A*32:01~B*07:02~C*02:10 | 0.0006 |  |  | A*74:01~B*53:01~C*02:02 | 0.0009 | A*24:02~B*15:07~C*03:03 | 0.0006 | A*31:01~B*51:01~C*03:02 | 0.0008 |
|  |  |  |  | A*32:01~B*07:05~C*07:02 | 0.0006 |  |  | A*74:01~B*53:01~C*04:01 | 0.0027 | A*24:02~B*15:17~C*07:01 | 0.0023 | A*31:01~B*51:01~C*08:01 | 0.0008 |
|  |  |  |  | A*32:01~B*15:01~C*04:01 | 0.0009 |  |  | A*74:01~B*57:03~C*18:01 | 0.0009 | A*24:02~B*15:24~C*03:03 | 0.0003 | A*31:01~B*51:01~C*15:02 | 0.0008 |
|  |  |  |  | A*32:01~B*15:03~C*04:01 | 0.0003 |  |  | A*74:01~B*58:01~C*07:04 | 0.0009 | A*24:02~B*18:01~C*07:01 | 0.0016 | A*31:01~B*52:01~C*15:02 | 0.0008 |
|  |  |  |  | A*32:01~B*42:01~C*17:00 | 0.0003 |  |  | A*74:01~B*58:02~C*06:02 | 0.0035 | A*24:02~B*18:01~C*12:03 | 0.0025 | A*31:01~B*57:03~C*07:01 | 0.0091 |
|  |  |  |  | A*32:01~B*42:01~C*17:01 | 0.0003 |  |  | A*80:01~B*15:03~C*02:10 | 0.0009 | A*24:02~B*27:02~C*02:02 | 0.0016 | A*31:01~B*57:03~C*07:621 | 0.0008 |
|  |  |  |  | A*32:01~B*44:03~C*02:10 | 0.0009 |  |  | A*80:01~B*18:01~C*02:02 | 0.0009 | A*24:02~B*27:05~C*01:02 | 0.0006 | A*32:01~B*08:01~C*07:01 | 0.0015 |
|  |  |  |  | A*32:106~B*07:02~C*02:10 | 0.0006 |  |  | A*80:01~B*53:01~C*04:01 | 0.0018 | A*24:02~B*27:05~C*02:02 | 0.0030 | A*32:01~B*14:01~C*08:02 | 0.0015 |
|  |  |  |  | A*32:106~B*07:05~C*07:02 | 0.0006 |  |  | A*80:01~B*58:01~C*03:02 | 0.0009 | A*24:02~B*27:07~C*15:02 | 0.0008 | A*32:01~B*15:16~C*16:01 | 0.0008 |
|  |  |  |  | A*32:106~B*44:03~C*02:10 | 0.0003 |  |  |  |  | A*24:02~B*27:12~C*02:02 | 0.0003 | A*32:01~B*15:18~C*07:04 | 0.0008 |
|  |  |  |  | A*32:106~B*58:02~C*06:02 | 0.0003 |  |  |  |  | A*24:02~B*35:01~C*03:04 | 0.0003 | A*32:01~B*18:01~C*07:04 | 0.0008 |
|  |  |  |  | A*33:01~B*07:02~C*07:02 | 0.0001 |  |  |  |  | A*24:02~B*35:01~C*04:01 | 0.0029 | A*32:01~B*18:01~C*12:03 | 0.0008 |
|  |  |  |  | A*33:01~B*15:220~C*04:01 | 0.0003 |  |  |  |  | A*24:02~B*35:02~C*04:01 | 0.0030 | A*32:01~B*35:02~C*02:02 | 0.0008 |
|  |  |  |  | A*33:01~B*41:02~C*17:00 | 0.0003 |  |  |  |  | A*24:02~B*35:03~C*04:01 | 0.0032 | A*32:01~B*39:06~C*07:02 | 0.0008 |
|  |  |  |  | A*33:01~B*42:01~C*17:00 | 0.0024 |  |  |  |  | A*24:02~B*35:03~C*12:03 | 0.0013 | A*32:01~B*39:10~C*14:03 | 0.0008 |
|  |  |  |  | A*33:01~B*42:01~C*17:01 | 0.0029 |  |  |  |  | A*24:02~B*35:08~C*04:01 | 0.0008 | A*32:01~B*40:01~C*03:04 | 0.0008 |
|  |  |  |  | A*33:01~B*44:03~C*07:01 | 0.0003 |  |  |  |  | A*24:02~B*35:43~C*01:02 | 0.0006 | A*32:01~B*40:02~C*02:02 | 0.0015 |
|  |  |  |  | A*33:01~B*57:03~C*08:02 | 0.0003 |  |  |  |  | A*24:02~B*37:01~C*06:02 | 0.0013 | A*32:01~B*42:02~C*17:00 | 0.0008 |
|  |  |  |  | A*33:01~B*58:02~C*06:02 | 0.0003 |  |  |  |  | A*24:02~B*38:01~C*12:03 | 0.0033 | A*32:01~B*44:02~C*02:02 | 0.0008 |
|  |  |  |  | A*33:01~B*81:01~C*18:01 | 0.0003 |  |  |  |  | A*24:02~B*39:01~C*07:02 | 0.0003 | A*32:01~B*44:02~C*05:01 | 0.0008 |
|  |  |  |  | A*33:03~B*07:02~C*07:02 | 0.0049 |  |  |  |  | A*24:02~B*39:01~C*12:03 | 0.0012 | A*32:01~B*53:01~C*06:02 | 0.0005 |
|  |  |  |  | A*33:03~B*15:16~C*14:02 | 0.0003 |  |  |  |  | A*24:02~B*39:05~C*18:01 | 0.0003 | A*32:01~B*57:03~C*02:02 | 0.0008 |
|  |  |  |  | A*33:03~B*42:01~C*17:00 | 0.0006 |  |  |  |  | A*24:02~B*39:06~C*07:02 | 0.0017 | A*32:01~B*57:03~C*18:00 | 0.0010 |
|  |  |  |  | A*33:03~B*42:01~C*17:01 | 0.0028 |  |  |  |  | A*24:02~B*40:01~C*03:03 | 0.0003 | A*32:01~B*81:01~C*08:04 | 0.0008 |
|  |  |  |  | A*33:03~B*44:03~C*02:10 | 0.0003 |  |  |  |  | A*24:02~B*40:01~C*03:04 | 0.0024 | A*32:01~B*81:01~C*18:02 | 0.0008 |
|  |  |  |  | A*33:03~B*53:01~C*04:01 | 0.0054 |  |  |  |  | A*24:02~B*40:02~C*02:02 | 0.0022 | A*33:01~B*07:05~C*07:02 | 0.0008 |
|  |  |  |  | A*33:03~B*58:01~C*03:02 | 0.0003 |  |  |  |  | A*24:02~B*40:02~C*03:04 | 0.0005 | A*33:01~B*08:01~C*07:01 | 0.0008 |
|  |  |  |  | A*33:03~B*58:02~C*06:02 | 0.0003 |  |  |  |  | A*24:02~B*40:02~C*03:05 | 0.0003 | A*33:01~B*14:02~C*08:02 | 0.0050 |
|  |  |  |  | A*34:01~B*57:01~C*06:02 | 0.0003 |  |  |  |  | A*24:02~B*40:02~C*03:06 | 0.0003 | A*33:01~B*35:01~C*04:01 | 0.0015 |
|  |  |  |  | A*34:02~B*08:01~C*07:01 | 0.0052 |  |  |  |  | A*24:02~B*40:06~C*08:01 | 0.0003 | A*33:01~B*35:01~C*06:02 | 0.0008 |
|  |  |  |  | A*34:02~B*14:02~C*08:02 | 0.0003 |  |  |  |  | A*24:02~B*41:01~C*17:00 | 0.0003 | A*33:01~B*39:10~C*12:03 | 0.0008 |
|  |  |  |  | A*34:02~B*15:03~C*02:10 | 0.0048 |  |  |  |  | A*24:02~B*44:02~C*05:01 | 0.0022 | A*33:01~B*42:01~C*17:01 | 0.0008 |
|  |  |  |  | A*34:02~B*18:01~C*07:04 | 0.0003 |  |  |  |  | A*24:02~B*44:02~C*16:04 | 0.0003 | A*33:01~B*57:03~C*07:01 | 0.0015 |
|  |  |  |  | A*34:02~B*39:10~C*12:03 | 0.0003 |  |  |  |  | A*24:02~B*44:03~C*04:01 | 0.0007 | A*33:01~B*58:01~C*03:02 | 0.0008 |
|  |  |  |  | A*34:02~B*42:01~C*17:00 | 0.0006 |  |  |  |  | A*24:02~B*44:03~C*16:01 | 0.0011 | A*33:01~B*58:01~C*07:01 | 0.0015 |
|  |  |  |  | A*34:02~B*42:01~C*17:01 | 0.0004 |  |  |  |  | A*24:02~B*44:05~C*02:02 | 0.0003 | A*33:01~B*78:01~C*16:01 | 0.0023 |
|  |  |  |  | A*34:02~B*44:03~C*02:17 | 0.0001 |  |  |  |  | A*24:02~B*45:01~C*02:02 | 0.0003 | A*33:01~B*81:01~C*18:00 | 0.0011 |
|  |  |  |  | A*34:02~B*44:03~C*04:01 | 0.0213 |  |  |  |  | A*24:02~B*48:01~C*08:01 | 0.0003 | A*33:03~B*07:02~C*07:02 | 0.0026 |
|  |  |  |  | A*34:02~B*44:03~C*07:01 | 0.0015 |  |  |  |  | A*24:02~B*50:01~C*06:02 | 0.0004 | A*33:03~B*07:05~C*15:05 | 0.0015 |
|  |  |  |  | A*34:02~B*44:03~C*07:06 | 0.0003 |  |  |  |  | A*24:02~B*51:01~C*01:02 | 0.0004 | A*33:03~B*08:01~C*07:01 | 0.0008 |
|  |  |  |  | A*34:02~B*45:01~C*06:02 | 0.0003 |  |  |  |  | A*24:02~B*51:01~C*02:02 | 0.0004 | A*33:03~B*13:02~C*06:02 | 0.0023 |
|  |  |  |  | A*34:02~B*53:01~C*04:01 | 0.0006 |  |  |  |  | A*24:02~B*51:01~C*12:03 | 0.0003 | A*33:03~B*14:02~C*08:02 | 0.0010 |
|  |  |  |  | A*34:02~B*57:03~C*18:02 | 0.0003 |  |  |  |  | A*24:02~B*51:01~C*14:02 | 0.0010 | A*33:03~B*15:03~C*02:10 | 0.0009 |
|  |  |  |  | A*34:02~B*58:02~C*06:02 | 0.0036 |  |  |  |  | A*24:02~B*51:01~C*16:01 | 0.0005 | A*33:03~B*15:03~C*18:00 | 0.0008 |
|  |  |  |  | A*34:02~B*58:02~C*18:00 | 0.0003 |  |  |  |  | A*24:02~B*51:09~C*01:02 | 0.0008 | A*33:03~B*15:10~C*03:04 | 0.0033 |
|  |  |  |  | A*34:02~B*81:00~C*08:04 | 0.0006 |  |  |  |  | A*24:02~B*51:22~C*15:02 | 0.0003 | A*33:03~B*15:16~C*14:02 | 0.0061 |
|  |  |  |  | A*34:02~B*81:01~C*08:04 | 0.0027 |  |  |  |  | A*24:02~B*52:01~C*12:02 | 0.0006 | A*33:03~B*38:01~C*12:03 | 0.0008 |
|  |  |  |  | A*34:02~B*81:03~C*08:04 | 0.0003 |  |  |  |  | A*24:02~B*54:01~C*01:02 | 0.0003 | A*33:03~B*39:10~C*12:03 | 0.0023 |
|  |  |  |  | A*36:01~B*07:02~C*03:04 | 0.0002 |  |  |  |  | A*24:02~B*55:01~C*01:02 | 0.0008 | A*33:03~B*40:12~C*15:05 | 0.0008 |
|  |  |  |  | A*36:01~B*15:10~C*04:01 | 0.0009 |  |  |  |  | A*24:02~B*55:01~C*03:03 | 0.0012 | A*33:03~B*42:01~C*03:02 | 0.0008 |
|  |  |  |  | A*36:01~B*18:01~C*07:04 | 0.0003 |  |  |  |  | A*24:02~B*57:01~C*06:02 | 0.0050 | A*33:03~B*42:01~C*17:00 | 0.0008 |
|  |  |  |  | A*36:01~B*42:01~C*17:00 | 0.0004 |  |  |  |  | A*24:02~B*57:01~C*07:01 | 0.0003 | A*33:03~B*42:01~C*17:01 | 0.0023 |
|  |  |  |  | A*36:01~B*53:01~C*04:01 | 0.0024 |  |  |  |  | A*24:02~B*57:01~C*07:02 | 0.0003 | A*33:03~B*44:03~C*07:01 | 0.0008 |
|  |  |  |  | A*43:01~B*07:02~C*02:10 | 0.0003 |  |  |  |  | A*24:02~B*57:03~C*07:01 | 0.0006 | A*33:03~B*49:01~C*07:01 | 0.0020 |
|  |  |  |  | A*43:01~B*07:05~C*07:02 | 0.0006 |  |  |  |  | A*24:02~B*58:01~C*03:02 | 0.0002 | A*33:03~B*49:01~C*18:00 | 0.0008 |
|  |  |  |  | A*43:01~B*08:01~C*07:01 | 0.0003 |  |  |  |  | A*24:02~B*58:01~C*06:02 | 0.0003 | A*33:03~B*51:01~C*04:01 | 0.0008 |
|  |  |  |  | A*43:01~B*13:02~C*16:01 | 0.0003 |  |  |  |  | A*24:02~B*73:01~C*15:05 | 0.0003 | A*33:03~B*51:01~C*16:01 | 0.0008 |
|  |  |  |  | A*43:01~B*14:01~C*08:04 | 0.0012 |  |  |  |  | A*24:03~B*14:01~C*08:02 | 0.0003 | A*33:03~B*53:01~C*04:01 | 0.0151 |
|  |  |  |  | A*43:01~B*15:01~C*04:01 | 0.0003 |  |  |  |  | A*24:03~B*15:17~C*07:01 | 0.0006 | A*33:03~B*53:01~C*04:13 | 0.0015 |
|  |  |  |  | A*43:01~B*15:03~C*18:00 | 0.0055 |  |  |  |  | A*24:03~B*18:01~C*02:02 | 0.0003 | A*33:03~B*53:01~C*07:01 | 0.0009 |
|  |  |  |  | A*43:01~B*15:03~C*18:01 | 0.0003 |  |  |  |  | A*24:03~B*18:01~C*12:03 | 0.0003 | A*33:03~B*56:01~C*08:04 | 0.0008 |
|  |  |  |  | A*43:01~B*15:03~C*18:02 | 0.0018 |  |  |  |  | A*24:03~B*27:05~C*02:02 | 0.0003 | A*33:03~B*57:02~C*18:00 | 0.0009 |
|  |  |  |  | A*43:01~B*15:10~C*03:04 | 0.0003 |  |  |  |  | A*24:03~B*35:43~C*01:02 | 0.0006 | A*33:03~B*58:01~C*03:02 | 0.0045 |
|  |  |  |  | A*43:01~B*15:10~C*04:01 | 0.0012 |  |  |  |  | A*24:03~B*49:01~C*07:01 | 0.0003 | A*33:03~B*58:01~C*06:02 | 0.0015 |
|  |  |  |  | A*43:01~B*35:02~C*04:01 | 0.0006 |  |  |  |  | A*24:03~B*50:01~C*06:02 | 0.0003 | A*33:03~B*58:01~C*07:01 | 0.0020 |
|  |  |  |  | A*43:01~B*39:10~C*12:03 | 0.0007 |  |  |  |  | A*24:03~B*51:01~C*04:01 | 0.0003 | A*33:05~B*44:03~C*07:02 | 0.0008 |
|  |  |  |  | A*43:01~B*42:01~C*17:01 | 0.0003 |  |  |  |  | A*24:03~B*56:01~C*01:02 | 0.0003 | A*34:02~B*07:02~C*15:05 | 0.0008 |
|  |  |  |  | A*43:01~B*44:03~C*02:10 | 0.0016 |  |  |  |  | A*24:03~B*57:03~C*07:01 | 0.0003 | A*34:02~B*07:05~C*07:02 | 0.0008 |
|  |  |  |  | A*43:01~B*44:03~C*04:01 | 0.0005 |  |  |  |  | A*24:05~B*40:08~C*03:04 | 0.0003 | A*34:02~B*13:02~C*18:00 | 0.0008 |
|  |  |  |  | A*43:01~B*44:03~C*07:01 | 0.0003 |  |  |  |  | A*24:23~B*51:01~C*14:02 | 0.0003 | A*34:02~B*14:02~C*08:02 | 0.0008 |
|  |  |  |  | A*43:01~B*57:03~C*07:01 | 0.0003 |  |  |  |  | A*24:314~B*18:01~C*12:03 | 0.0003 | A*34:02~B*15:03~C*02:10 | 0.0030 |
|  |  |  |  | A*43:01~B*57:03~C*18:00 | 0.0003 |  |  |  |  | A*24:95~B*39:01~C*12:03 | 0.0003 | A*34:02~B*15:17~C*05:01 | 0.0008 |
|  |  |  |  | A*43:01~B*58:02~C*06:02 | 0.0103 |  |  |  |  | A*25:01~B*07:02~C*07:02 | 0.0015 | A*34:02~B*15:18~C*07:01 | 0.0008 |
|  |  |  |  | A*66:01~B*08:01~C*02:17 | 0.0003 |  |  |  |  | A*25:01~B*08:01~C*04:01 | 0.0003 | A*34:02~B*18:01~C*05:01 | 0.0008 |
|  |  |  |  | A*66:01~B*08:01~C*03:04 | 0.0003 |  |  |  |  | A*25:01~B*08:01~C*07:01 | 0.0025 | A*34:02~B*18:01~C*08:02 | 0.0008 |
|  |  |  |  | A*66:01~B*08:01~C*06:02 | 0.0003 |  |  |  |  | A*25:01~B*14:02~C*08:02 | 0.0017 | A*34:02~B*27:12~C*02:02 | 0.0008 |
|  |  |  |  | A*66:01~B*08:01~C*07:01 | 0.0008 |  |  |  |  | A*25:01~B*15:01~C*03:03 | 0.0008 | A*34:02~B*39:10~C*12:03 | 0.0015 |
|  |  |  |  | A*66:01~B*13:02~C*06:02 | 0.0004 |  |  |  |  | A*25:01~B*15:01~C*03:04 | 0.0006 | A*34:02~B*41:03~C*17:03 | 0.0008 |
|  |  |  |  | A*66:01~B*15:03~C*02:10 | 0.0007 |  |  |  |  | A*25:01~B*18:01~C*05:01 | 0.0003 | A*34:02~B*44:03~C*04:01 | 0.0085 |
|  |  |  |  | A*66:01~B*15:03~C*17:00 | 0.0003 |  |  |  |  | A*25:01~B*18:01~C*07:01 | 0.0001 | A*34:02~B*44:03~C*07:01 | 0.0023 |
|  |  |  |  | A*66:01~B*15:03~C*18:00 | 0.0003 |  |  |  |  | A*25:01~B*18:01~C*12:03 | 0.0110 | A*34:02~B*44:15~C*04:07 | 0.0008 |
|  |  |  |  | A*66:01~B*15:10~C*03:04 | 0.0004 |  |  |  |  | A*25:01~B*27:05~C*01:02 | 0.0001 | A*34:02~B*49:01~C*07:01 | 0.0015 |
|  |  |  |  | A*66:01~B*15:18~C*18:00 | 0.0003 |  |  |  |  | A*25:01~B*27:05~C*03:03 | 0.0003 | A*34:02~B*51:01~C*16:01 | 0.0015 |
|  |  |  |  | A*66:01~B*35:01~C*04:01 | 0.0007 |  |  |  |  | A*25:01~B*27:05~C*15:02 | 0.0003 | A*34:02~B*53:01~C*03:03 | 0.0008 |
|  |  |  |  | A*66:01~B*39:10~C*12:03 | 0.0045 |  |  |  |  | A*25:01~B*35:01~C*04:01 | 0.0008 | A*34:02~B*53:01~C*04:01 | 0.0034 |
|  |  |  |  | A*66:01~B*42:01~C*17:00 | 0.0004 |  |  |  |  | A*25:01~B*35:41~C*04:01 | 0.0003 | A*34:02~B*58:01~C*07:01 | 0.0008 |
|  |  |  |  | A*66:01~B*44:03~C*04:01 | 0.0012 |  |  |  |  | A*25:01~B*37:01~C*06:02 | 0.0003 | A*34:02~B*58:02~C*04:01 | 0.0008 |
|  |  |  |  | A*66:01~B*57:02~C*18:00 | 0.0003 |  |  |  |  | A*25:01~B*39:01~C*12:03 | 0.0021 | A*34:02~B*78:01~C*02:10 | 0.0008 |
|  |  |  |  | A*66:01~B*58:01~C*03:02 | 0.0006 |  |  |  |  | A*25:01~B*39:11~C*07:02 | 0.0003 | A*34:02~B*81:01~C*08:04 | 0.0032 |
|  |  |  |  | A*66:01~B*58:02~C*04:01 | 0.0003 |  |  |  |  | A*25:01~B*40:02~C*02:02 | 0.0005 | A*36:01~B*15:10~C*03:04 | 0.0018 |
|  |  |  |  | A*66:01~B*58:02~C*06:02 | 0.0269 |  |  |  |  | A*25:01~B*44:02~C*05:01 | 0.0016 | A*36:01~B*15:16~C*14:02 | 0.0012 |
|  |  |  |  | A*66:01~B*81:01~C*18:00 | 0.0006 |  |  |  |  | A*25:01~B*44:03~C*07:06 | 0.0003 | A*36:01~B*18:01~C*04:01 | 0.0008 |
|  |  |  |  | A*66:01~B*82:02~C*03:02 | 0.0003 |  |  |  |  | A*25:01~B*44:03~C*16:01 | 0.0003 | A*36:01~B*39:10~C*12:03 | 0.0008 |
|  |  |  |  | A*66:02~B*15:03~C*02:10 | 0.0006 |  |  |  |  | A*25:01~B*51:01~C*03:04 | 0.0003 | A*36:01~B*40:06~C*15:02 | 0.0008 |
|  |  |  |  | A*66:02~B*42:01~C*17:00 | 0.0032 |  |  |  |  | A*25:01~B*55:01~C*03:03 | 0.0002 | A*36:01~B*42:01~C*17:00 | 0.0015 |
|  |  |  |  | A*66:02~B*42:01~C*17:01 | 0.0055 |  |  |  |  | A*25:01~B*57:01~C*06:02 | 0.0008 | A*36:01~B*44:03~C*04:01 | 0.0015 |
|  |  |  |  | A*66:02~B*44:03~C*04:01 | 0.0007 |  |  |  |  | A*25:01~B*58:01~C*03:02 | 0.0003 | A*36:01~B*51:01~C*15:02 | 0.0008 |
|  |  |  |  | A*66:02~B*44:03~C*17:01 | 0.0003 |  |  |  |  | A*25:01~B*58:01~C*07:01 | 0.0017 | A*36:01~B*51:01~C*18:02 | 0.0008 |
|  |  |  |  | A*66:03~B*35:01~C*04:01 | 0.0003 |  |  |  |  | A*26:01~B*07:02~C*07:02 | 0.0014 | A*36:01~B*52:01~C*16:01 | 0.0008 |
|  |  |  |  | A*66:03~B*44:03~C*04:01 | 0.0003 |  |  |  |  | A*26:01~B*07:02~C*16:01 | 0.0003 | A*36:01~B*53:01~C*04:01 | 0.0080 |
|  |  |  |  | A*66:03~B*57:03~C*07:01 | 0.0003 |  |  |  |  | A*26:01~B*07:05~C*15:05 | 0.0003 | A*36:01~B*57:03~C*16:01 | 0.0008 |
|  |  |  |  | A*68:01~B*07:02~C*02:10 | 0.0003 |  |  |  |  | A*26:01~B*08:01~C*07:01 | 0.0003 | A*36:01~B*58:02~C*06:02 | 0.0018 |
|  |  |  |  | A*68:01~B*07:05~C*07:01 | 0.0003 |  |  |  |  | A*26:01~B*13:02~C*06:02 | 0.0012 | A*66:01~B*08:01~C*07:02 | 0.0008 |
|  |  |  |  | A*68:01~B*08:01~C*02:10 | 0.0003 |  |  |  |  | A*26:01~B*14:01~C*08:02 | 0.0008 | A*66:01~B*13:02~C*02:10 | 0.0008 |
|  |  |  |  | A*68:01~B*08:01~C*06:02 | 0.0003 |  |  |  |  | A*26:01~B*15:17~C*07:01 | 0.0003 | A*66:01~B*15:03~C*04:01 | 0.0008 |
|  |  |  |  | A*68:01~B*15:03~C*18:00 | 0.0003 |  |  |  |  | A*26:01~B*18:01~C*01:02 | 0.0003 | A*66:01~B*15:10~C*03:04 | 0.0008 |
|  |  |  |  | A*68:01~B*15:10~C*16:01 | 0.0003 |  |  |  |  | A*26:01~B*18:01~C*05:01 | 0.0005 | A*66:01~B*35:01~C*04:01 | 0.0022 |
|  |  |  |  | A*68:01~B*27:05~C*02:02 | 0.0003 |  |  |  |  | A*26:01~B*18:01~C*12:03 | 0.0003 | A*66:01~B*39:10~C*12:03 | 0.0024 |
|  |  |  |  | A*68:01~B*35:01~C*04:01 | 0.0018 |  |  |  |  | A*26:01~B*27:02~C*02:02 | 0.0003 | A*66:01~B*41:02~C*17:03 | 0.0008 |
|  |  |  |  | A*68:01~B*41:01~C*17:00 | 0.0006 |  |  |  |  | A*26:01~B*27:02~C*05:01 | 0.0003 | A*66:01~B*42:01~C*06:02 | 0.0008 |
|  |  |  |  | A*68:01~B*41:01~C*17:01 | 0.0026 |  |  |  |  | A*26:01~B*27:05~C*01:02 | 0.0016 | A*66:01~B*42:01~C*17:01 | 0.0015 |
|  |  |  |  | A*68:01~B*42:01~C*17:00 | 0.0006 |  |  |  |  | A*26:01~B*27:05~C*02:02 | 0.0018 | A*66:01~B*44:03~C*04:01 | 0.0008 |
|  |  |  |  | A*68:01~B*44:03~C*04:01 | 0.0003 |  |  |  |  | A*26:01~B*35:01~C*04:01 | 0.0022 | A*66:01~B*44:03~C*14:03 | 0.0008 |
|  |  |  |  | A*68:01~B*51:01~C*16:04 | 0.0003 |  |  |  |  | A*26:01~B*35:02~C*04:01 | 0.0003 | A*66:01~B*45:01~C*04:07 | 0.0008 |
|  |  |  |  | A*68:01~B*58:01~C*06:02 | 0.0030 |  |  |  |  | A*26:01~B*35:03~C*04:01 | 0.0003 | A*66:01~B*45:01~C*18:00 | 0.0008 |
|  |  |  |  | A*68:01~B*58:02~C*06:02 | 0.0154 |  |  |  |  | A*26:01~B*37:01~C*06:02 | 0.0005 | A*66:01~B*49:01~C*08:02 | 0.0008 |
|  |  |  |  | A*68:01~B*82:02~C*03:02 | 0.0015 |  |  |  |  | A*26:01~B*38:01~C*12:03 | 0.0069 | A*66:01~B*51:07~C*14:02 | 0.0008 |
|  |  |  |  | A*68:02~B*07:02~C*03:04 | 0.0004 |  |  |  |  | A*26:01~B*39:01~C*12:03 | 0.0002 | A*66:01~B*52:01~C*16:01 | 0.0008 |
|  |  |  |  | A*68:02~B*07:02~C*07:01 | 0.0003 |  |  |  |  | A*26:01~B*39:24~C*07:01 | 0.0003 | A*66:01~B*53:01~C*04:01 | 0.0008 |
|  |  |  |  | A*68:02~B*07:02~C*07:02 | 0.0139 |  |  |  |  | A*26:01~B*44:02~C*01:02 | 0.0003 | A*66:01~B*53:01~C*06:02 | 0.0015 |
|  |  |  |  | A*68:02~B*07:02~C*15:05 | 0.0003 |  |  |  |  | A*26:01~B*44:02~C*05:01 | 0.0010 | A*66:01~B*58:02~C*06:02 | 0.0030 |
|  |  |  |  | A*68:02~B*08:01~C*07:01 | 0.0003 |  |  |  |  | A*26:01~B*44:02~C*15:02 | 0.0003 | A*66:02~B*39:01~C*07:01 | 0.0008 |
|  |  |  |  | A*68:02~B*08:01~C*08:04 | 0.0003 |  |  |  |  | A*26:01~B*44:03~C*04:01 | 0.0005 | A*66:02~B*53:01~C*04:01 | 0.0008 |
|  |  |  |  | A*68:02~B*14:01~C*08:02 | 0.0040 |  |  |  |  | A*26:01~B*45:01~C*06:02 | 0.0011 | A*66:02~B*58:01~C*07:01 | 0.0068 |
|  |  |  |  | A*68:02~B*14:01~C*08:04 | 0.0009 |  |  |  |  | A*26:01~B*48:01~C*08:01 | 0.0003 | A*66:03~B*41:02~C*17:01 | 0.0008 |
|  |  |  |  | A*68:02~B*14:02~C*08:02 | 0.0034 |  |  |  |  | A*26:01~B*49:01~C*07:01 | 0.0025 | A*66:03~B*44:03~C*07:06 | 0.0008 |
|  |  |  |  | A*68:02~B*14:06~C*08:02 | 0.0003 |  |  |  |  | A*26:01~B*51:01~C*02:02 | 0.0002 | A*68:01~B*07:02~C*07:02 | 0.0038 |
|  |  |  |  | A*68:02~B*15:03~C*02:10 | 0.0022 |  |  |  |  | A*26:01~B*51:01~C*14:02 | 0.0003 | A*68:01~B*07:02~C*08:02 | 0.0008 |
|  |  |  |  | A*68:02~B*15:03~C*04:04 | 0.0003 |  |  |  |  | A*26:01~B*51:01~C*15:02 | 0.0013 | A*68:01~B*07:02~C*15:05 | 0.0015 |
|  |  |  |  | A*68:02~B*15:03~C*12:03 | 0.0003 |  |  |  |  | A*26:01~B*52:01~C*12:02 | 0.0003 | A*68:01~B*15:01~C*03:04 | 0.0008 |
|  |  |  |  | A*68:02~B*15:10~C*03:04 | 0.0215 |  |  |  |  | A*26:01~B*55:01~C*03:03 | 0.0007 | A*68:01~B*15:03~C*02:10 | 0.0009 |
|  |  |  |  | A*68:02~B*15:10~C*08:04 | 0.0117 |  |  |  |  | A*26:01~B*58:01~C*07:01 | 0.0003 | A*68:01~B*15:03~C*08:01 | 0.0008 |
|  |  |  |  | A*68:02~B*15:16~C*03:04 | 0.0030 |  |  |  |  | A*26:01~B*58:01~C*14:02 | 0.0003 | A*68:01~B*15:17~C*07:01 | 0.0008 |
|  |  |  |  | A*68:02~B*15:16~C*14:02 | 0.0003 |  |  |  |  | A*26:08~B*27:02~C*02:02 | 0.0003 | A*68:01~B*35:01~C*04:01 | 0.0016 |
|  |  |  |  | A*68:02~B*15:24~C*03:04 | 0.0003 |  |  |  |  | A*26:08~B*27:05~C*04:01 | 0.0003 | A*68:01~B*35:01~C*16:01 | 0.0014 |
|  |  |  |  | A*68:02~B*18:01~C*02:02 | 0.0004 |  |  |  |  | A*26:08~B*39:01~C*12:03 | 0.0008 | A*68:01~B*35:03~C*07:04 | 0.0008 |
|  |  |  |  | A*68:02~B*18:01~C*05:01 | 0.0009 |  |  |  |  | A*26:08~B*45:01~C*06:02 | 0.0003 | A*68:01~B*39:10~C*12:03 | 0.0008 |
|  |  |  |  | A*68:02~B*18:01~C*07:04 | 0.0006 |  |  |  |  | A*29:01~B*07:02~C*07:02 | 0.0004 | A*68:01~B*40:02~C*15:02 | 0.0008 |
|  |  |  |  | A*68:02~B*18:01~C*15:05 | 0.0020 |  |  |  |  | A*29:01~B*07:05~C*15:05 | 0.0014 | A*68:01~B*42:01~C*17:01 | 0.0008 |
|  |  |  |  | A*68:02~B*27:05~C*02:02 | 0.0006 |  |  |  |  | A*29:01~B*14:02~C*08:02 | 0.0003 | A*68:01~B*44:03~C*04:01 | 0.0015 |
|  |  |  |  | A*68:02~B*35:01~C*04:01 | 0.0004 |  |  |  |  | A*29:01~B*35:02~C*04:01 | 0.0003 | A*68:01~B*51:01~C*15:02 | 0.0008 |
|  |  |  |  | A*68:02~B*35:02~C*04:01 | 0.0002 |  |  |  |  | A*29:01~B*44:02~C*05:01 | 0.0002 | A*68:01~B*51:02~C*16:01 | 0.0008 |
|  |  |  |  | A*68:02~B*41:01~C*07:01 | 0.0003 |  |  |  |  | A*29:01~B*51:01~C*15:02 | 0.0003 | A*68:01~B*57:02~C*08:02 | 0.0008 |
|  |  |  |  | A*68:02~B*42:01~C*17:00 | 0.0018 |  |  |  |  | A*29:01~B*52:01~C*12:02 | 0.0006 | A*68:01~B*57:03~C*18:00 | 0.0015 |
|  |  |  |  | A*68:02~B*42:01~C*17:01 | 0.0018 |  |  |  |  | A*29:01~B*57:01~C*06:02 | 0.0003 | A*68:01~B*58:02~C*06:02 | 0.0083 |
|  |  |  |  | A*68:02~B*44:03~C*04:01 | 0.0009 |  |  |  |  | A*29:02~B*07:02~C*07:02 | 0.0016 | A*68:02~B*07:02~C*07:02 | 0.0029 |
|  |  |  |  | A*68:02~B*44:03~C*07:01 | 0.0010 |  |  |  |  | A*29:02~B*07:08~C*07:02 | 0.0003 | A*68:02~B*07:05~C*07:02 | 0.0015 |
|  |  |  |  | A*68:02~B*44:03~C*07:06 | 0.0012 |  |  |  |  | A*29:02~B*08:01~C*07:01 | 0.0003 | A*68:02~B*08:01~C*07:01 | 0.0009 |
|  |  |  |  | A*68:02~B*51:01~C*07:01 | 0.0003 |  |  |  |  | A*29:02~B*14:01~C*08:02 | 0.0003 | A*68:02~B*14:01~C*08:02 | 0.0015 |
|  |  |  |  | A*68:02~B*53:01~C*04:01 | 0.0021 |  |  |  |  | A*29:02~B*14:02~C*08:02 | 0.0007 | A*68:02~B*15:10~C*03:04 | 0.0063 |
|  |  |  |  | A*68:02~B*53:01~C*04:226 | 0.0003 |  |  |  |  | A*29:02~B*27:02~C*02:02 | 0.0002 | A*68:02~B*15:16~C*14:02 | 0.0026 |
|  |  |  |  | A*68:02~B*57:02~C*18:00 | 0.0021 |  |  |  |  | A*29:02~B*27:02~C*05:01 | 0.0003 | A*68:02~B*15:220~C*07:01 | 0.0008 |
|  |  |  |  | A*68:02~B*57:02~C*18:02 | 0.0015 |  |  |  |  | A*29:02~B*27:05~C*01:02 | 0.0003 | A*68:02~B*18:01~C*05:01 | 0.0008 |
|  |  |  |  | A*68:02~B*57:03~C*07:01 | 0.0009 |  |  |  |  | A*29:02~B*27:05~C*02:02 | 0.0003 | A*68:02~B*35:01~C*07:05 | 0.0008 |
|  |  |  |  | A*68:02~B*58:01~C*03:02 | 0.0016 |  |  |  |  | A*29:02~B*35:01~C*04:01 | 0.0007 | A*68:02~B*39:01~C*02:10 | 0.0008 |
|  |  |  |  | A*68:02~B*58:01~C*03:04 | 0.0014 |  |  |  |  | A*29:02~B*35:12~C*04:01 | 0.0003 | A*68:02~B*42:01~C*17:00 | 0.0008 |
|  |  |  |  | A*68:02~B*58:01~C*07:01 | 0.0007 |  |  |  |  | A*29:02~B*39:06~C*16:01 | 0.0003 | A*68:02~B*44:03~C*04:01 | 0.0011 |
|  |  |  |  | A*68:02~B*81:01~C*04:01 | 0.0006 |  |  |  |  | A*29:02~B*39:11~C*12:03 | 0.0003 | A*68:02~B*45:01~C*06:02 | 0.0008 |
|  |  |  |  | A*68:04~B*58:02~C*06:02 | 0.0003 |  |  |  |  | A*29:02~B*41:02~C*17:03 | 0.0003 | A*68:02~B*45:01~C*16:01 | 0.0019 |
|  |  |  |  | A*68:27~B*41:02~C*17:01 | 0.0003 |  |  |  |  | A*29:02~B*42:01~C*17:00 | 0.0003 | A*68:02~B*47:01~C*07:01 | 0.0008 |
|  |  |  |  | A*68:27~B*44:03~C*04:01 | 0.0015 |  |  |  |  | A*29:02~B*44:02~C*05:01 | 0.0006 | A*68:02~B*49:01~C*07:01 | 0.0023 |
|  |  |  |  | A*68:27~B*44:03~C*08:04 | 0.0003 |  |  |  |  | A*29:02~B*44:03~C*04:01 | 0.0005 | A*68:02~B*51:01~C*03:04 | 0.0008 |
|  |  |  |  | A*68:27~B*57:03~C*07:01 | 0.0003 |  |  |  |  | A*29:02~B*44:03~C*16:01 | 0.0226 | A*68:02~B*51:01~C*16:01 | 0.0008 |
|  |  |  |  | A*69:02~B*45:07~C*16:01 | 0.0003 |  |  |  |  | A*29:02~B*44:04~C*16:01 | 0.0006 | A*68:02~B*53:01~C*04:01 | 0.0139 |
|  |  |  |  | A*69:02~B*58:02~C*06:02 | 0.0000 |  |  |  |  | A*29:02~B*45:01~C*06:02 | 0.0014 | A*68:02~B*57:03~C*07:01 | 0.0049 |
|  |  |  |  | A*74:00~B*15:03~C*02:10 | 0.0079 |  |  |  |  | A*29:02~B*45:01~C*16:01 | 0.0003 | A*68:02~B*57:03~C*08:02 | 0.0018 |
|  |  |  |  | A*74:00~B*15:10~C*08:04 | 0.0003 |  |  |  |  | A*29:02~B*48:01~C*08:01 | 0.0003 | A*68:02~B*58:01~C*03:02 | 0.0008 |
|  |  |  |  | A*74:00~B*18:01~C*02:10 | 0.0003 |  |  |  |  | A*29:02~B*50:01~C*05:01 | 0.0003 | A*68:02~B*58:01~C*07:01 | 0.0008 |
|  |  |  |  | A*74:00~B*35:01~C*04:01 | 0.0057 |  |  |  |  | A*29:02~B*50:01~C*06:02 | 0.0002 | A*68:02~B*58:02~C*06:02 | 0.0015 |
|  |  |  |  | A*74:00~B*42:01~C*17:00 | 0.0008 |  |  |  |  | A*29:02~B*51:01~C*01:02 | 0.0005 | A*68:02~B*81:01~C*08:04 | 0.0030 |
|  |  |  |  | A*74:00~B*44:03~C*04:01 | 0.0001 |  |  |  |  | A*29:02~B*51:01~C*02:10 | 0.0003 | A*68:02~B*81:01~C*18:00 | 0.0037 |
|  |  |  |  | A*74:00~B*44:03~C*07:01 | 0.0003 |  |  |  |  | A*29:02~B*51:01~C*14:02 | 0.0003 | A*68:02~B*81:01~C*18:01 | 0.0015 |
|  |  |  |  | A*74:00~B*49:01~C*07:01 | 0.0009 |  |  |  |  | A*29:02~B*51:01~C*15:02 | 0.0003 | A*68:10~B*44:02~C*05:01 | 0.0008 |
|  |  |  |  | A*74:00~B*51:01~C*07:01 | 0.0003 |  |  |  |  | A*29:02~B*55:01~C*12:03 | 0.0003 | A*69:01~B*15:01~C*07:04 | 0.0008 |
|  |  |  |  | A*74:00~B*57:03~C*07:01 | 0.0052 |  |  |  |  | A*29:02~B*57:02~C*18:00 | 0.0003 | A*74:00~B*07:05~C*07:02 | 0.0008 |
|  |  |  |  | A*74:00~B*58:01~C*03:02 | 0.0003 |  |  |  |  | A*29:02~B*57:03~C*18:00 | 0.0003 | A*74:00~B*13:02~C*08:04 | 0.0008 |
|  |  |  |  | A*74:00~B*58:01~C*18:00 | 0.0003 |  |  |  |  | A*29:02~B*58:01~C*07:01 | 0.0011 | A*74:00~B*15:03~C*02:10 | 0.0013 |
|  |  |  |  | A*74:01~B*07:02~C*02:10 | 0.0004 |  |  |  |  | A*29:10~B*14:02~C*08:02 | 0.0006 | A*74:00~B*18:01~C*04:01 | 0.0008 |
|  |  |  |  | A*74:01~B*07:02~C*07:02 | 0.0003 |  |  |  |  | A*30:01~B*08:01~C*06:02 | 0.0003 | A*74:00~B*27:05~C*02:02 | 0.0008 |
|  |  |  |  | A*74:01~B*08:01~C*07:01 | 0.0004 |  |  |  |  | A*30:01~B*13:02~C*06:02 | 0.0116 | A*74:00~B*45:01~C*06:02 | 0.0006 |
|  |  |  |  | A*74:01~B*14:01~C*02:10 | 0.0002 |  |  |  |  | A*30:01~B*13:02~C*12:03 | 0.0006 | A*74:00~B*49:01~C*07:01 | 0.0023 |
|  |  |  |  | A*74:01~B*15:03~C*02:10 | 0.0194 |  |  |  |  | A*30:01~B*15:01~C*03:03 | 0.0008 | A*74:00~B*53:01~C*04:01 | 0.0010 |
|  |  |  |  | A*74:01~B*15:10~C*03:04 | 0.0003 |  |  |  |  | A*30:01~B*18:01~C*07:01 | 0.0003 | A*74:00~B*57:02~C*08:02 | 0.0008 |
|  |  |  |  | A*74:01~B*18:01~C*07:04 | 0.0003 |  |  |  |  | A*30:01~B*18:01~C*12:03 | 0.0006 | A*74:00~B*57:02~C*18:00 | 0.0008 |
|  |  |  |  | A*74:01~B*35:01~C*04:01 | 0.0069 |  |  |  |  | A*30:01~B*27:02~C*02:02 | 0.0003 | A*74:00~B*57:03~C*07:01 | 0.0008 |
|  |  |  |  | A*74:01~B*42:01~C*17:01 | 0.0013 |  |  |  |  | A*30:01~B*35:01~C*04:01 | 0.0003 | A*74:00~B*57:03~C*18:00 | 0.0015 |
|  |  |  |  | A*74:01~B*44:03~C*02:10 | 0.0003 |  |  |  |  | A*30:01~B*35:14~C*04:01 | 0.0003 | A*74:00~B*58:01~C*07:01 | 0.0008 |
|  |  |  |  | A*74:01~B*49:01~C*07:01 | 0.0003 |  |  |  |  | A*30:01~B*37:01~C*07:02 | 0.0003 | A*74:00~B*78:01~C*16:01 | 0.0008 |
|  |  |  |  | A*74:01~B*50:01~C*06:02 | 0.0003 |  |  |  |  | A*30:01~B*40:02~C*02:02 | 0.0003 | A*74:00~B*81:01~C*18:00 | 0.0015 |
|  |  |  |  | A*74:01~B*57:03~C*07:01 | 0.0046 |  |  |  |  | A*30:01~B*42:01~C*17:01 | 0.0003 | A*74:01~B*07:02~C*07:02 | 0.0023 |
|  |  |  |  | A*74:01~B*58:01~C*02:10 | 0.0003 |  |  |  |  | A*30:01~B*42:02~C*18:00 | 0.0003 | A*74:01~B*07:02~C*15:05 | 0.0008 |
|  |  |  |  | A*74:01~B*58:01~C*03:02 | 0.0006 |  |  |  |  | A*30:01~B*45:01~C*06:02 | 0.0003 | A*74:01~B*07:05~C*15:05 | 0.0015 |
|  |  |  |  | A*74:01~B*58:01~C*04:01 | 0.0003 |  |  |  |  | A*30:01~B*49:01~C*07:01 | 0.0003 | A*74:01~B*08:01~C*05:01 | 0.0008 |
|  |  |  |  | A*74:01~B*81:01~C*04:01 | 0.0003 |  |  |  |  | A*30:01~B*50:01~C*06:02 | 0.0003 | A*74:01~B*14:02~C*08:02 | 0.0015 |
|  |  |  |  | A*80:01~B*18:01~C*02:02 | 0.0082 |  |  |  |  | A*30:01~B*51:01~C*14:02 | 0.0003 | A*74:01~B*15:03~C*02:10 | 0.0134 |
|  |  |  |  | A*80:01~B*39:10~C*17:00 | 0.0003 |  |  |  |  | A*30:01~B*53:01~C*04:01 | 0.0008 | A*74:01~B*15:10~C*08:04 | 0.0008 |
|  |  |  |  | A*80:01~B*42:01~C*02:02 | 0.0003 |  |  |  |  | A*30:01~B*53:01~C*06:02 | 0.0003 | A*74:01~B*15:16~C*14:02 | 0.0008 |
|  |  |  |  |  |  |  |  |  |  | A*30:01~B*56:01~C*01:02 | 0.0003 | A*74:01~B*15:17~C*05:01 | 0.0008 |
|  |  |  |  |  |  |  |  |  |  | A*30:01~B*57:03~C*17:01 | 0.0003 | A*74:01~B*18:01~C*02:10 | 0.0015 |
|  |  |  |  |  |  |  |  |  |  | A*30:01~B*58:02~C*06:02 | 0.0003 | A*74:01~B*27:03~C*02:02 | 0.0008 |
|  |  |  |  |  |  |  |  |  |  | A*30:02~B*08:01~C*07:01 | 0.0008 | A*74:01~B*35:01~C*04:01 | 0.0026 |
|  |  |  |  |  |  |  |  |  |  | A*30:02~B*14:02~C*08:02 | 0.0003 | A*74:01~B*35:01~C*16:01 | 0.0008 |
|  |  |  |  |  |  |  |  |  |  | A*30:02~B*15:01~C*01:02 | 0.0003 | A*74:01~B*35:02~C*04:01 | 0.0008 |
|  |  |  |  |  |  |  |  |  |  | A*30:02~B*15:03~C*12:03 | 0.0003 | A*74:01~B*42:01~C*17:00 | 0.0008 |
|  |  |  |  |  |  |  |  |  |  | A*30:02~B*18:01~C*02:02 | 0.0002 | A*74:01~B*44:03~C*04:01 | 0.0008 |
|  |  |  |  |  |  |  |  |  |  | A*30:02~B*18:01~C*05:01 | 0.0029 | A*74:01~B*45:01~C*16:01 | 0.0004 |
|  |  |  |  |  |  |  |  |  |  | A*30:02~B*40:02~C*03:04 | 0.0003 | A*74:01~B*49:01~C*07:01 | 0.0019 |
|  |  |  |  |  |  |  |  |  |  | A*30:02~B*40:16~C*07:02 | 0.0003 | A*74:01~B*51:01~C*16:01 | 0.0008 |
|  |  |  |  |  |  |  |  |  |  | A*30:02~B*42:01~C*17:01 | 0.0003 | A*74:01~B*52:01~C*16:01 | 0.0031 |
|  |  |  |  |  |  |  |  |  |  | A*30:02~B*44:02~C*05:01 | 0.0006 | A*74:01~B*53:01~C*04:01 | 0.0034 |
|  |  |  |  |  |  |  |  |  |  | A*30:02~B*44:03~C*16:01 | 0.0008 | A*74:01~B*53:01~C*17:01 | 0.0008 |
|  |  |  |  |  |  |  |  |  |  | A*30:02~B*45:01~C*08:04 | 0.0003 | A*74:01~B*57:02~C*18:02 | 0.0008 |
|  |  |  |  |  |  |  |  |  |  | A*30:02~B*49:01~C*07:01 | 0.0003 | A*74:01~B*57:03~C*07:01 | 0.0092 |
|  |  |  |  |  |  |  |  |  |  | A*30:02~B*53:01~C*04:01 | 0.0014 | A*74:01~B*57:03~C*18:02 | 0.0009 |
|  |  |  |  |  |  |  |  |  |  | A*30:02~B*57:03~C*18:00 | 0.0006 | A*74:01~B*81:01~C*08:04 | 0.0025 |
|  |  |  |  |  |  |  |  |  |  | A*30:02~B*57:03~C*18:02 | 0.0003 | A*74:01~B*81:01~C*18:00 | 0.0025 |
|  |  |  |  |  |  |  |  |  |  | A*30:02~B*58:01~C*03:02 | 0.0006 | A*74:01~B*82:01~C*03:02 | 0.0008 |
|  |  |  |  |  |  |  |  |  |  | A*30:04~B*14:01~C*08:02 | 0.0006 | A*74:03~B*41:01~C*17:00 | 0.0008 |
|  |  |  |  |  |  |  |  |  |  | A*30:04~B*14:02~C*08:02 | 0.0003 | A*74:03~B*81:01~C*07:04 | 0.0008 |
|  |  |  |  |  |  |  |  |  |  | A*30:04~B*15:01~C*07:02 | 0.0003 | A*74:03~B*81:01~C*08:04 | 0.0015 |
|  |  |  |  |  |  |  |  |  |  | A*30:04~B*15:17~C*07:01 | 0.0003 | A*74:03~B*82:02~C*16:01 | 0.0008 |
|  |  |  |  |  |  |  |  |  |  | A*30:04~B*27:02~C*07:01 | 0.0003 | A*74:11~B*15:03~C*02:10 | 0.0008 |
|  |  |  |  |  |  |  |  |  |  | A*30:04~B*39:08~C*07:02 | 0.0003 | A*80:01~B*08:01~C*07:01 | 0.0008 |
|  |  |  |  |  |  |  |  |  |  | A*30:04~B*41:01~C*17:01 | 0.0003 | A*80:01~B*15:03~C*02:10 | 0.0008 |
|  |  |  |  |  |  |  |  |  |  | A*30:04~B*44:03~C*07:01 | 0.0003 | A*80:01~B*18:01~C*02:02 | 0.0008 |
|  |  |  |  |  |  |  |  |  |  | A*30:04~B*49:01~C*07:01 | 0.0003 | A*80:01~B*18:01~C*02:10 | 0.0008 |
|  |  |  |  |  |  |  |  |  |  | A*30:04~B*52:01~C*12:02 | 0.0003 | A*80:01~B*35:01~C*06:02 | 0.0008 |
|  |  |  |  |  |  |  |  |  |  | A*30:04~B*58:02~C*06:02 | 0.0003 | A*80:01~B*44:03~C*04:01 | 0.0015 |
|  |  |  |  |  |  |  |  |  |  | A*30:10~B*41:01~C*06:02 | 0.0006 | A*80:01~B*57:03~C*07:01 | 0.0008 |
|  |  |  |  |  |  |  |  |  |  | A*31:01~B*07:02~C*03:04 | 0.0003 | A*80:01~B*81:01~C*18:00 | 0.0008 |
|  |  |  |  |  |  |  |  |  |  | A*31:01~B*07:02~C*07:02 | 0.0010 |  |  |
|  |  |  |  |  |  |  |  |  |  | A*31:01~B*08:01~C*07:01 | 0.0017 |  |  |
|  |  |  |  |  |  |  |  |  |  | A*31:01~B*08:02~C*07:01 | 0.0003 |  |  |
|  |  |  |  |  |  |  |  |  |  | A*31:01~B*13:02~C*06:02 | 0.0007 |  |  |
|  |  |  |  |  |  |  |  |  |  | A*31:01~B*14:01~C*08:02 | 0.0006 |  |  |
|  |  |  |  |  |  |  |  |  |  | A*31:01~B*14:02~C*08:02 | 0.0006 |  |  |
|  |  |  |  |  |  |  |  |  |  | A*31:01~B*14:02~C*12:03 | 0.0003 |  |  |
|  |  |  |  |  |  |  |  |  |  | A*31:01~B*15:01~C*03:03 | 0.0010 |  |  |
|  |  |  |  |  |  |  |  |  |  | A*31:01~B*15:01~C*04:01 | 0.0003 |  |  |
|  |  |  |  |  |  |  |  |  |  | A*31:01~B*15:220~C*12:03 | 0.0003 |  |  |
|  |  |  |  |  |  |  |  |  |  | A*31:01~B*18:01~C*07:01 | 0.0007 |  |  |
|  |  |  |  |  |  |  |  |  |  | A*31:01~B*27:02~C*02:02 | 0.0003 |  |  |
|  |  |  |  |  |  |  |  |  |  | A*31:01~B*27:05~C*01:02 | 0.0003 |  |  |
|  |  |  |  |  |  |  |  |  |  | A*31:01~B*27:05~C*01:127 | 0.0006 |  |  |
|  |  |  |  |  |  |  |  |  |  | A*31:01~B*27:05~C*02:02 | 0.0015 |  |  |
|  |  |  |  |  |  |  |  |  |  | A*31:01~B*35:01~C*04:01 | 0.0014 |  |  |
|  |  |  |  |  |  |  |  |  |  | A*31:01~B*35:01~C*14:02 | 0.0003 |  |  |
|  |  |  |  |  |  |  |  |  |  | A*31:01~B*35:02~C*04:01 | 0.0004 |  |  |
|  |  |  |  |  |  |  |  |  |  | A*31:01~B*35:03~C*12:03 | 0.0003 |  |  |
|  |  |  |  |  |  |  |  |  |  | A*31:01~B*35:08~C*04:01 | 0.0008 |  |  |
|  |  |  |  |  |  |  |  |  |  | A*31:01~B*38:01~C*12:03 | 0.0003 |  |  |
|  |  |  |  |  |  |  |  |  |  | A*31:01~B*39:01~C*12:03 | 0.0007 |  |  |
|  |  |  |  |  |  |  |  |  |  | A*31:01~B*39:12~C*07:02 | 0.0003 |  |  |
|  |  |  |  |  |  |  |  |  |  | A*31:01~B*40:01~C*03:04 | 0.0076 |  |  |
|  |  |  |  |  |  |  |  |  |  | A*31:01~B*40:02~C*02:02 | 0.0004 |  |  |
|  |  |  |  |  |  |  |  |  |  | A*31:01~B*40:02~C*04:01 | 0.0003 |  |  |
|  |  |  |  |  |  |  |  |  |  | A*31:01~B*40:31~C*03:04 | 0.0003 |  |  |
|  |  |  |  |  |  |  |  |  |  | A*31:01~B*41:02~C*17:00 | 0.0006 |  |  |
|  |  |  |  |  |  |  |  |  |  | A*31:01~B*44:02~C*05:01 | 0.0010 |  |  |
|  |  |  |  |  |  |  |  |  |  | A*31:01~B*44:03~C*04:01 | 0.0005 |  |  |
|  |  |  |  |  |  |  |  |  |  | A*31:01~B*44:04~C*16:01 | 0.0003 |  |  |
|  |  |  |  |  |  |  |  |  |  | A*31:01~B*45:01~C*06:02 | 0.0003 |  |  |
|  |  |  |  |  |  |  |  |  |  | A*31:01~B*49:01~C*07:01 | 0.0003 |  |  |
|  |  |  |  |  |  |  |  |  |  | A*31:01~B*49:01~C*07:02 | 0.0006 |  |  |
|  |  |  |  |  |  |  |  |  |  | A*31:01~B*51:01~C*03:04 | 0.0003 |  |  |
|  |  |  |  |  |  |  |  |  |  | A*31:01~B*51:01~C*04:01 | 0.0003 |  |  |
|  |  |  |  |  |  |  |  |  |  | A*31:01~B*51:01~C*14:02 | 0.0005 |  |  |
|  |  |  |  |  |  |  |  |  |  | A*31:01~B*51:01~C*15:02 | 0.0044 |  |  |
|  |  |  |  |  |  |  |  |  |  | A*31:01~B*51:09~C*01:02 | 0.0003 |  |  |
|  |  |  |  |  |  |  |  |  |  | A*31:01~B*55:01~C*03:03 | 0.0009 |  |  |
|  |  |  |  |  |  |  |  |  |  | A*31:01~B*56:01~C*01:02 | 0.0003 |  |  |
|  |  |  |  |  |  |  |  |  |  | A*31:01~B*57:01~C*03:04 | 0.0003 |  |  |
|  |  |  |  |  |  |  |  |  |  | A*31:01~B*57:01~C*06:02 | 0.0009 |  |  |
|  |  |  |  |  |  |  |  |  |  | A*31:01~B*57:01~C*15:02 | 0.0003 |  |  |
|  |  |  |  |  |  |  |  |  |  | A*31:01~B*57:03~C*12:03 | 0.0003 |  |  |
|  |  |  |  |  |  |  |  |  |  | A*31:02~B*49:01~C*03:04 | 0.0003 |  |  |
|  |  |  |  |  |  |  |  |  |  | A*32:01~B*07:02~C*07:02 | 0.0004 |  |  |
|  |  |  |  |  |  |  |  |  |  | A*32:01~B*07:05~C*15:05 | 0.0006 |  |  |
|  |  |  |  |  |  |  |  |  |  | A*32:01~B*08:01~C*07:01 | 0.0020 |  |  |
|  |  |  |  |  |  |  |  |  |  | A*32:01~B*14:01~C*08:02 | 0.0051 |  |  |
|  |  |  |  |  |  |  |  |  |  | A*32:01~B*14:02~C*08:02 | 0.0014 |  |  |
|  |  |  |  |  |  |  |  |  |  | A*32:01~B*15:01~C*03:03 | 0.0023 |  |  |
|  |  |  |  |  |  |  |  |  |  | A*32:01~B*15:03~C*16:01 | 0.0003 |  |  |
|  |  |  |  |  |  |  |  |  |  | A*32:01~B*15:17~C*07:01 | 0.0003 |  |  |
|  |  |  |  |  |  |  |  |  |  | A*32:01~B*15:24~C*02:02 | 0.0003 |  |  |
|  |  |  |  |  |  |  |  |  |  | A*32:01~B*18:01~C*02:02 | 0.0003 |  |  |
|  |  |  |  |  |  |  |  |  |  | A*32:01~B*18:01~C*07:01 | 0.0012 |  |  |
|  |  |  |  |  |  |  |  |  |  | A*32:01~B*27:02~C*01:02 | 0.0003 |  |  |
|  |  |  |  |  |  |  |  |  |  | A*32:01~B*27:02~C*02:02 | 0.0003 |  |  |
|  |  |  |  |  |  |  |  |  |  | A*32:01~B*27:05~C*01:02 | 0.0035 |  |  |
|  |  |  |  |  |  |  |  |  |  | A*32:01~B*27:05~C*02:02 | 0.0005 |  |  |
|  |  |  |  |  |  |  |  |  |  | A*32:01~B*27:05~C*05:01 | 0.0003 |  |  |
|  |  |  |  |  |  |  |  |  |  | A*32:01~B*27:09~C*18:02 | 0.0003 |  |  |
|  |  |  |  |  |  |  |  |  |  | A*32:01~B*35:01~C*04:01 | 0.0023 |  |  |
|  |  |  |  |  |  |  |  |  |  | A*32:01~B*35:03~C*04:01 | 0.0003 |  |  |
|  |  |  |  |  |  |  |  |  |  | A*32:01~B*35:03~C*12:03 | 0.0003 |  |  |
|  |  |  |  |  |  |  |  |  |  | A*32:01~B*35:187~C*15:05 | 0.0003 |  |  |
|  |  |  |  |  |  |  |  |  |  | A*32:01~B*35:32~C*01:02 | 0.0003 |  |  |
|  |  |  |  |  |  |  |  |  |  | A*32:01~B*38:01~C*01:02 | 0.0003 |  |  |
|  |  |  |  |  |  |  |  |  |  | A*32:01~B*39:01~C*07:02 | 0.0002 |  |  |
|  |  |  |  |  |  |  |  |  |  | A*32:01~B*39:06~C*12:03 | 0.0003 |  |  |
|  |  |  |  |  |  |  |  |  |  | A*32:01~B*40:01~C*03:04 | 0.0032 |  |  |
|  |  |  |  |  |  |  |  |  |  | A*32:01~B*40:01~C*05:01 | 0.0003 |  |  |
|  |  |  |  |  |  |  |  |  |  | A*32:01~B*40:02~C*02:02 | 0.0047 |  |  |
|  |  |  |  |  |  |  |  |  |  | A*32:01~B*40:02~C*15:02 | 0.0001 |  |  |
|  |  |  |  |  |  |  |  |  |  | A*32:01~B*41:01~C*17:01 | 0.0002 |  |  |
|  |  |  |  |  |  |  |  |  |  | A*32:01~B*44:02~C*05:01 | 0.0060 |  |  |
|  |  |  |  |  |  |  |  |  |  | A*32:01~B*44:03~C*04:01 | 0.0004 |  |  |
|  |  |  |  |  |  |  |  |  |  | A*32:01~B*47:01~C*06:02 | 0.0003 |  |  |
|  |  |  |  |  |  |  |  |  |  | A*32:01~B*50:01~C*06:02 | 0.0003 |  |  |
|  |  |  |  |  |  |  |  |  |  | A*32:01~B*51:01~C*02:02 | 0.0005 |  |  |
|  |  |  |  |  |  |  |  |  |  | A*32:01~B*51:01~C*15:02 | 0.0011 |  |  |
|  |  |  |  |  |  |  |  |  |  | A*32:01~B*51:07~C*14:02 | 0.0003 |  |  |
|  |  |  |  |  |  |  |  |  |  | A*32:01~B*52:01~C*12:02 | 0.0003 |  |  |
|  |  |  |  |  |  |  |  |  |  | A*32:01~B*53:01~C*01:02 | 0.0003 |  |  |
|  |  |  |  |  |  |  |  |  |  | A*32:01~B*55:01~C*01:02 | 0.0003 |  |  |
|  |  |  |  |  |  |  |  |  |  | A*32:01~B*55:01~C*03:03 | 0.0011 |  |  |
|  |  |  |  |  |  |  |  |  |  | A*32:01~B*55:01~C*03:04 | 0.0003 |  |  |
|  |  |  |  |  |  |  |  |  |  | A*32:01~B*57:01~C*06:02 | 0.0025 |  |  |
|  |  |  |  |  |  |  |  |  |  | A*32:01~B*81:01~C*16:01 | 0.0003 |  |  |
|  |  |  |  |  |  |  |  |  |  | A*32:114~B*39:01~C*12:03 | 0.0003 |  |  |
|  |  |  |  |  |  |  |  |  |  | A*33:01~B*14:02~C*08:02 | 0.0076 |  |  |
|  |  |  |  |  |  |  |  |  |  | A*33:01~B*15:03~C*02:10 | 0.0003 |  |  |
|  |  |  |  |  |  |  |  |  |  | A*33:01~B*27:05~C*07:04 | 0.0003 |  |  |
|  |  |  |  |  |  |  |  |  |  | A*33:01~B*35:02~C*04:01 | 0.0003 |  |  |
|  |  |  |  |  |  |  |  |  |  | A*33:01~B*44:02~C*07:04 | 0.0003 |  |  |
|  |  |  |  |  |  |  |  |  |  | A*33:01~B*44:03~C*02:02 | 0.0006 |  |  |
|  |  |  |  |  |  |  |  |  |  | A*33:03~B*07:05~C*15:05 | 0.0003 |  |  |
|  |  |  |  |  |  |  |  |  |  | A*33:03~B*08:01~C*07:01 | 0.0004 |  |  |
|  |  |  |  |  |  |  |  |  |  | A*33:03~B*13:02~C*04:01 | 0.0003 |  |  |
|  |  |  |  |  |  |  |  |  |  | A*33:03~B*15:16~C*14:02 | 0.0003 |  |  |
|  |  |  |  |  |  |  |  |  |  | A*33:03~B*15:17~C*03:02 | 0.0003 |  |  |
|  |  |  |  |  |  |  |  |  |  | A*33:03~B*18:01~C*12:03 | 0.0003 |  |  |
|  |  |  |  |  |  |  |  |  |  | A*33:03~B*35:02~C*04:01 | 0.0003 |  |  |
|  |  |  |  |  |  |  |  |  |  | A*33:03~B*35:08~C*04:01 | 0.0006 |  |  |
|  |  |  |  |  |  |  |  |  |  | A*33:03~B*50:01~C*04:01 | 0.0003 |  |  |
|  |  |  |  |  |  |  |  |  |  | A*33:03~B*50:01~C*06:02 | 0.0006 |  |  |
|  |  |  |  |  |  |  |  |  |  | A*33:03~B*57:01~C*06:02 | 0.0005 |  |  |
|  |  |  |  |  |  |  |  |  |  | A*33:03~B*58:01~C*03:02 | 0.0008 |  |  |
|  |  |  |  |  |  |  |  |  |  | A*33:03~B*81:01~C*07:02 | 0.0003 |  |  |
|  |  |  |  |  |  |  |  |  |  | A*33:05~B*14:02~C*08:02 | 0.0003 |  |  |
|  |  |  |  |  |  |  |  |  |  | A*34:02~B*08:01~C*07:01 | 0.0014 |  |  |
|  |  |  |  |  |  |  |  |  |  | A*34:02~B*35:03~C*04:01 | 0.0003 |  |  |
|  |  |  |  |  |  |  |  |  |  | A*34:02~B*39:10~C*12:03 | 0.0003 |  |  |
|  |  |  |  |  |  |  |  |  |  | A*34:02~B*53:01~C*04:01 | 0.0003 |  |  |
|  |  |  |  |  |  |  |  |  |  | A*36:01~B*44:02~C*05:01 | 0.0006 |  |  |
|  |  |  |  |  |  |  |  |  |  | A*36:01~B*53:01~C*04:01 | 0.0008 |  |  |
|  |  |  |  |  |  |  |  |  |  | A*66:01~B*07:05~C*15:05 | 0.0003 |  |  |
|  |  |  |  |  |  |  |  |  |  | A*66:01~B*14:02~C*08:02 | 0.0003 |  |  |
|  |  |  |  |  |  |  |  |  |  | A*66:01~B*15:220~C*12:03 | 0.0003 |  |  |
|  |  |  |  |  |  |  |  |  |  | A*66:01~B*35:02~C*17:03 | 0.0003 |  |  |
|  |  |  |  |  |  |  |  |  |  | A*66:01~B*38:01~C*12:03 | 0.0008 |  |  |
|  |  |  |  |  |  |  |  |  |  | A*66:01~B*41:01~C*17:00 | 0.0003 |  |  |
|  |  |  |  |  |  |  |  |  |  | A*66:01~B*41:02~C*17:00 | 0.0003 |  |  |
|  |  |  |  |  |  |  |  |  |  | A*66:01~B*41:02~C*17:03 | 0.0008 |  |  |
|  |  |  |  |  |  |  |  |  |  | A*66:01~B*51:01~C*16:02 | 0.0003 |  |  |
|  |  |  |  |  |  |  |  |  |  | A*66:01~B*53:01~C*06:02 | 0.0003 |  |  |
|  |  |  |  |  |  |  |  |  |  | A*68:01~B*07:02~C*07:02 | 0.0013 |  |  |
|  |  |  |  |  |  |  |  |  |  | A*68:01~B*07:02~C*07:04 | 0.0003 |  |  |
|  |  |  |  |  |  |  |  |  |  | A*68:01~B*08:01~C*07:01 | 0.0007 |  |  |
|  |  |  |  |  |  |  |  |  |  | A*68:01~B*08:01~C*08:02 | 0.0003 |  |  |
|  |  |  |  |  |  |  |  |  |  | A*68:01~B*13:02~C*06:02 | 0.0005 |  |  |
|  |  |  |  |  |  |  |  |  |  | A*68:01~B*14:01~C*08:02 | 0.0005 |  |  |
|  |  |  |  |  |  |  |  |  |  | A*68:01~B*15:01~C*03:03 | 0.0008 |  |  |
|  |  |  |  |  |  |  |  |  |  | A*68:01~B*15:16~C*14:02 | 0.0003 |  |  |
|  |  |  |  |  |  |  |  |  |  | A*68:01~B*15:18~C*07:04 | 0.0005 |  |  |
|  |  |  |  |  |  |  |  |  |  | A*68:01~B*15:31~C*04:07 | 0.0003 |  |  |
|  |  |  |  |  |  |  |  |  |  | A*68:01~B*18:01~C*05:01 | 0.0003 |  |  |
|  |  |  |  |  |  |  |  |  |  | A*68:01~B*18:01~C*07:01 | 0.0003 |  |  |
|  |  |  |  |  |  |  |  |  |  | A*68:01~B*27:05~C*01:02 | 0.0003 |  |  |
|  |  |  |  |  |  |  |  |  |  | A*68:01~B*27:05~C*02:02 | 0.0011 |  |  |
|  |  |  |  |  |  |  |  |  |  | A*68:01~B*27:05~C*07:04 | 0.0003 |  |  |
|  |  |  |  |  |  |  |  |  |  | A*68:01~B*35:01~C*04:01 | 0.0006 |  |  |
|  |  |  |  |  |  |  |  |  |  | A*68:01~B*35:02~C*02:02 | 0.0003 |  |  |
|  |  |  |  |  |  |  |  |  |  | A*68:01~B*35:03~C*04:01 | 0.0022 |  |  |
|  |  |  |  |  |  |  |  |  |  | A*68:01~B*35:05~C*04:01 | 0.0003 |  |  |
|  |  |  |  |  |  |  |  |  |  | A*68:01~B*35:08~C*04:01 | 0.0003 |  |  |
|  |  |  |  |  |  |  |  |  |  | A*68:01~B*38:01~C*12:03 | 0.0012 |  |  |
|  |  |  |  |  |  |  |  |  |  | A*68:01~B*39:01~C*12:03 | 0.0003 |  |  |
|  |  |  |  |  |  |  |  |  |  | A*68:01~B*40:01~C*03:03 | 0.0002 |  |  |
|  |  |  |  |  |  |  |  |  |  | A*68:01~B*40:01~C*03:04 | 0.0008 |  |  |
|  |  |  |  |  |  |  |  |  |  | A*68:01~B*40:02~C*02:02 | 0.0002 |  |  |
|  |  |  |  |  |  |  |  |  |  | A*68:01~B*40:02~C*03:04 | 0.0003 |  |  |
|  |  |  |  |  |  |  |  |  |  | A*68:01~B*40:04~C*03:04 | 0.0003 |  |  |
|  |  |  |  |  |  |  |  |  |  | A*68:01~B*44:02~C*05:01 | 0.0011 |  |  |
|  |  |  |  |  |  |  |  |  |  | A*68:01~B*44:02~C*07:04 | 0.0039 |  |  |
|  |  |  |  |  |  |  |  |  |  | A*68:01~B*44:03~C*07:02 | 0.0003 |  |  |
|  |  |  |  |  |  |  |  |  |  | A*68:01~B*44:03~C*16:01 | 0.0003 |  |  |
|  |  |  |  |  |  |  |  |  |  | A*68:01~B*47:01~C*03:04 | 0.0003 |  |  |
|  |  |  |  |  |  |  |  |  |  | A*68:01~B*47:01~C*06:02 | 0.0002 |  |  |
|  |  |  |  |  |  |  |  |  |  | A*68:01~B*51:01~C*01:02 | 0.0005 |  |  |
|  |  |  |  |  |  |  |  |  |  | A*68:01~B*51:01~C*02:02 | 0.0002 |  |  |
|  |  |  |  |  |  |  |  |  |  | A*68:01~B*51:01~C*04:01 | 0.0003 |  |  |
|  |  |  |  |  |  |  |  |  |  | A*68:01~B*51:01~C*14:02 | 0.0009 |  |  |
|  |  |  |  |  |  |  |  |  |  | A*68:01~B*51:01~C*15:02 | 0.0020 |  |  |
|  |  |  |  |  |  |  |  |  |  | A*68:01~B*52:01~C*03:04 | 0.0006 |  |  |
|  |  |  |  |  |  |  |  |  |  | A*68:01~B*52:01~C*12:02 | 0.0006 |  |  |
|  |  |  |  |  |  |  |  |  |  | A*68:01~B*52:01~C*16:01 | 0.0003 |  |  |
|  |  |  |  |  |  |  |  |  |  | A*68:01~B*55:01~C*03:03 | 0.0003 |  |  |
|  |  |  |  |  |  |  |  |  |  | A*68:01~B*57:01~C*06:02 | 0.0012 |  |  |
|  |  |  |  |  |  |  |  |  |  | A*68:02~B*14:02~C*08:02 | 0.0055 |  |  |
|  |  |  |  |  |  |  |  |  |  | A*68:02~B*14:06~C*08:02 | 0.0003 |  |  |
|  |  |  |  |  |  |  |  |  |  | A*68:02~B*15:10~C*03:04 | 0.0004 |  |  |
|  |  |  |  |  |  |  |  |  |  | A*68:02~B*18:01~C*05:01 | 0.0004 |  |  |
|  |  |  |  |  |  |  |  |  |  | A*68:02~B*27:05~C*01:02 | 0.0004 |  |  |
|  |  |  |  |  |  |  |  |  |  | A*68:02~B*38:01~C*12:03 | 0.0003 |  |  |
|  |  |  |  |  |  |  |  |  |  | A*68:02~B*40:02~C*02:02 | 0.0002 |  |  |
|  |  |  |  |  |  |  |  |  |  | A*68:02~B*41:01~C*17:00 | 0.0003 |  |  |
|  |  |  |  |  |  |  |  |  |  | A*68:02~B*49:01~C*07:01 | 0.0003 |  |  |
|  |  |  |  |  |  |  |  |  |  | A*68:02~B*53:01~C*04:01 | 0.0028 |  |  |
|  |  |  |  |  |  |  |  |  |  | A*68:02~B*57:01~C*12:03 | 0.0003 |  |  |
|  |  |  |  |  |  |  |  |  |  | A*68:02~B*57:03~C*07:01 | 0.0033 |  |  |
|  |  |  |  |  |  |  |  |  |  | A*68:03~B*39:08~C*07:02 | 0.0003 |  |  |
|  |  |  |  |  |  |  |  |  |  | A*68:03~B*44:03~C*04:01 | 0.0003 |  |  |
|  |  |  |  |  |  |  |  |  |  | A*68:03~B*44:05~C*03:05 | 0.0003 |  |  |
|  |  |  |  |  |  |  |  |  |  | A*68:07~B*39:08~C*07:02 | 0.0003 |  |  |
|  |  |  |  |  |  |  |  |  |  | A*68:07~B*40:02~C*15:02 | 0.0003 |  |  |
|  |  |  |  |  |  |  |  |  |  | A*68:15~B*57:03~C*08:02 | 0.0003 |  |  |
|  |  |  |  |  |  |  |  |  |  | A*69:01~B*07:05~C*15:05 | 0.0003 |  |  |
|  |  |  |  |  |  |  |  |  |  | A*69:01~B*39:01~C*12:03 | 0.0003 |  |  |
|  |  |  |  |  |  |  |  |  |  | A*69:01~B*44:02~C*05:01 | 0.0003 |  |  |
|  |  |  |  |  |  |  |  |  |  | A*69:01~B*52:01~C*12:02 | 0.0006 |  |  |
|  |  |  |  |  |  |  |  |  |  | A*69:01~B*55:01~C*01:02 | 0.0008 |  |  |
|  |  |  |  |  |  |  |  |  |  | A*69:01~B*55:01~C*03:03 | 0.0003 |  |  |
|  |  |  |  |  |  |  |  |  |  | A*69:01~B*57:03~C*07:01 | 0.0003 |  |  |
|  |  |  |  |  |  |  |  |  |  | A*74:00~B*15:03~C*02:10 | 0.0003 |  |  |
|  |  |  |  |  |  |  |  |  |  | A*74:00~B*15:10~C*06:02 | 0.0003 |  |  |
|  |  |  |  |  |  |  |  |  |  | A*74:01~B*18:01~C*02:10 | 0.0003 |  |  |
|  |  |  |  |  |  |  |  |  |  | A*74:01~B*35:01~C*16:01 | 0.0003 |  |  |
|  |  |  |  |  |  |  |  |  |  | A*74:03~B*51:01~C*14:02 | 0.0003 |  |  |
|  |  |  |  |  |  |  |  |  |  | A*80:01~B*40:02~C*04:01 | 0.0003 |  |  |

**Supplementary Table 8|** Unique alleles in each country at different locus

| **Locus** | **Populations** | | | | | | | |
| --- | --- | --- | --- | --- | --- | --- | --- | --- |
|  | Kenya | Rwanda | South Africa | Uganda | Zambia | EUAM | | Africa America |
| A | Nil | A*26:03 | A*01:23 | A*31:03 | A*03new | A*02:09 | A*24:05 | A*01:22N |
|  |  | A*74:02 | A*03:21 |  | A*24:31 | A*02:17 | A*24:23 | A*02:16 |
|  |  | A*74:05 | A*23 new |  |  | A*02:20 | A*24:314 | A*11:02 |
|  |  |  | A*26:121 |  |  | A*02:22 | A*24:95 | A*30:151 |
|  |  |  | A*29:11 |  |  | A*02:274 | A*26:08 | A*68:10 |
|  |  |  | A*32:106 |  |  | A*02:30 | A*29:10 | A*74:11 |
|  |  |  | A*34:01 |  |  | A*02:724 | A*31:02 |  |
|  |  |  | A*68:04 |  |  | A*03:05 | A*32:114 |  |
|  |  |  | A*68:27 |  |  | A*03:21N | A*68:03 |  |
|  |  |  | A*69:02 |  |  | A*03:49 | A*68:07 |  |
|  |  |  |  |  |  | A*11:04 | A*68:15 |  |
|  |  |  |  |  |  | A*11:67 |  |  |
| B | Nil | Nil | B*13:01 | B*15:37 | B*07:51 | B*07:08 | B*39:08 | B*07:09 |
|  |  |  | B*15:08 |  | B*15:47 | B*08:02 | B*39:11 | B*27:06 |
|  |  |  | B*15:13 |  | B*15:83 | B*15:02 | B*39:12 | B*35:04 |
|  |  |  | B*15:22 |  | B*58:15 | B*15:11 | B*40:04 | B*39:20 |
|  |  |  | B*38:02 |  |  | B*15:40 | B*40:08 | B*41:03 |
|  |  |  | B*45:00 |  |  | B*18:05 | B*40:31 | B*44:10 |
|  |  |  | B*58:10 |  |  | B*18:20 | B*44:04 | B*51:02 |
|  |  |  | B*81:00 |  |  | B*27:07 | B*44:27 | B*51:64 |
|  |  |  | B*81:03 |  |  | B*27:09 | B*46:01 | B*58:11 |
|  |  |  |  |  |  | B*27:13 | B*48:02 | B*78:01 |
|  |  |  |  |  |  | B*35:12 | B*50:02 |  |
|  |  |  |  |  |  | B*35:14 | B*51:05 |  |
|  |  |  |  |  |  | B*35:17 | B*51:09 |  |
|  |  |  |  |  |  | B*35:187 | B*51:22 |  |
|  |  |  |  |  |  | B*35:32 | B*52:02 |  |
|  |  |  |  |  |  | B*35:41 | B*54:01 |  |
|  |  |  |  |  |  | B*35:42 | B*67:01 |  |
|  |  |  |  |  |  | B*39:02 |  |  |
| C | Nil | Nil | C*02:05 | Nil | C*06:03 | C*01:127 |  | C*04:13 |
|  |  |  | C*02:17 |  | C*17new | C*02:29 |  | C*04:27 |
|  |  |  | C*04:03 |  |  | C*03:06 |  | C*04:29 |
|  |  |  | C*04:04 |  |  | C*03:09 |  | C*06:08 |
|  |  |  | C*04:226 |  |  | C*04:08 |  | C*07:05 |
|  |  |  | C*07:18 |  |  | C*08:03 |  | C*07:621 |
|  |  |  |  |  |  | C*15:06 |  | C*08:13 |
|  |  |  |  |  |  | C*15:13 |  | C*08:43 |

**Supplementary Table 9|** Unique alleles between Africa, EUAM and Africa America populations at different locus

| **Locus** | **Population** | | | | |
| --- | --- | --- | --- | --- | --- |
|  | Africa | | EUAM | | Africa America |
| A | A*01:23 | A*31:03 | A*02:09 | A*24:05 | A*01:22N |
|  | A*02:14 | A*31:04 | A*02:17 | A*24:23 | A*02:16 |
|  | A*03 new | A*32:106 | A*02:20 | A*24:314 | A*11:02 |
|  | A*03:21 | A*34:01 | A*02:22 | A*24:95 | A*30:151 |
|  | A*23 new | A*43:01 | A*02:274 | A*26:08 | A*68:10 |
|  | A*23:02 | A*68:04 | A*02:30 | A*29:10 | A*74:11 |
|  | A*24:31 | A*68:27 | A*02:724 | A*31:02 |  |
|  | A*26:03 | A*69:02 | A*03:05 | A*32:114 |  |
|  | A*26:12 | A*74:02 | A*03:21N | A*68:03 |  |
|  | A*26:121 | A*74:05 | A*03:49 | A*68:07 |  |
|  | A*29:11 |  | A*11:04 | A*68:15 |  |
|  |  |  | A*11:67 |  |  |
| B | B*07:51 | B*18:03 | B*07:08 | B*39:08 | B*07:09 |
|  | B*13:01 | B*38:02 | B*08:02 | B*39:11 | B*27:06 |
|  | B*13:03 | B*45:00 | B*15:02 | B*39:12 | B*35:04 |
|  | B*15:08 | B*45:07 | B*15:11 | B*40:04 | B*39:20 |
|  | B*15:13 | B*47:03 | B*15:40 | B*40:08 | B*41:03 |
|  | B*15:22 | B*58:10 | B*18:05 | B*40:31 | B*44:10 |
|  | B*15:37 | B*58:15 | B*18:20 | B*44:04 | B*51:02 |
|  | B*15:47 | B*81:00 | B*27:07 | B*44:27 | B*51:64 |
|  | B*15:83 | B*81:03 | B*27:09 | B*46:01 | B*58:11 |
|  |  |  | B*27:13 | B*48:02 | B*78:01 |
|  |  |  | B*35:12 | B*50:02 |  |
|  |  |  | B*35:14 | B*51:05 |  |
|  |  |  | B*35:17 | B*51:09 |  |
|  |  |  | B*35:187 | B*51:22 |  |
|  |  |  | B*35:32 | B*52:02 |  |
|  |  |  | B*35:41 | B*54:01 |  |
|  |  |  | B*35:42 | B*67:01 |  |
|  |  |  | B*39:02 |  |  |
| C | C*02:05 | | C*01:127 | | C*04:13 |
|  | C*02:17 | | C*02:29 | | C*04:27 |
|  | C*04:03 | | C*03:06 | | C*04:29 |
|  | C*04:04 | | C*03:09 | | C*06:08 |
|  | C*04:226 | | C*04:08 | | C*07:05 |
|  | C*06:03 | | C*08:03 | | C*07:621 |
|  | C*07:18 | | C*15:06 | | C*08:13 |
|  | C*15:25 | | C*15:13 | | C*08:43 |
|  | C*17new | |  |  |  |

**Supplementary Table 10|** Exact test using Markov chain for HWE parameters for tribes.

| **Population** | **Tribe** |  | **Locus** | | | | | | | | |
| --- | --- | --- | --- | --- | --- | --- | --- | --- | --- | --- | --- |
|  |  | **No of**  **Gen.** | **A** | | | **B** | | | **C** | | |
|  |  |  | Obs. Het. | Exp. Het. | P-value (Adj) | Obs. Het. | Exp. Het. | P-value (Adj) | Obs. Het. | Exp. Het. | P-value (Adj) |
| Kenya | Kikuyu | 25 | 1.0000 | 0.9118 | 0.9858 | 1.0000 | 0.9420 | 0.7360 | 0.8800 | 0.8971 | 0.8028 |
|  | Luhya | 21 | 0.9048 | 0.9187 | 0.9858 | 0.9524 | 0.9431 | 0.9167 | 0.9524 | 0.9396 | 0.9976 |
| RSA | Zulu | 1624 | 0.9360 | 0.9428 | 0.8942 | 0.9323 | 0.9355 | 0.5781 | 0.9015 | 0.9167 | 0.0533 |
| Uganda | Muganda | 134 | 0.9552 | 0.9349 | 0.8942 | 0.9254 | 0.9460 | 0.7360 | 0.9105 | 0.9121 | 0.8028 |
|  | Munyankole | 25 | 1.0000 | 0.9404 | 0.9858 | 0.9600 | 0.9567 | 0.7360 | 0.9600 | 0.9208 | 0.8798 |
|  | Munyarwanda | 26 | 0.8462 | 0.9329 | 0.8942 | 0.9231 | 0.9419 | 0.9167 | 0.9231 | 0.8733 | 0.9898 |
| Zambia | Bemba | 142 | 0.9366 | 0.9256 | 0.9858 | 0.9648 | 0.9398 | 0.9167 | 0.9366 | 0.9038 | 0.6123 |
|  | Lozi | 23 | 0.8696 | 0.9353 | 0.8942 | 1.0000 | 0.9449 | 0.9167 | 0.9130 | 0.8831 | 0.9976 |
|  | Chewa | 63 | 0.9365 | 0.9281 | 0.9858 | 0.9361 | 0.9478 | 0.9167 | 0.9206 | 0.9209 | 0.8798 |
|  | Ngoni | 44 | 0.9318 | 0.9410 | 0.8942 | 0.8636 | 0.9436 | 0.0182* | 0.8636 | 0.9214 | 0.1671 |
|  | Nsenga | 70 | 0.9429 | 0.9248 | 0.9858 | 0.9286 | 0.9427 | 0.5781 | 0.8286 | 0.9081 | 0.2997 |
|  | Tonga | 29 | 0.8621 | 0.9141 | 0.8942 | 0.9655 | 0.9365 | 0.9167 | 0.9310 | 0.9220 | 0.9976 |
|  | Tumbuka | 29 | 0.9310 | 0.9135 | 0.9858 | 1.0000 | 0.9534 | 0.7360 | 0.9310 | 0.9135 | 0.2877 |

** Statistically significant)*. *Obs. Het., observed heterozygosity; Exp. Het., expected heterozygosity, No of Gen.; No of Genotypes*

**Supplementary Table 11|** Slatkin’s implementation of EW homozygosity test of neutrality for tribes

| **Population** | **Tribe** | **Locus** | | | | | | | | | | | |
| --- | --- | --- | --- | --- | --- | --- | --- | --- | --- | --- | --- | --- | --- |
|  |  | **A** | | | | **B** | | | | **C** | | | |
|  |  | Obs.  (Homo)  F | Exp.  (Homo)  F | Fnd | p-value  (Adj) | Obs.  (Homo) F | Exp.  (Homo)  F | Fnd | p-value  (Adj) | Obs.  (Homo)  F | Exp.  (Homo)  F | Fnd | p-value  (Adj) |
| Kenya | Kikuyu | 0.1064 | 0.1374 | -0.8468 | 0.2019 | 0.0768 | 0.0864 | -0.5239 | 0.3164 | 0.1208 | 0.1815 | -1.1345 | 0.0847 |
|  | Luhya | 0.1032 | 0.1164 | -0.4871 | 0.3742 | 0.0794 | 0.0986 | -0.9161 | 0.1620 | 0.0828 | 0.1164 | -1.2393 | 0.0784 |
| South Africa | Zulu | 0.0575 | 0.1110 | -1.4174 | 0.0598 | 0.0648 | 0.0939 | -0.9456 | 0.1620 | 0.0833 | 0.149 | -1.1909 | 0.0847 |
| Uganda | Muganda | 0.0686 | 0.1105 | -1.2079 | 0.0855 | 0.0575 | 0.1061 | -1.4699 | 0.0208* | 0.0913 | 0.1999 | -1.4871 | 0.0130* |
|  | Munyankole | 0.0784 | 0.1075 | -1.1403 | 0.0960 | 0.0624 | 0.0708 | -0.6445 | 0.2661 | 0.0976 | 0.1163 | -0.6459 | 0.2723 |
|  | Munyarwanda | 0.0851 | 0.1184 | -1.1095 | 0.0960 | 0.0762 | 0.1018 | -1.0687 | 0.1620 | 0.1435 | 0.2276 | -1.1864 | 0.0847 |
| Zambia | Bemba | 0.0777 | 0.1336 | -1.2609 | 0.0855 | 0.0635 | 0.0898 | -0.9922 | 0.1620 | 0.0994 | 0.1399 | -0.8526 | 0.2054 |
|  | Chewa | 0.0792 | 0.0811 | -0.0889 | 0.5663 | 0.0597 | 0.0777 | -0.9169 | 0.1620 | 0.0864 | 0.1537 | -1.3501 | 0.0455* |
|  | Lozi | 0.0851 | 0.0957 | -0.5097 | 0.3742 | 0.0756 | 0.0888 | -0.7196 | 0.2661 | 0.1361 | 0.176 | -0.7897 | 0.2242 |
|  | Ngoni | 0.0697 | 0.1052 | -1.2458 | 0.0855 | 0.0671 | 0.0889 | -0.9836 | 0.1620 | 0.0891 | 0.1193 | -0.8923 | 0.1963 |
|  | Nsenga | 0.0818 | 0.1250 | -1.1172 | 0.0960 | 0.0641 | 0.1127 | -1.4386 | 0.0208* | 0.0984 | 0.1587 | -1.1505 | 0.0847 |
|  | Tonga | 0.1017 | 0.1345 | -0.8944 | 0.1992 | 0.0797 | 0.1073 | -1.0326 | 0.1620 | 0.0939 | 0.1152 | -0.7140 | 0.2500 |
|  | Tumbuka | 0.1023 | 0.1345 | -0.8782 | 0.1992 | 0.0630 | 0.0932 | -1.3903 | 0.0303* | 0.1023 | 0.1461 | -1.0626 | 0.1029 |

**Statistically significant.*

**Supplementary Table 12|** Topmost haplotypes at different loci across populations

| **Populations** | **Loci** | | | | | | | |
| --- | --- | --- | --- | --- | --- | --- | --- | --- |
|  | **A:B** | | **A:C** | | **B:C** | | **A:B:C** | |
|  | A~B | HF | A~C | HF | B~C | HF | A~B~C | HF |
| Kenya | A*68:02~B*15:10 | 0.0548 | A*68:02~C*03:04 | 0.0596 | B*42:01~C*17:01 | 0.0734 | A*68:02~B*27:03~C*02:02 | 0.0596 |
| Rwanda | A*02:01~B*15:03 | 0.0513 | A*02:02~C*06:02 | 0.0571 | B*58:02~C*06:02 | 0.1098 | A*02:01~B*15:03~C*02:10 | 0.0484 |
| RSA | A*30:01~B*42:01 | 0.0465 | A*30:01~C*17:01 | 0.0355 | B*58:02~C*06:02 | 0.1134 | A*30:01~B*42:01~C*17:01 | 0.0275 |
| Uganda | A*02:01~B*15:03 | 0.0426 | A*02:01~C*02:10 | 0.0388 | B*53:01~C*04:01 | 0.0824 | A*02:01~B*15:03~C*02:10 | 0.0405 |
| Zambia | A*30:01~B*42:01 | 0.0480 | A*30:01~C*17:01 | 0.0557 | B*15:03~C*02:10 | 0.0937 | A*30:01~B*42:01~C*17:01 | 0.0463 |
| EUAM | A*01:01~B*08:01 | 0.0501 | A*01:01~C*07:01 | 0.0587 | B*07:02~C*07:02 | 0.0929 | A*01:01~B*08:01~C*07:01 | 0.0503 |
| AFAM | A*30:02~B*57:03 | 0.0267 | A*02:01~C*16:01 | 0.0234 | B*53:01~C*04:01 | 0.0763 | A*33:03~B*53:01~C*04:01 | 0.0151 |

*HF: Haplotype Frequency*


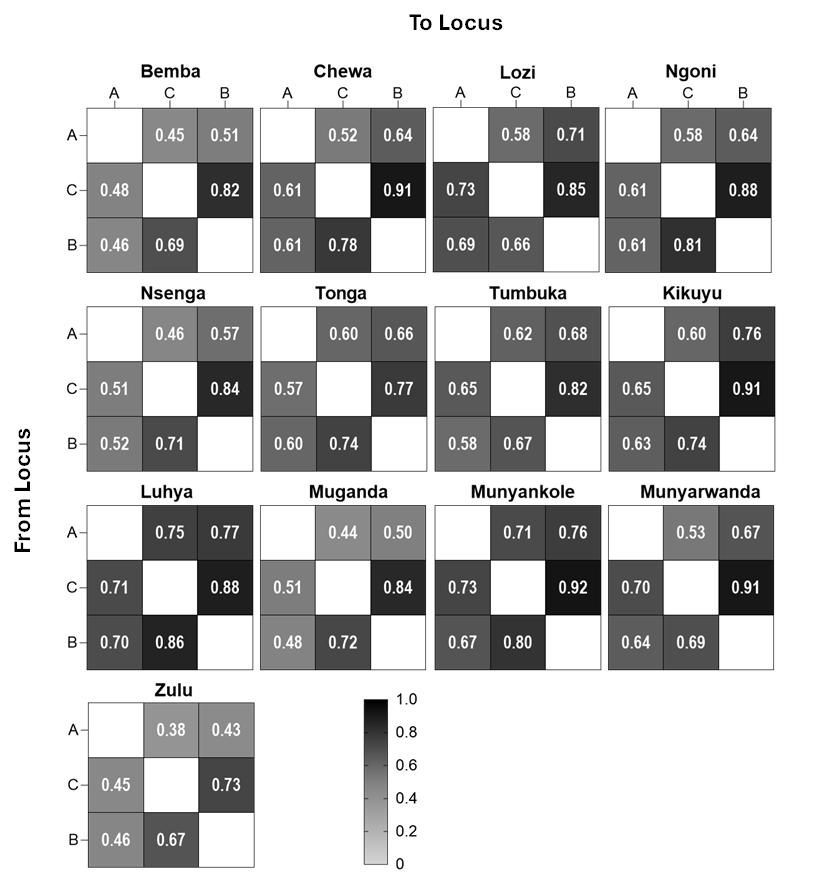


**Supplementary Figure 1|** Linkage Disequilibrium plot based on asymmetric linkage disequilibrium measures (row gene conditional on column gene) for HLA genes across tribes.


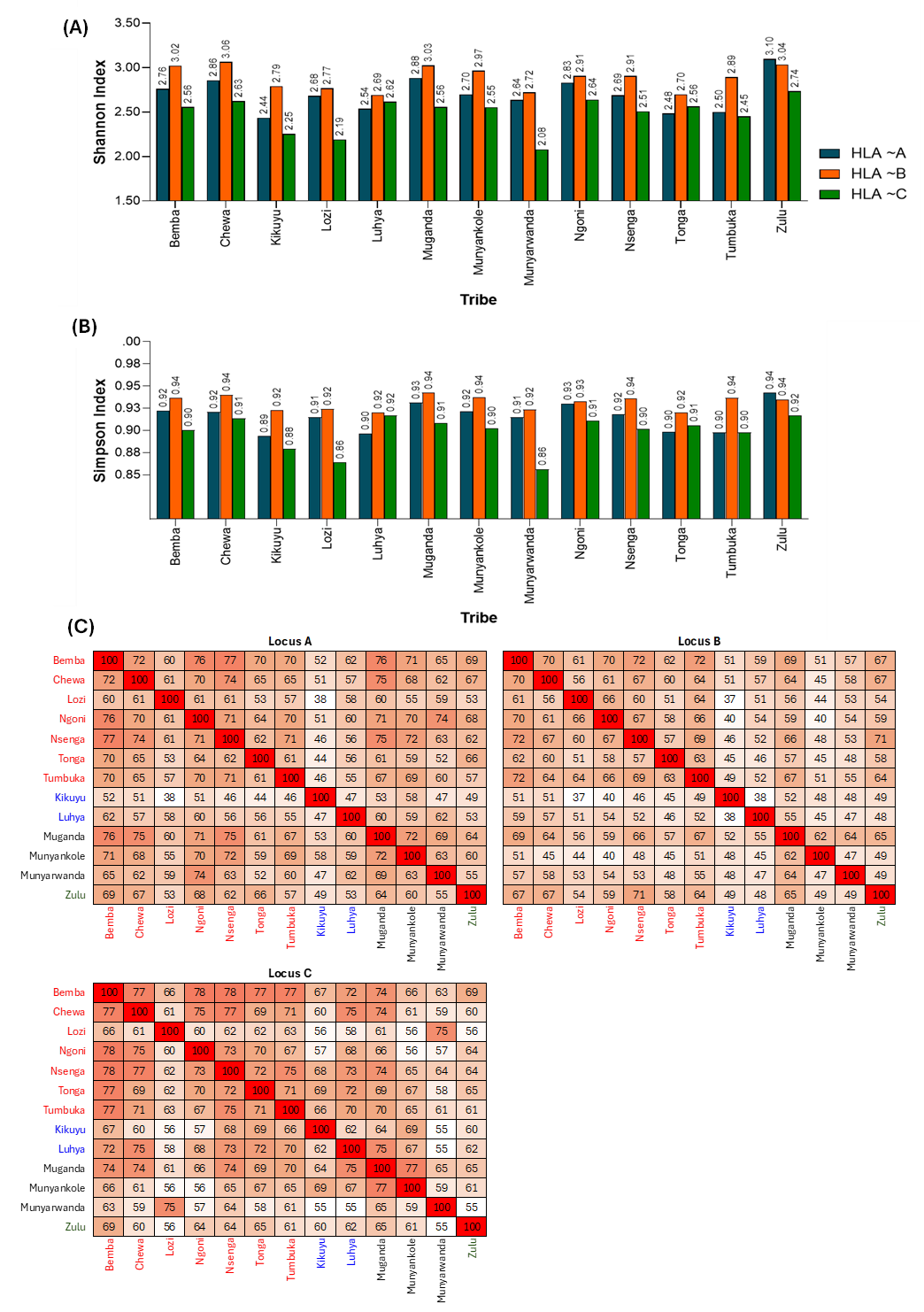


**Supplementary figure 2|** Graphs of Shannon **(A)**, Simpson **(B)** indices across African tribes and **(C)** non-clustered heatmap of similarity index (Jaccard) among tribes of the African populations.


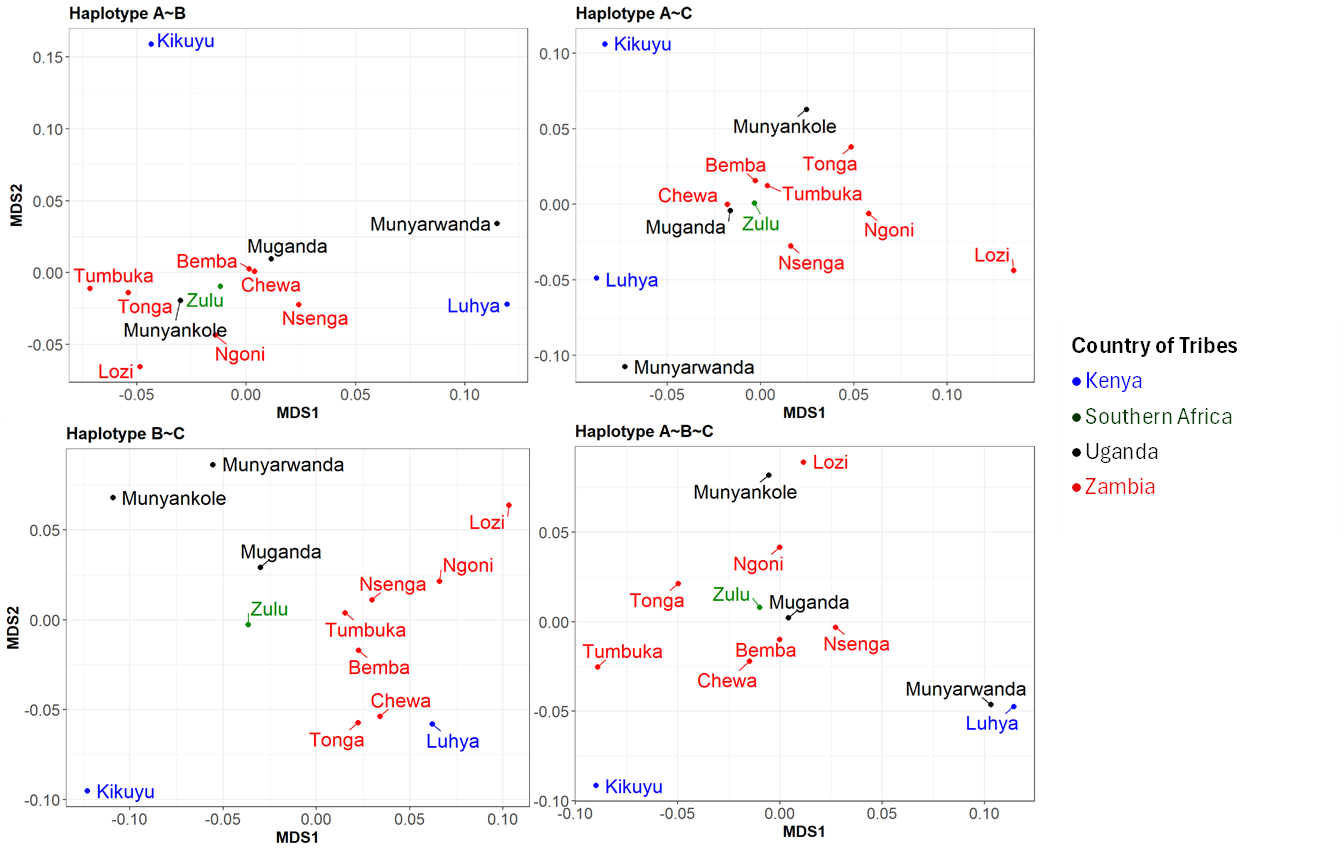


**Supplementary figure 3|** Cartography of the similarity in global haplotypes between tribes in the African populations.


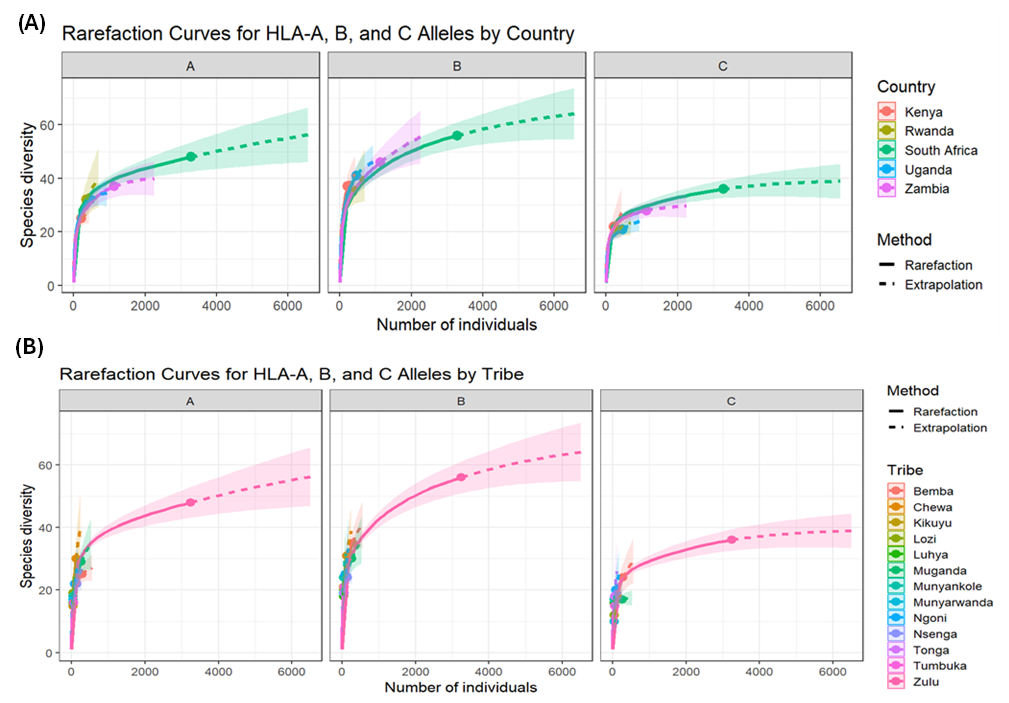


**Supplementary figure 4|** Rarefaction curves by HLA gene and populations estimating the allelic diversity or richness. The figure is a rarefaction curve with bootstrap sampling of size 50 and 95% confidence interval for alleles at different loci in the different populations. It’s a curve of the number of unique alleles against number of participants which is also a form of diversity measures. The steeply rising rarefaction curves across different loci for each population indicate that the current sampling has not yet captured the full extent of HLA allele diversity. This trend suggests that additional, possibly rare alleles are likely to be discovered with increased sampling effort. If the curve plateaus, it indicates that the sampling effort was sufficient to capture most of the allele diversity at that locus for the population. The Shaded areas represent the 95% confidence intervals around the diversity estimates. Wider intervals suggest greater uncertainty, usually due to smaller sample sizes. The dashed lines show extrapolated diversity — i.e., predictions about what the diversity would be if more individuals were sampled.


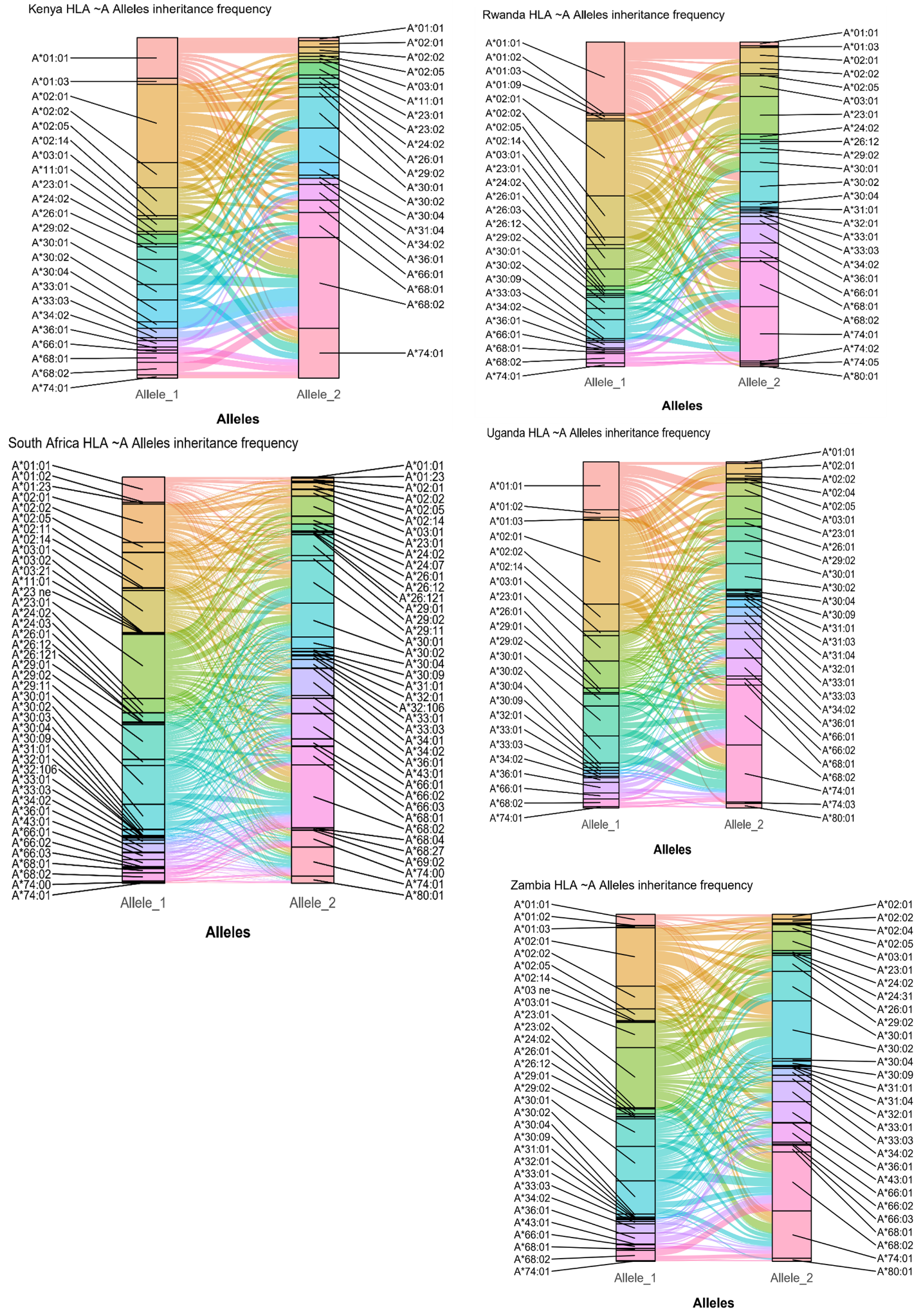


**Supplementary figure 5|** plots showing frequency how HLA ~A alleles were inherited together by participants in each country.


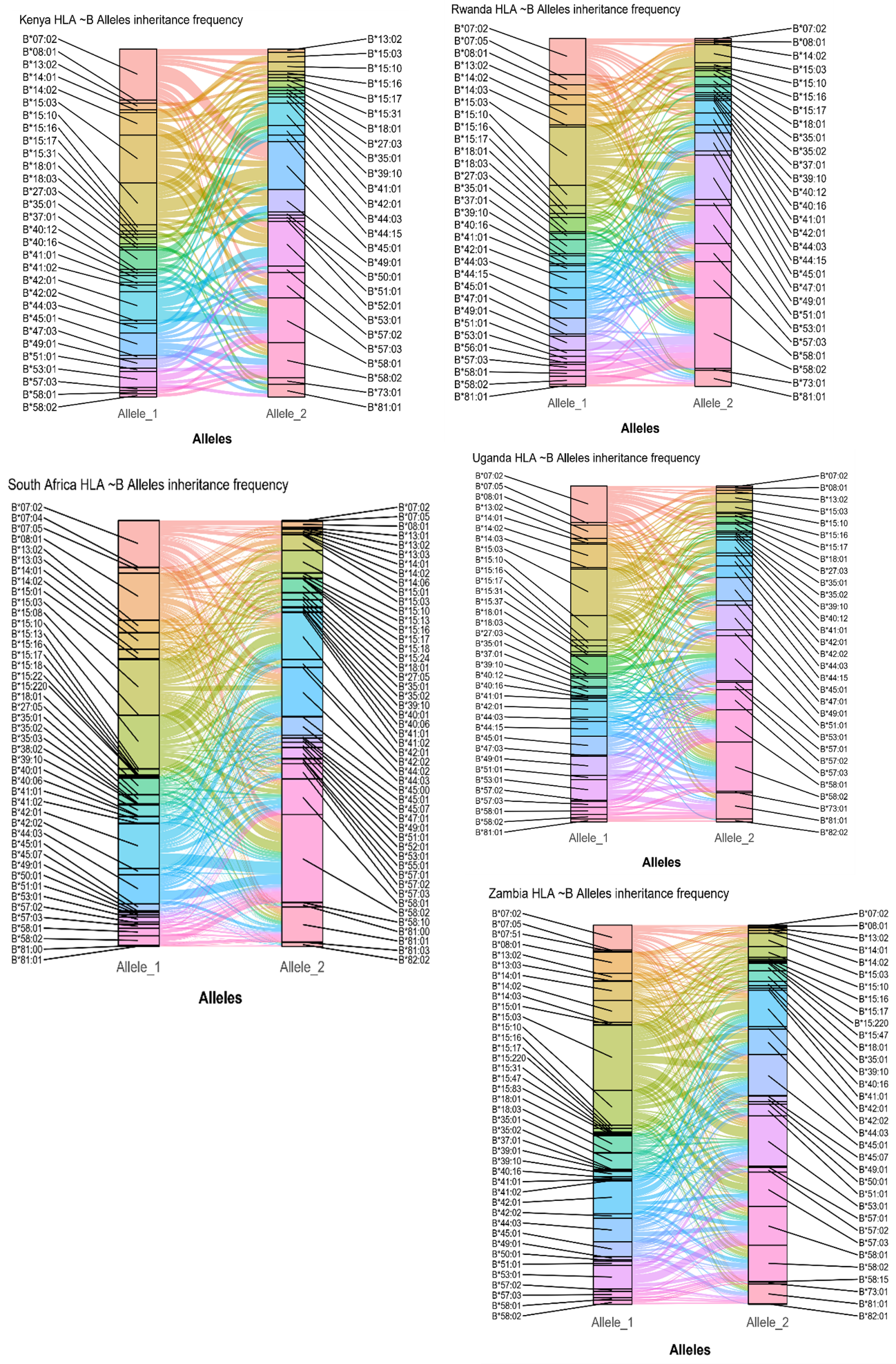


**Supplementary figure 6|** plots showing frequency how HLA ~B alleles were inherited together by participants in each country


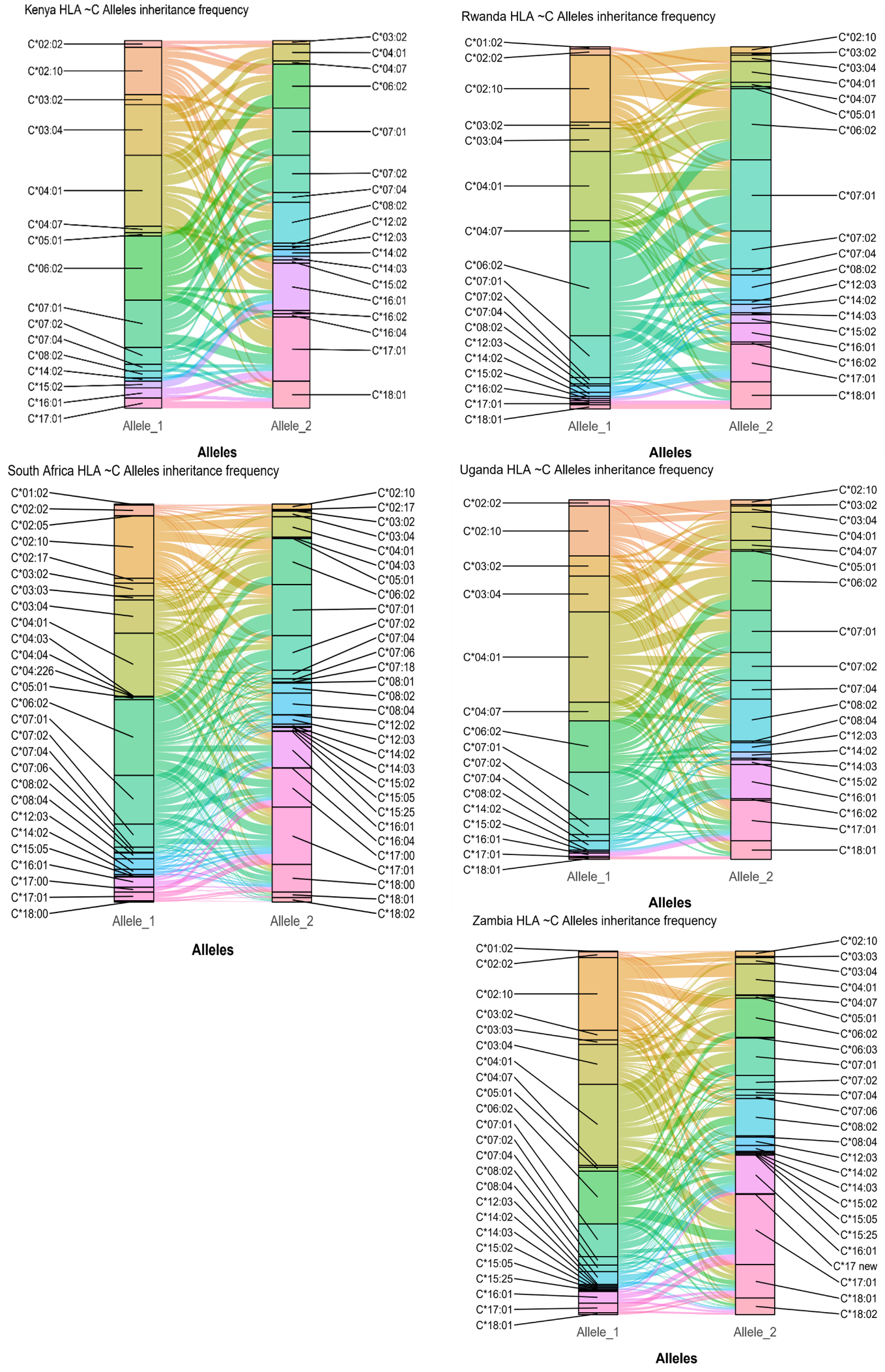


**Supplementary figure 7|** plots showing frequency how HLA ~C alleles were inherited together by participants in each country


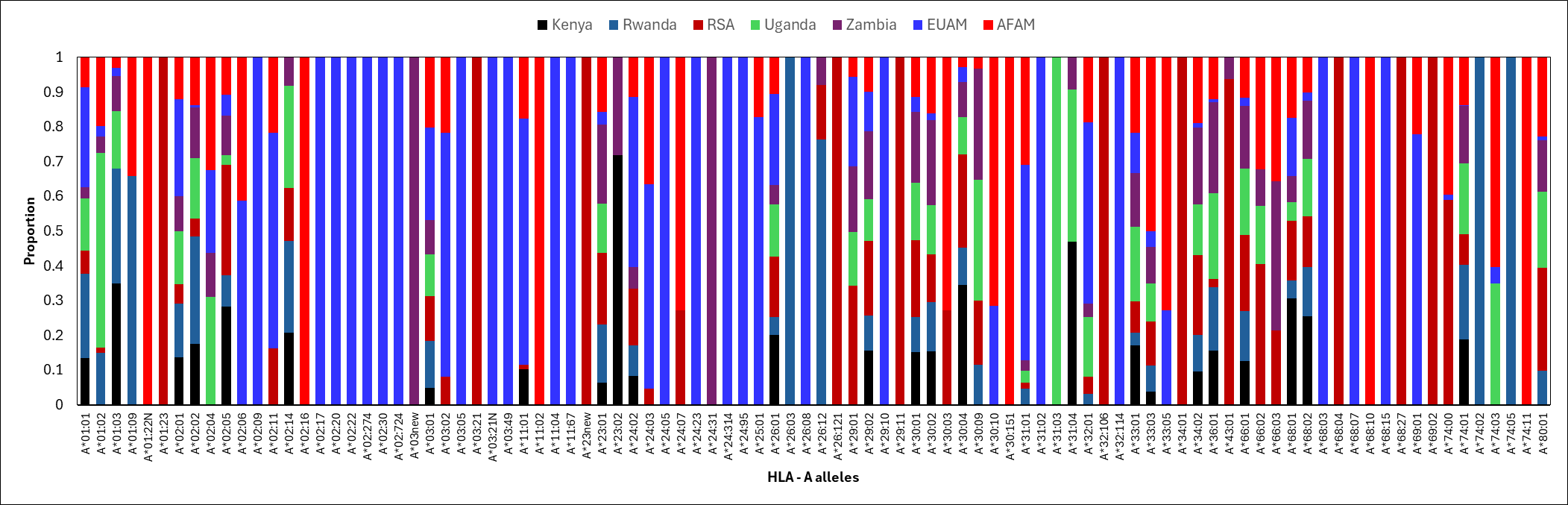


**Supplementary figure 8|** Stacked bar plot for proportion of HLA – A alleles across populations


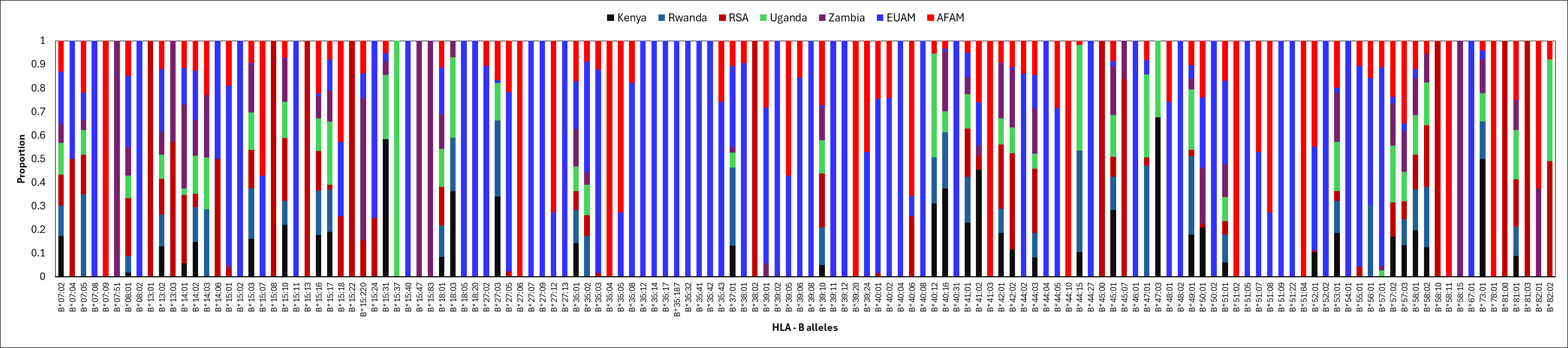


**Supplementary figure 9|** Stacked bar plot for proportion of HLA – B alleles across populations


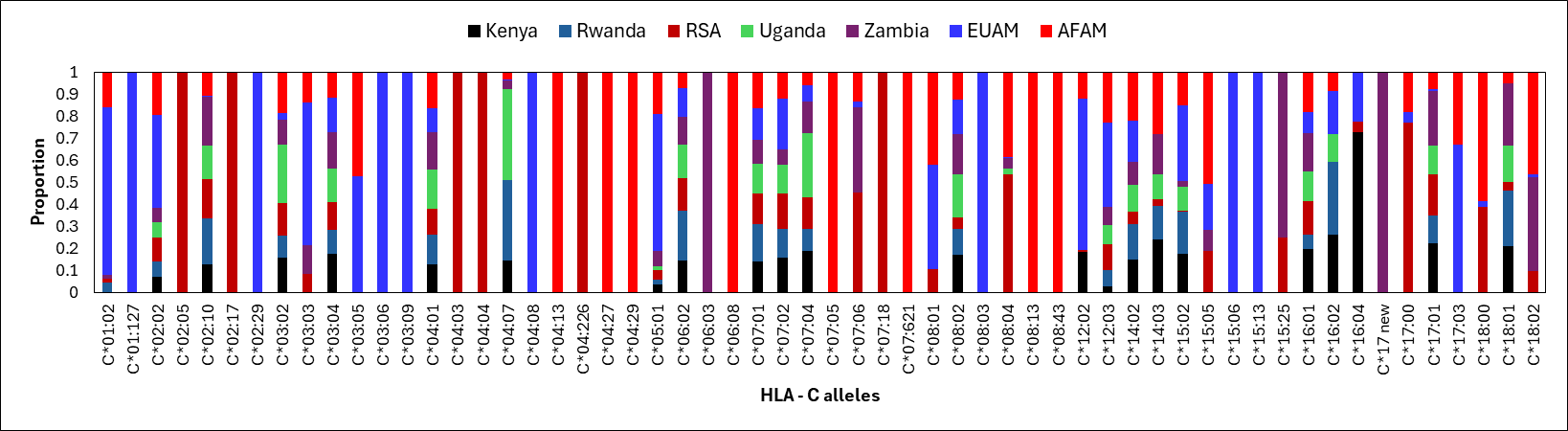


**Supplementary figure 10|** Stacked bar plot for proportion of HLA – C alleles across populations


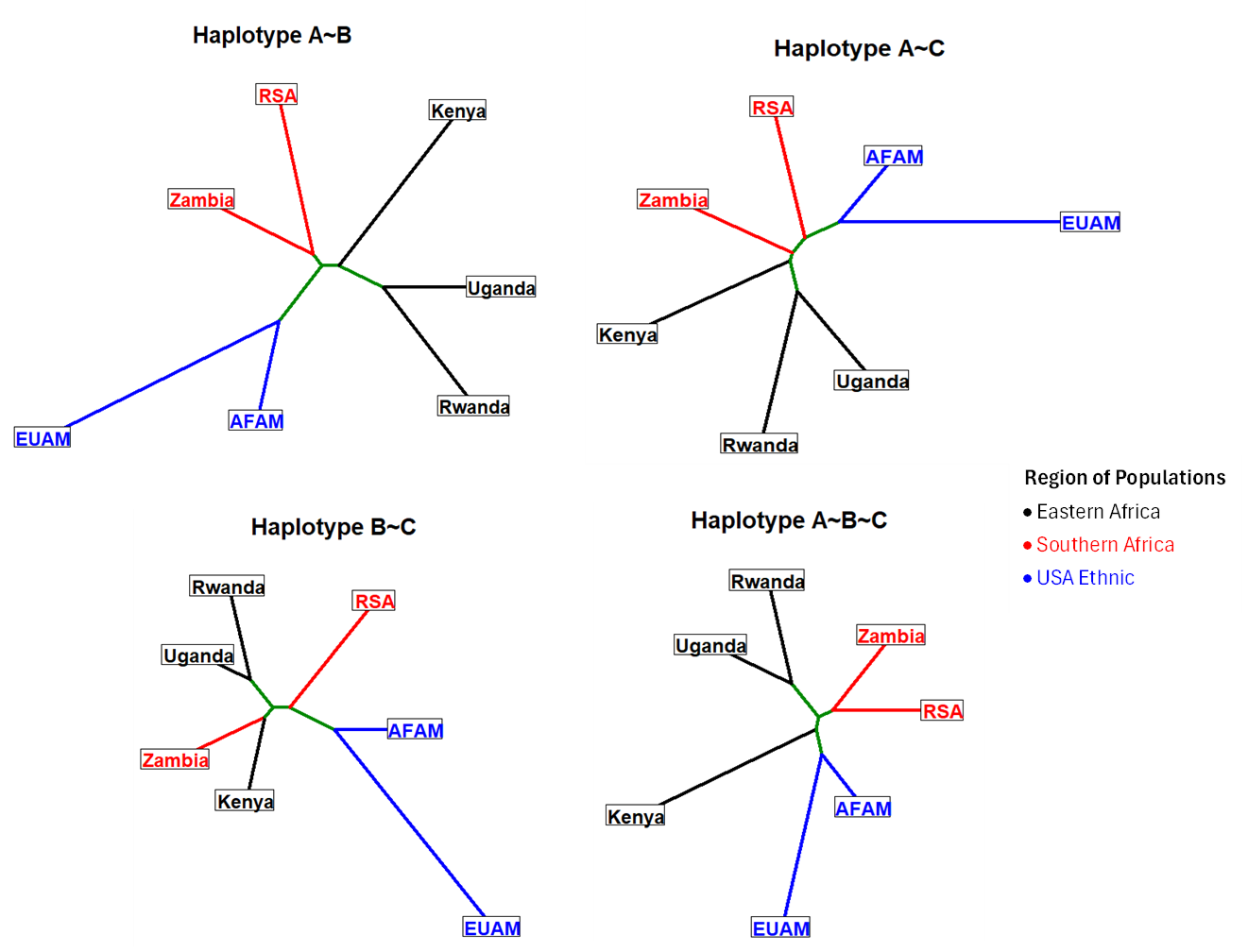


**Supplementary figure 11|** Unrooted phylogenetic trees of haplotypes among populations
